# Multistability and Long-Timescale Transients Encoded by Network Structure in a Model of *C. elegans* Connectome Dynamics

**DOI:** 10.3389/fncom.2017.00053

**Published:** 2017-06-13

**Authors:** James M. Kunert-Graf, Eli Shlizerman, Andrew Walker, J. Nathan Kutz

**Affiliations:** ^1^Department of Physics, University of WashingtonSeattle, WA, United States; ^2^Department of Applied Mathematics, University of WashingtonSeattle, WA, United States; ^3^Department of Electrical Engineering, University of WashingtonSeattle, WA, United States

**Keywords:** multistability, transient dynamics, nonlinear networks, *C. elegans*, bifurcations

## Abstract

The neural dynamics of the nematode *Caenorhabditis elegans* are experimentally low-dimensional and may be understood as long-timescale transitions between multiple low-dimensional attractors. Previous modeling work has found that dynamic models of the worm's full neuronal network are capable of generating reasonable dynamic responses to certain inputs, even when all neurons are treated as identical save for their connectivity. This study investigates such a model of *C. elegans* neuronal dynamics, finding that a wide variety of multistable responses are generated in response to varied inputs. Specifically, we generate bifurcation diagrams for all possible single-neuron inputs, showing the existence of fixed points and limit cycles for different input regimes. The nature of the dynamical response is seen to vary according to the type of neuron receiving input; for example, input into sensory neurons is more likely to drive a bifurcation in the system than input into motor neurons. As a specific example we consider compound input into the neuron pairs PLM and ASK, discovering bistability of a limit cycle and a fixed point. The transient timescales in approaching each of these states are much longer than any intrinsic timescales of the system. This suggests consistency of our model with the characterization of dynamics in neural systems as long-timescale transitions between discrete, low-dimensional attractors corresponding to behavioral states.

## 1. Introduction

The complex structure of neuronal networks must be designed to balance a variety of competing factors. Such networks must robustly respond to a wide range of inputs with a broad variety of output behaviors, while also approximately minimizing constraints, such as their overall wiring cost (Chen et al., 2006; Bullmore and Sporns, 2012). It is no surprise, then, that the connectivities of such networks are typically far from random (Sporns, 2011). Despite their inherent complexity, the dynamical patterns of activity generated by neuronal networks are often fundamentally low-dimensional. That is, the robust functional responses and behavioral assays are characterized by low-dimensional attractors or transient trajectories between them (Gold and Shadlen, 1999; Laurent et al., 2001; Rabinovich et al., 2001, 2008; Jones et al., 2007; Rabinovich and Varona, 2011; Stephens et al., 2011; Kunert et al., 2014; Shlizerman et al., 2014).

Computational modeling can serve to establish the role which network connectivity may play in encoding dynamic responses, as opposed to the intrinsic dynamics of individual nodes or additional biochemical factors. This can be accomplished through modeling efforts which approximate all neurons in a complex network as identical, distinguished only by their connectivity. For example, Gollo et al. (2015) analyzed a model of the macaque cortex which assigned all neurons the same characteristic timescales, and showed that a hierarchy of timescales could arise through the network structure alone.

The nematode *Caenorhabditis elegans* is an important model system for these computational modeling efforts (Izquierdo and Beer, 2016), partly due to the fact that the connectivity between its 302 neurons (its “connectome”) has been resolved (White et al., 1986; Varshney et al., 2011). While the exact role of the connectome in neuronal computation remains unresolved and controversial, it has been shown that simple computational models of *C. elegans* neural dynamics (combining specific connectivity data with simple unfit parameter estimates and dynamics, while approximating all neurons as identical) are capable of generating non-trivial, qualitatively correct responses to given stimuli. For example, Kunert et al. (2014) found a neural proxy for behavior consisting simply of a single limit cycle within the system, similar to the low-dimensional behavioral dynamics observed in Stephens et al. (2008).

In this paper, we explore the input space of model for the neuronal network dynamics of *C. elegans* developed in Kunert et al. (2014), and find that various multistabilities can arise in response to inputs. Specifically, we survey all inputs corresponding to single-neuron current injections and generate bifurcation diagrams showing the existence limit cycles or fixed points at a given input level. When an input drives the system into a multistable regime, simulated transient dynamics are seen to be much slower (on the order of seconds to tens of seconds) than any intrinsic neuronal timescales (which in our model do not exceed a few 100 ms). This helps to support recent biophysical conjectures that the transients themselves are critical in understanding behavioral assays (Rabinovich and Varona, 2011). The transient trajectories themselves are low-dimensional and could potentially be associated with network-produced functionalities, such as neural proxies for movement.

As a first particular example, we investigate input into the PLM neuron pair, which is known experimentally to excite forward motion (Chalfie et al., 1985) and within our model creates a two-dimensional limit cycle response (Kunert et al., 2014). We then use the low-dimensional PLM response plane to consider the dynamics of a compound input vector PLM + ASK, where ASK stimulation is known to facilitate transitions (i.e., turns Gray et al., 2005). Our bifurcation analysis reveals that this induces bi-stability, in which the system goes either into a fixed point or a limit cycle. Transient timescales are shown to be considerably longer in this bistable case than the intrinsic timescales of the system. This allows for long timescales in the system in the presence of discrete, low-dimensional attractors corresponding to behavioral states, consistent with the experimentally-based framework of Stephens et al. (2011). This further supports the perspective of Gollo et al. (2015) that connectivity structure alone is capable of generating a hierarchy of slow timescales. This input scenario demonstrates how our bifurcation analysis methodology prescribes a generic approach for identifying multi-stable states and their transient timescales in response to arbitrary inputs. Since we model neurons as identical save for their connectivity, it further indicates that their connectivity alone can encode the creation and destruction of multiple behavioral attractors.

## 2. Materials and methods

### 2.1. Model for coupled neural dynamics

The dynamic model used is constructed to represent the graded responses of the neurons of *C. elegans*. Experiments show that many neurons in the organism are nearly isopotential, such that it is a reasonable approximation to model neurons as single compartments with membrane voltage as a state variable for the neurons (Goodman et al., 1998; Lockery and Goodman, 2009). Wicks et al. (1996) used this to construct a single-compartment membrane model for neuron dynamics. Building on this, Kunert et al. (2014), constructed a full connectomic dynamics model which was shown to yield reasonable low-dimensional neural proxies for known behavioral responses (specifically, it was shown that simulating excitation of the tail-touch mechanosensory pair PLM creates a two-mode oscillatory limit cycle in the body-wall motorneurons). As in Kunert et al. (2014), neural membrane voltage dynamics are governed by:

(1) $$C\dot{V}=-G^{c}(V_{i}-E^{cell})-I_{i}^{Gap}(\vec{V})-I_{i}^{Syn}(\vec{V})+I_{i}^{Ext}$$

*C* is the whole-cell membrane capacitance, *G*^*c*^ is the membrane leakage conductance and *E*^*cell*^ is the leakage potential. The external input current (which we change to specify the external stimulus) is given by $I_{i}^{Ext}$, while neural interaction via gap junctions and synapses is modeled by input currents $I_{i}^{Gap}\left(\vec{\text{V}}\right)$ (gap) and $I_{i}^{Syn}\left(\vec{\text{V}}\right)$ (synaptic). Their equations are:

(2) $$I_{i}^{Gap}=\sum_{j}G_{ij}^{g}(V_{i}-V_{j})$$

(3) $$I_{i}^{Syn}=\sum_{j}G_{ij}^{s}s_{j}(V_{i}-E_{j})$$

Gap junctions are taken as ohmic resistances connecting each neuron where $G_{ij}^{g}$ is the total conductivity of the gap junctions between *i* and *j*. Synaptic current is proportional to the displacement from reversal potentials *E*_*j*_. $G_{ij}^{s}$ is the maximum total conductivity of synapses to *i* from *j*, modulated by the synaptic activity variable *s*_*i*_, which is governed by:

(4) $${\dot{s}}_{i}=a_{r}\phi (\beta (V_{i}-V_{i}^{th}))\cdot (1-s_{i})-a_{d}s_{i},$$

where *a*_*r*_ and *a*_*d*_ correspond to the synaptic activity's rise and decay time, and ϕ is the sigmoid function ϕ(*x*) = 1/(1 + exp(−*x*)), set here with width β and center $V_{i}^{th}$. Solving Equation 4 for its equilibrium value at $\dot{s_{i}}=0$ yields:

(5) $${\dot{s}}_{i}=0\Rightarrow s_{i}=\frac{\phi (\beta (V_{i}-V_{i}^{th})+\ln(1+a_{r}/a_{d}))}{\left(1+a_{d}/a_{r}\right)}.$$

Thus the equilibrium value of *s*_*i*_ depends sigmoidally upon the membrane voltage *V*_*i*_. As in equation 3, the synaptic current $I_{i}^{Syn}$ into neuron *i* depends upon the values of *s*_*j*_ for all presynaptic neurons *j*, and *s*_*j*_ depends directly upon *V*_*j*_. Thus, $I_{i}^{Syn}$ depends directly upon the membrane voltages of presynaptic neurons.

### 2.2. Model parameters

We keep the parameters values of Kunert et al. (2014), as summarized in Table 1. In particular, the connectivity parameters $G_{ij}^{g}$ and $G_{ij}^{s}$ are prescribed by the full connectome (Varshney et al., 2011). The relative significance of these specific connectivity values is maintained by not fitting any of the other global parameters. Instead, these parameters are estimated to a reasonable order of magnitude from the literature and assumed equal for each neuron. Specific parameter values are as follows, as taken from Kunert et al. (2014): gap junctions and synapses are both given individual conductances of *g* = 100pS; the synaptic sigmoid width parameter is set to β = 0.25 *mV*^−1^; cell membranes are set to a conductance of *G*^*c*^ = 10pS and given a leakage potential of *E*^*cell*^ = −35 *mV*; and membrane capacitances are set to 1pF. All neurons are modeled as identical except for their connectivity and the assignment of them as excitatory or inhibitory (where *E*_*j*_ is set as 0mV for excitatory neurons and −45mV for inhibitory neurons). Note, however, that this necessarily means that any functionality which depends upon the precise electrophysiological properties of individual neurons and connections will not be captured by this model.

**Table 1:** Parameter values assigned within the model.

**Parameters (from Kunert et al., 2014)**	**Value**
Membrane conductance *G*^*c*^	10 pS
Membrane capacitance *C*_*H*_	1 pF
Leakage potential *E*_*c*_	−35 mV
Gap junction conductivity *g*	100 pS
Synaptic conductivity *g*	100 pS
Reversal potential *E*_*j*_ (Excitatory)	0 mV
Reversal potential *E*_*j*_ (Inhibitory)	−45 mV
Sigmoidal width β	0.125 mV^−1^
Synaptic rise constant *a*_*r*_	1 s^−1^
Synaptic decay constant *a*_*d*_	5 s^−1^

Sigmoid centers $V_{i}^{th}$ are set as in Kunert et al. (2014) and similar to Wicks et al. (1996): Neurons are assumed to have $\phi \left(\beta \left(V_{i}^{eq}-V_{i}^{th}\right)\right)=1/2$ at equilibrium voltages $V_{i}^{eq}$. Using this condition with Equations 1 and 4 allows us to solve for both the values of $V_{i}^{th}$ and $V_{i}^{eq}$, which we call the “standard” equilibrium. Note that this equilibrium always exists, though is not necessarily the only fixed point within the system, nor is it always stable.

### 2.3. Modeling neurons as identical units

The model does not include various extra-synaptic features known to drive or regulate responses. For example, there is evidence that self-sustained forward locomotion in *C. elegans* is regulated by proprioception within motor neurons (Wen et al., 2012) (compare how our model, lacking this, does not sustain oscillation in the absence of explicit external input). Computational modeling which includes stretch-receptive proprioception shows that such feedback loops can control behavioral features, such as gait modulation between differing environments (Bryden and Cohen, 2008; Boyle et al., 2012). The lack of such feedback mechanisms and other signaling mechanisms (such as various neuromodulators, monoamines, and peptides, Vidal-Gadea et al., 2011; Qi et al., 2013), in combination with the simple neuron model and parameter assumptions, mean that specific responses to given inputs seen within the model can be encoded only within the network's connectivity. However, it should also be noted that these and other effects are highly important, and any functionality which depends on such effects will not be captured by this model. However, this reductive approach yields information as to how behavioral responses could be encoded within the structure of the connectome.

### 2.4. Model timescales

Of particular relevance to this paper are the timescales within the system. From the first term in Equation (1), we see that the exponential free decay constant of an unconnected neuron (i.e., decay through the membrane leakage term alone, with $I_{i}^{Gap}=I_{i}^{Syn}=I_{i}^{ext}=0$) would be $\tau_{free}=C/G^{c}=100\text{ms}$. Similarly, the time constant value given by gap junctions would be τ_*gap*_ = *C*/*g* = 10 ms.

There are also timescales intrinsic to the synaptic dynamics. We approximate these by considering the dynamics when voltages are held constant, and thus $\phi \left(\beta \left(V_{i}-V_{i}^{th}\right)\right)\equiv \phi_{i}$ is constant. Then Equation (4) becomes:

(6) $${\dot{s}}_{i}=a_{r}\phi_{i}-(a_{r}\phi_{i}+a_{d})s_{i}$$

and thus the synapses will exponentially approach equilibrium with a time constant of τ_*syn*_ = 1/(*a*_*r*_ϕ_*i*_ + *a*_*d*_). Since $a_{r}={1\text{s}}^{-1}$, $a_{d}={5\text{s}}^{-1}$, and ϕ_*i*_ ∈ (0, 1), synapses must have exponential time constants in the range τ_*syn*_ ∈ (166, 200)ms.

This collection of timescales is summarized in Table 2. It will be shown that, when the system is in a bistable regime, the timescales of transient dynamics within the system can be orders of magnitude above any of these intrinsic time constants within the system (on the order of 10s, for example).

**Table 2:** Orders of magnitude for various timescales within the system for the parameters chosen.

**Interaction**	**Timescale**
Single-neuron membrane leakage	100 ms
Gap junctions	10 ms
Synaptic connections	200 ms

### 2.5. Response to PLM stimulation: defining the low-dimensional projection

Kunert et al. (2014) found that stimulating the tail-touch mechanosensory neuron pair PLM within this model, gives rise via a bifurcation to a limit cycle within the forward-motion motorneurons. Experimentally, stimulation of PLM drives forward motion (Leifer et al., 2011; Stirman et al., 2011). This limit cycle consists of only two modes, which together account for 99.3% of the variance in motorneuron activity. This is in agreement with the behavioral observation that the worm's body shape during forward motion is well-described by a similar two-mode oscillation (Stephens et al., 2008). The non-triviality of this agreement was established by showing that simulated ablation studies affected this response in agreement with experimental ablation studies (e.g., ablation of the AVB interneurons destroys the response both experimentally and in the model, Chalfie et al., 1985).

**Figure 1:**
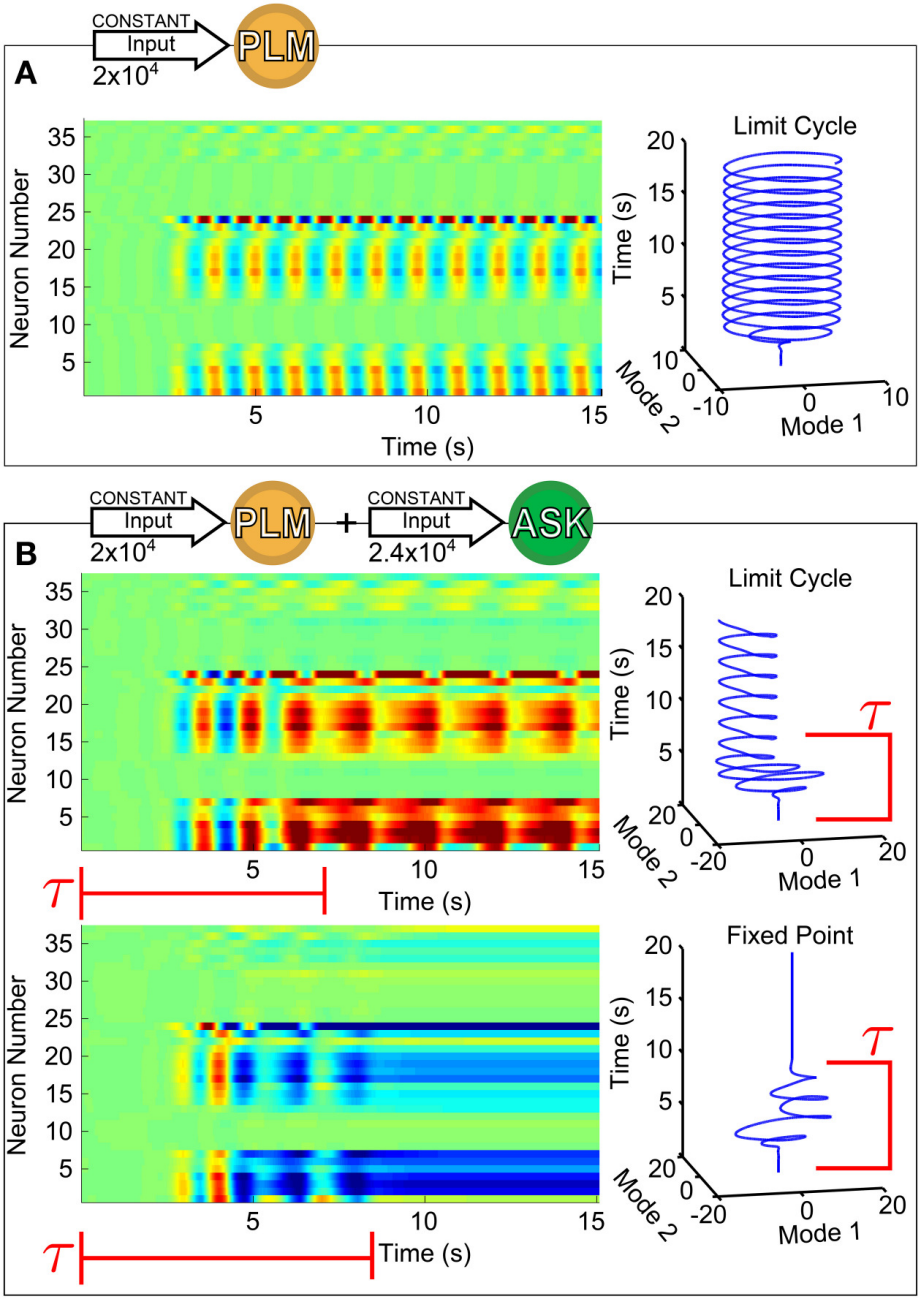
Voltage dynamics of forward-motion motorneurons (neurons of classes DB, DD, VB, and VD) in response to the following sensory inputs: in **(A)**, an input of 2 × 10^4^ (Arb. Units) into the PLM sensory neuron pair (known experimentally to drive forward motion Chalfie et al., 1985); in **(B)**, an input of 2 × 10^4^ into the PLM pair with an additional input of 2.4 × 10^4^ into the ASK sensory neuron pair (known experimentally to promote turning Gray et al., 2005). Simultaneous PLM + ASK stimulation causes bistability, with relatively long transient times τ. To the right of each raster plot is the trajectory within the Forward-Motion 2D Plane (defined by the trajectory in **(A)**, and used for all subsequent projections).

Figure 1 shows the response of forward motion motorneurons to various inputs as a function of time. Figure 1A of the figure shows a raster plot of motorneuron voltages in response to PLM input [through the *I*^*Ext*^ term in Equation (1)], for which the two-mode oscillatory response can be observed (Kunert et al., 2014). The trajectory of these two leading modes are plotted as a function of time on the right. We use this same low-dimensional space (defined as the two forward-motion motorneuron modes which oscillate during PLM activation) throughout the paper. In other words, we use the same projection for the low-dimensional trajectories in Figure 1B and in all further figures.

Specifically, we calculate this plane (as in Kunert et al., 2014) by taking time snapshots of forward-motion motorneuron membrane voltages ${\vec{V}}_{M}\left(t\right)$, collecting them into a matrix *V* (the matrix plotted in the raster plot of Figure 1A, and taking that matrix's singular value decomposition. That is:

(7) $$V=[{\vec{V}}_{M}(t_{0}),{\vec{V}}_{M}(t_{1})\ldots ]=P\cdot \Sigma \cdot \;Q^{T},$$

where *P* and *Q* are unitary and Σ is diagonal. The columns of *P* are the principal orthogonal modes. As in Kunert et al. (2014), the first two of these modes (the first two columns of *P*) almost entirely capture the dynamics of the system within this subspace under constant PLM stimulation. Projection of the full-system dynamics onto this plane consists of projecting the system's motorneuron dynamics onto these modes.

Importantly, all of the neuron membrane voltages which have a nonzero projection onto this plane are forward-motion motorneurons (that is, motorneurons of class DB, DD, VB, and VD). All neurons outside of these classes (and thus all sensory and interneurons) belong to the null space of this projection; that is, only motorneuron dynamics project onto this plane. All low-dimensional trajectories within this study can therefore be understood as corresponding to motorneuron dynamics, aiding in their potential biological interpretability (in the sense that motorneuron dynamics should map onto muscular dynamics).

This specific two-dimensional plane represents the dominant motorneuron neural response modes for PLM stimulation. Each stable fixed point within this plane corresponds to a static neural response, whereas a limit cycle corresponds to an oscillatory trajectory. The exact mapping of these neural modes onto muscular activities remains ambiguous, but it is plausible that a fixed point in body-wall motorneuron activity could correspond to fixed muscular postures, whereas oscillatory body-wall motorneuron activity could drive periodic body-shape motion (via an undefined mapping). Inter- and motorneurons do, indeed, display low-dimensional dynamical trajectories; however, there is generally insufficient biophysiological evidence for interpreting any behavioral implications of such dynamics. States which we identify in our projection plane, on the other hand, indicate possible neural proxies for low-dimensional body movements in *C. elegans*.

## 3. Results

### 3.1. Existence of multistable dynamics

The response to PLM stimulation alone consists of a single possible state (i.e., a limit cycle trajectory), but if the model is capable of describing the dynamics in terms of long-timescale transitions between states under the same input, then we wish to find inputs which allow multiple states and transitional dynamics. We find that such inputs indeed exist. As an example, we consider the response to simultaneous stimulation of the PLM neuron pair along with the ASK neuron pair. We choose this stimulation since excitation of ASK neurons have been shown experimentally to promote turning (Gray et al., 2005) and their ablation greatly increases the duration of periods of forward motion (Wakabayashi et al., 2004).

As we show in Figure 1B, for this combined input there coexist two different attractors, i.e., the system is bistable. The two trajectories plotted are in response to the same constant input amplitudes into PLM and ASK, and differ only by their initial conditions. Note that the transients before convergence into the eventual fixed point or limit cycle have long timescales (relative to the intrinsic timescales of the system as discussed in Section 2.4). The model therefore does exhibit multistability for this given input, but given the large dimensionality of the input space, the discovery, identification and interpretation of these multistable regimes is not trivial. Since we wish to understand the neural dynamics as consisting of long-timescale transients in the presence of multiple discrete attractors, we develop a method for (1) identifying the existence and nature of attractors in response to arbitrary inputs, (2) characterizing transient timescales, and (3) providing interpretable biophysical meaning to calculated trajectories via projection onto a meaningful low-dimensional space.

### 3.2. Bifurcation diagrams for state identifications

Motivated by observational studies which describe *C. elegans* behavioral dynamics in terms of low-dimensional attractor dynamics (Stephens et al., 2011), we wish to understand our simulated neural dynamics in the presence of multiple discrete attractors. Numerical bifurcation analysis can be useful in revealing the states existing within high-dimensional neural models (see e.g., Laing, 2014). We therefore propose to construct bifurcation diagrams, which show the attractors which exist within the system under arbitrary inputs. By fixing the direction of the input vector *I*^*Ext*^ in Equation (1) and using its amplitude as our bifurcation parameter, such diagrams will show us at a glance the set of states created in response to a given input, and provide us with a method of identifying induced multistability.

**Figures 3, 4** show examples of such bifurcation diagrams, in which we plot the furthest *L*^2^ distance from standard equilibrium (within the 2D Forward-Motion Plane) of attractors which are present as a function of input amplitude. Full detail on the algorithm used to generate such diagrams can be found in the Supplementary Materials. Our exploratory method has a few implications for the interpretation of these diagrams: first, we search only for fixed point or limit cycle attractors (and not, e.g., chaotic attractors); second, there is no theoretical guarantee that we find *all* possible states of the system, such that others could exist; third, the fixed point/limit cycle convergence criteria are defined for the full set of neurons, such that when the motorneurons are in a fixed point (or limit cycle), this is also true of the sensory and interneurons.

We generated these diagrams for all 279 of the single-neuron inputs into the system. Note that the figures from these simulations, as shown in the Supplementary Materials, are done over a much coarser range than those in **Figures 3, 4**. The purpose of these coarse figures is to quickly give an indication of the likely number of states for each range of inputs. Thus, these diagrams give a means of identifying what attractors will exist within the system for a broad range of arbitrary inputs, and of easily identifying regions of multistability in the input space. This set of diagrams is included within the Supplementary Materials.

Note that some care must be taken in interpreting these figures as they give only the response to stimulation of a single neuron. Notably, compound inputs may lead to qualitative differences between the corresponding single-input diagrams. Compare, for instance, the bifurcation diagrams for PLML + PLMR (as in **Figure 3**) and for PLML + PLMR + ASKL + ASKR (as in **Figure 4**) with the diagrams of the constituent neurons (as within the Supplementary Materials). However, it is no more difficult to generate the diagram of any compound input than it is for a single input. Any combination of constant-in-time inputs defines a different constant vector, the magnitude of which is our bifurcation parameter; however, sparsity of this vector is not a factor in any of our calculations. Thus, our algorithm prescribes a method for exploratory bifurcation diagram generation even for complex, many-neuron inputs.

**Figure 2:**
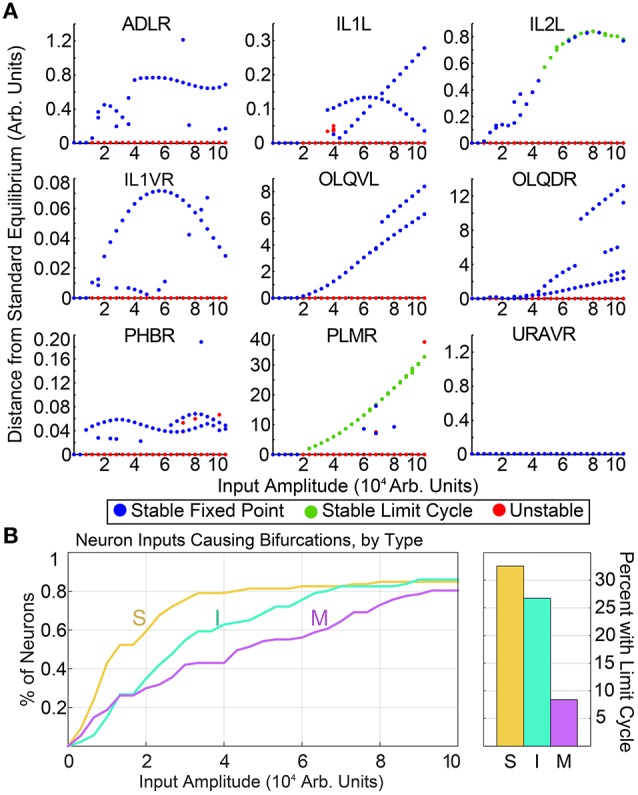
**(A)** shows a selection of bifurcation diagrams for inputs into different neurons, showcasing the variety of multistable behaviors within the system. Similar diagrams for all neurons are included in the Supplementary Materials. **(B)** shows the input amplitude of the first bifurcation, by neuron type. The vertical axis shows the percentage sensory neurons, interneurons, and motorneurons for which the standard equilibrium is unstable at the corresponding input amplitude. On average, sensory neurons drive bifurcations at a lower input amplitudes than inter- or motorneurons. Motorneurons are much less likely to drive limit cycles within the system.

Generating these diagrams for all possible single inputs allows for the qualitative comparison of features within each neuron's bifurcation diagram. Similar features in the bifurcation diagrams of neurons may suggest similar functionalities. As a simple example, in Figure 2, we compare the input amplitude at which the standard equilibrium first becomes unstable for sensory neurons, interneurons and motorneurons. The majority of sensory neurons are seen to drive bifurcations in the system at lower input levels than for most interneurons, which in turn require lower inputs than most motorneurons. Intuitively, this suggests that the system is typically more sensitive to input into sensory neurons than it is to interneuron or motorneuron inputs. Furthermore, for each group of neurons we compute the percentage of single neuron inputs which promote limit cycle attractors. We find that within our input range, 32.6% of sensory neurons and 26.7% of interneurons give rise to oscillatory dynamics, whereas only 8.4% of motorneurons result in oscillation when stimulated. This points to the sensitivity and particular ability of sensory neurons to drive complex dynamics within the network.

### 3.3. A defining example: response to PLM input

**Figure 3:**
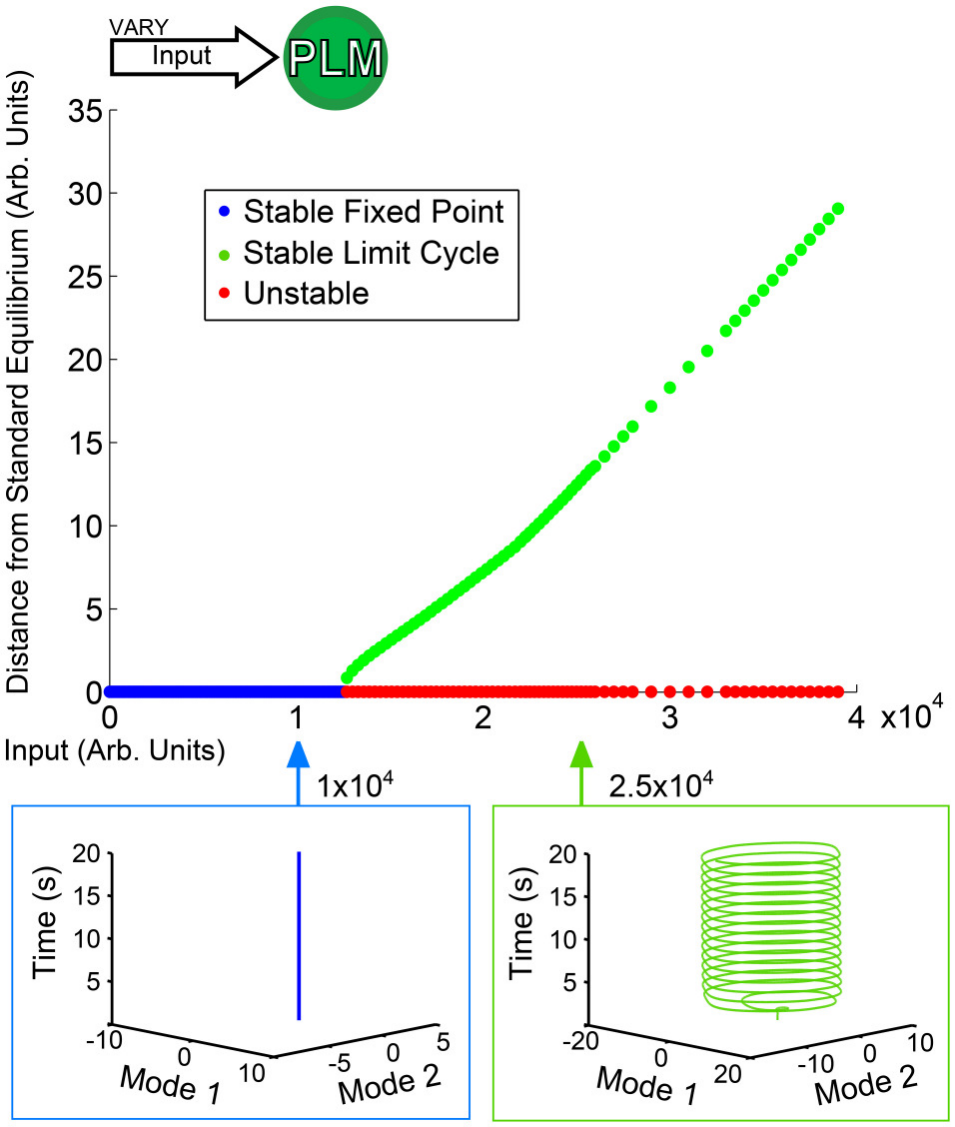
Bifurcation diagram for constant PLM stimulation of varying amplitude. Below an input of 1.2 × 10^4^ the system goes to a stable fixed point very close to the standard equilibrium, but beyond that input level the system goes to a stable limit cycle (where the plotted point gives the furthest distance from standard equilibrium on the limit cycle). The diagram shows the two qualitatively distinct regions of interest for PLM inputs: the low input level in which the system remains at a fixed point, and the higher input level beyond which the system enters into a limit cycle (which in this case can be considered to serve as a proxy for forward motion Kunert et al., 2014).

Figure 3 shows a low-dimensional bifurcation diagram for constant PLM input. The figure shows the creation of a stable limit cycle in response to input into the neurons PLML/R. By evaluating this bifurcation diagram we can identify the regions of interest which have qualitatively distinct responses (in this case, the region with a lone attractor which is a stable fixed point and the second region with a lone stable attractor which is a limit cycle after the fixed point attractor becomes unstable). For each region we can perform simulations which are then projected onto the low-dimensional plane (the PLM limit cycle being what defines this plane). Given the putative correspondence of this limit cycle to forward motion in Kunert et al. (2014), these low-dimensional neuronal trajectories may (via some unknown mapping) map onto bodily dynamics. It is plausible that the fixed point in neuronal dynamics could lead to static bodily movement, or that to oscillatory dynamics of the body-wall motorneurons could map onto oscillatory dynamics of the body of the worm.

### 3.4. Characterizing bistable dynamics

**Figure 4:**
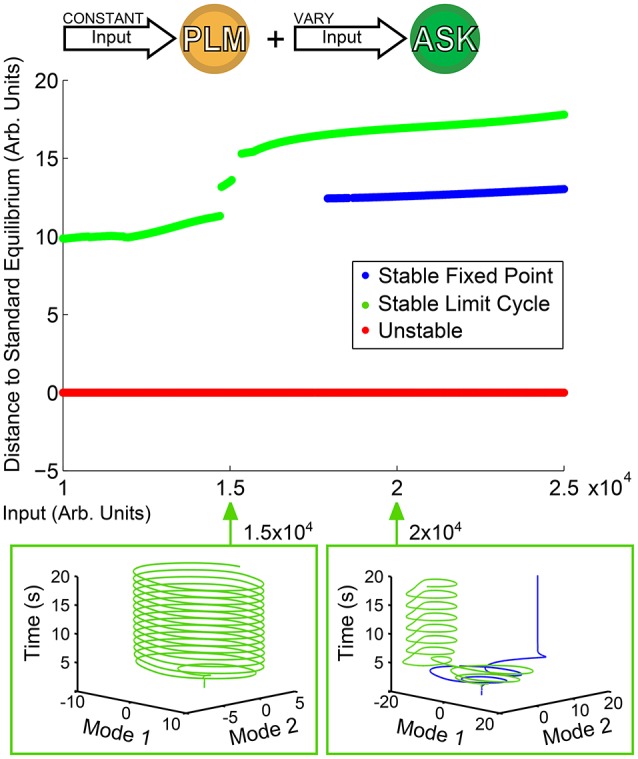
Bifurcation Diagram for varying amplitude of input into the ASK pair. Input into PLM is fixed at 2 × 10^4^. Note that as input into ASK increases, the forward-motion limit cycle remains relatively undisturbed until it reaches about 1.5 × 10^4^, after which the distance jumps and a fixed point becomes stable, giving rise to a bistability within the system.

Of greater interest are responses to compound activations; that is, more complicated inputs leading to more complicated responses. We consider as an example the dual input into the PLM and ASK neuron pairs as discussed in Section 3.1. We keep a constant input of 2 × 10^4^ into the PLM pair and use as our bifurcation parameter the input into the ASK pair. Figure 4 shows the resulting bifurcation diagram. At inputs below 1.5 × 10^4^, the limit cycle remains relatively undisturbed. At greater inputs, however, a series of bifurcations occur such that there is a sudden jump in the distance of the limit cycle, and at about 1.7 × 10^4^ the system becomes bistable with the addition of a new fixed point. Thus, we are able to immediately identify from this figure multistability within the system, which we may then go on to investigate further. Specifically, we are interested in the further investigation of transient timescales of the system.

### 3.5. Long transient timescales

**Figure 5:**
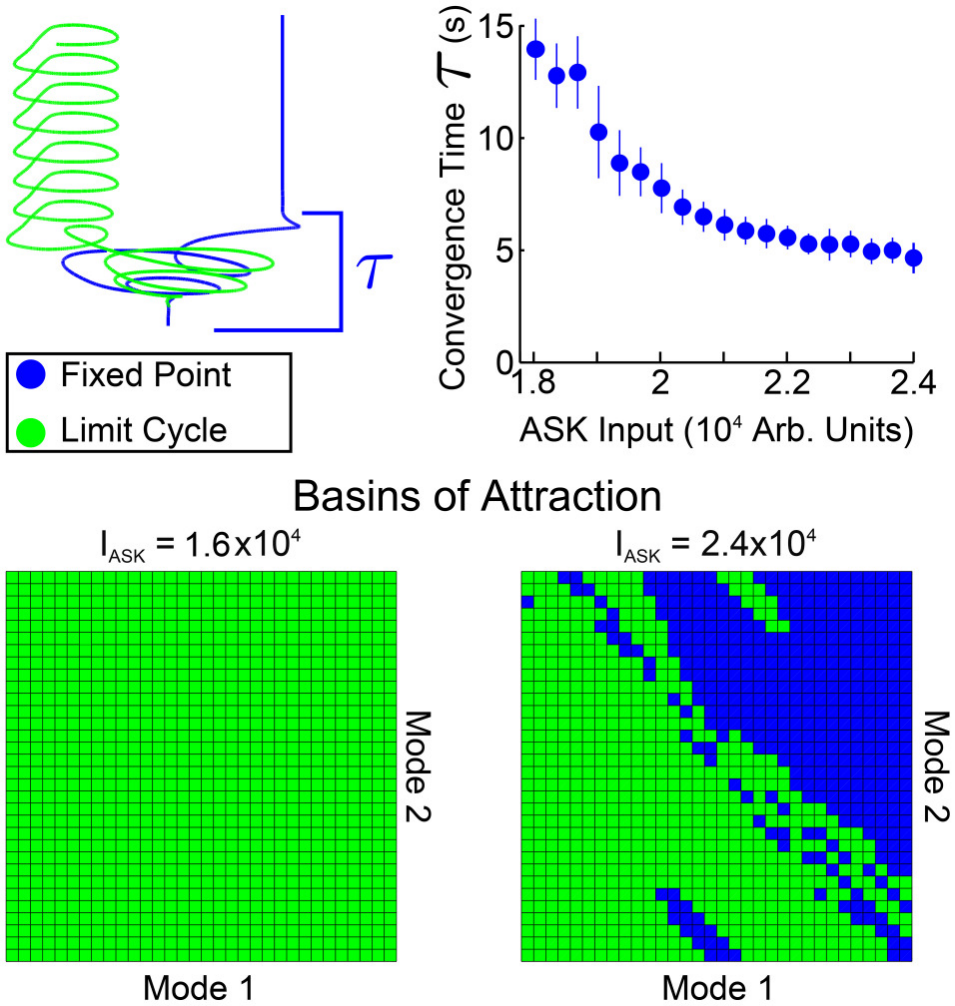
Spatial and temporal properties of convergence for PLM + ASK input (i.e., the bistable region of Figure 4). The upper-right plot shows fixed point convergence times as a function of input amplitude (from 200 trials at each point). Note the relatively long transient timescale. The second row shows the spatial basins of attraction for different inputs. Each grid covers a small region around the standard equilibrium, plotting on (−4, 4) × 10^−6^ for both modes. At an ASK input of 1.6 × 10^4^ all initial conditions converge to a limit cycle, but initial conditions on the plane are split between the limit cycle and fixed points at higher inputs, such as 2.4 × 10^4^.

In Figure 5 we investigate spatial and temporal aspects of the convergence into one of the two bistable states. An ensemble of 200 simulations (with random initial conditions in the neighborhood of the standard equilibrium) were performed for each ASK input level. From those, the trials converging to the fixed point solution were taken and the convergence time τ was calculated by calculating, for each fixed point trial, the time after which all points of the trajectory are within a distance ϵ of the final value (using here ϵ = 0.004). The average and standard deviation of these convergence times are shown in the top right of Figure 5. Convergence times for the limit cycle solutions are qualitatively similar when comparing trajectories, such as those in the upper-left of the figure. Note that these convergence times are considerably longer than other timescales within the system (comparing, for example, the model's free neuron decay constant of 10 ms, Varshney et al., 2011, or other trajectory timescales, such as the limit cycle period, which remains approximately 2 s regardless of ASK input).

Shown also are the basins of attraction for trajectories starting on the low-dimensional plane, on a grid of initial conditions centered at the standard equilibrium point (which we choose as our origin). The size of the grid is chosen to be within a small neighborhood of zero [within the range (−4, 4) × 10^−6^] since we find that trajectories initiated farther away are first attracted toward the zero point before being rerouted to the fixed point or limit cycle attractors.

Note that the basins of attraction in Figure 5 only display this structure when initializing on the plane itself (that is, when displaced from the standard equilibrium point only along the two modes). Displacing the system in the full-dimensional space then projecting the higher-dimensional basins of attraction onto this plane fails to show any clear structure, indicating that the separation of the basins has higher-dimensional structure. Therefore these basin of attraction plots indicate distinct regions in which initial conditions starting on this plane will, under compound PLM + ASK input, go toward either solution (i.e., initializing in the most of the upper-right portion of the plane leads to the fixed point solution, whereas initializing the the lower-left portion of the plane leads to the limit cycle solution). Since this is the plane on which the PLM response limit cycle proceeds, these figures show which portions of this cycle (which goes through both of these regions) are more prone to ASK-driven transitions into a fixed point.

The existence of bistability between a limit cycle and fixed point within our model given simultaneous PLM stimulation (which promotes forward motion) and ASK stimulation (which promotes turning) is thus suggestive of an interpretation in which the worm's motorneuron activity will either, depending on the neural state upon the onset of this stimulus, oscillate, or approach a fixed state.

## 4. Discussion

We explored the input space of a *C. elegans* neural dynamic model which incorporates its fully-resolved connectome and demonstrated that various multistabilities arise in response to inputs. Using a low-dimensional projection space based upon forward motion, we are able to systematically explore responses to complex inputs and understand them in a framework of low-dimensional attractor dynamics. In our study, the bifurcation diagram is constructed by using the constant-in-time input as our bifurcation parameter. We show that such diagrams are capable of revealing and mapping multiple attractors within the system by using a low-dimensional projection space which guides the search for attractors, identifying their stability and their effect upon forward movement. Furthermore, the low-dimensional projection helps in the interpretation of the dynamics upon the discovered attractors, especially the dynamics associated with multistability. We characterize such multistable dynamics, noting specifically that when the system enters into a multistable regime, transient timescales within the system can be very long relative to intrinsic neural timescales (comparing, for example, the three orders of magnitude between the ~100 ms neural timescales in Section 2.4 to the ~10 s transient lengths in Figure 4).

These long transient timescales generated by the multistability of the system have critical implications. These longer timescales, on the order of many seconds, are on a similar order to many behavioral timescales, such as forward crawling survival time (Stephens et al., 2011). This suggests that various behavioral responses could be associated not with the attractor itself, but rather with the transient leading to that attractor. This is consistent with theoretical constructions and experimental observations of transient orbits between attractors (Rabinovich et al., 2001, 2008; Rabinovich and Varona, 2011). Importantly, this viewpoint is supported *independently* and in a completely different theoretical framework by direct connectomic simulations from biophysically appropriate neuron dynamics within the worm, i.e., the multistability of attractors and long-time transients are not engineered in the model to fit the data and observations, rather they naturally arise from the dynamics associated with the connectome. Future work may analyze the structure of perturbations which drive transitions between different states, to predict inputs which result in said behavioral transients.

This study suggests that neural computations can consist of both dynamics on attractors (as in our PLM-driven limit cycle) and of long-timescale transients toward multiple attractors which may arise in the system (as we show in the long-timescale transients approaching the multistable states from PLM + ASK input). We have demonstrated that both dynamical features can arise by applying simple, identical neuron models onto the *C. elegans* connectome data, suggesting that these responses are encoded within the connectome itself. This lends support to the viewpoint of Gollo et al. (2015) that hierarchies of slow neural timescales can emerge from complex network structure alone.

Future work will explore the broader range of dynamics which may be present in the system. It is possible that certain inputs could evoke dynamics in the system besides simple fixed points or limit cycles, such as chaotic dynamics. The discovery of such dynamical regimes in a neuronal dynamics model could yield testable predictions for the types of dynamics evoked in response to given inputs. However, this is closely coupled to another important avenue for future work: the development of more realistic and more directly interpretable models for neuronal dynamics and corresponding behavior. The precise electrophysiological properties as well as neuronal input/output functions have not been fully characterized in *C. elegans*, making the construction of such models difficult, but “in silica” modeling efforts, such as OpenWorm are working to overcome these challenges (Szigeti et al., 2014). The approach within this paper could be applied to such models, the dynamics of which would be both more biophysiologically realistic and more readily interpretable.

More broadly, many networked dynamical systems across the engineering, physical, and biological sciences may also be dominated by patterns of activity and long-time transients induced by the structure of the network architecture. Understanding the basic principles of such behaviors is critical for optimizing performance and controlling deleterious effects. The analysis above may be able to help understand how the network architecture encodes deleterious patterns of activity when combined with relevant dynamics. In contrast, one might desire to generate a network architecture to induce a transient that is beneficial for some purpose relative to an application (for instance, a crawling motion in the case of the *C. elegans*). Understanding how the network connectivity graph drives such activity would be critical for inducing such beneficial patterns of activity, perhaps even suggesting network control protocols for achieving desired results. The theoretical framework presented here highlights the rich and complex dynamics that emerge with networked architectures.

## Author contributions

JMK, ES, and JNK conceptualized the study. JMK and AW wrote the software and performed the simulations. JMK, ES, AW, and JNK contributed to the investigation and formal analysis. ES and JNK supervised and administrated the project. JMK performed the visualization and wrote the original draft. JMK, ES, and JNK contributed to the review and editing of the paper.

### Conflict of interest statement

The authors declare that the research was conducted in the absence of any commercial or financial relationships that could be construed as a potential conflict of interest.
