# Supplementary material for: Multistability and Long-Timescale Transients Encoded by Network Structure in a Model of C. elegans Connectome Dynamics
Source: Front Comput Neurosci. 2017 Jun 13;11:53. doi: 10.3389/fncom.2017.00053 (PMC5468412; doi:10.3389/fncom.2017.00053)
Supplement: Supplementary file 1 [file Presentation1.PDF]

Supplementary Material: Multistability and Long-Timescale Transients encoded by Network Structure in a model of *C. elegans* Connectome Dynamic

James Kunert-Graf [1,*], Eli Shlizerman [2] Andrew Walker [2]. and J. Nathan Kutz [1,2]

*Correspondence:
James Kunert-Graf
kunert@uw.edu

1 GENERATING BIFURCATION DIAGRAMS

1.1 Goals of Algorithm

Numerical bifurcation analysis is a broad and mature field, and a variety of methods exist. Indeed, multiple software packages exist for these purposes and are commonly used, such as MATCONT[1] and AUTO[2]. Furthermore, there exist a variety of more sophisticated approaches which could be readily applied in a similar manner as our method (see, for example, Laing (2014)). Within the scope of this manuscript, however, our goals are twofold:

- Firstly, we wish to broadly characterize fixed points and limit cycles which arise in response to an arbitrary high-dimensional input. Within the scope of this initial exploration, we neglect more exotic dynamical behaviors such as chaotic dynamics which may exist within the system.
- Secondly, we wish to perform these calculations in a conceptually simple way. In particular, our analysis should not be highly contingent upon any particular features of our specific model, as future work should be able to extend these methods to more biophysiologically refined future models of Connectome dynamics (as discussed in the main text).

1.2 Components of Algorithm

For a given input vector into the system, we wish to compute the resulting fixed points and limit cycles of the system in a quick and conceptually simple manner. We accomplish this with a combination of two simple, standard numerical methods: finding fixed points via Newton's method, supplemented with straightforward numerical simulation of the system.

[1] Dhooge A, Govaerts W, Kuznetsov YA. **Matcont: a Matlab package for numerical bifurcation analysis of ODEs**. *ACM Trans. Math. Softw.* 2003, **29**(2):141–164.

[2] Doedel EJ, Champneys AR, Fairgrieve TF, Kuznetsov YA, Sandstede B, Wang X: **Auto97: Continuation and bifurcation software for ordinary differential equations**; 1998

1.2.1 Note on Notation

Within this section, we simplify our notation by concatenating our membrane voltages $\vec{v}$ and synaptic activation variables $\vec{s}$ into a single state vector $\vec{x}$:

$$\vec{x} = \begin{bmatrix} \vec{v} \\ \vec{s} \end{bmatrix}$$

This allows us to write the governing equation of our system as:

$$\frac{\partial \vec{x}}{\partial t} = g(\vec{x}, \mu) \tag{S1}$$

where μ is the set of all parameters of the system (including the input current vector $\vec{I}$).

1.2.2 Newton's Method

Fixed points in the system satisfy the equation:

$$g(\vec{x}, \mu) = 0 \tag{S2}$$

We use the standard technique of Newton's method to find solutions iteratively, starting from some state $\vec{x_0}$. For a given state $\vec{x}$ and set of parameter values μ, we can directly compute the Jacobian matrix J:

$$J_{ij}(\vec{x}, \mu) = \frac{\partial g_i(\vec{x}, \mu)}{\partial x_j} \tag{S3}$$

As per Newton's method, we then move our state approximation towards the fixed point of the linearized system defined by J. Specifically, we iteratively calculate:

$$\vec{x}_{n+1} = \vec{x}_n - h \cdot J(\vec{x}_n, \mu)^{-1} g(\vec{x}_n) \tag{S4}$$

where J^{-1} is the psuedo-inverse of the Jacobian. We include step scaling parameter h to improve the convergence, which was heuristically set at $h = 0.1$. We iterate until the solution converges, but deem it unsuccessful if it does not converge within a set number of iterations (specifically, we used the convergence criterion $|\vec{x}_{n+1} - \vec{x}_n|_2 < 10^{-12}$ with a maximum of 10^3 iterations).

Typically, this process will converge to a nearby fixed point both more accurately and more quickly than direct numerical simulation of the system. However, it is itself insufficient for a number of reasons:

- Convergence is not guaranteed.
- As described, it can only find fixed points and not limit cycles.
- It converges without regard to the stability of a fixed point. We are most interested in stable solutions of the system.

We supplement this method with direct simulations as described below.

1.2.3 Simulation

If Newton's method does not converge, or if it does converge to a previously-discovered *unstable* fixed point within the system a simulation is performed. Specifically, we use the order-4 Runge-Kutta method to

integrate the system from a given initial condition. This simulation continues until one of the following conditions is met:

- The system converges.
- Periodicity is detected within the system
- The maximum number of timesteps is exceeded.

Note that the simulation's periodicity check is performed as follows: trajectory positions are recorded as reference points at random time intervals. After the trajectory leaves a small neighborhood around the reference point, its distance from the point is continuously monitored, and trajectories which return to the point's neighborhood are labeled as periodic (starting after the trajectory has left the neighborhood of the reference point, to exclude fixed point solutions). Clearly, the distance threshold for detection must be sufficiently small so as to exclude transient spirals towards fixed points. For any threshold, there could exist a sufficiently slow transient decay such that the trajectory was detected to be "periodic", but the perspective of this manuscript would consider very slow transients (as might re-enter a sufficiently small neighborhood of a reference point) to be functionally periodic, and so this method of checking periodicity suffices.

1.3 Algorithm for Diagram Generation

A summary of precisely how we combine Newton's Method with Simulations is shown in Figure S1(A). We first initialize a set of "guess" points $X_0 = [\vec{x_{01}}, \vec{x_{02}}, ...]$, with the goal of, ideally, finding stable solution(s) near each. Fixed points found at this input level are added to X_i. If this is the first set of calculations (i.e. the first input level), X_0 contains only the standard equilibrium point. If previous input levels have been calculated, the set of points found for the previous input level are used. The standard equilibrium for the new input level is added to this set if it is missing.

The members of X_0 generate the initial guesses for Newton's method; specifically, Newton's method is initialized from a point displaced along the least stable direction from the point in X_0 (i.e. the direction corresponding to the Jacobian eigenvalue with the maximum real component). In practice, this is seen to lead to a much faster convergence of Newton's method, and compared to a random perturbation is more successful in leading Newton's method to new fixed points (rather than just collapsing onto the already-found fixed point).

If Newton's method is not successful (either failing to converge within the maximum number of cycles, or converging to a point already in X_i) then a simulation is performed starting at a random point in the full-dimensional space. If Newton's method is successful but converges to a previously-found unstable point, a simulation is performed from the neighborhood of this unstable point. If it converges to a new, different unstable point, the algorithm records this new point and tries Newton's method again. If Newton's method is successful at finding a stable fixed point, it is recorded in X_i and the loop ends.

Simulations have ongoing convergence and periodicity checks, along with a maximum number of timesteps. If the maximum number of timesteps is exceeded without finding a stable solution, then Newton's method is tried again from last simulation point (which may be, ideally, within the basin of attraction of a new stable solution). If a stable fixed point or limit cycle is detected in a simulation, it is recorded in X_i, and the loop ends.

1.4 Comments on Generating Higher-Resolution Figures

Note that the algorithm, by design, continuously searches for new stable solutions which are not necessarily close to old branches of the diagram. These exploratory processes are unnecessary if the

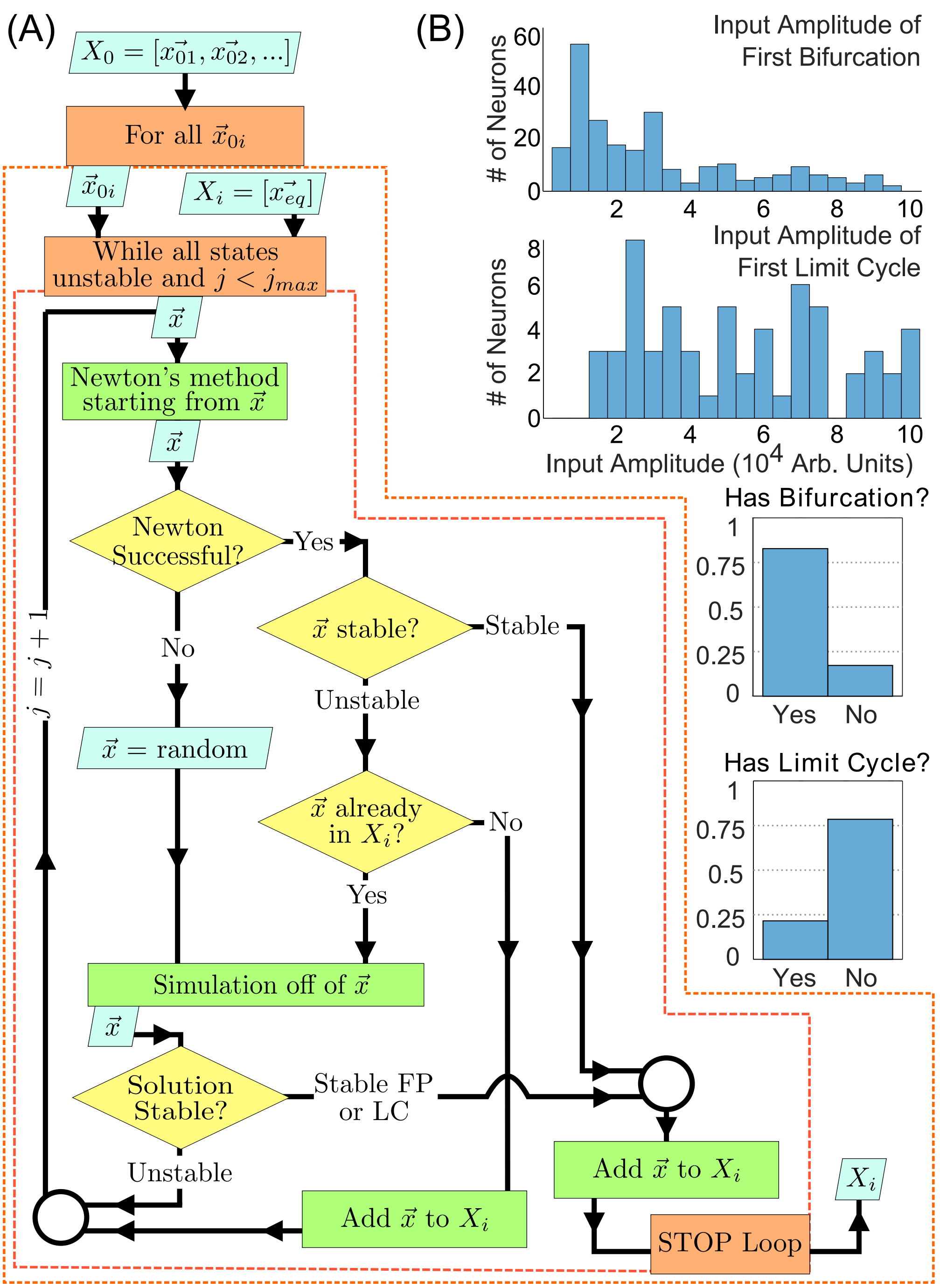

Figure S1. Panel (A) outlines the algorithm used at each input level of each diagram to automatically generate bifurcation diagrams such as the ones in Figures 2 and 3 and the Supplementary Materials. We discuss this algorithm in Section 1.3. We used this to generate 279 bifurcation diagrams for single-neuron inputs, all of which are included as Supplementary Materials. In Panel (B) we summarize some features from the diagrams generated for inputs into each of the 279 individual neurons.

number and type of fixed points within an input region are already known. The higher resolution bifurcation diagrams, such as those within Figures 2 and 3 of the main text, were therefore generated by initially generating a low-resolution diagram, then performing simulations to fill in each branch. For example, the fact that there appears to be only one stable solution for all inputs of Figure 2 means that the diagram can be generated simply by running a single simulation at each input point. Similarly, for Figure 3, one can use

the basin of attraction as in Figure 4 to choose two initial points, in the first and third quadrant, which are likely to converge to the fixed point and limit cycle solution respectively. Thus increasing the resolution of these branches was seen to typically require only two simulations per input level.

1.5 Bifurcation Diagrams for all Single-Neuron Inputs

Bifurcation diagrams were generated for the set of all possible single-neuron inputs into the system, and are included within the Supplementary Materials. A summary of a few properties of this set of diagrams is given in Figure S1(B). In particular, we show the fraction of single-neuron inputs which result in any bifurcation (i.e. for which the standard equilibrium becomes unstable) and the fraction of those for which we detect a limit cycle. The input amplitudes at which this first occurs is also shown.
