# Supplementary figures and images for: Multistability and Long-Timescale Transients Encoded by Network Structure in a Model of C. elegans Connectome Dynamics (part 2 of 2)
Source: Front Comput Neurosci. 2017 Jun 13;11:53. doi: 10.3389/fncom.2017.00053 (PMC5468412; doi:10.3389/fncom.2017.00053)

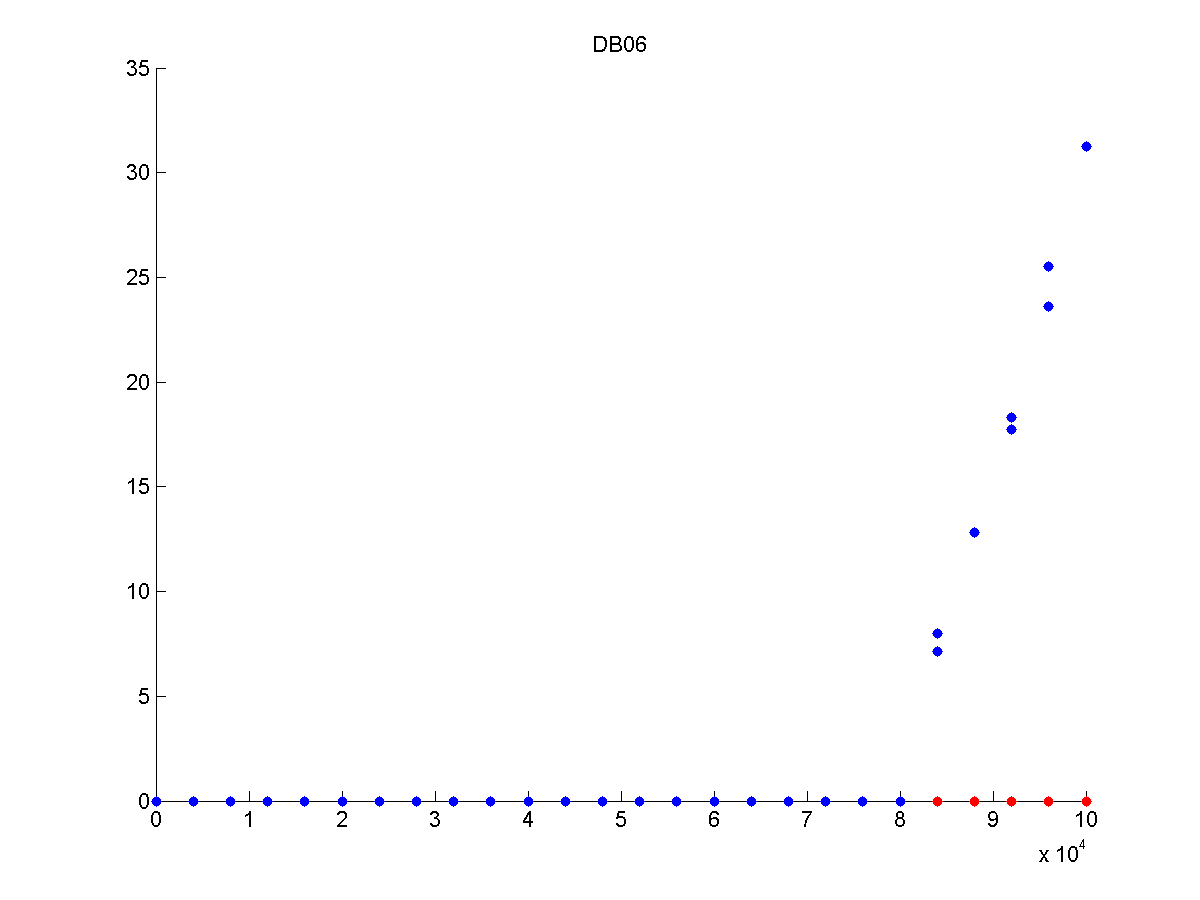

Supplement: Supplementary file 2 [file Presentation2.ZIP › DB06.png]

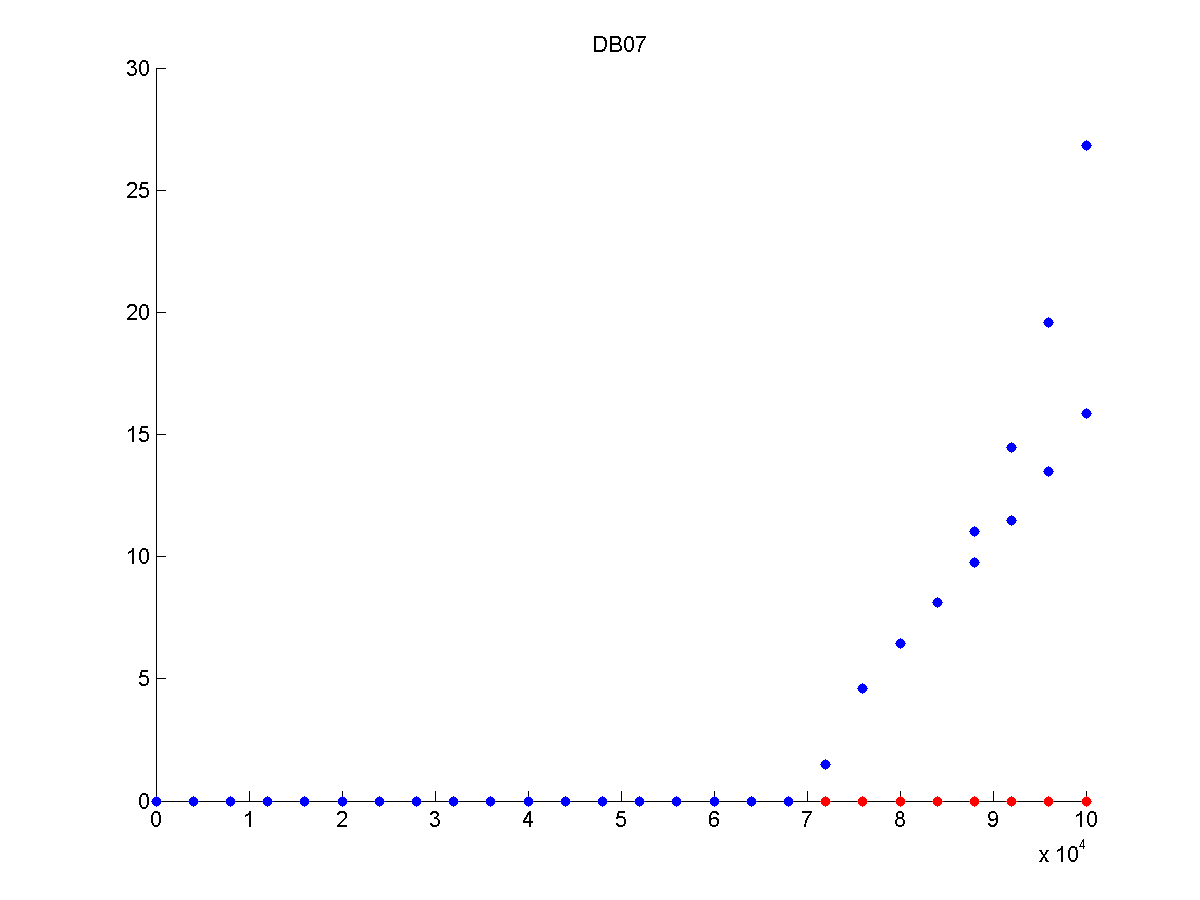

Supplement: Supplementary file 2 [file Presentation2.ZIP › DB07.png]

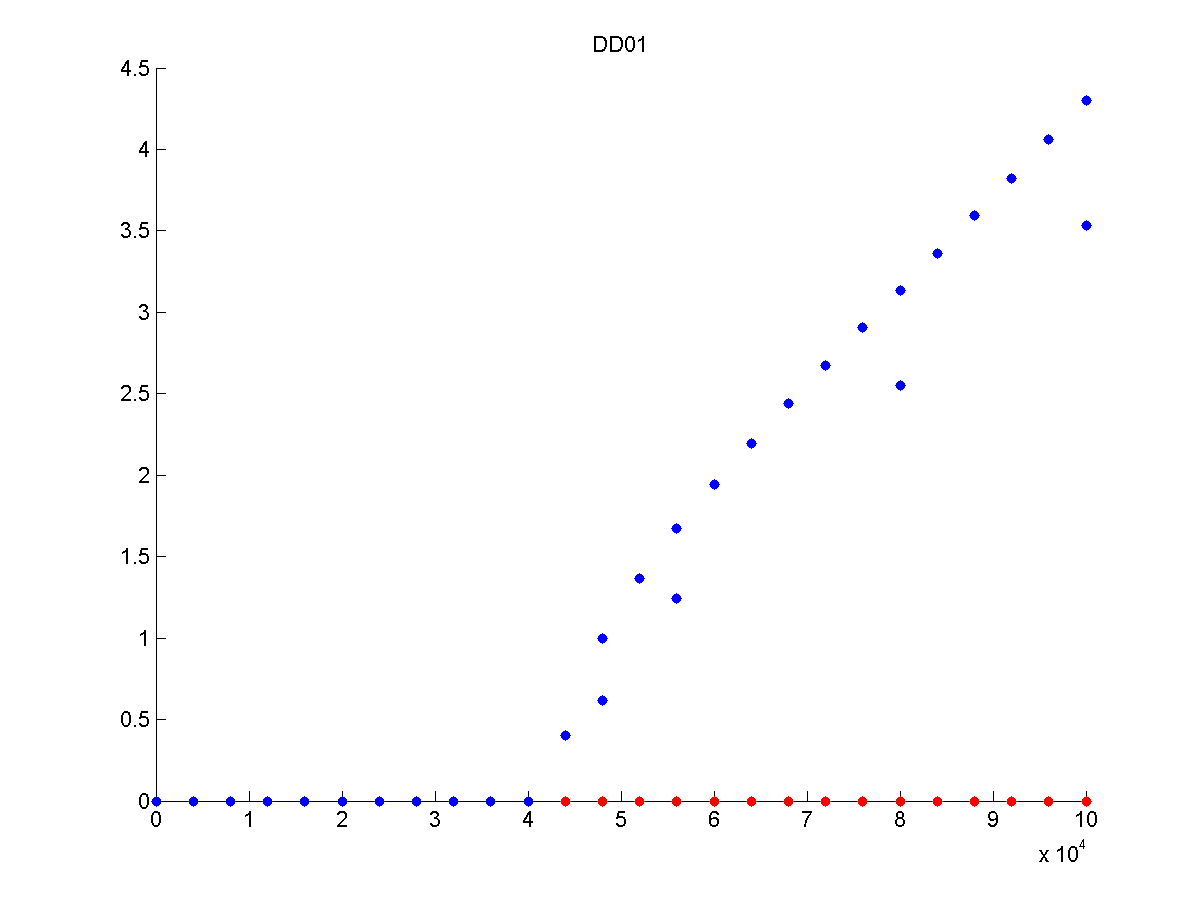

Supplement: Supplementary file 2 [file Presentation2.ZIP › DD01.png]

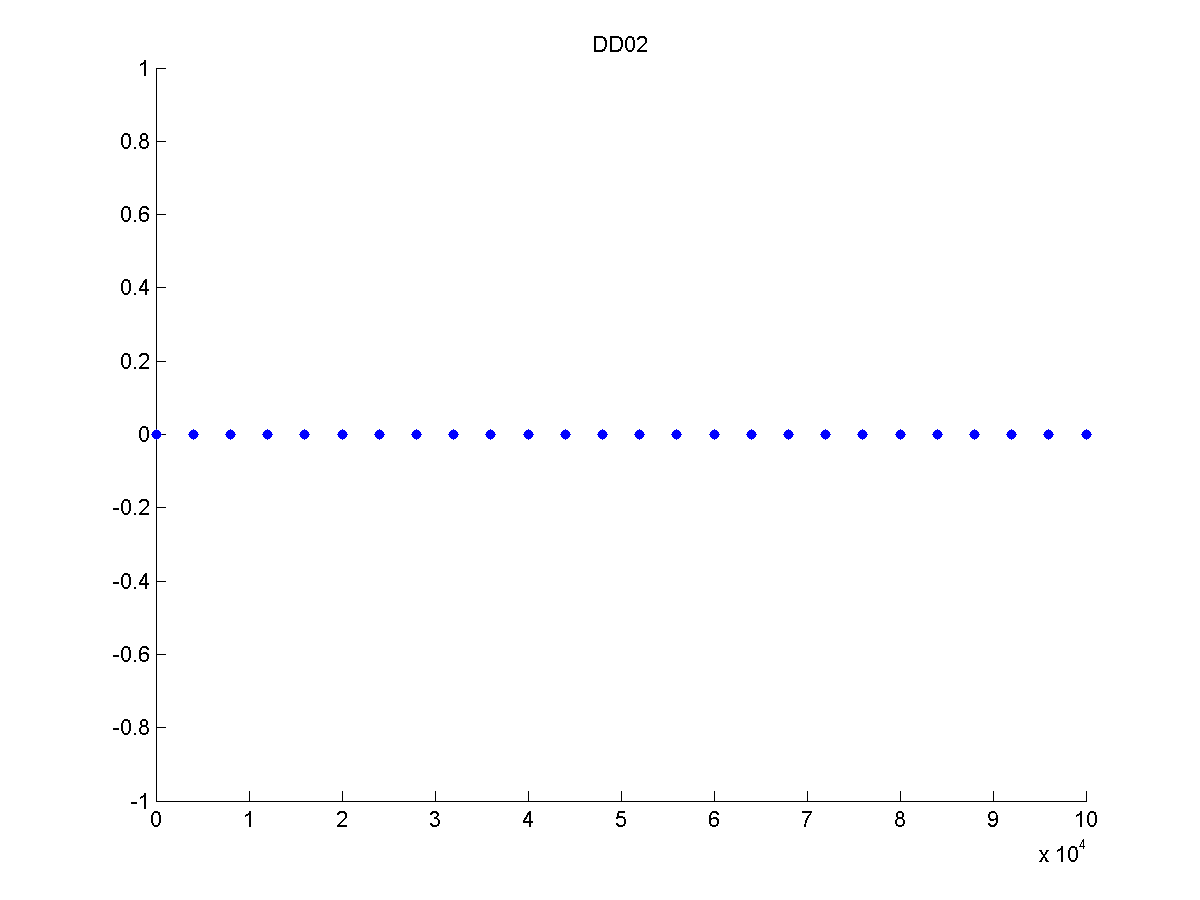

Supplement: Supplementary file 2 [file Presentation2.ZIP › DD02.png]

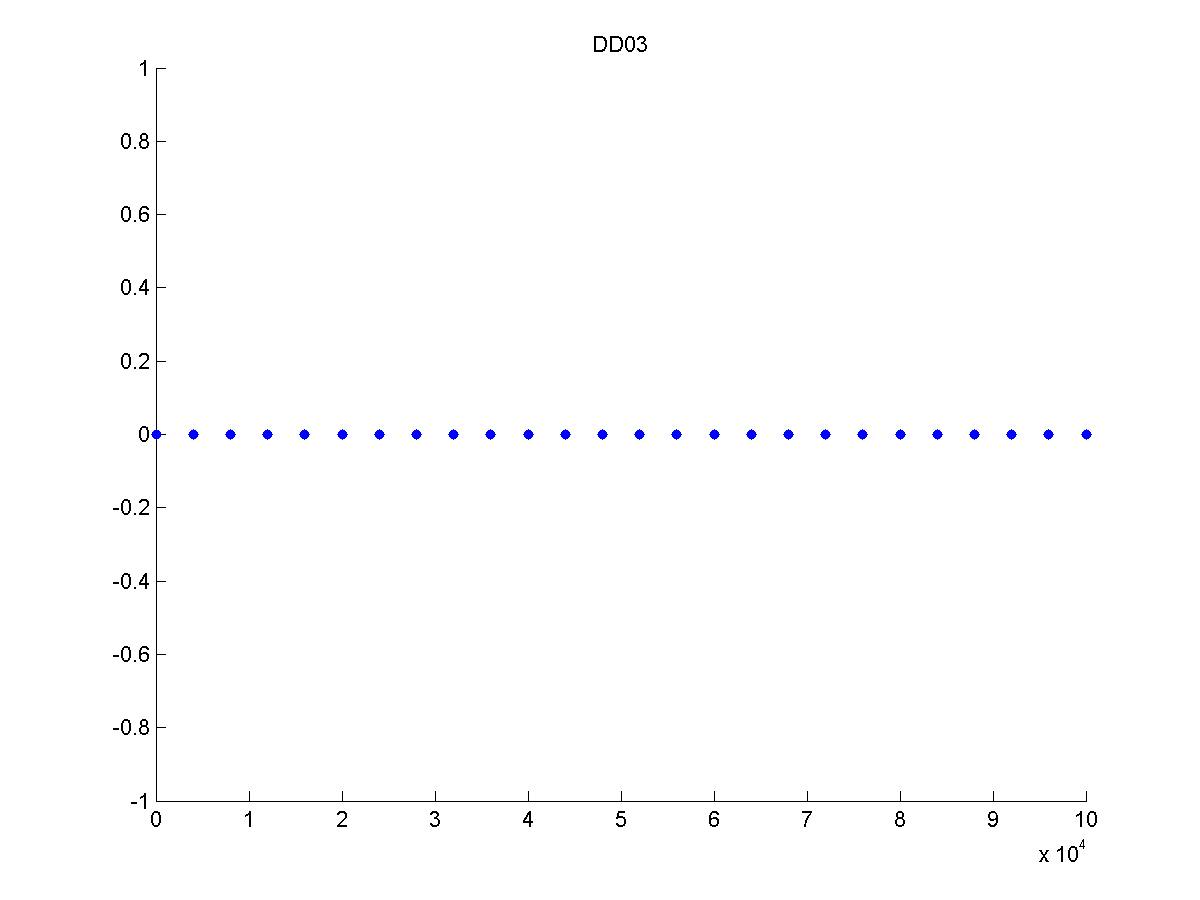

Supplement: Supplementary file 2 [file Presentation2.ZIP › DD03.png]

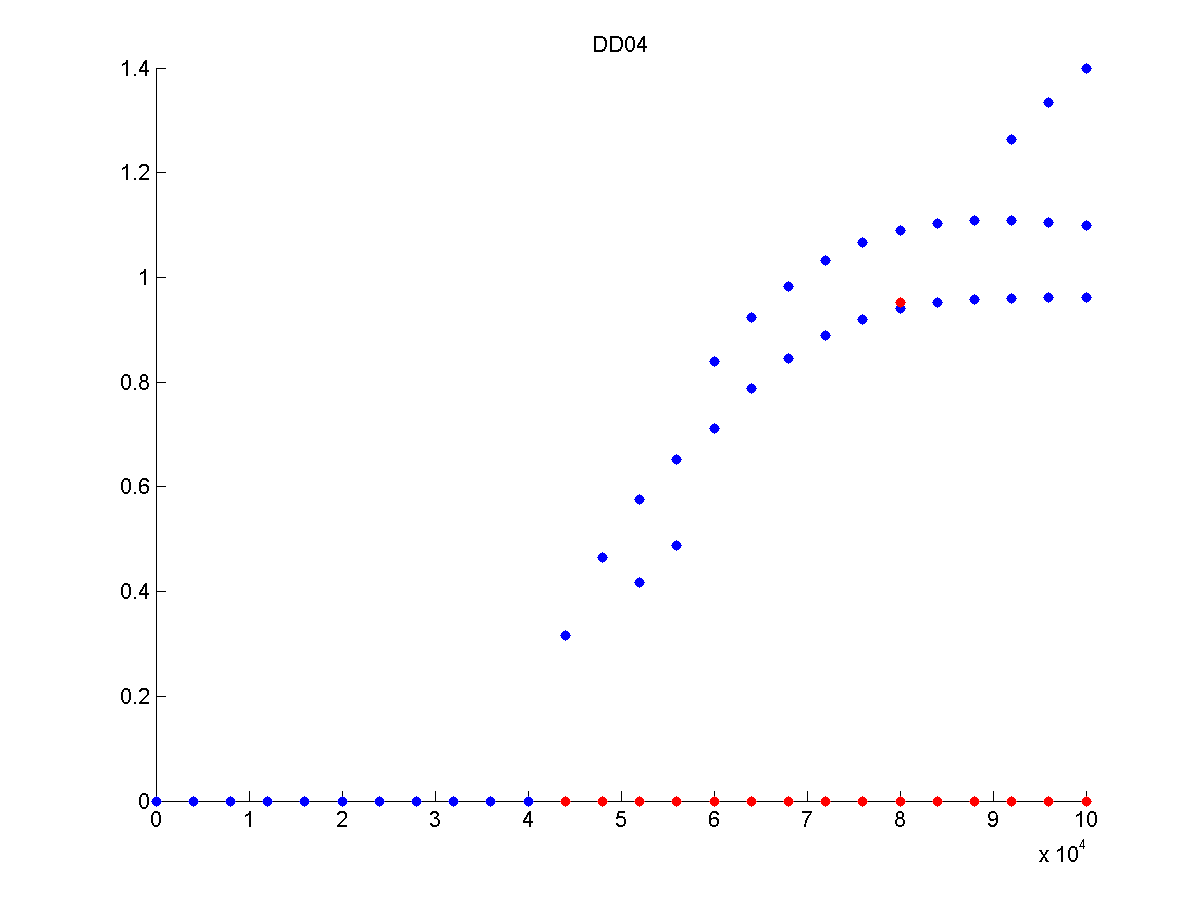

Supplement: Supplementary file 2 [file Presentation2.ZIP › DD04.png]

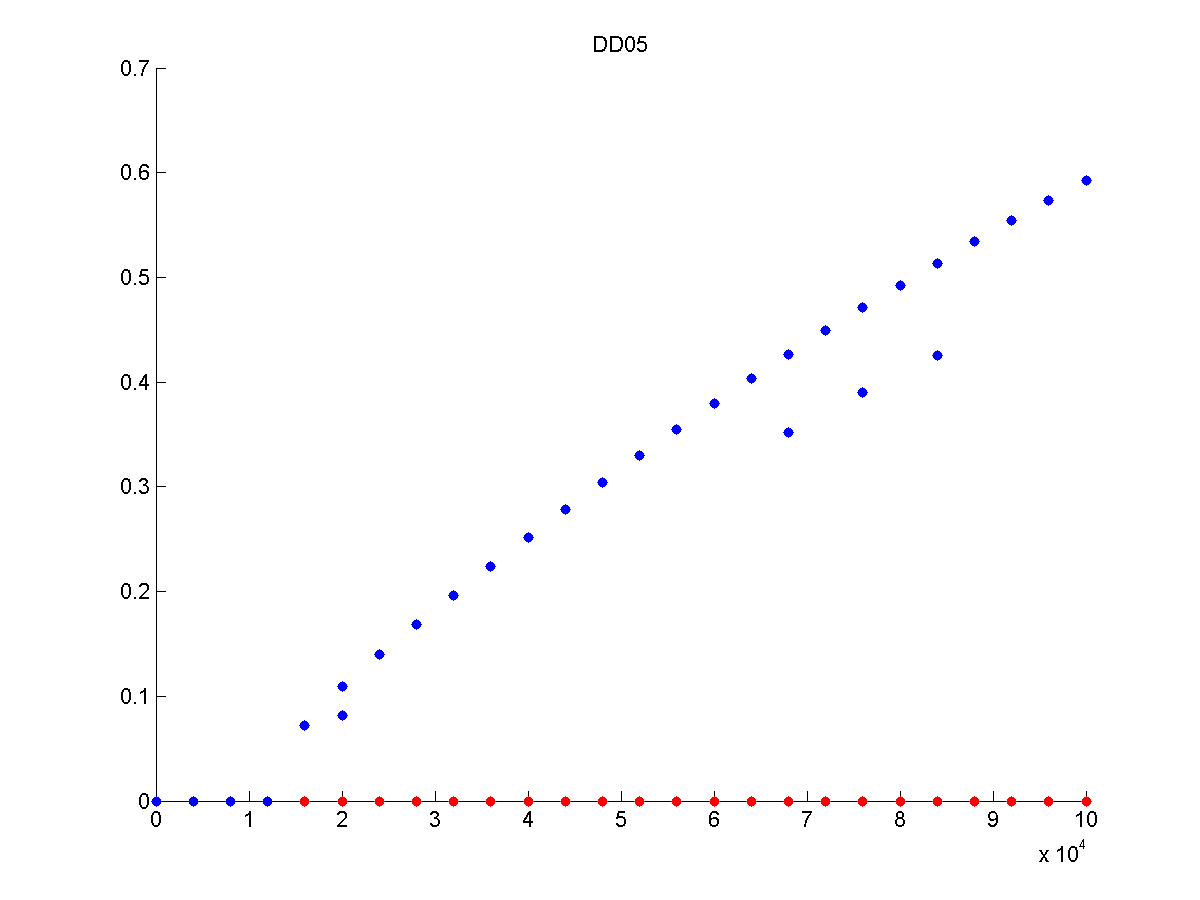

Supplement: Supplementary file 2 [file Presentation2.ZIP › DD05.png]

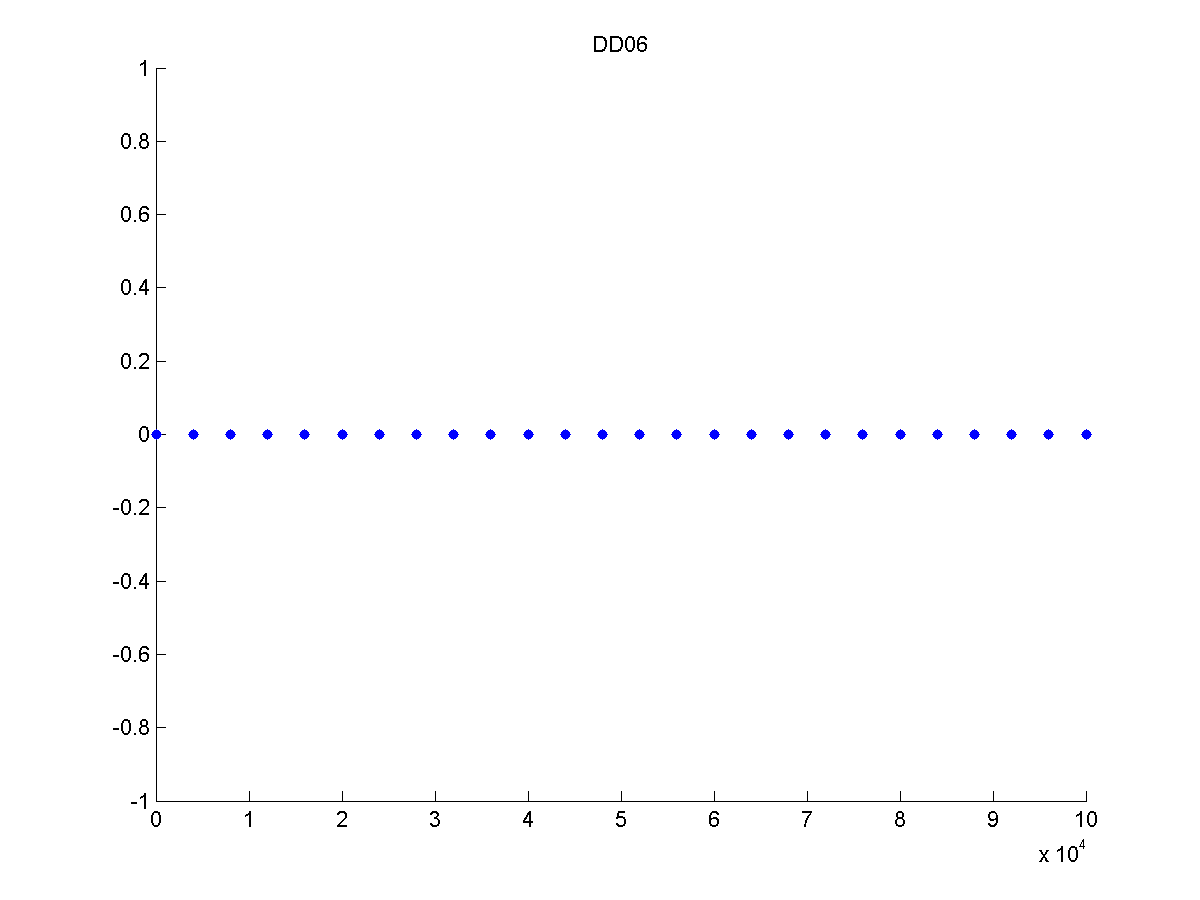

Supplement: Supplementary file 2 [file Presentation2.ZIP › DD06.png]

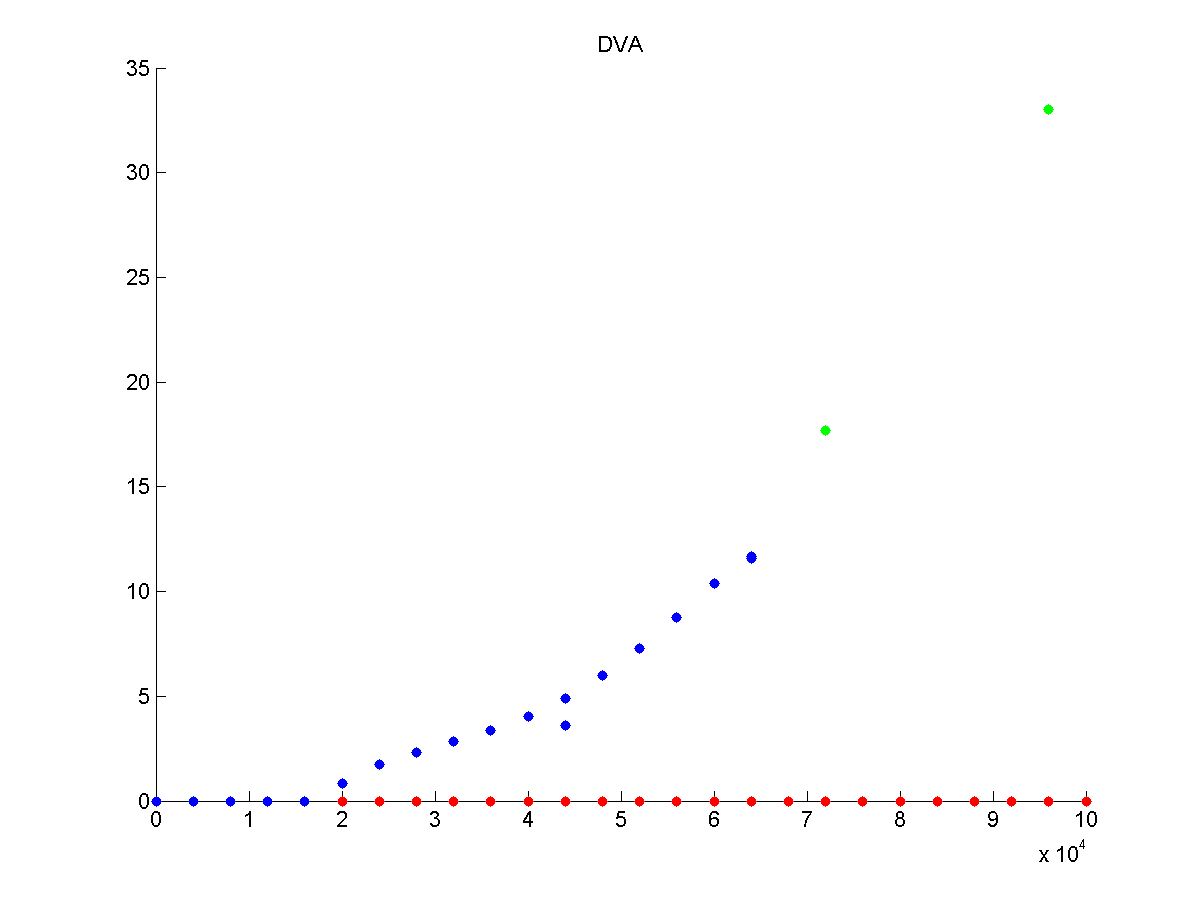

Supplement: Supplementary file 2 [file Presentation2.ZIP › DVA.png]

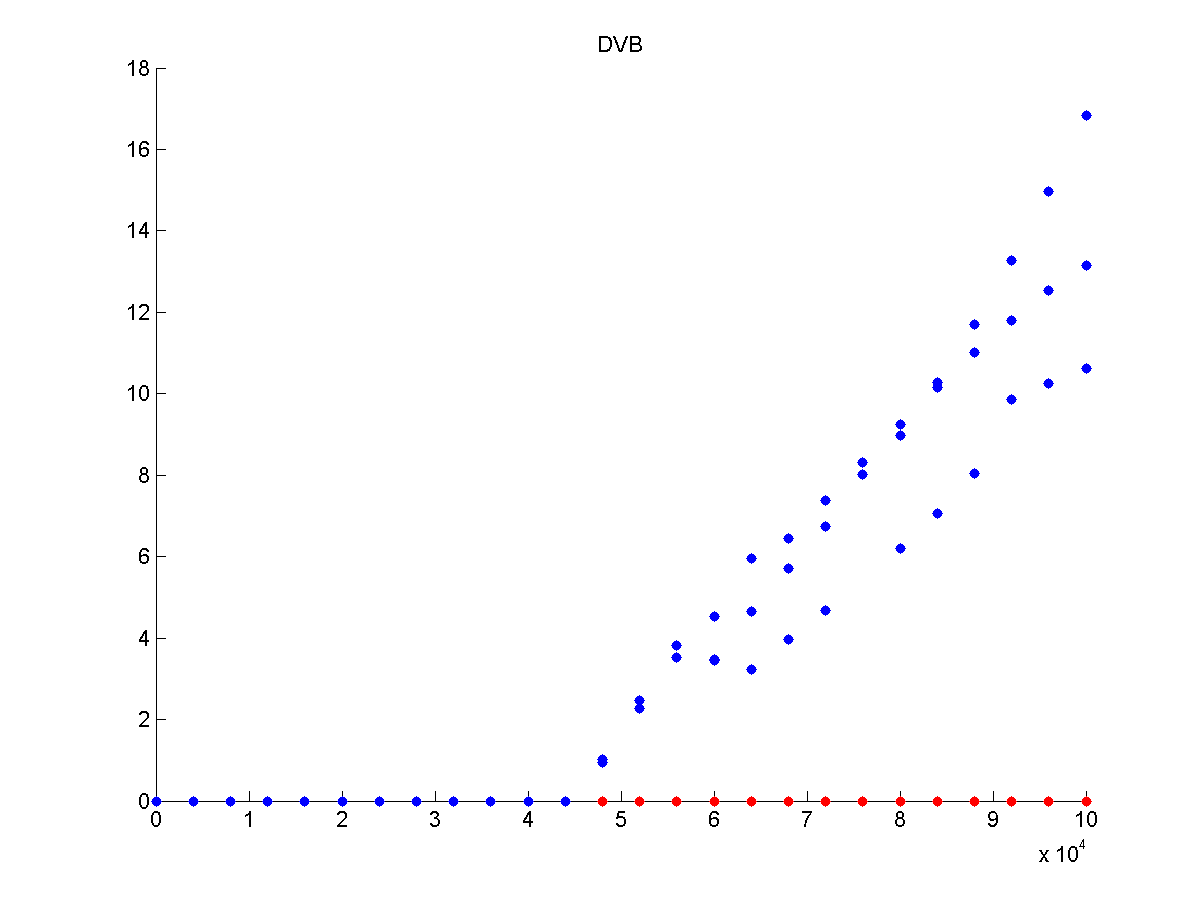

Supplement: Supplementary file 2 [file Presentation2.ZIP › DVB.png]

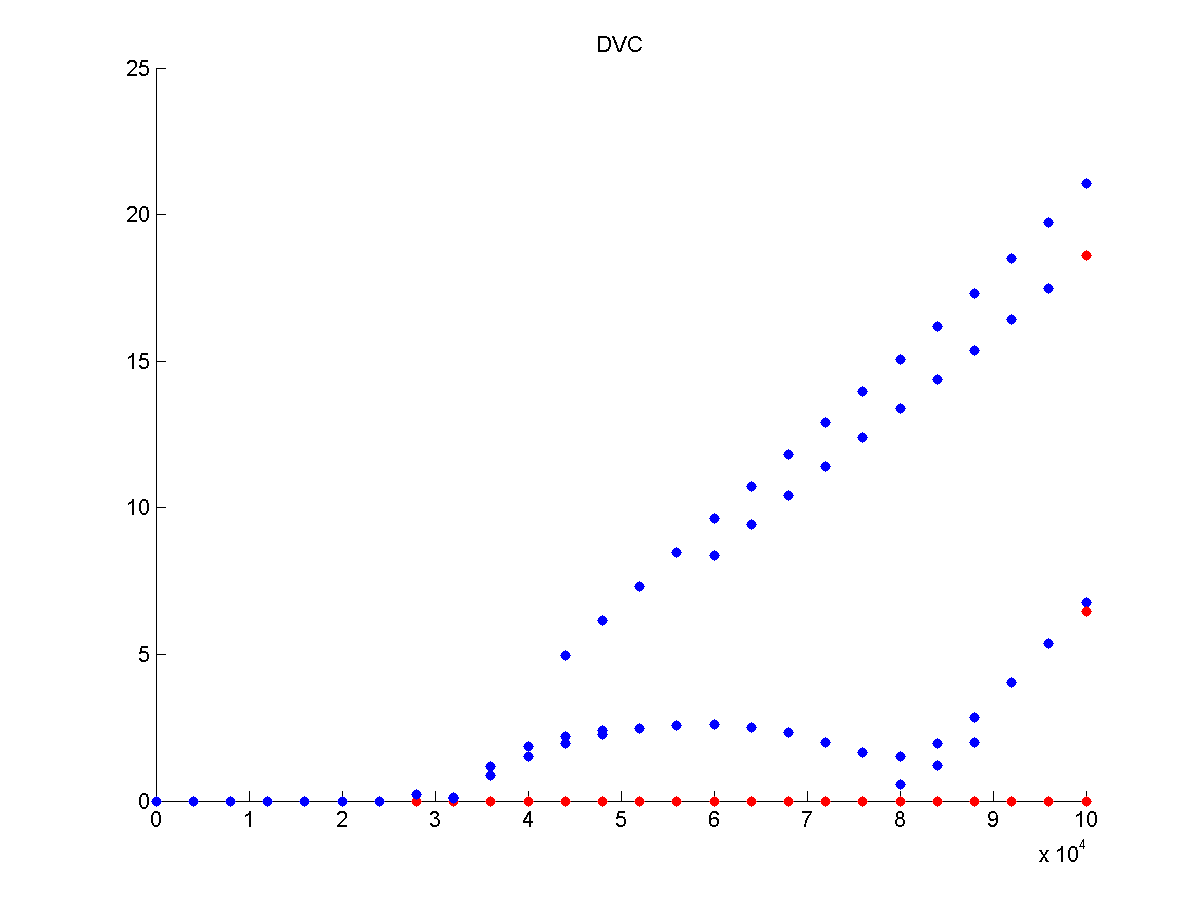

Supplement: Supplementary file 2 [file Presentation2.ZIP › DVC.png]

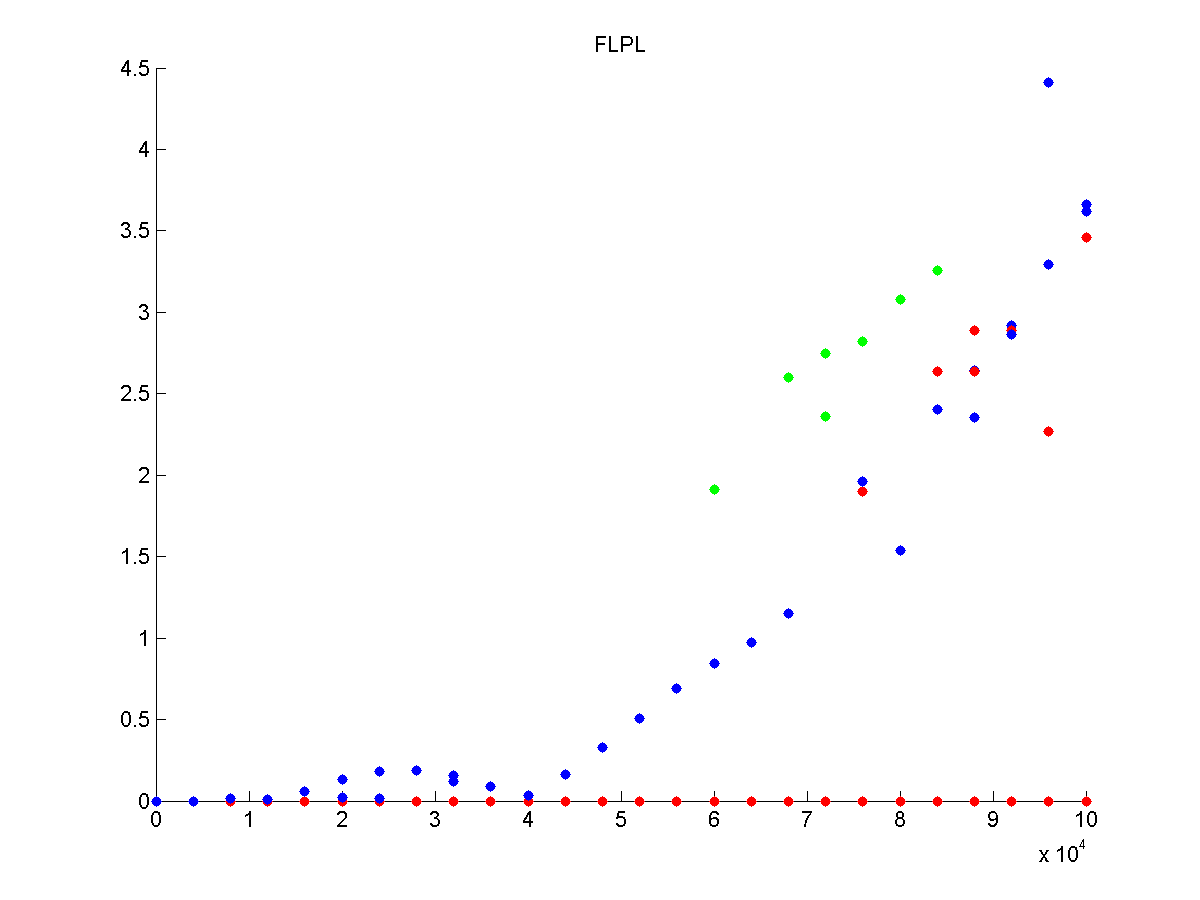

Supplement: Supplementary file 2 [file Presentation2.ZIP › FLPL.png]

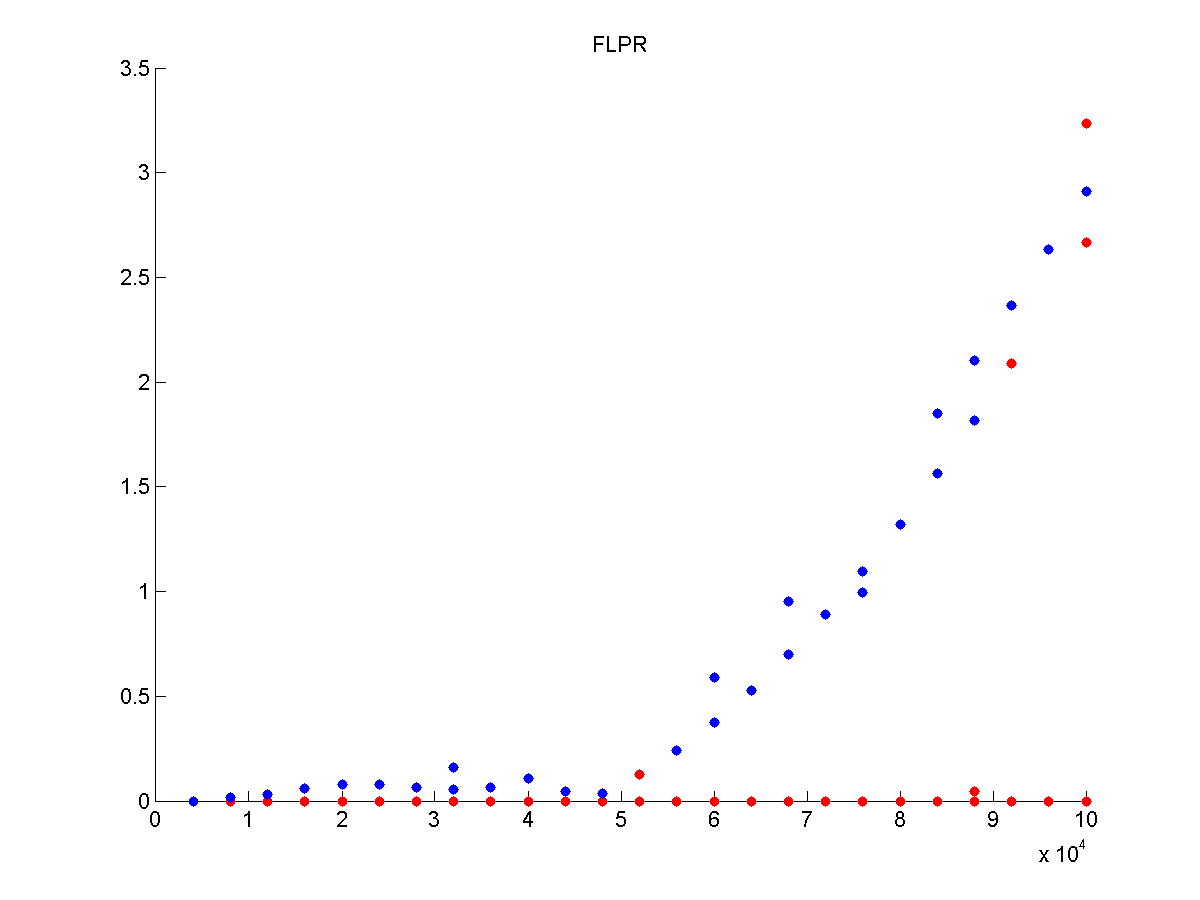

Supplement: Supplementary file 2 [file Presentation2.ZIP › FLPR.png]

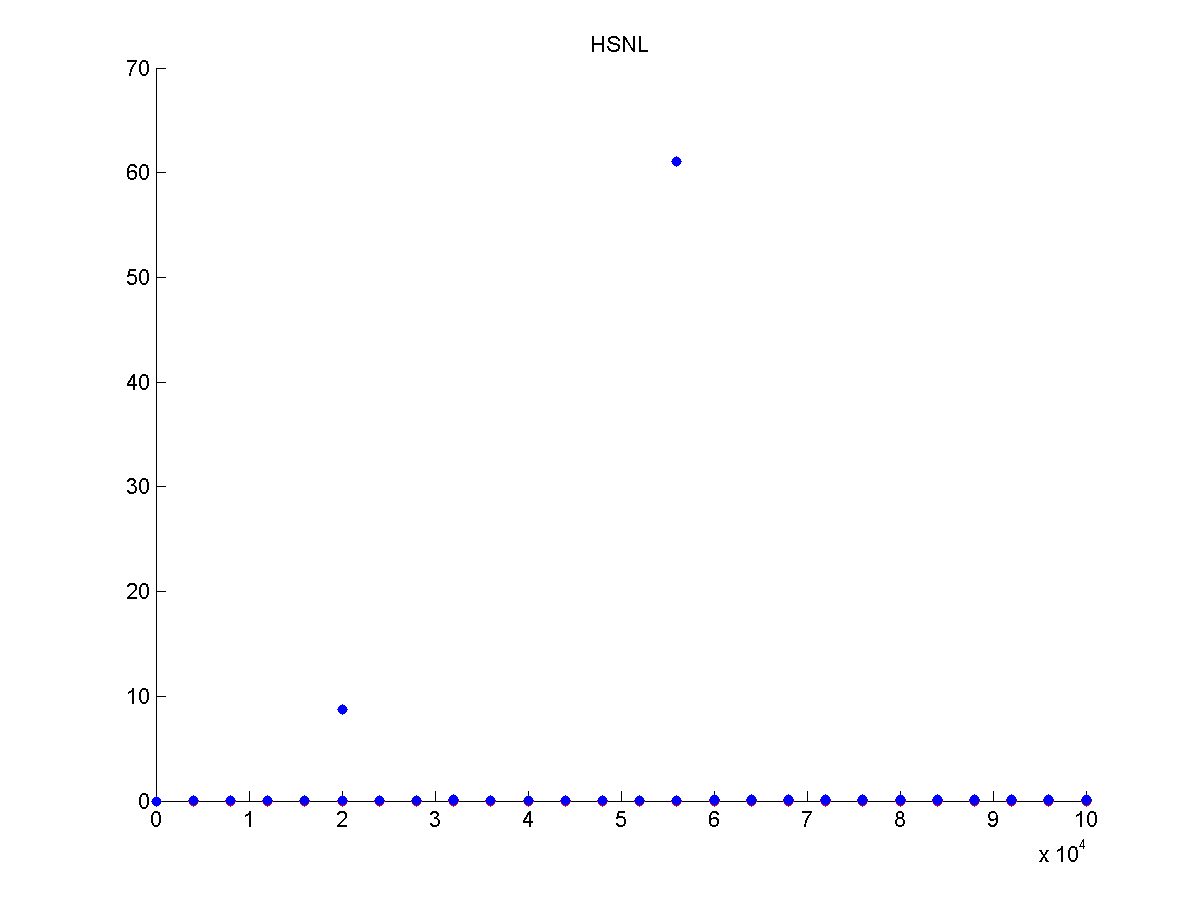

Supplement: Supplementary file 2 [file Presentation2.ZIP › HSNL.png]

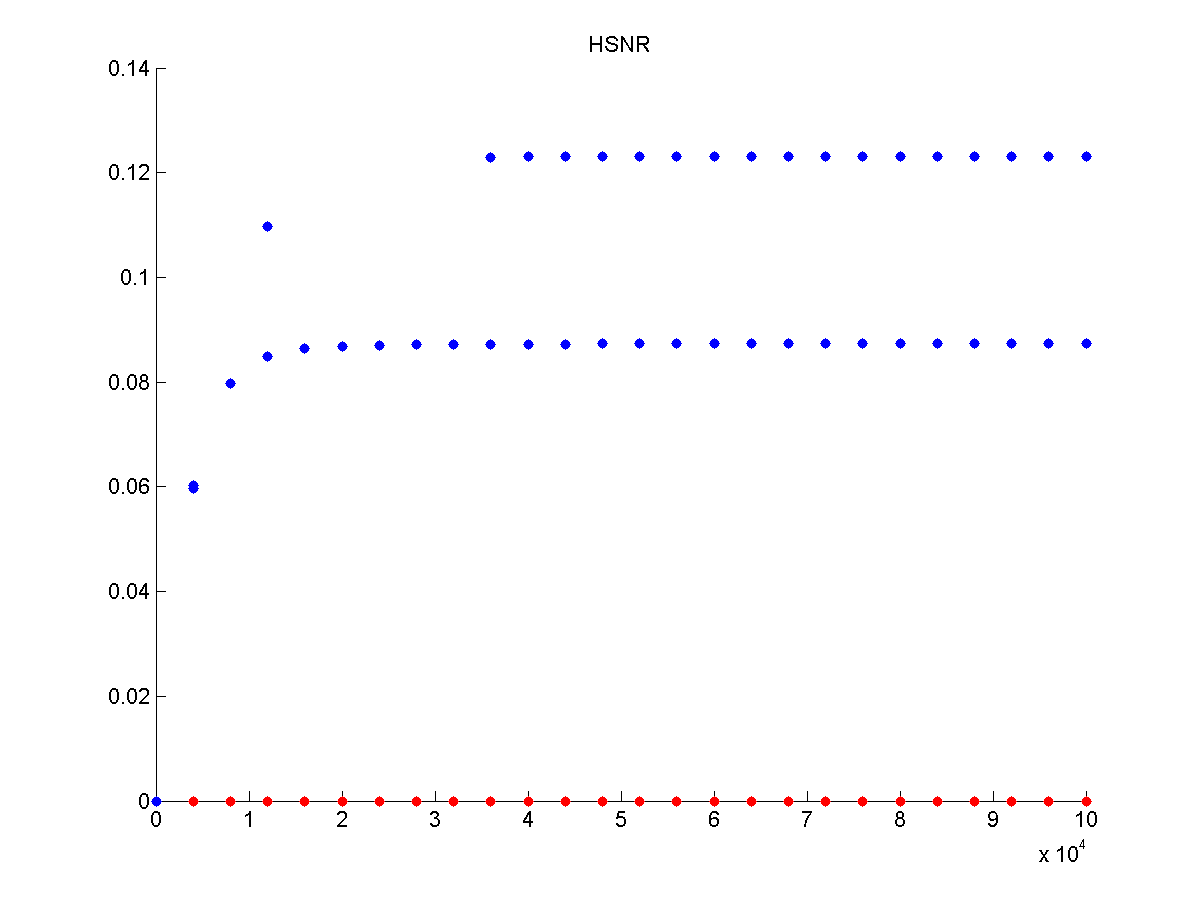

Supplement: Supplementary file 2 [file Presentation2.ZIP › HSNR.png]

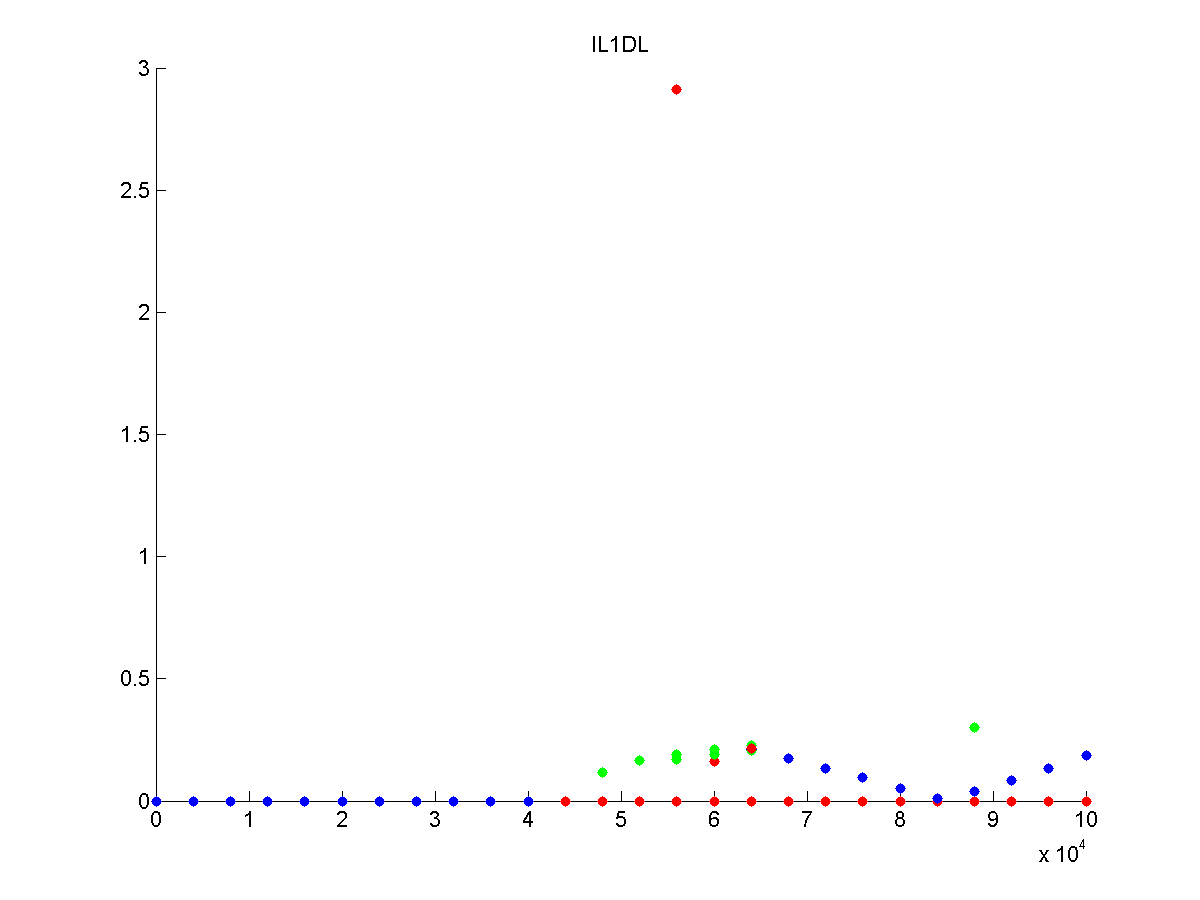

Supplement: Supplementary file 2 [file Presentation2.ZIP › IL1DL.png]

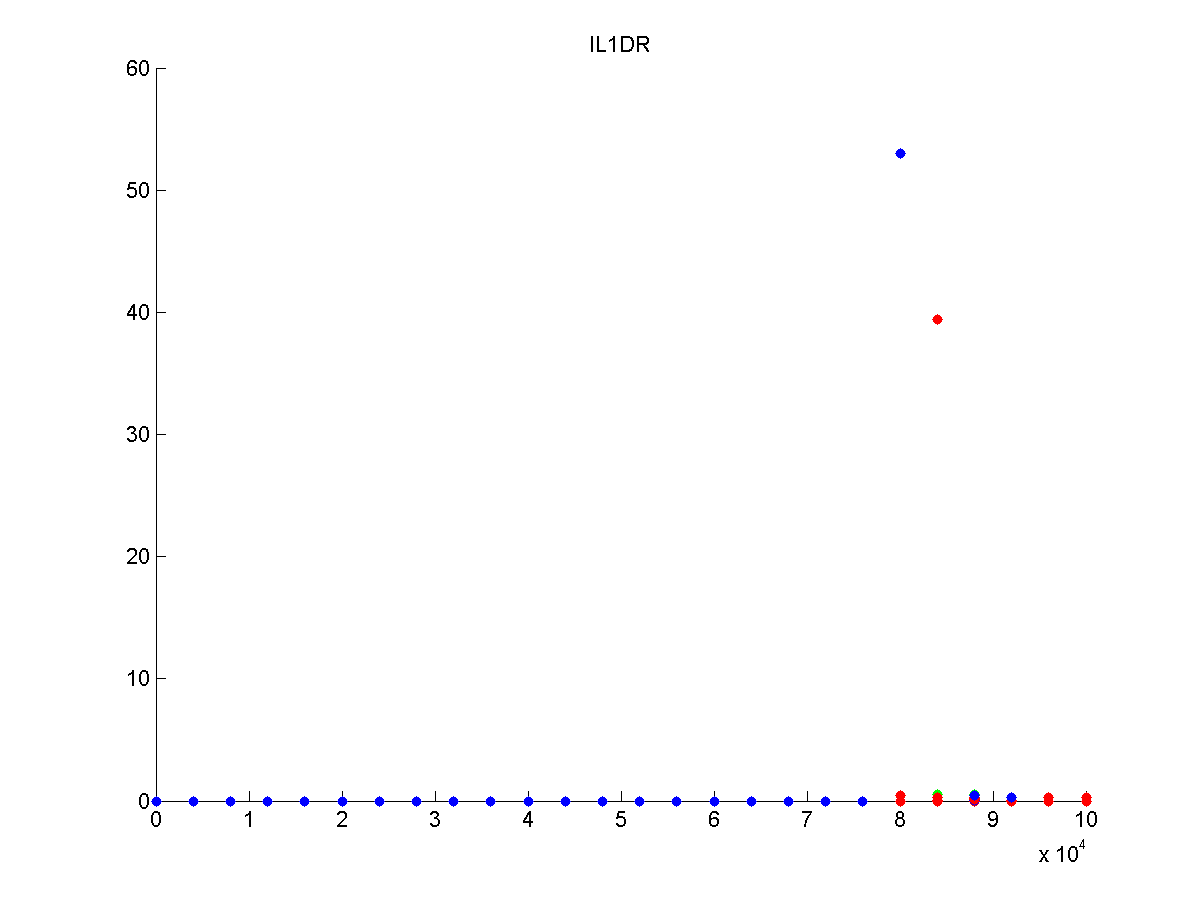

Supplement: Supplementary file 2 [file Presentation2.ZIP › IL1DR.png]

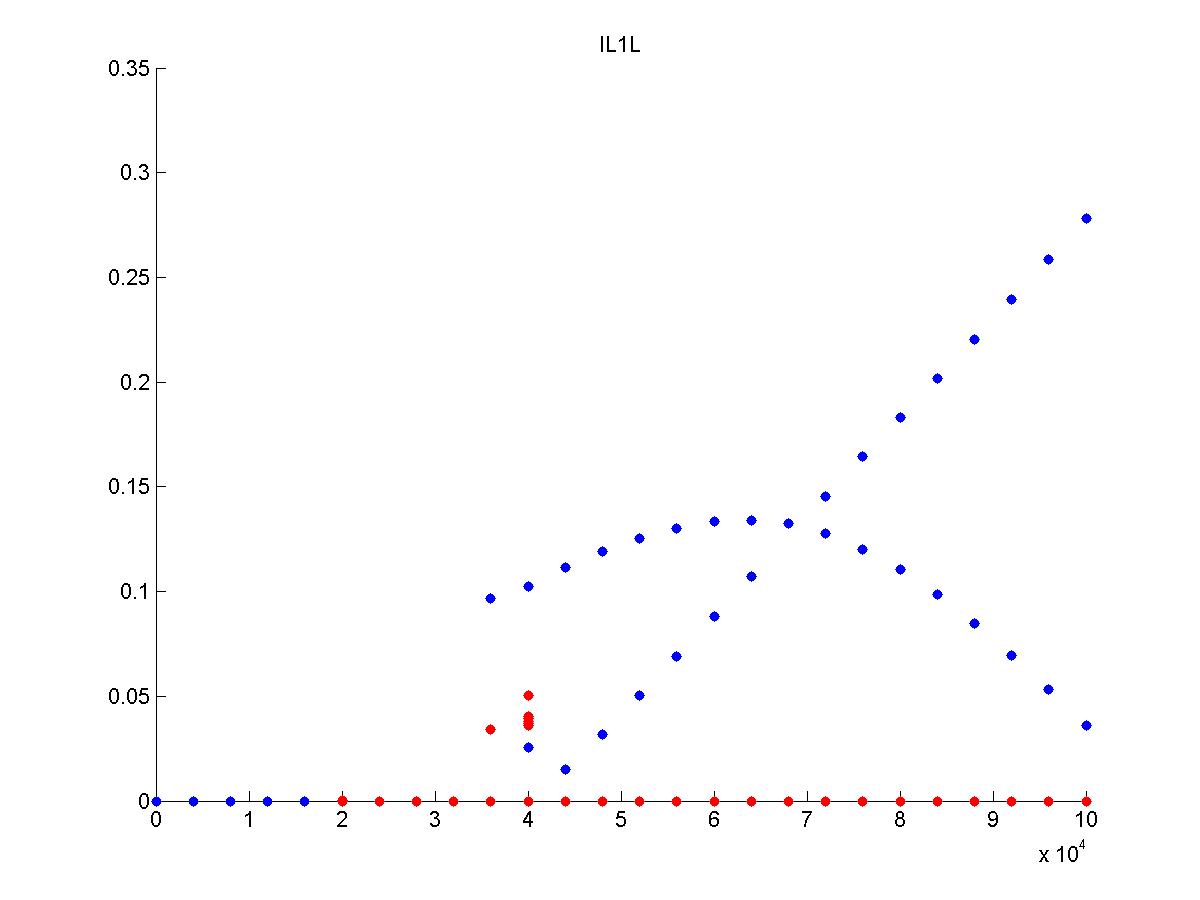

Supplement: Supplementary file 2 [file Presentation2.ZIP › IL1L.png]

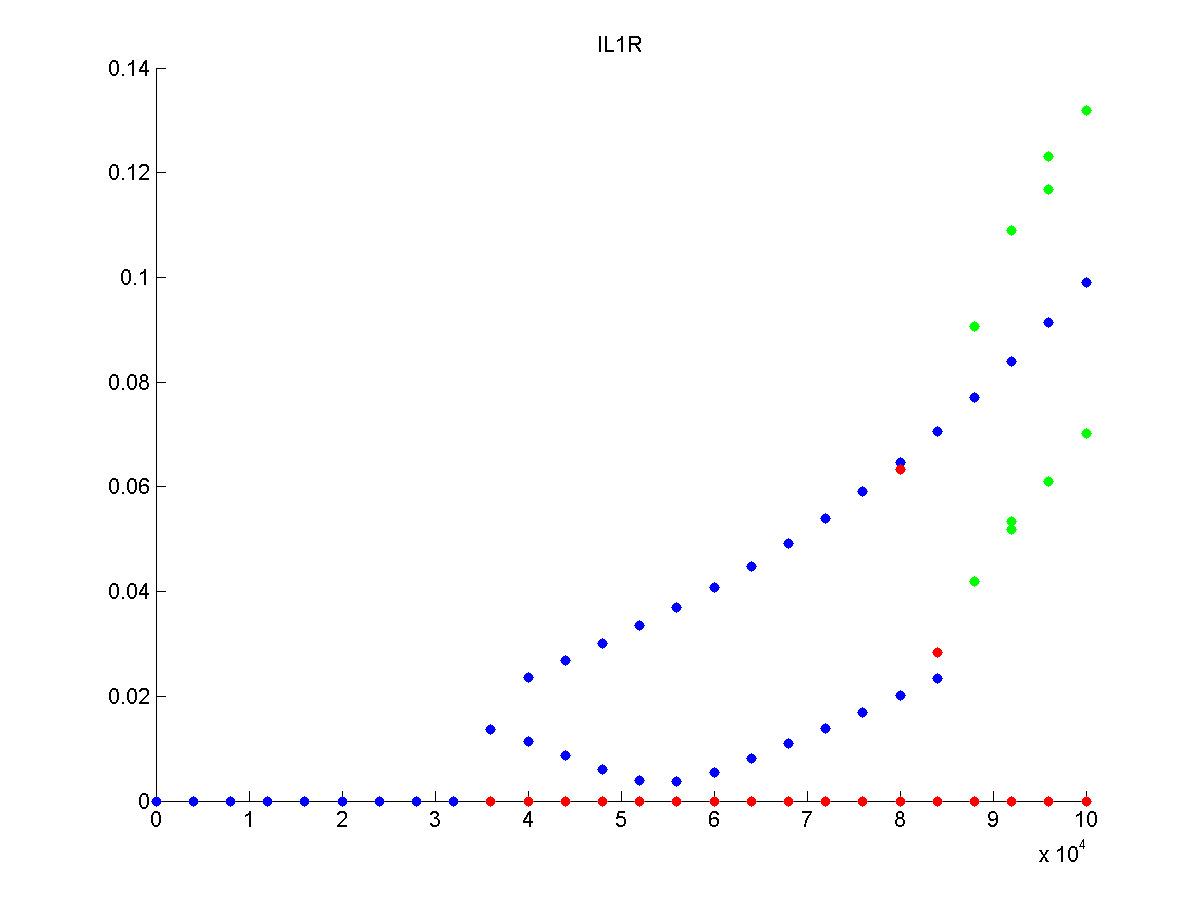

Supplement: Supplementary file 2 [file Presentation2.ZIP › IL1R.png]

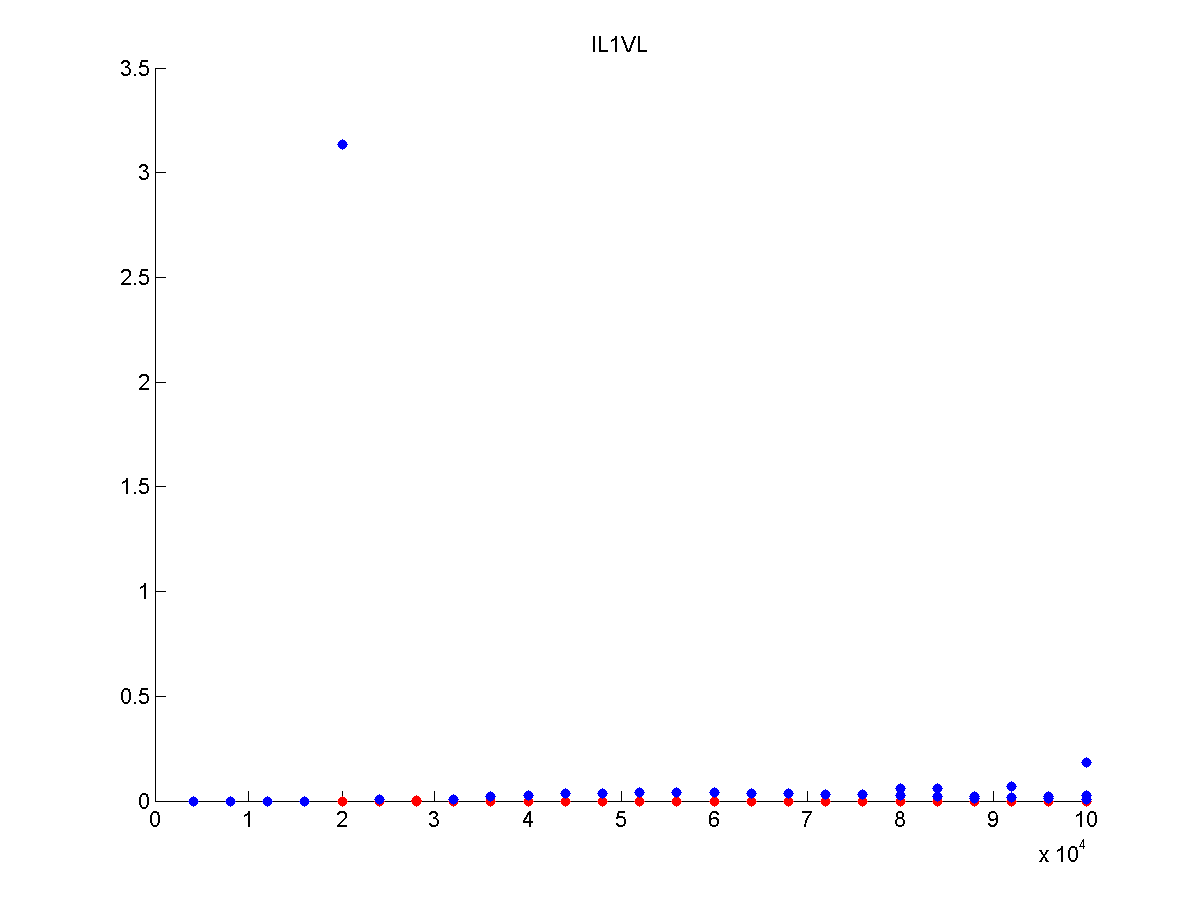

Supplement: Supplementary file 2 [file Presentation2.ZIP › IL1VL.png]

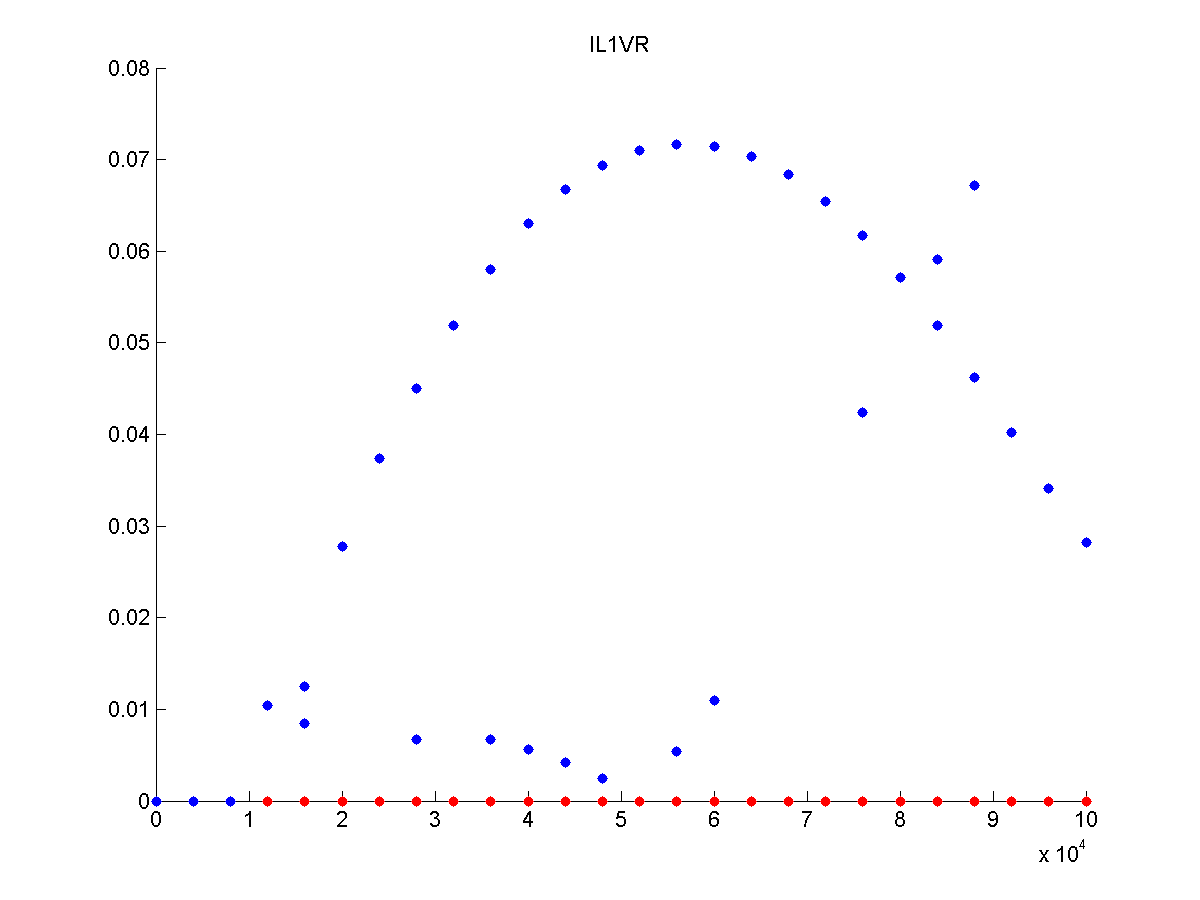

Supplement: Supplementary file 2 [file Presentation2.ZIP › IL1VR.png]

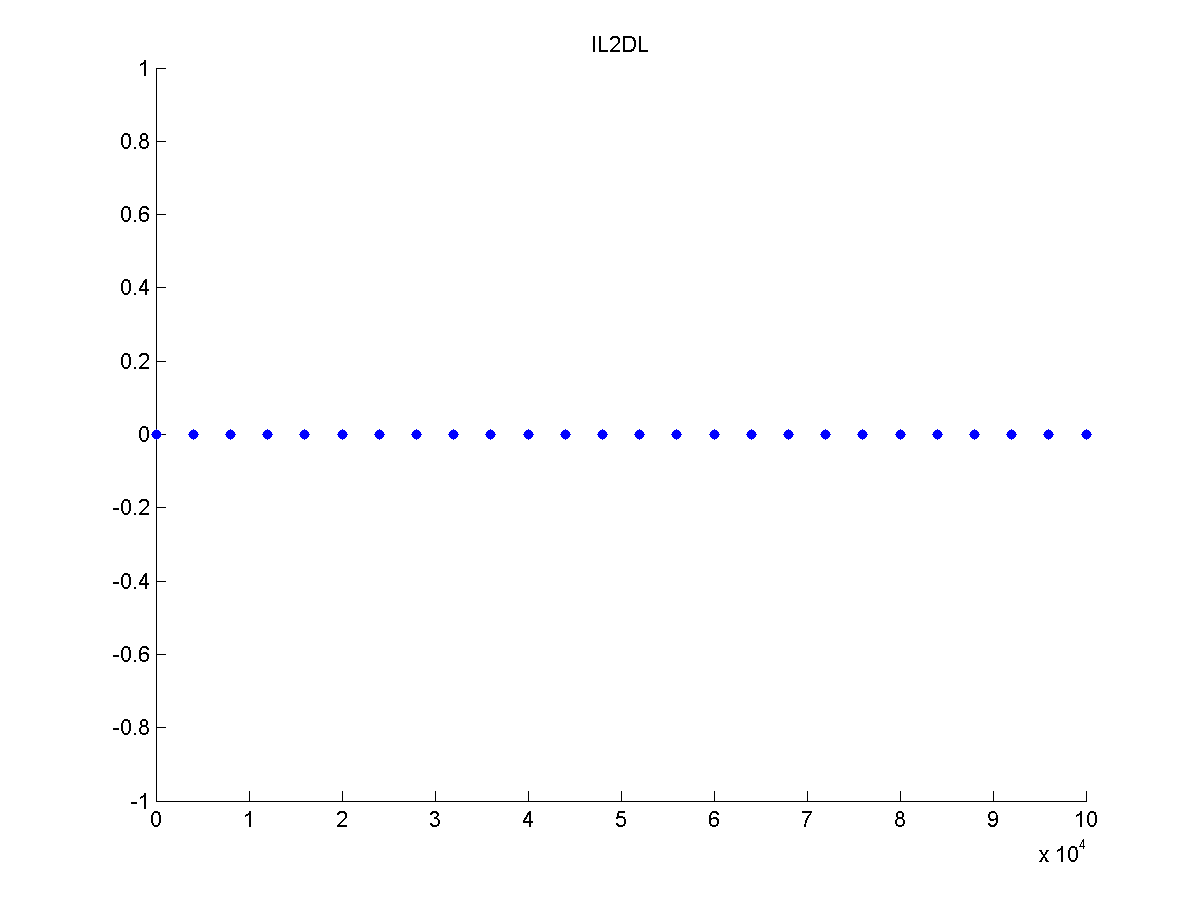

Supplement: Supplementary file 2 [file Presentation2.ZIP › IL2DL.png]

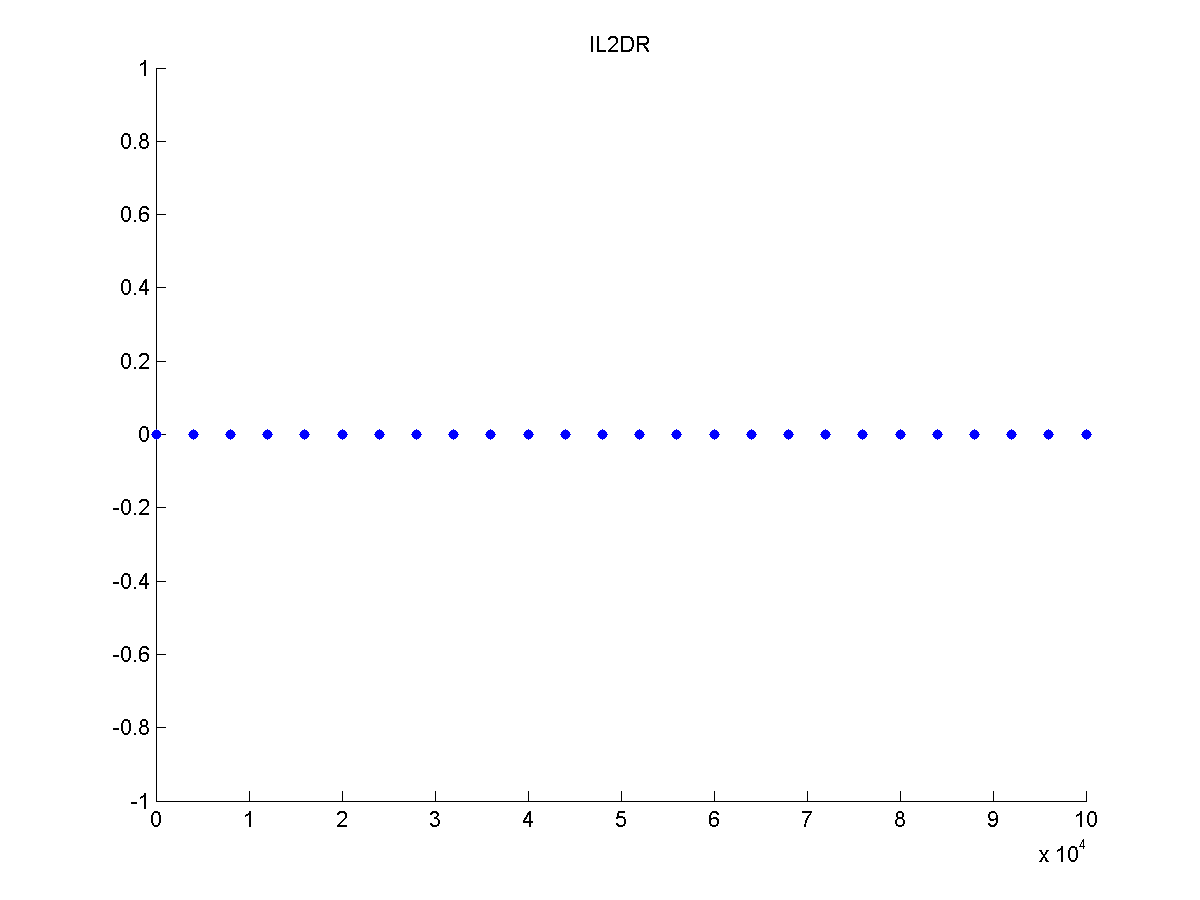

Supplement: Supplementary file 2 [file Presentation2.ZIP › IL2DR.png]

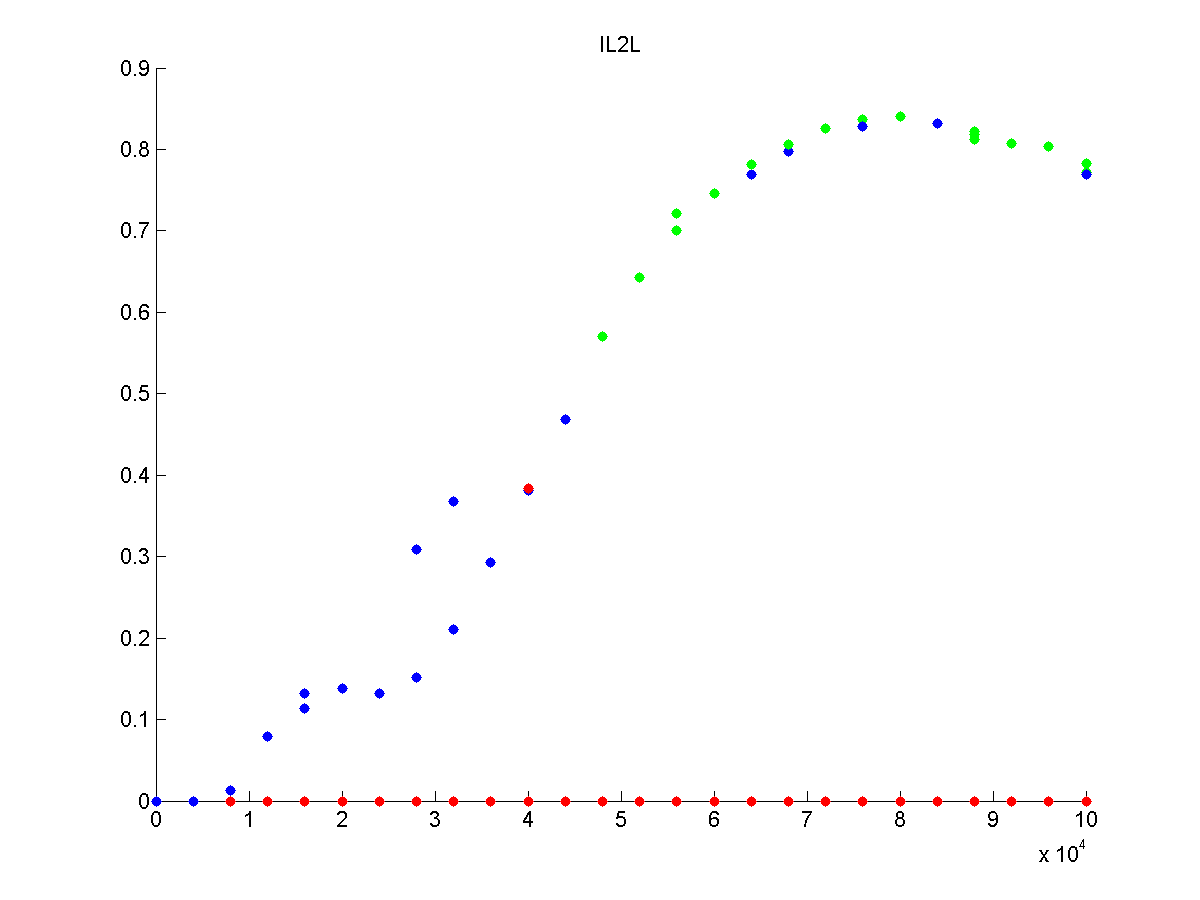

Supplement: Supplementary file 2 [file Presentation2.ZIP › IL2L.png]

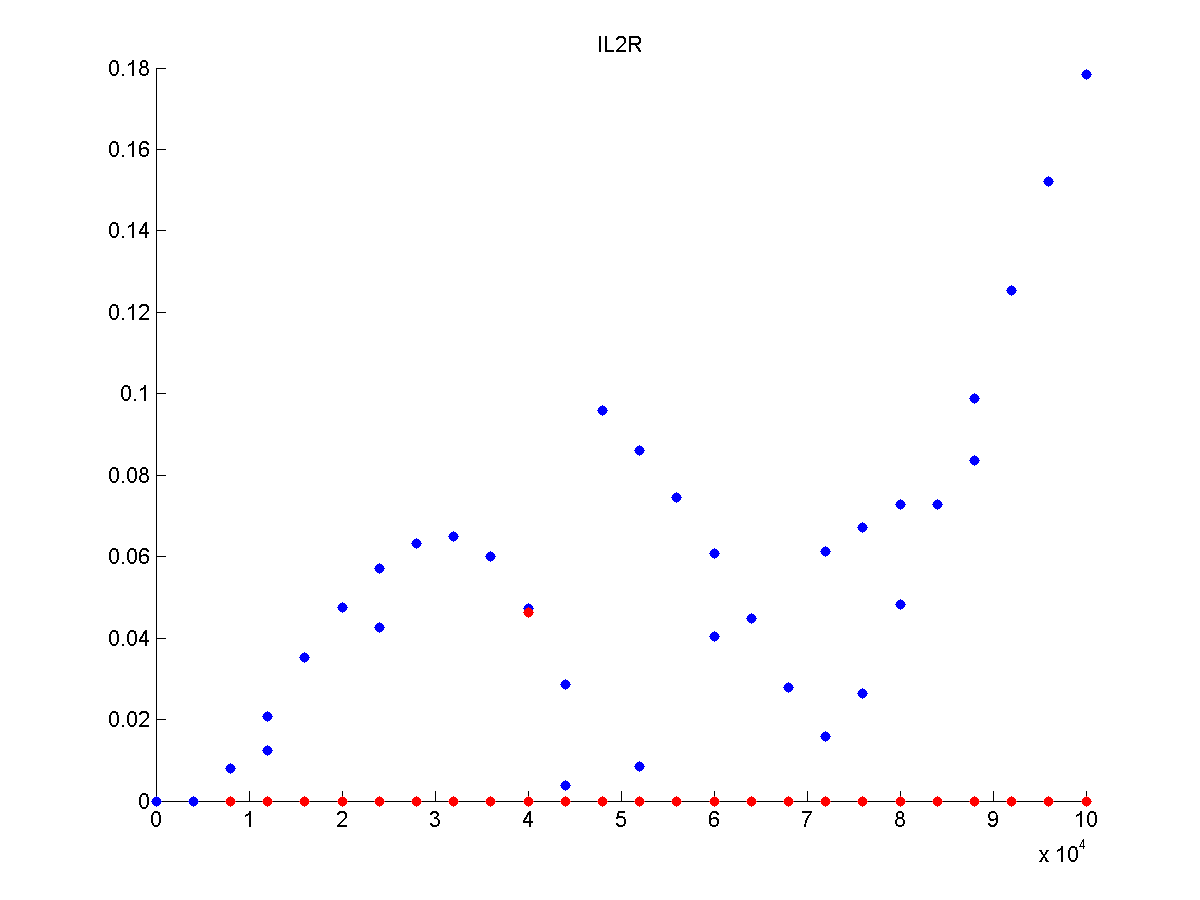

Supplement: Supplementary file 2 [file Presentation2.ZIP › IL2R.png]

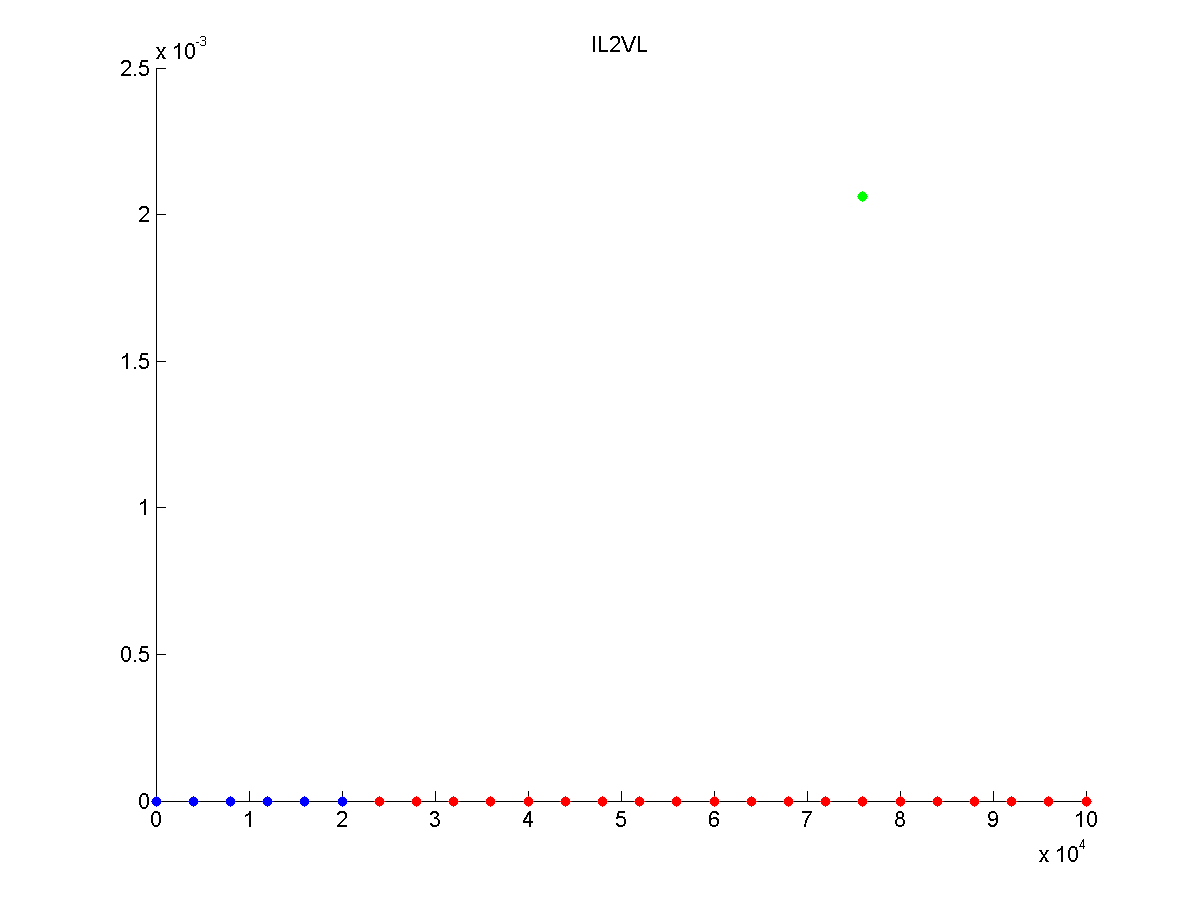

Supplement: Supplementary file 2 [file Presentation2.ZIP › IL2VL.png]

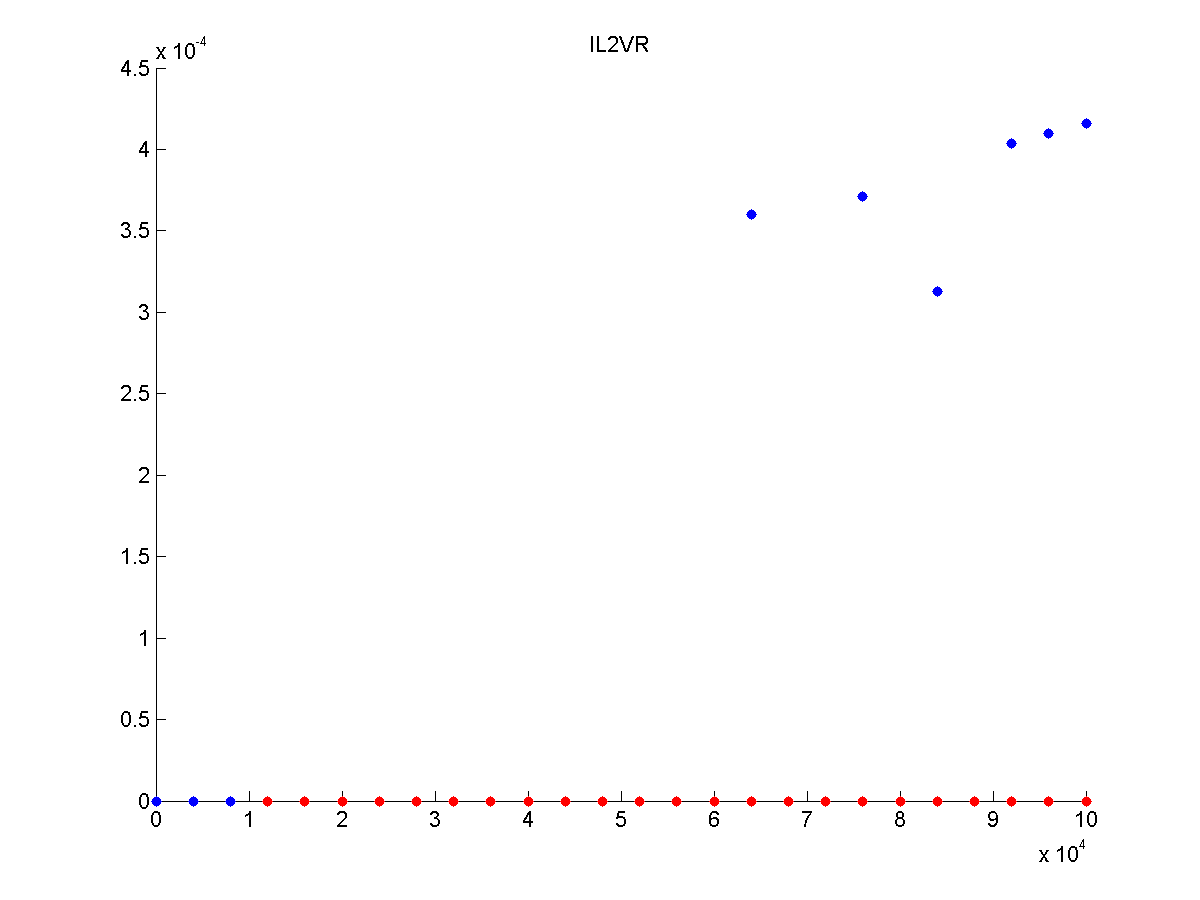

Supplement: Supplementary file 2 [file Presentation2.ZIP › IL2VR.png]

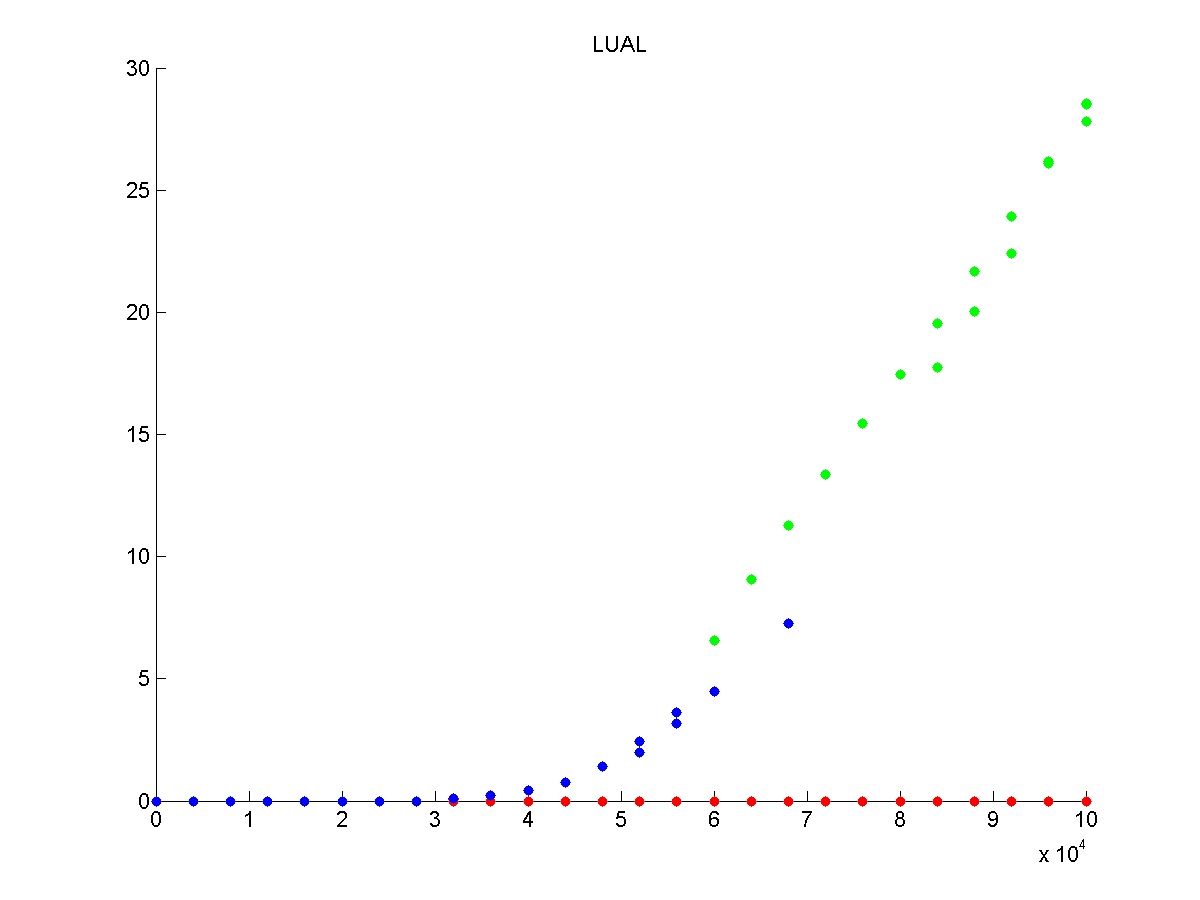

Supplement: Supplementary file 2 [file Presentation2.ZIP › LUAL.png]

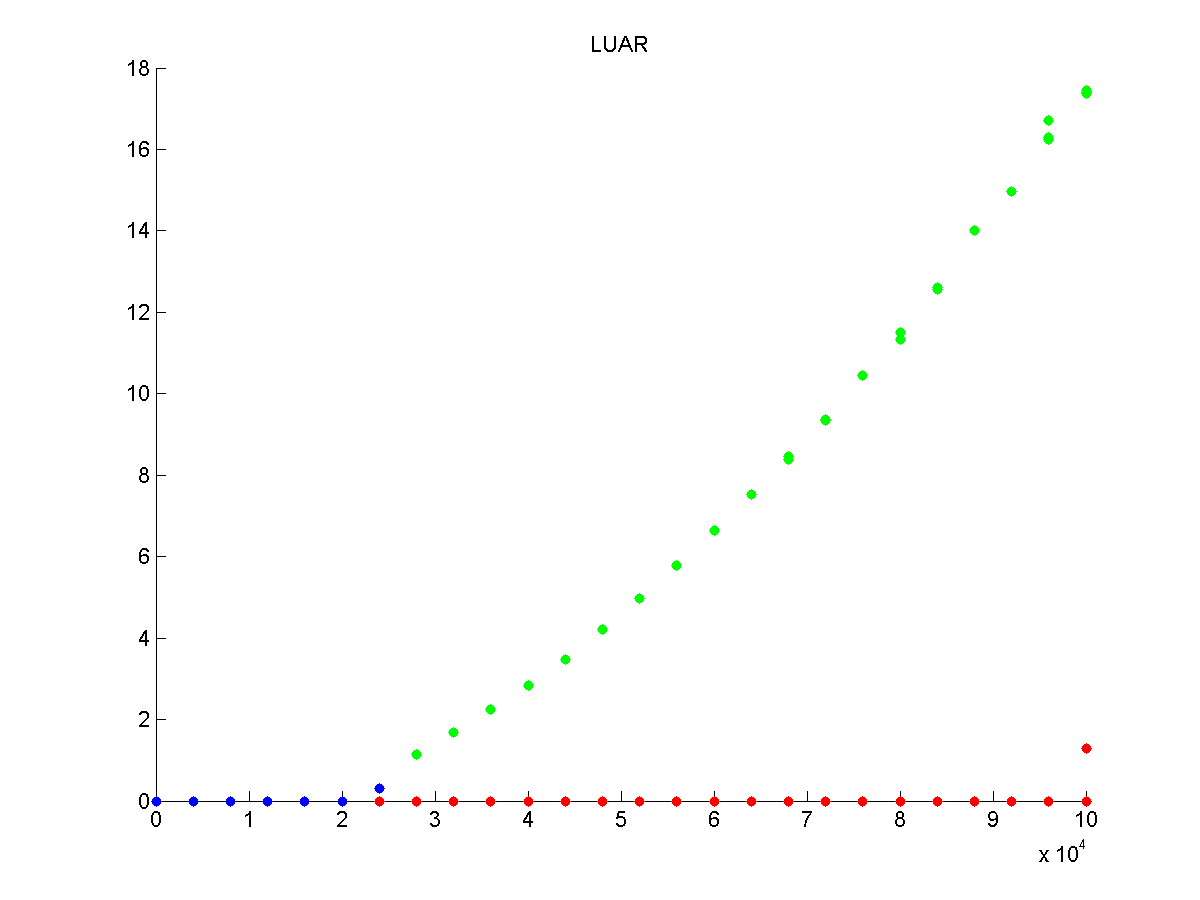

Supplement: Supplementary file 2 [file Presentation2.ZIP › LUAR.png]

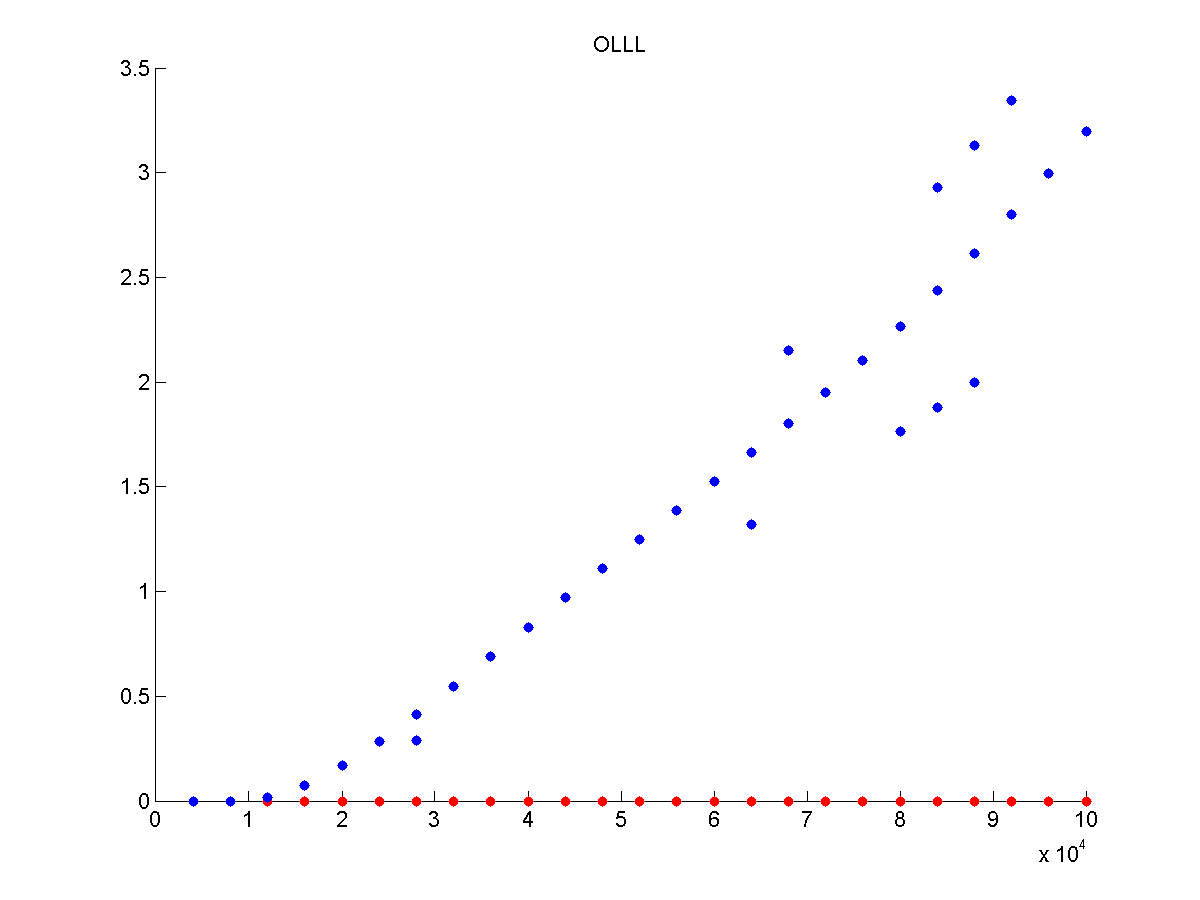

Supplement: Supplementary file 2 [file Presentation2.ZIP › OLLL.png]

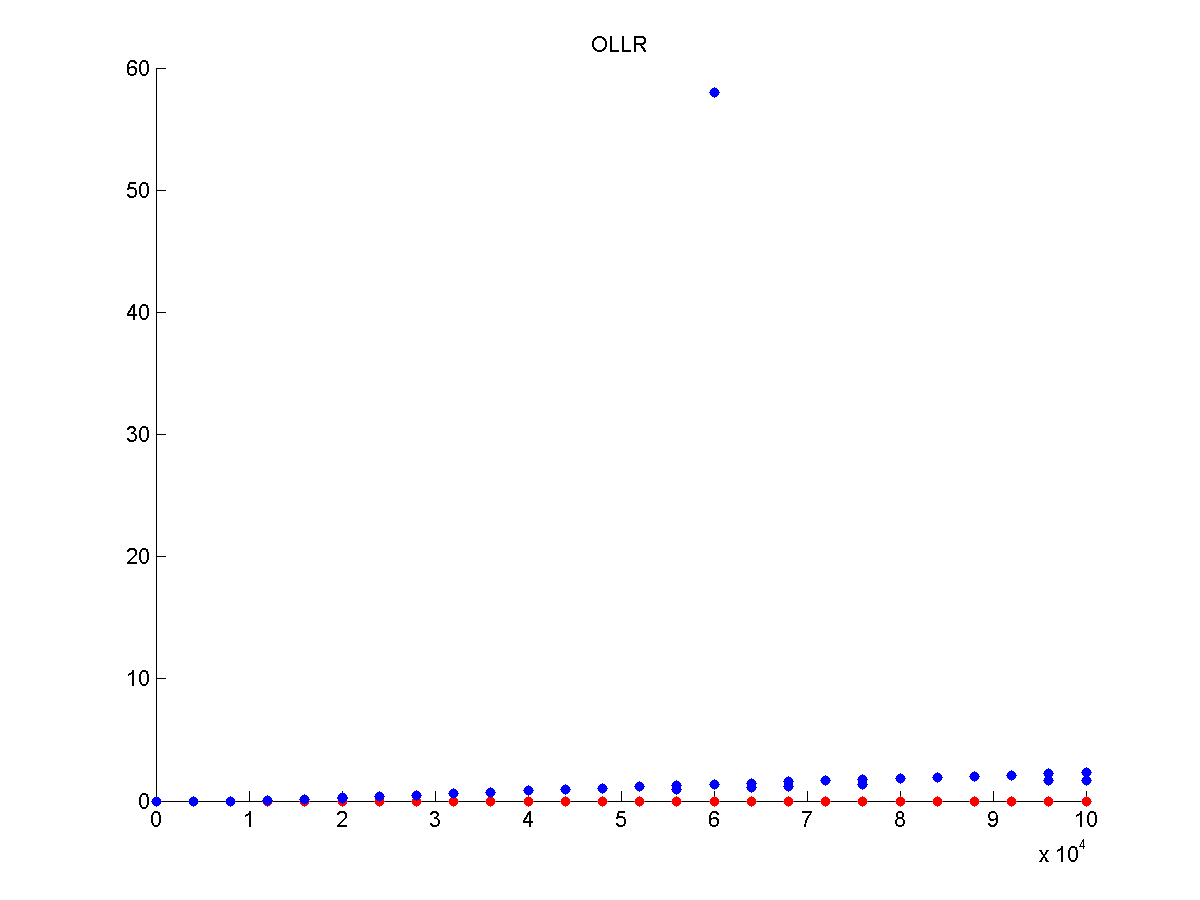

Supplement: Supplementary file 2 [file Presentation2.ZIP › OLLR.png]

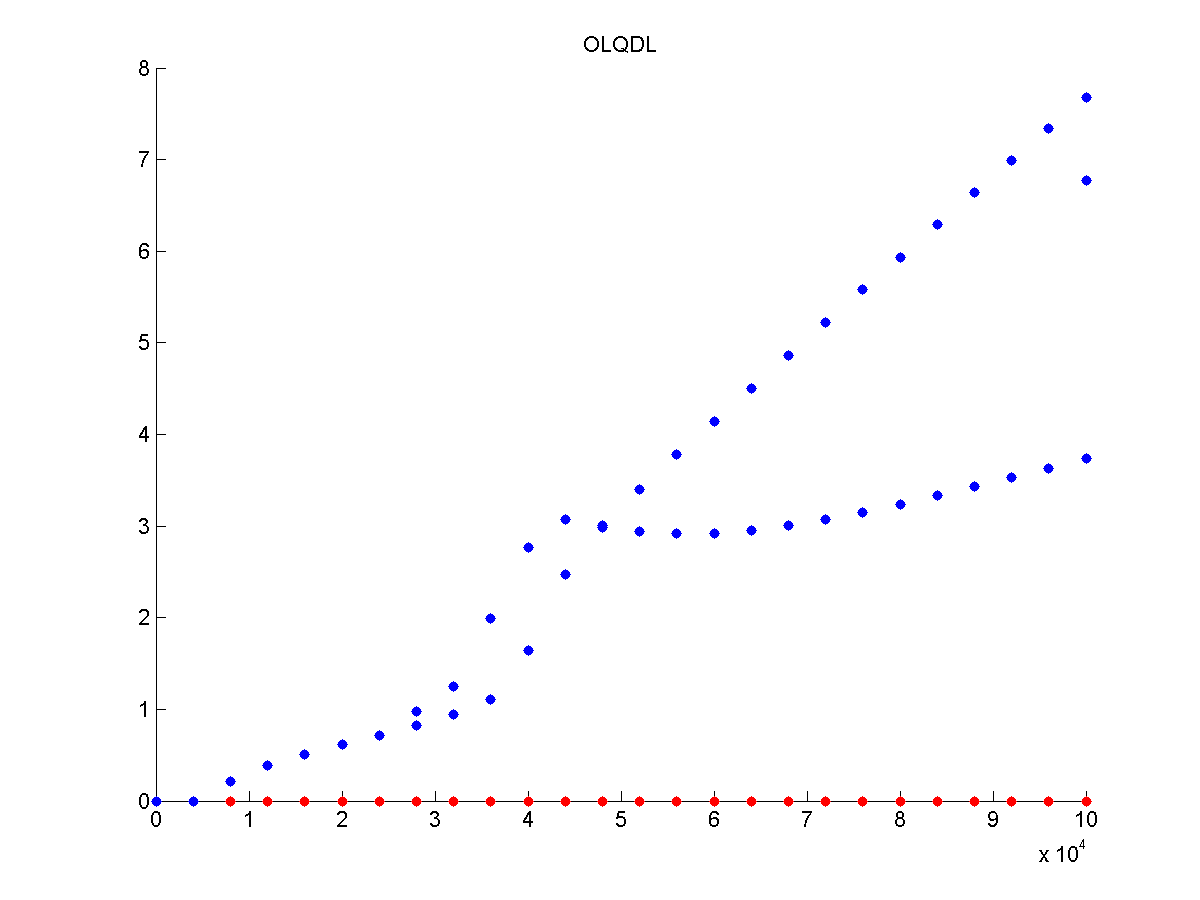

Supplement: Supplementary file 2 [file Presentation2.ZIP › OLQDL.png]

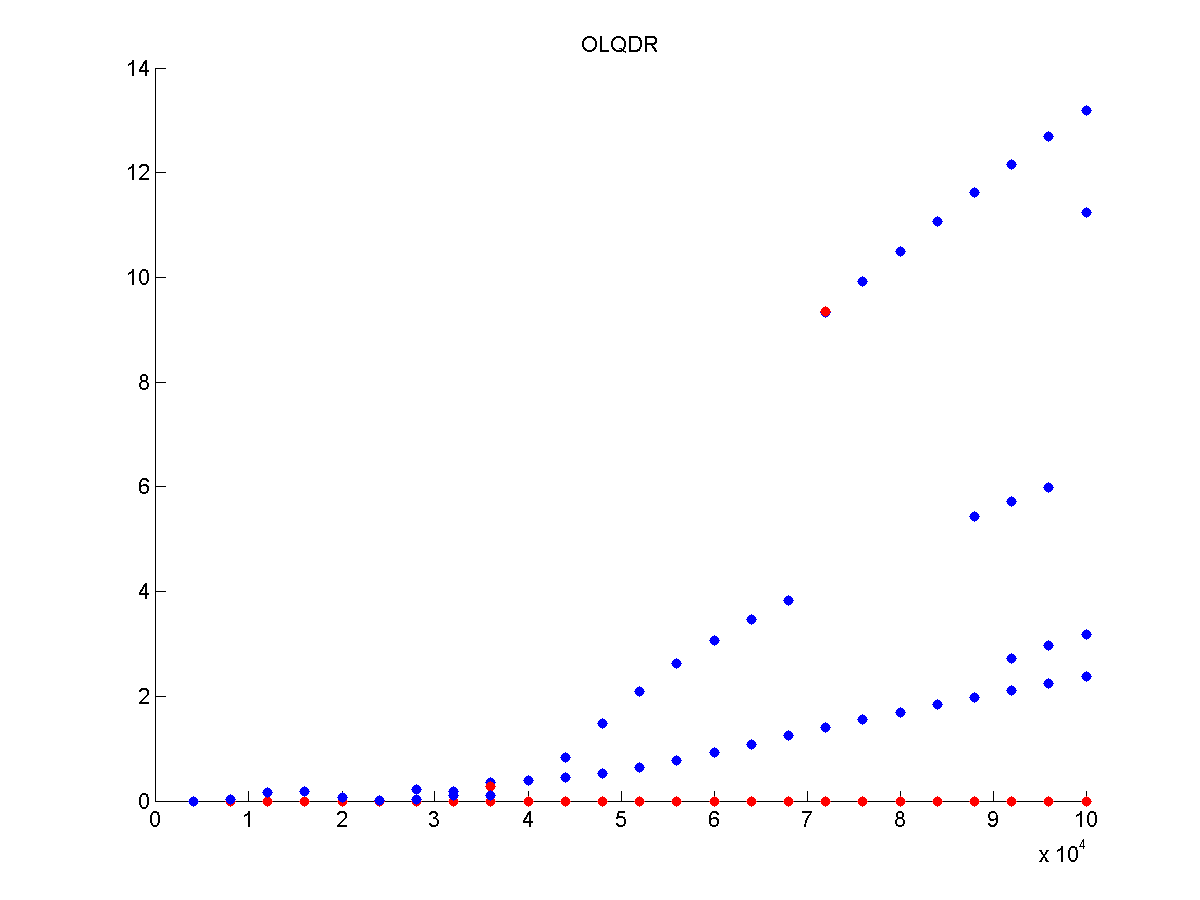

Supplement: Supplementary file 2 [file Presentation2.ZIP › OLQDR.png]

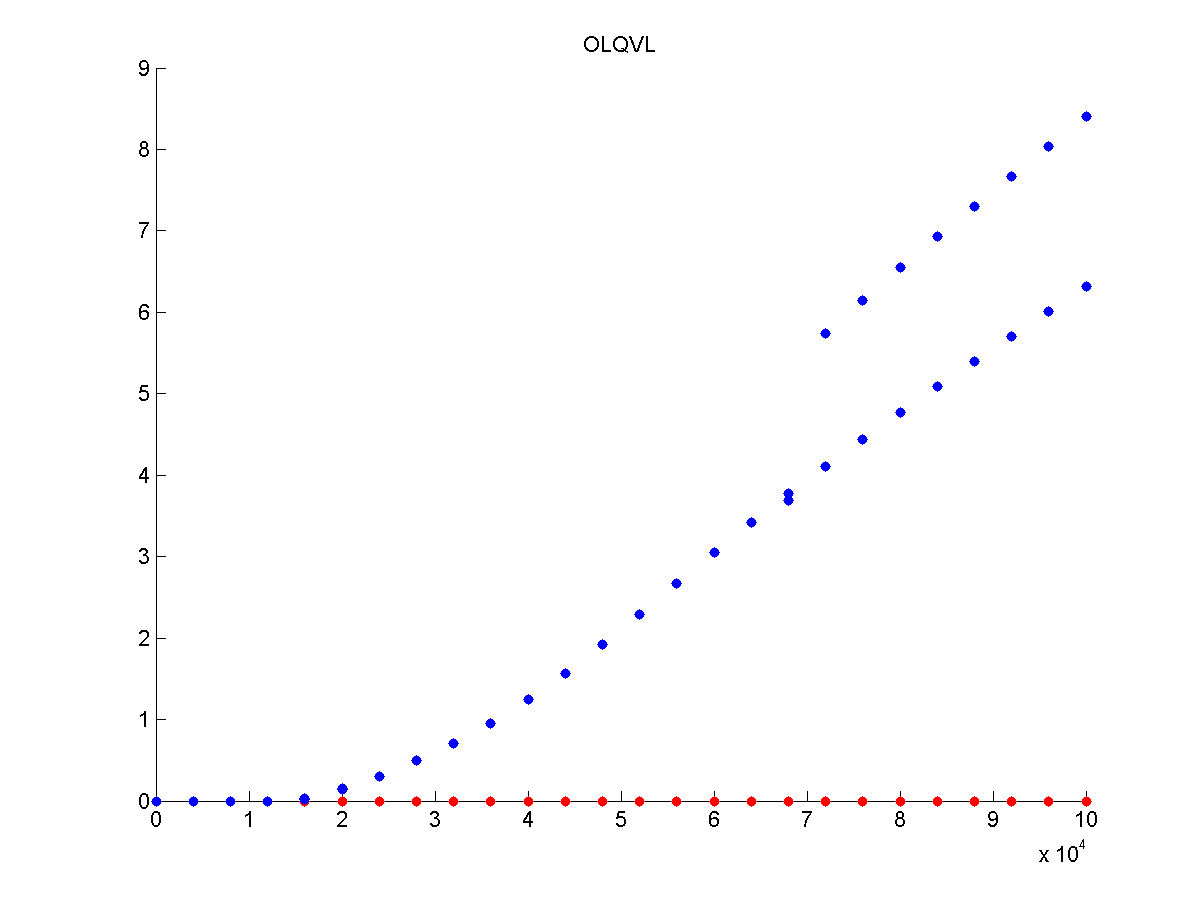

Supplement: Supplementary file 2 [file Presentation2.ZIP › OLQVL.png]

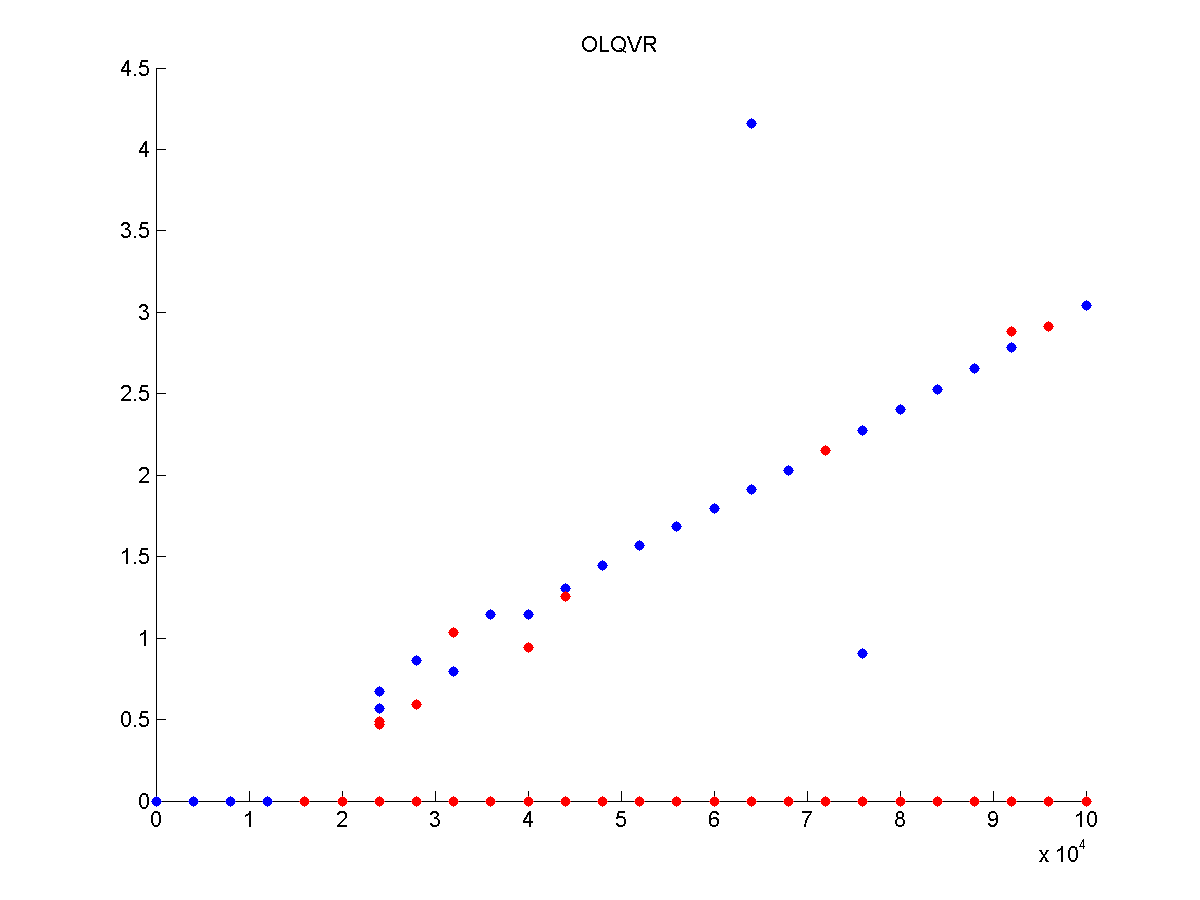

Supplement: Supplementary file 2 [file Presentation2.ZIP › OLQVR.png]

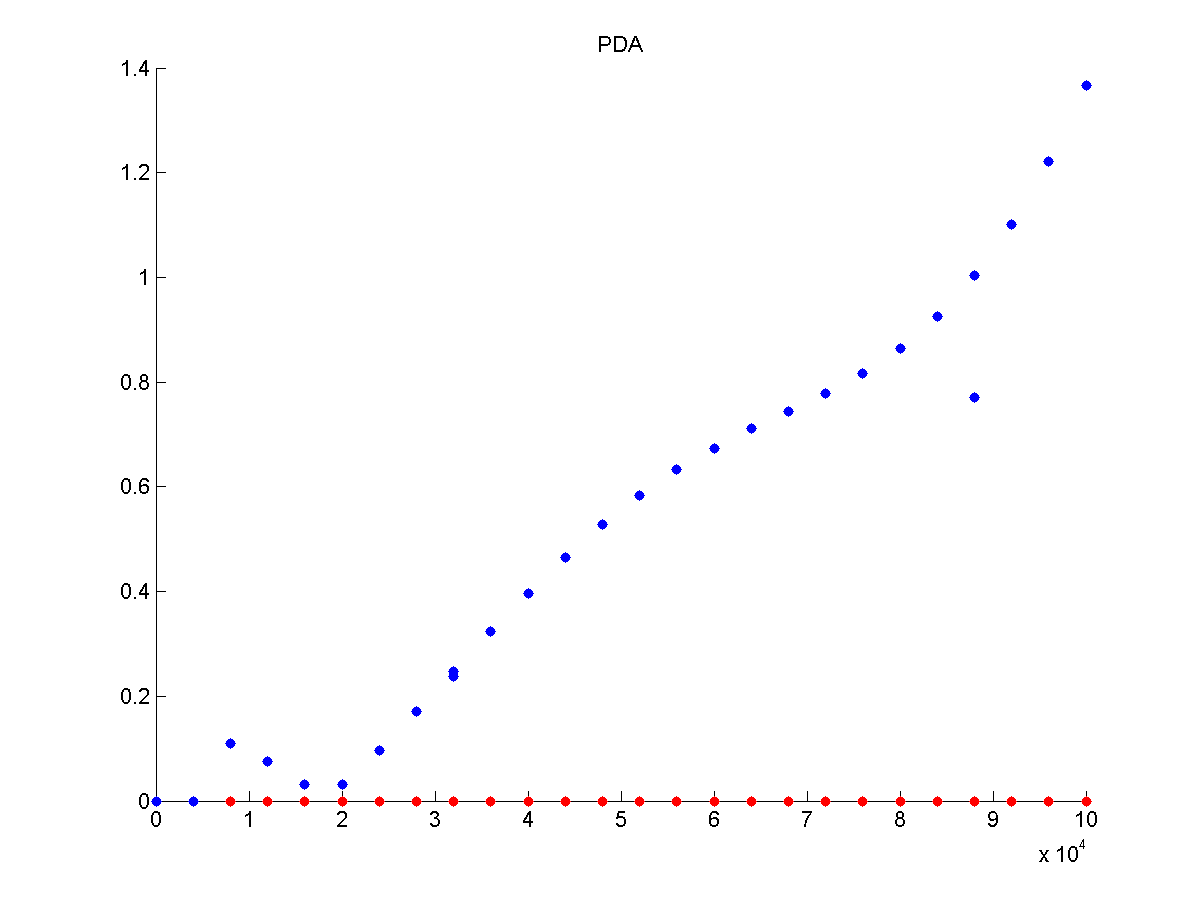

Supplement: Supplementary file 2 [file Presentation2.ZIP › PDA.png]

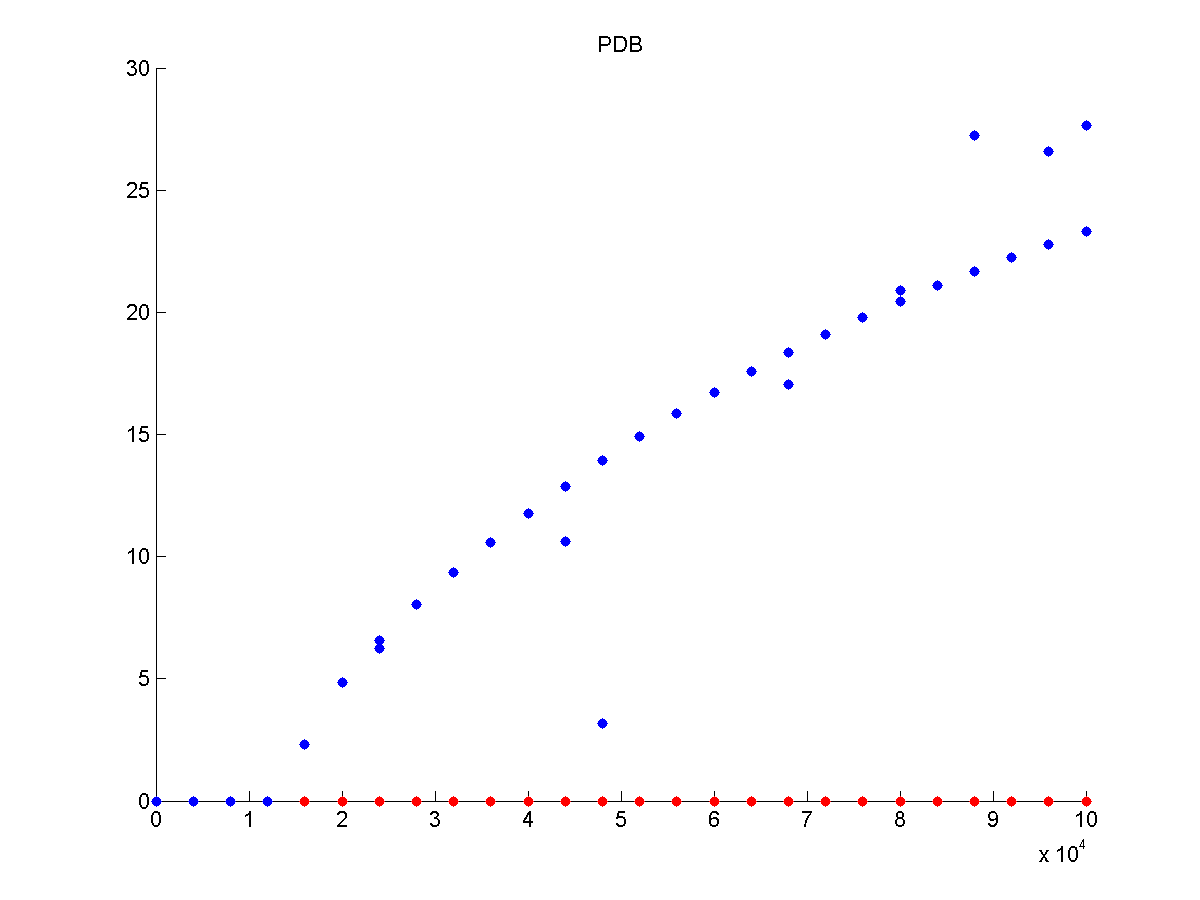

Supplement: Supplementary file 2 [file Presentation2.ZIP › PDB.png]

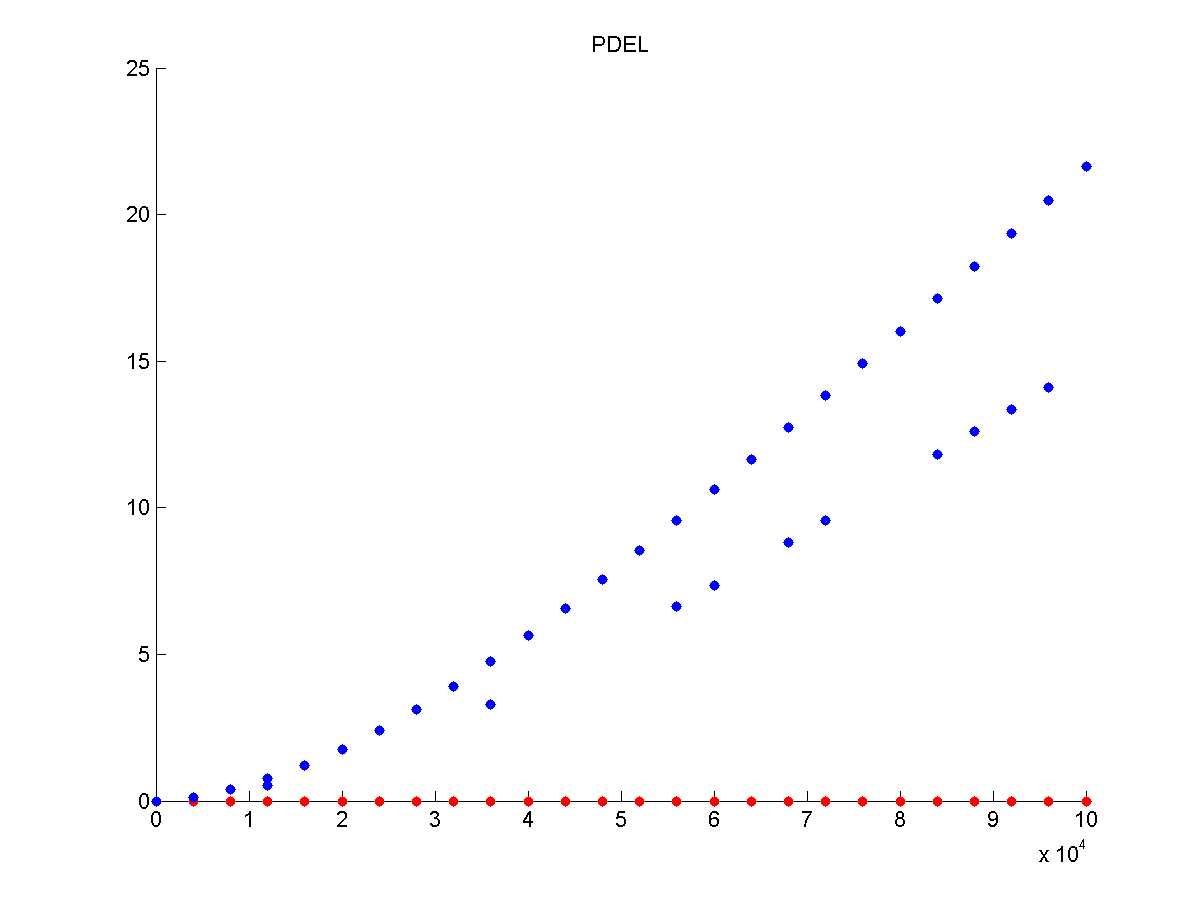

Supplement: Supplementary file 2 [file Presentation2.ZIP › PDEL.png]

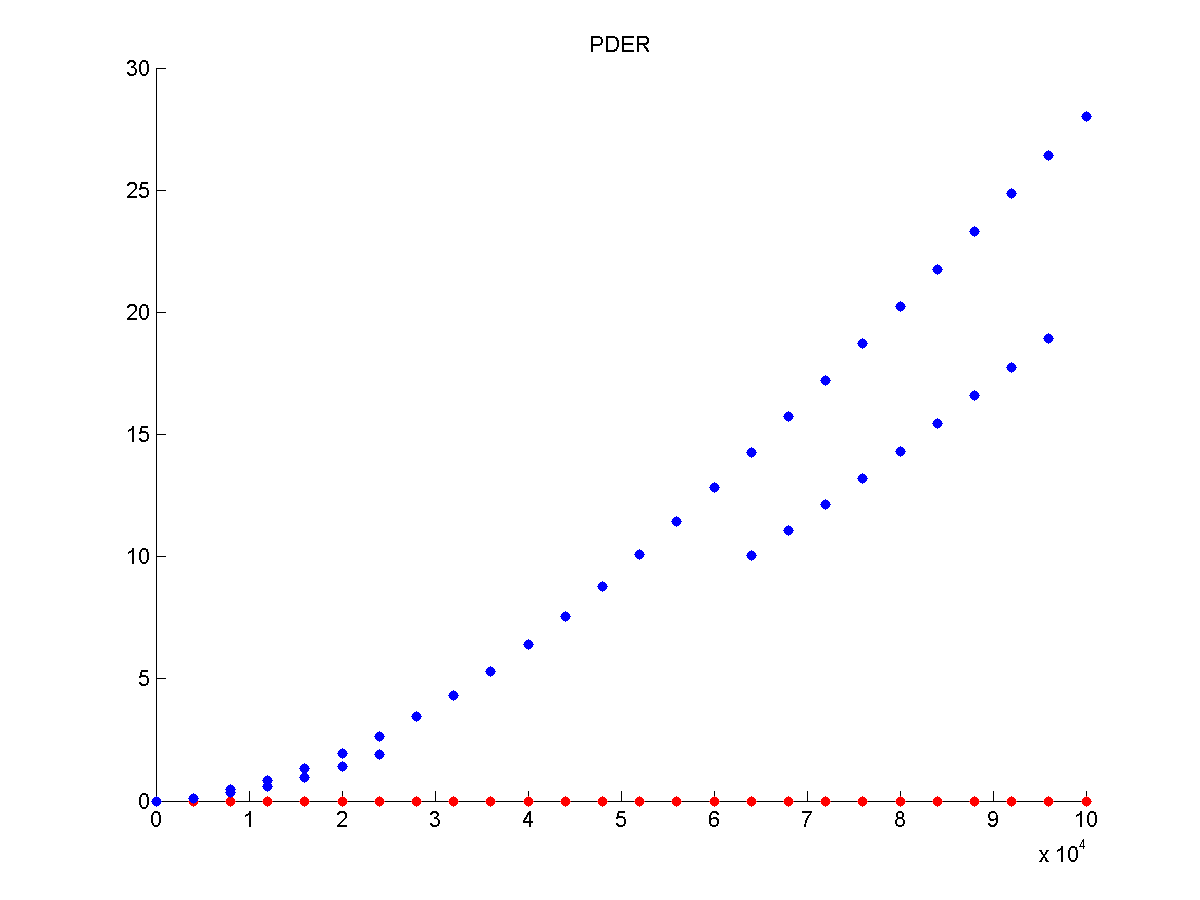

Supplement: Supplementary file 2 [file Presentation2.ZIP › PDER.png]

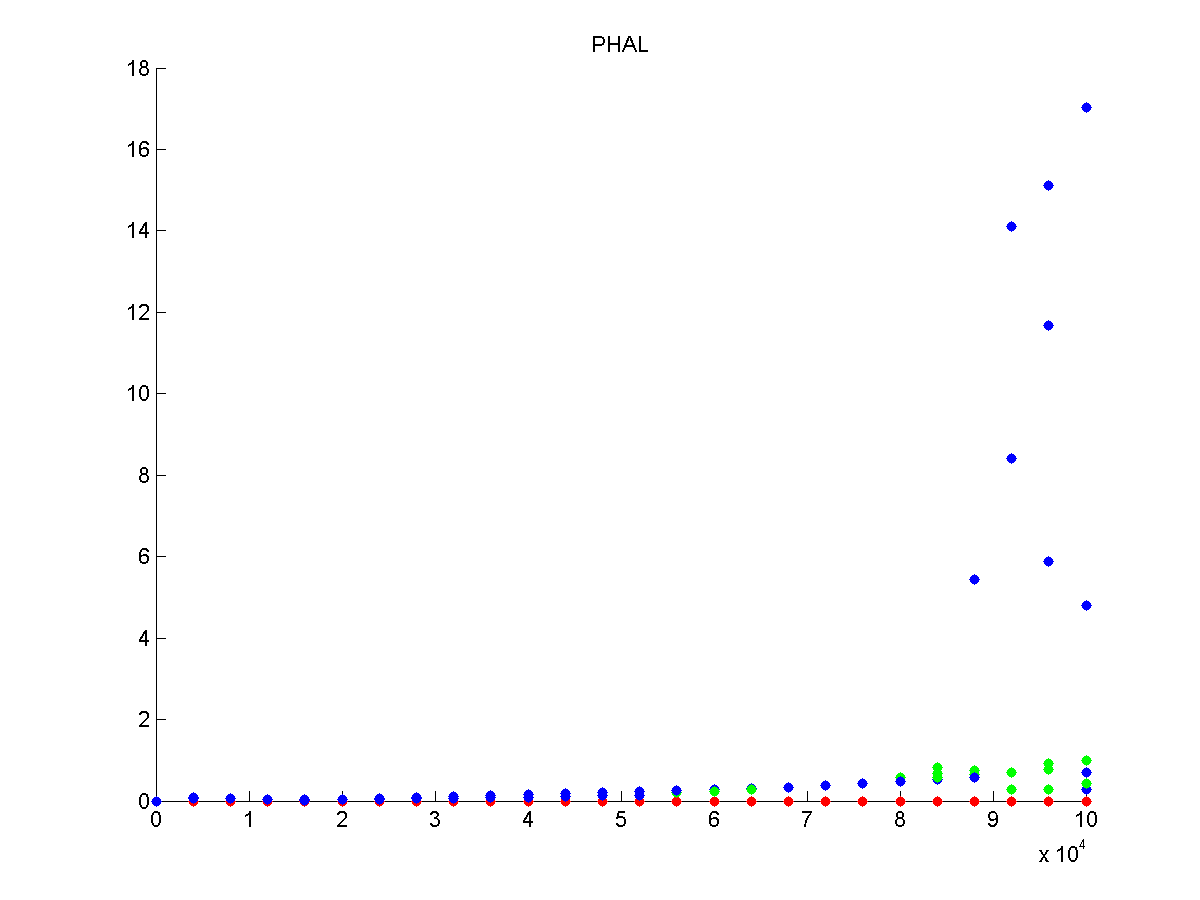

Supplement: Supplementary file 2 [file Presentation2.ZIP › PHAL.png]

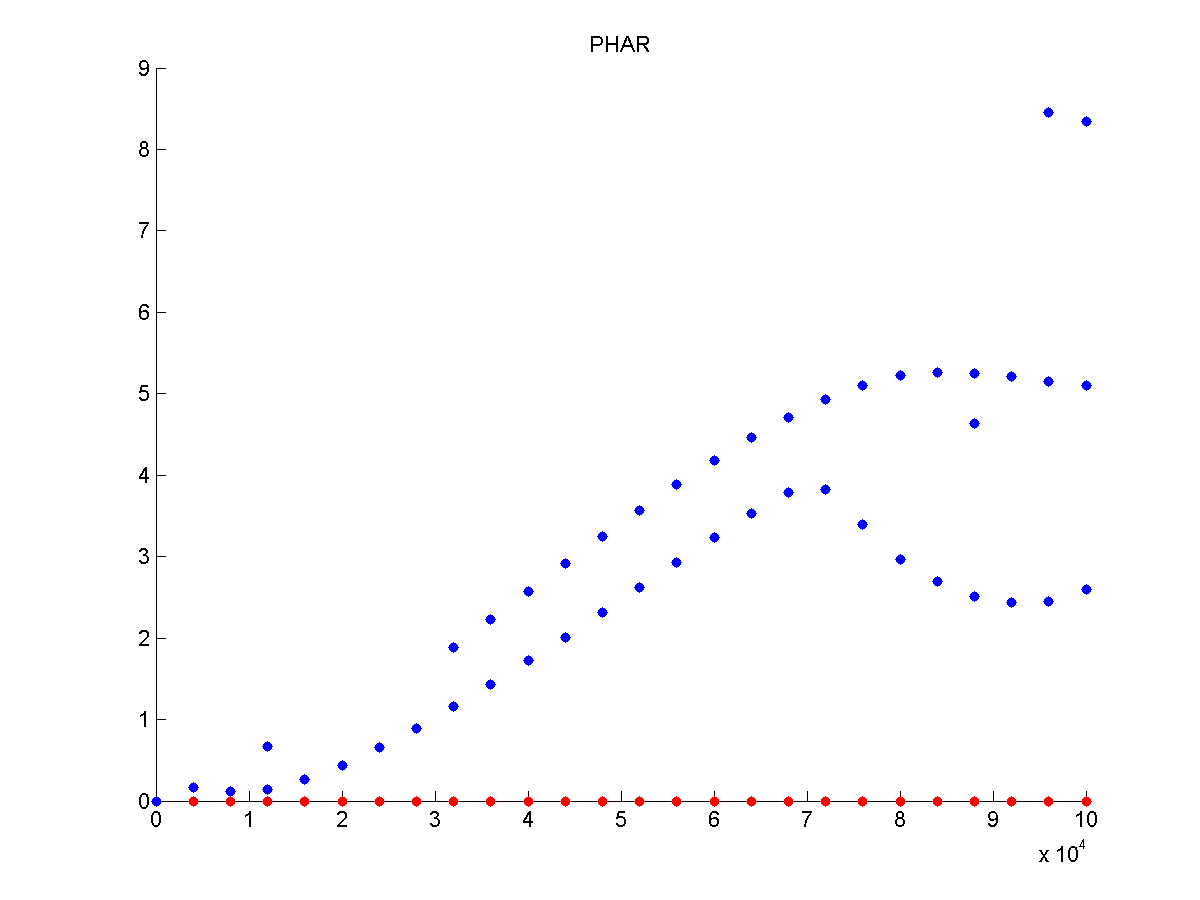

Supplement: Supplementary file 2 [file Presentation2.ZIP › PHAR.png]

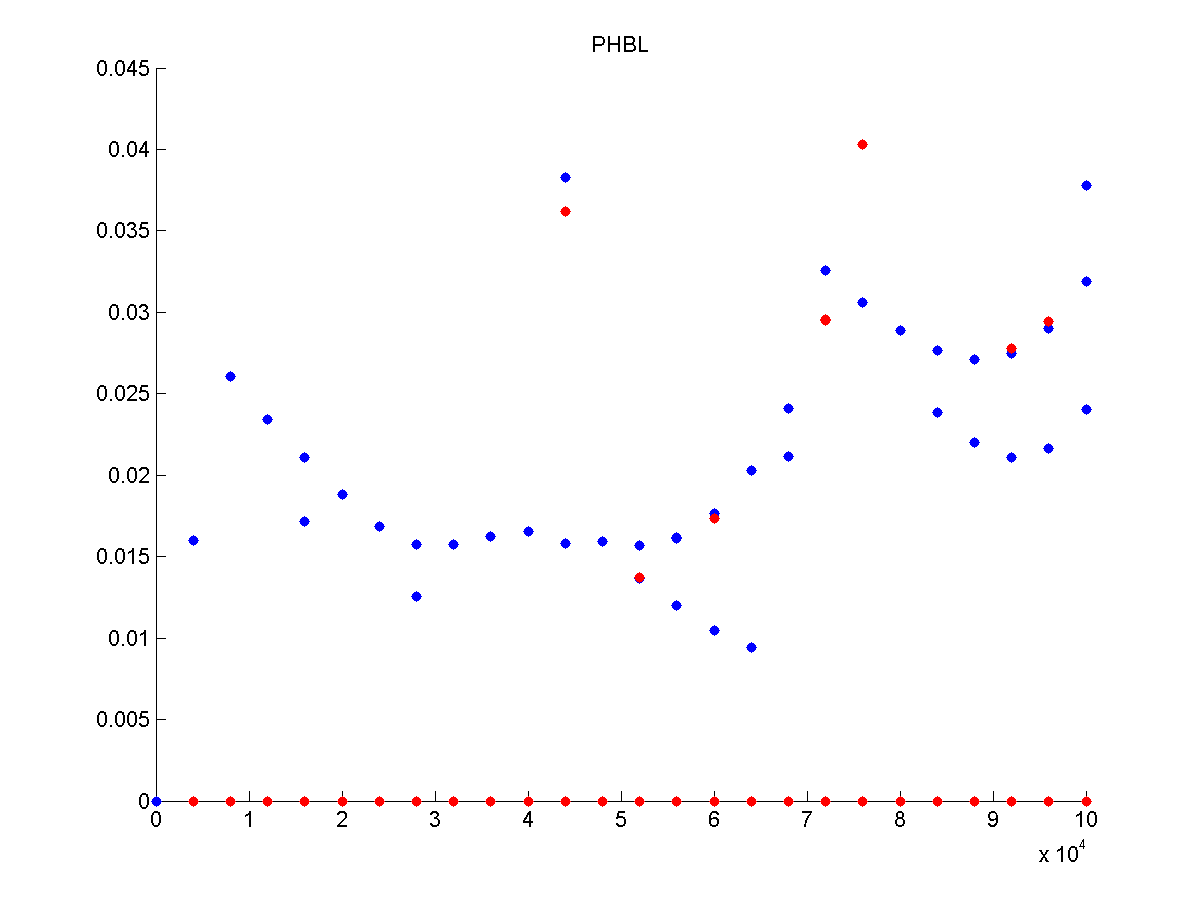

Supplement: Supplementary file 2 [file Presentation2.ZIP › PHBL.png]

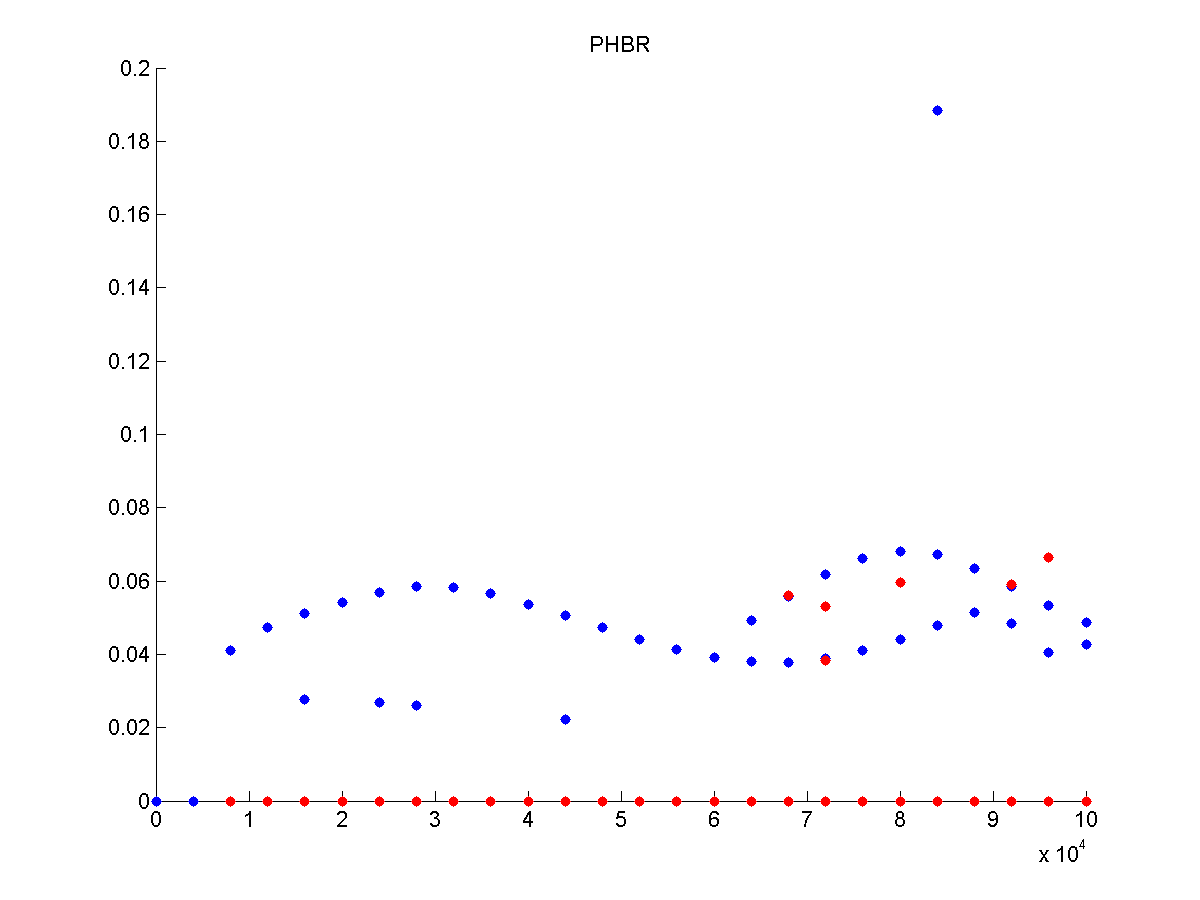

Supplement: Supplementary file 2 [file Presentation2.ZIP › PHBR.png]

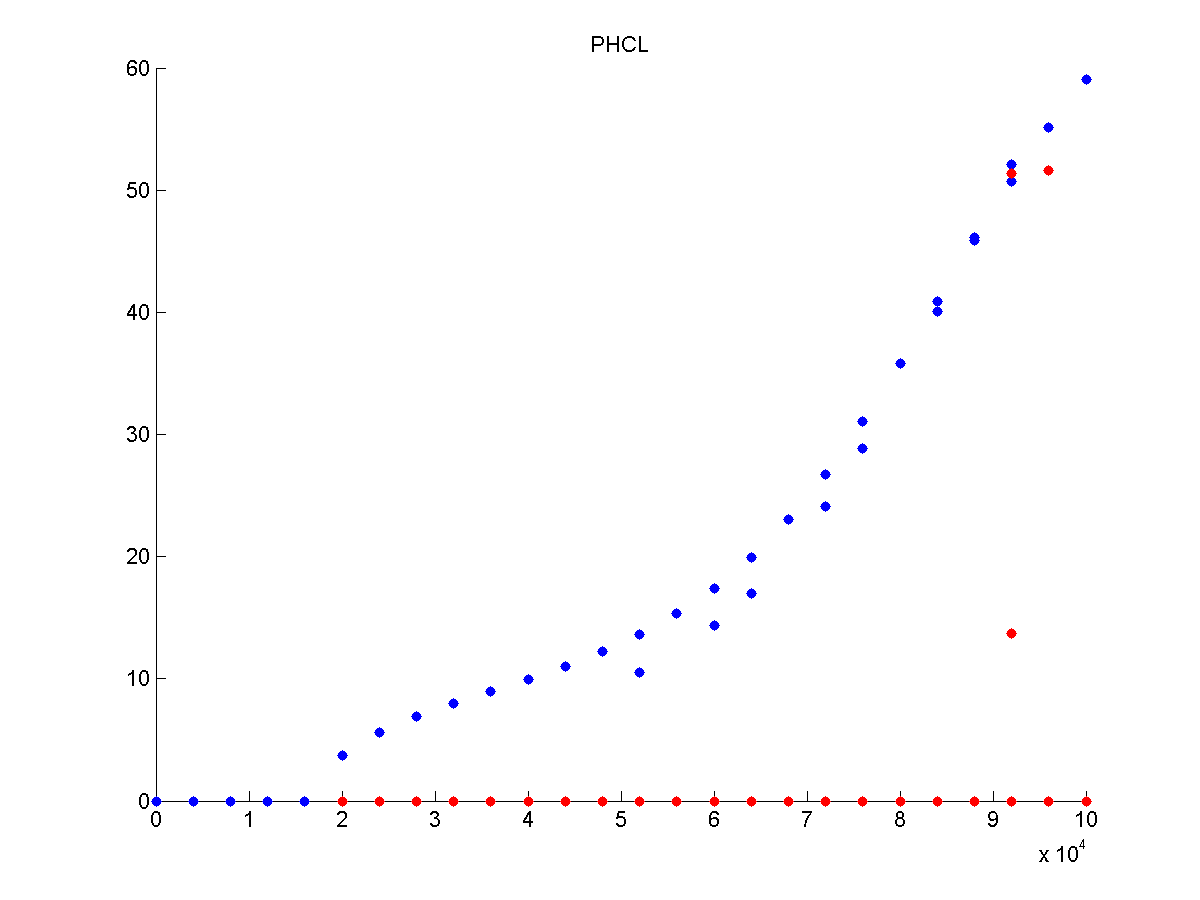

Supplement: Supplementary file 2 [file Presentation2.ZIP › PHCL.png]

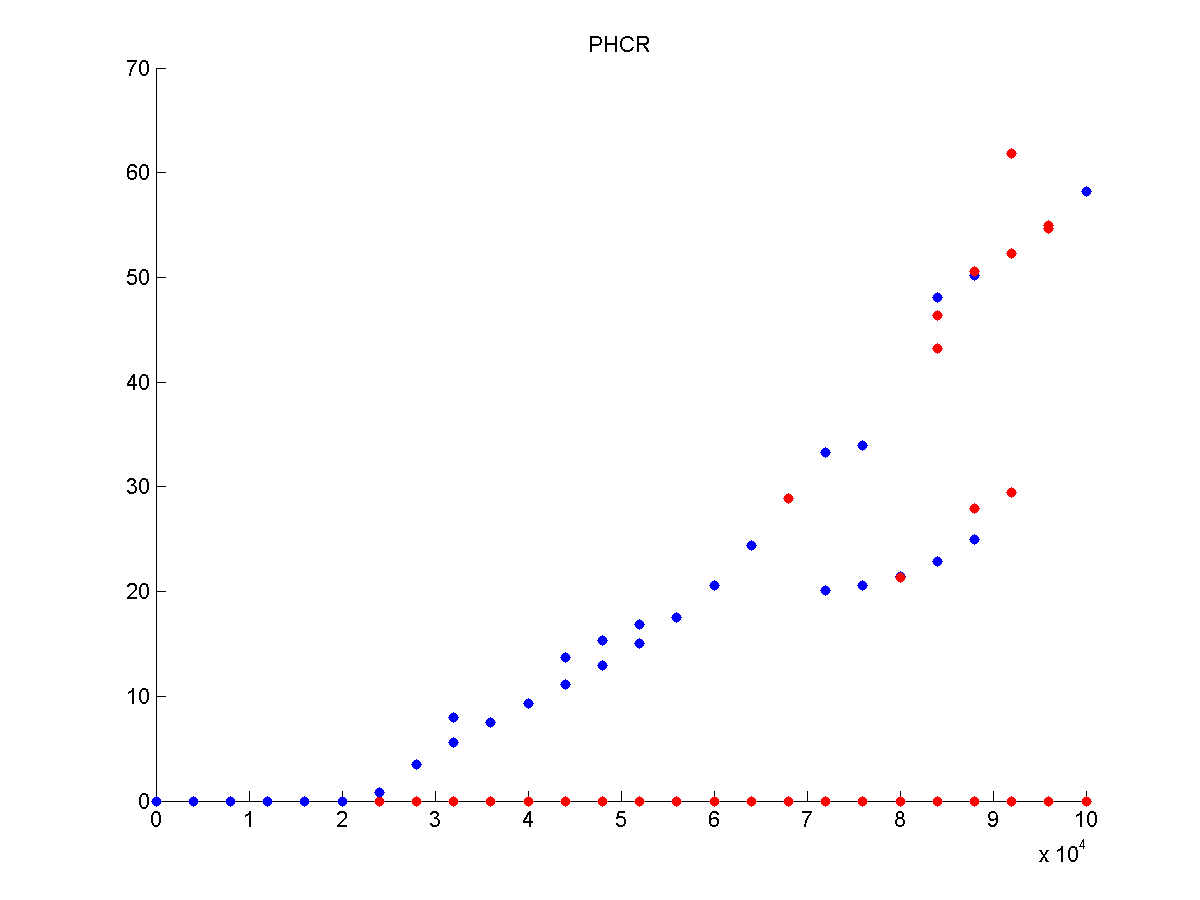

Supplement: Supplementary file 2 [file Presentation2.ZIP › PHCR.png]

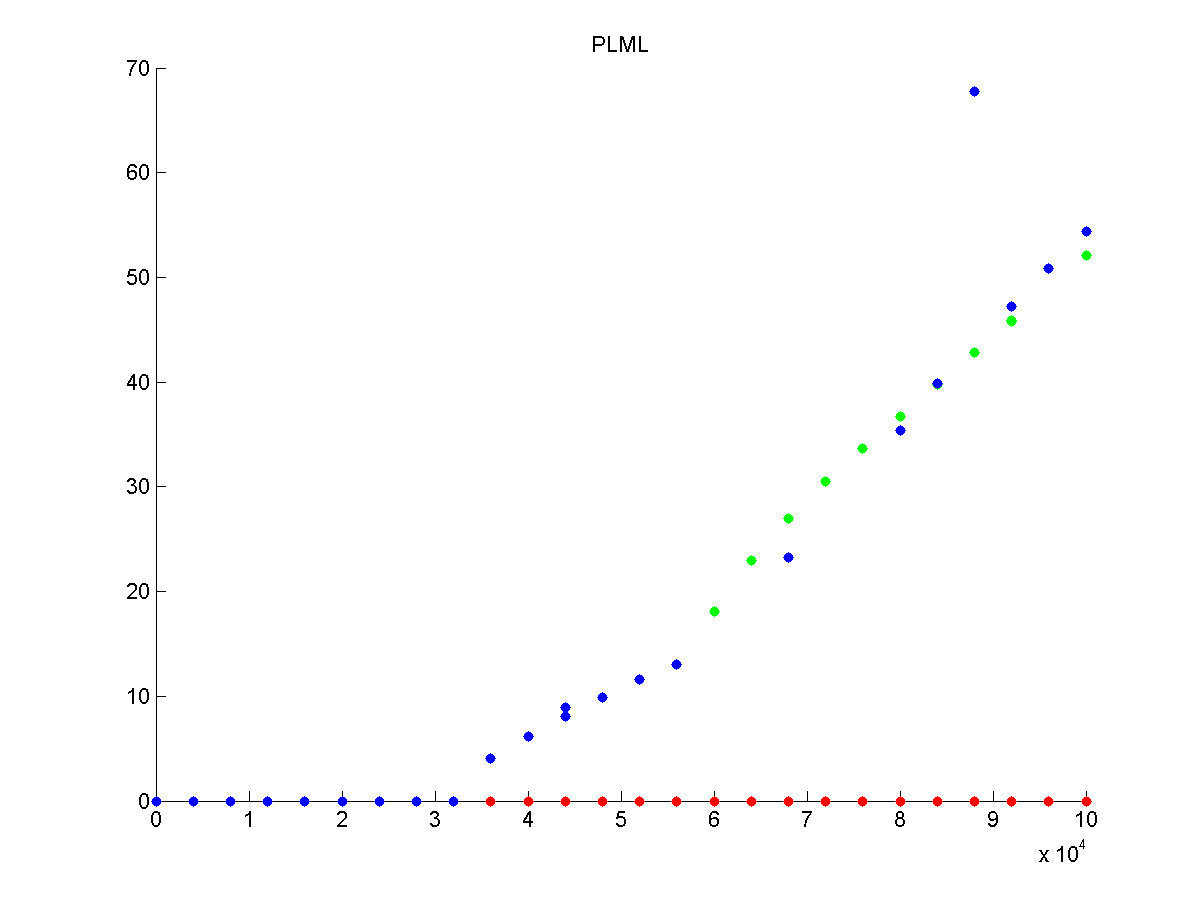

Supplement: Supplementary file 2 [file Presentation2.ZIP › PLML.png]

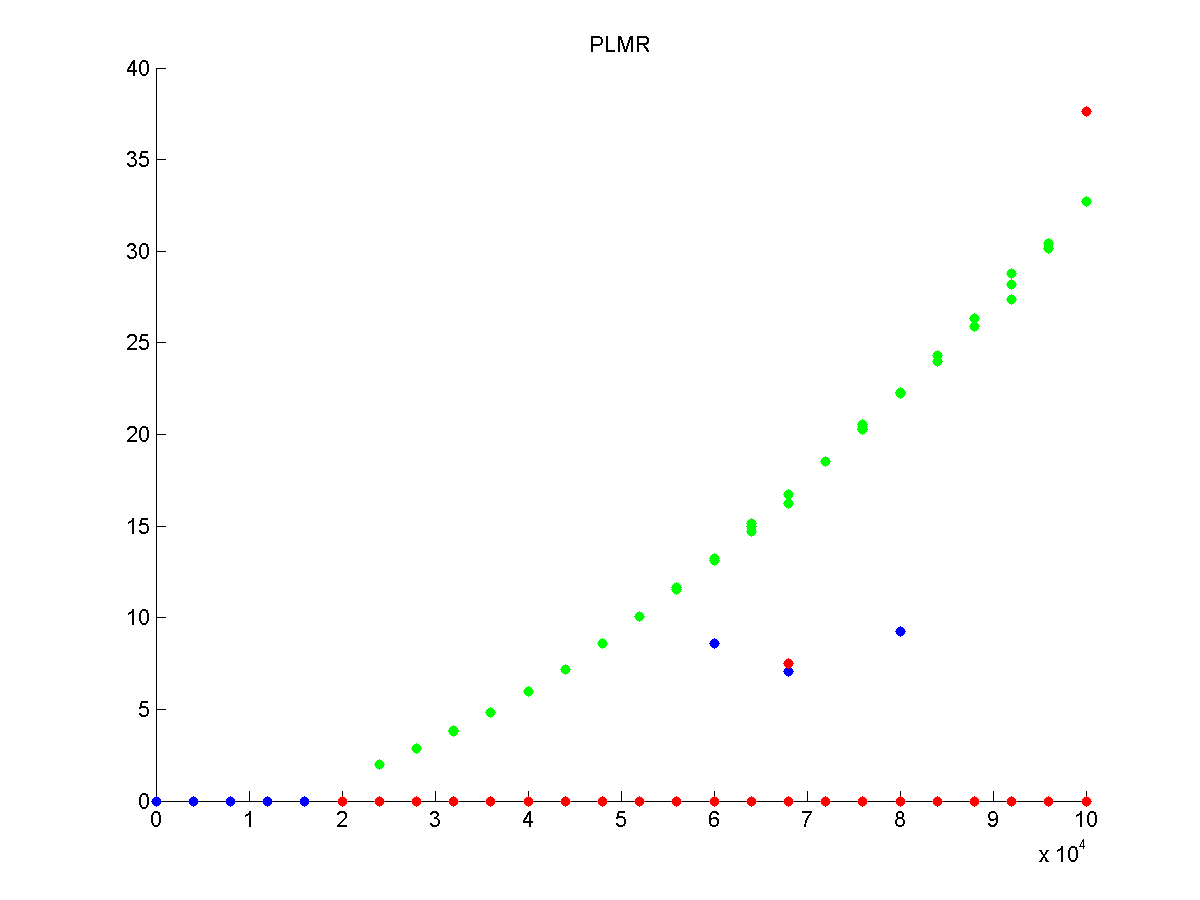

Supplement: Supplementary file 2 [file Presentation2.ZIP › PLMR.png]

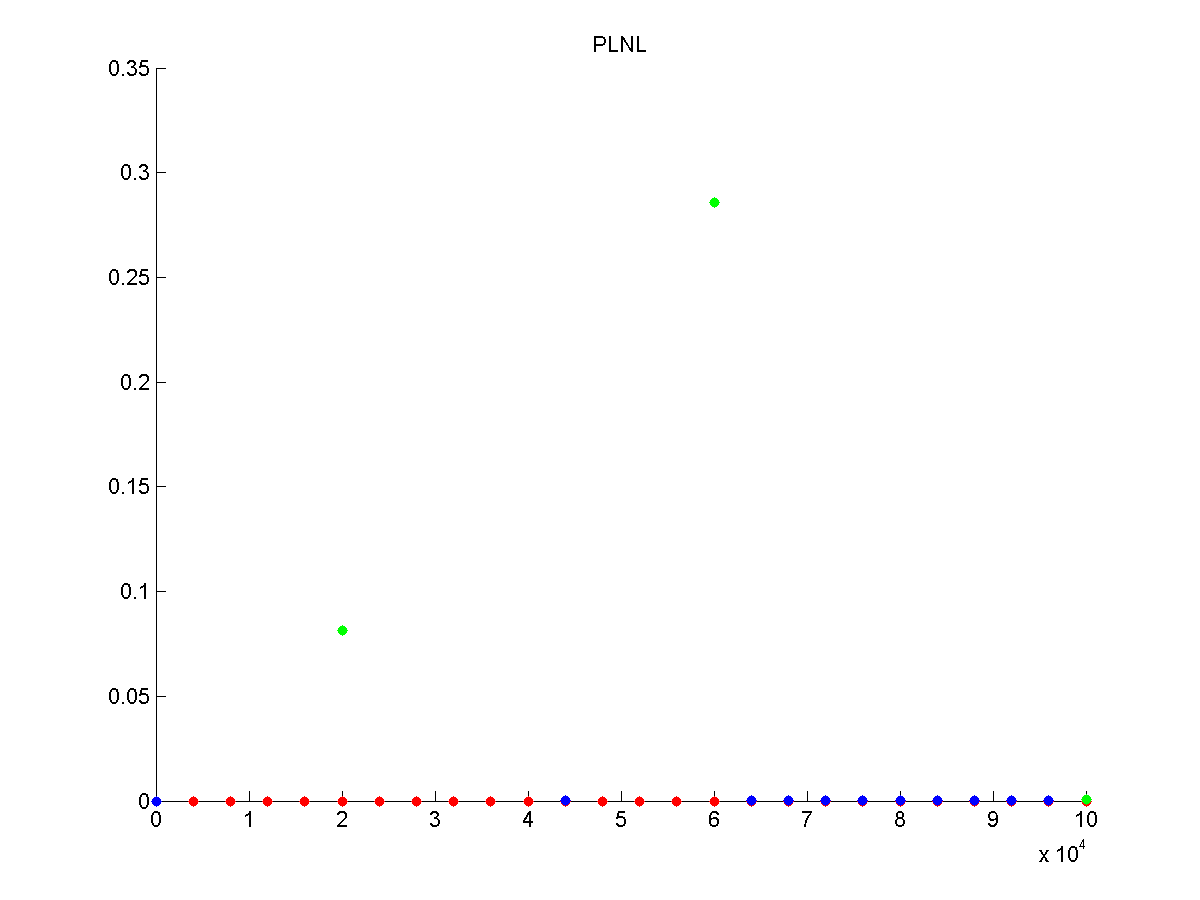

Supplement: Supplementary file 2 [file Presentation2.ZIP › PLNL.png]

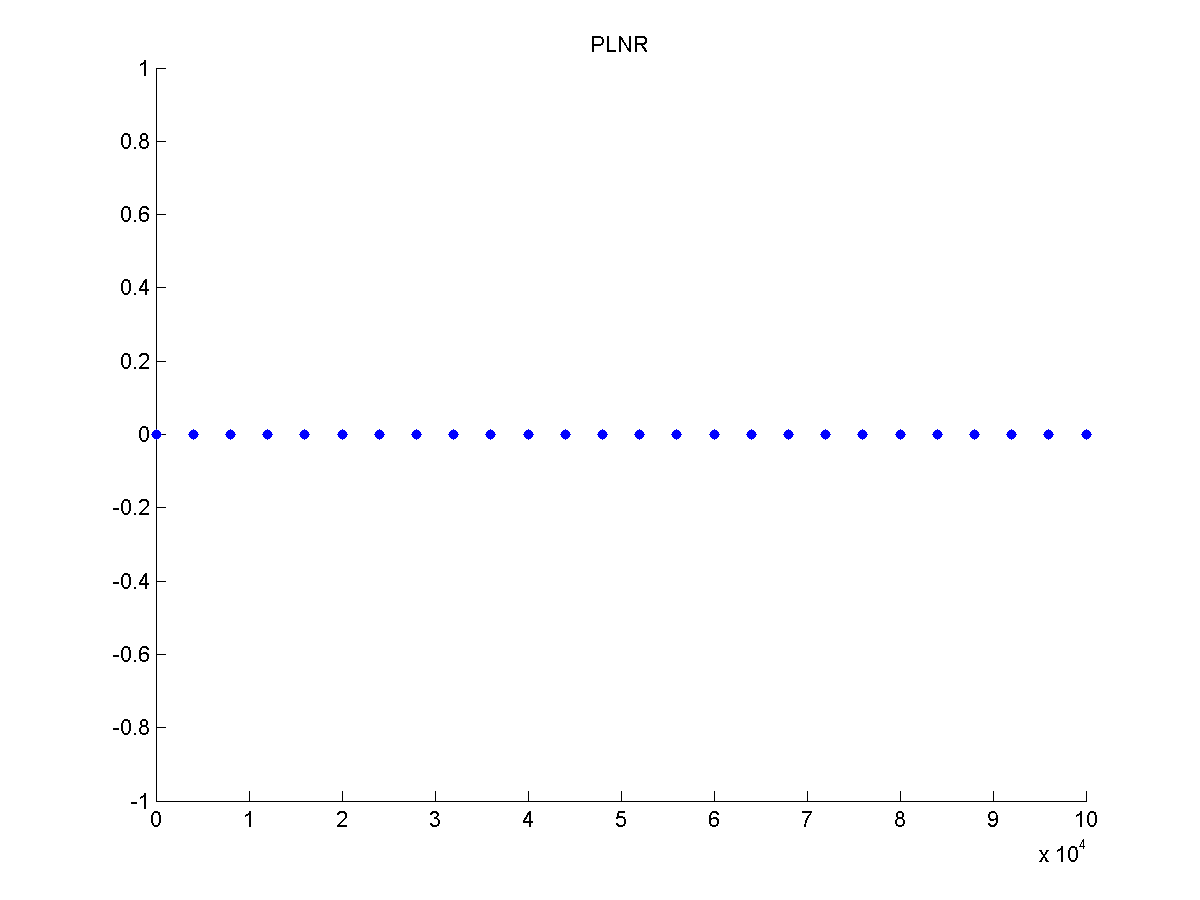

Supplement: Supplementary file 2 [file Presentation2.ZIP › PLNR.png]

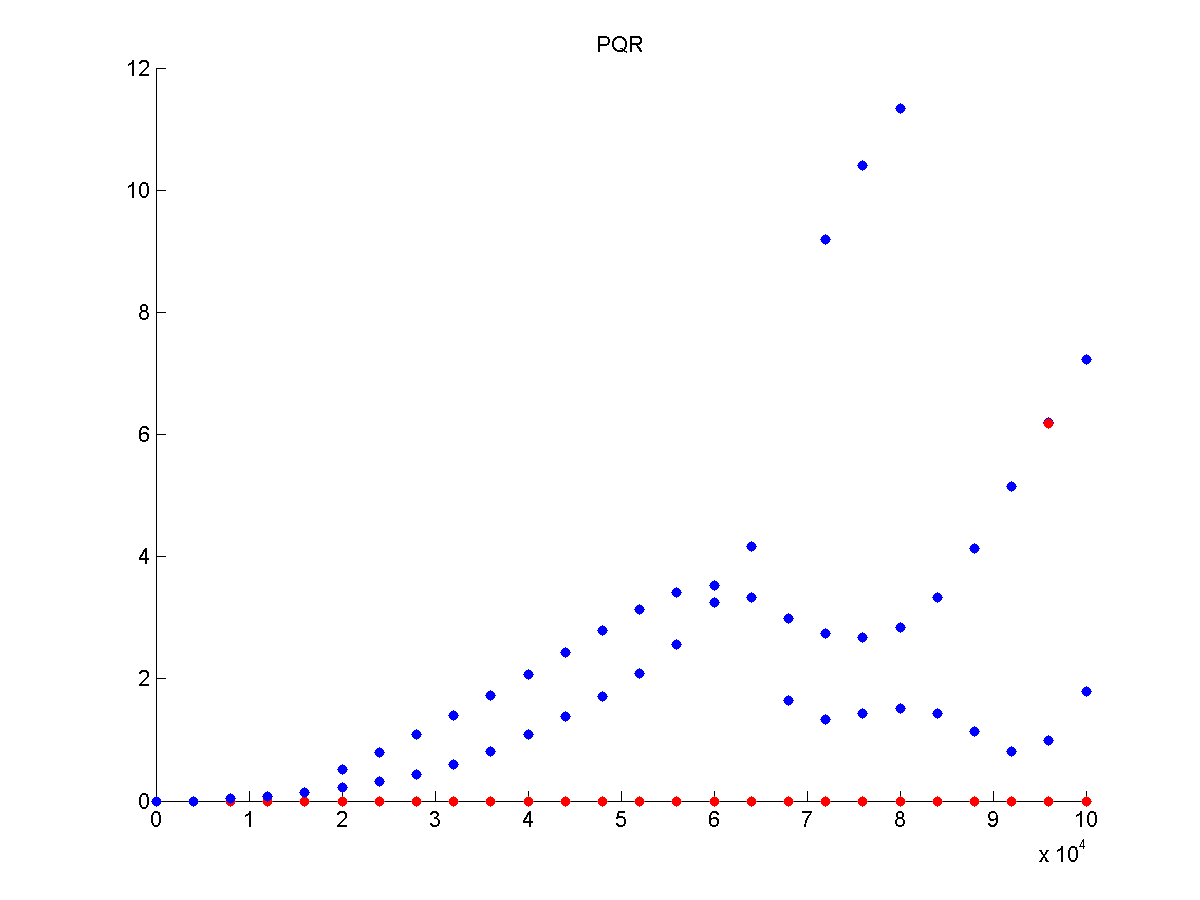

Supplement: Supplementary file 2 [file Presentation2.ZIP › PQR.png]

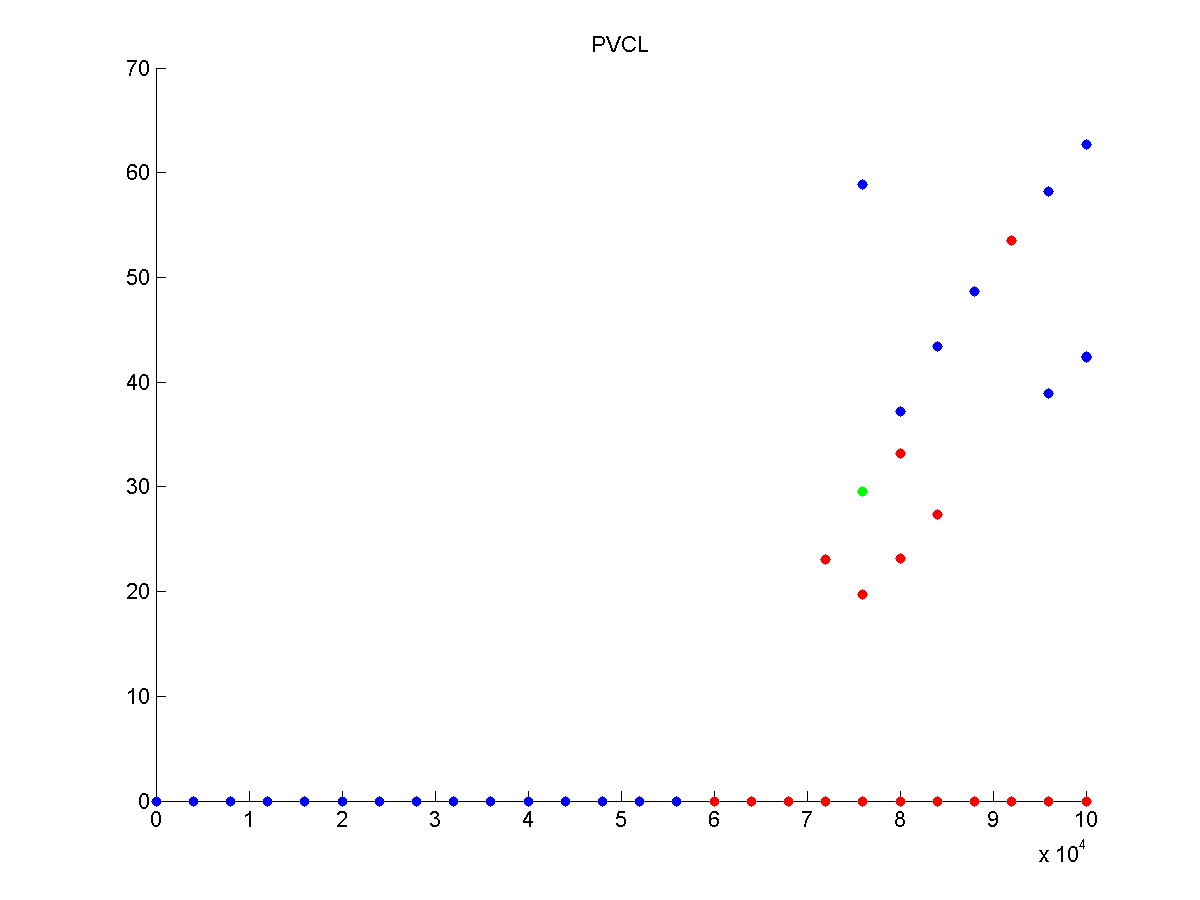

Supplement: Supplementary file 2 [file Presentation2.ZIP › PVCL.png]

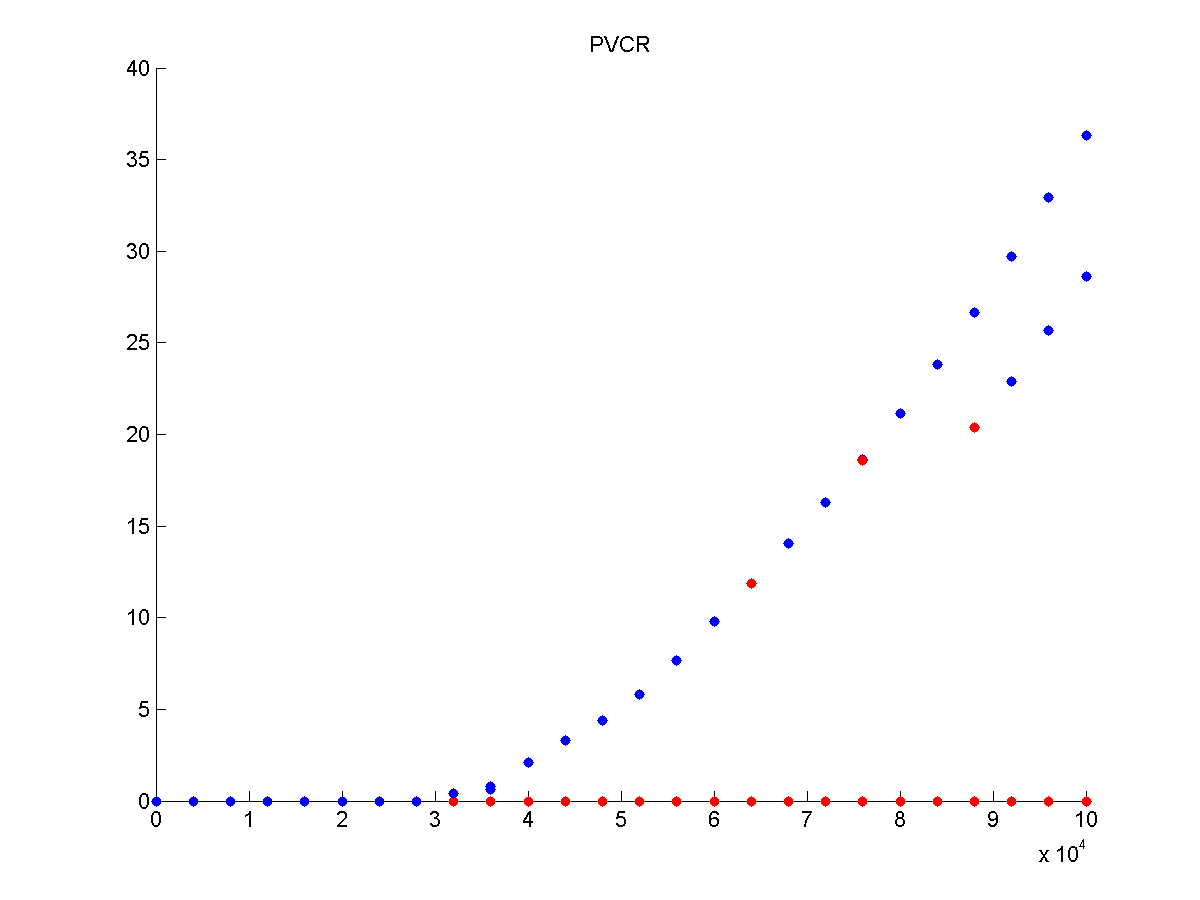

Supplement: Supplementary file 2 [file Presentation2.ZIP › PVCR.png]

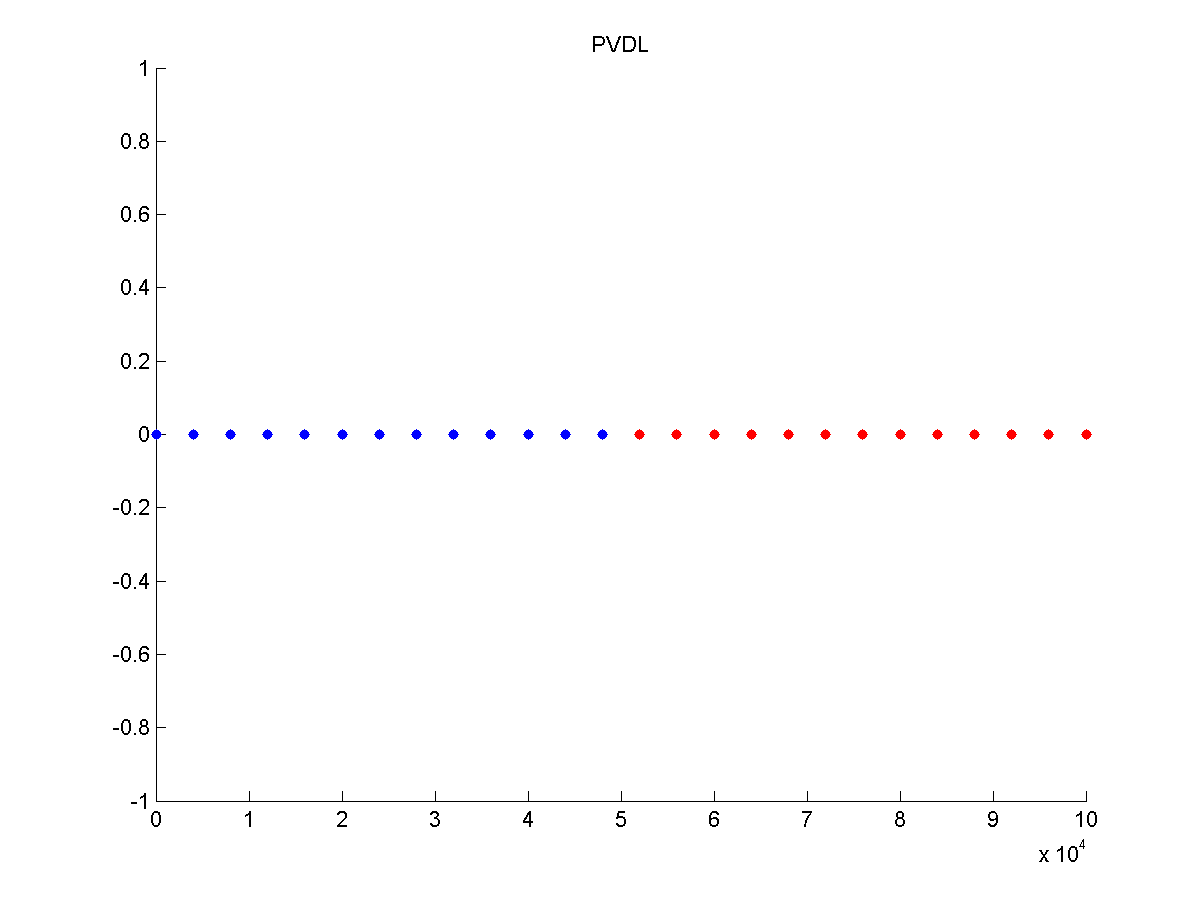

Supplement: Supplementary file 2 [file Presentation2.ZIP › PVDL.png]

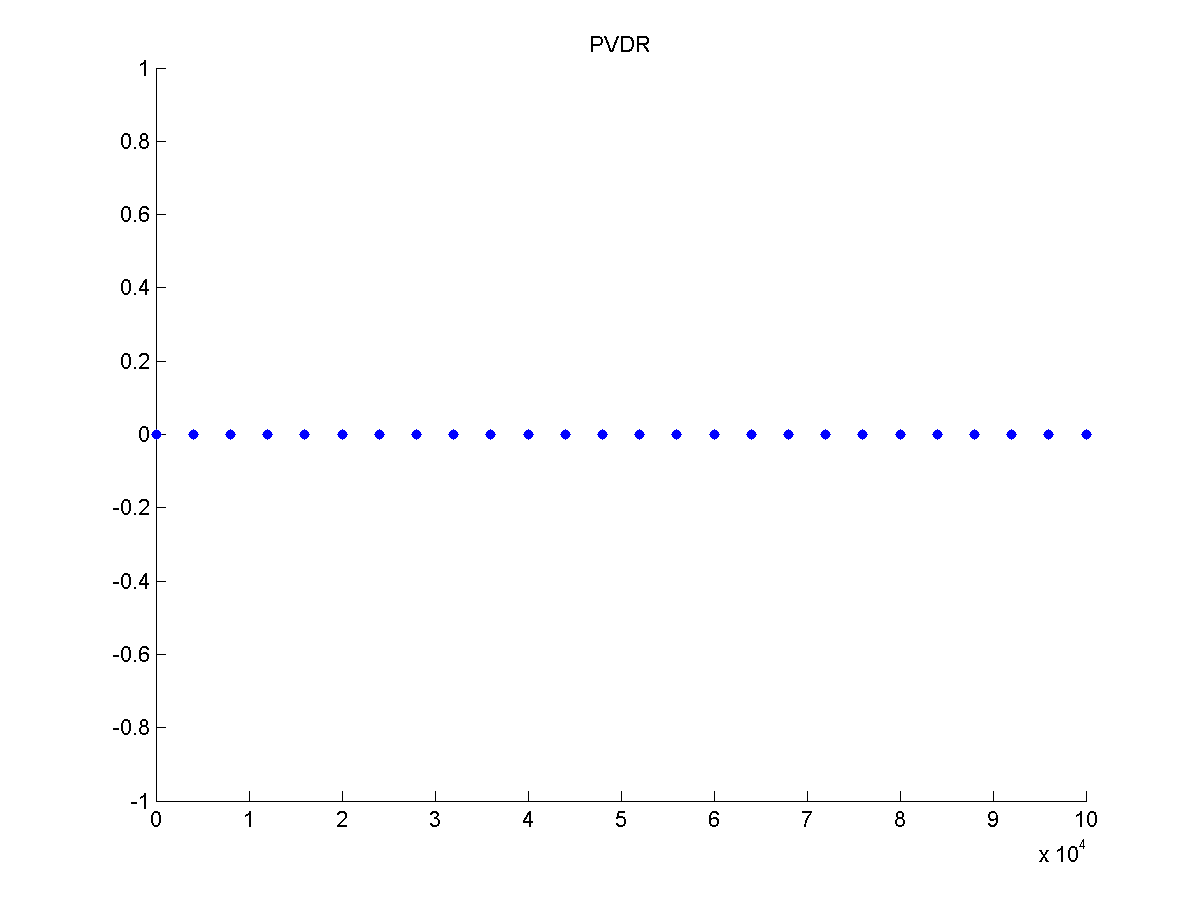

Supplement: Supplementary file 2 [file Presentation2.ZIP › PVDR.png]

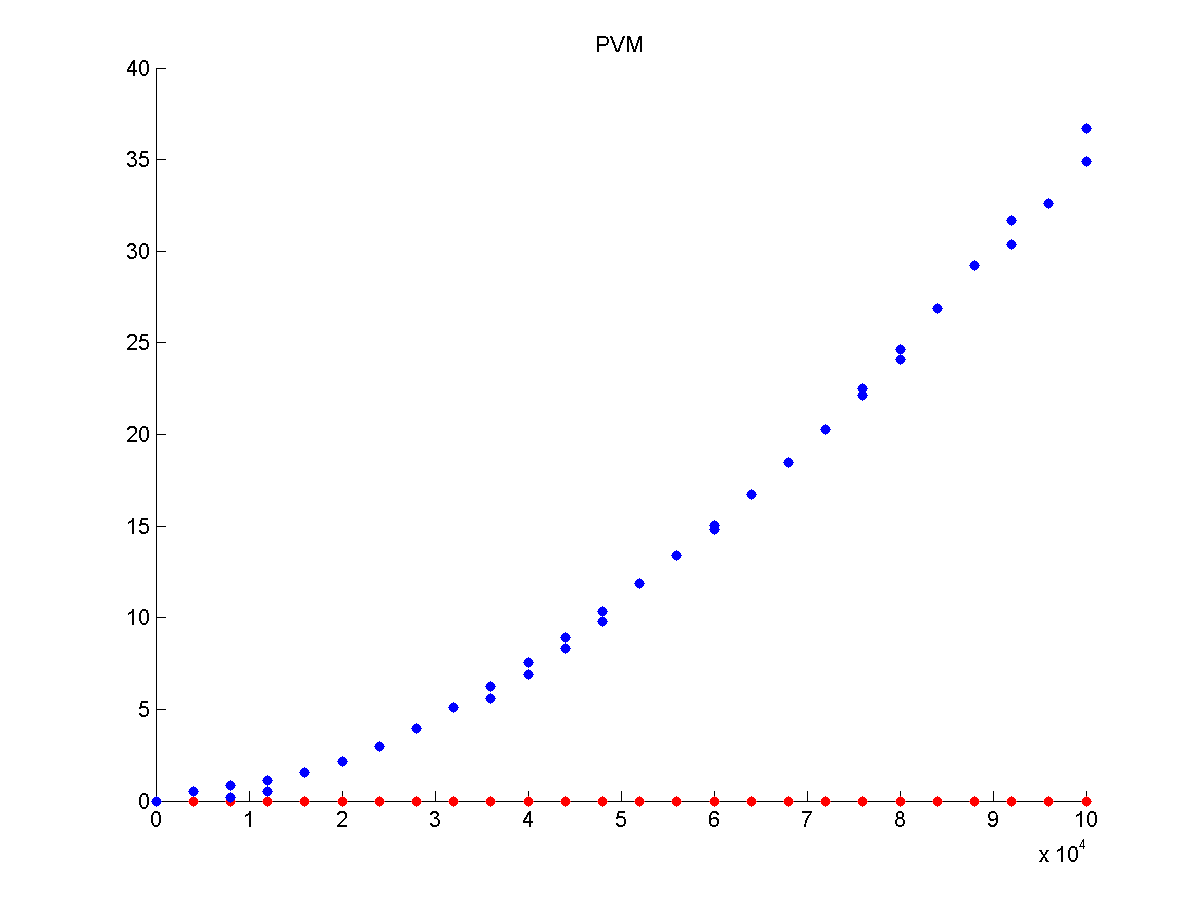

Supplement: Supplementary file 2 [file Presentation2.ZIP › PVM.png]

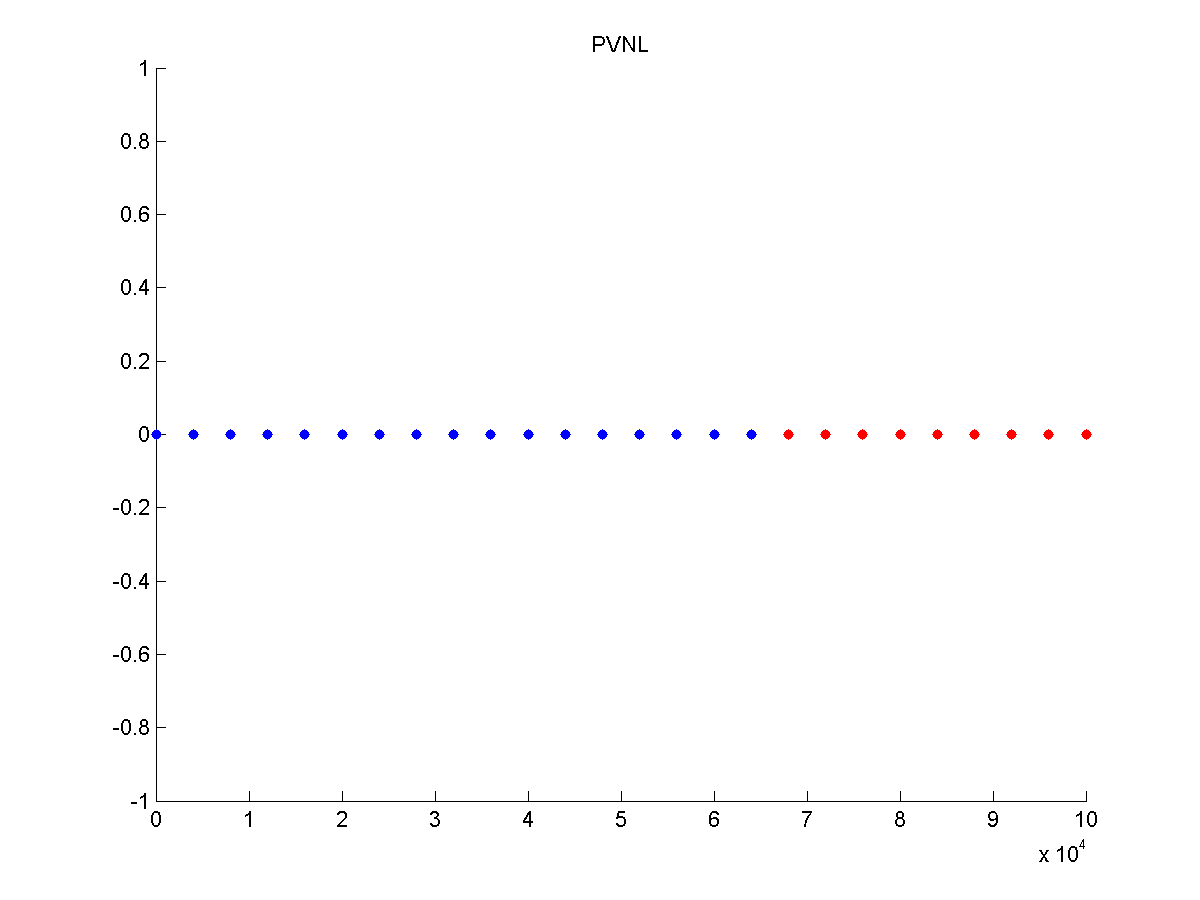

Supplement: Supplementary file 2 [file Presentation2.ZIP › PVNL.png]

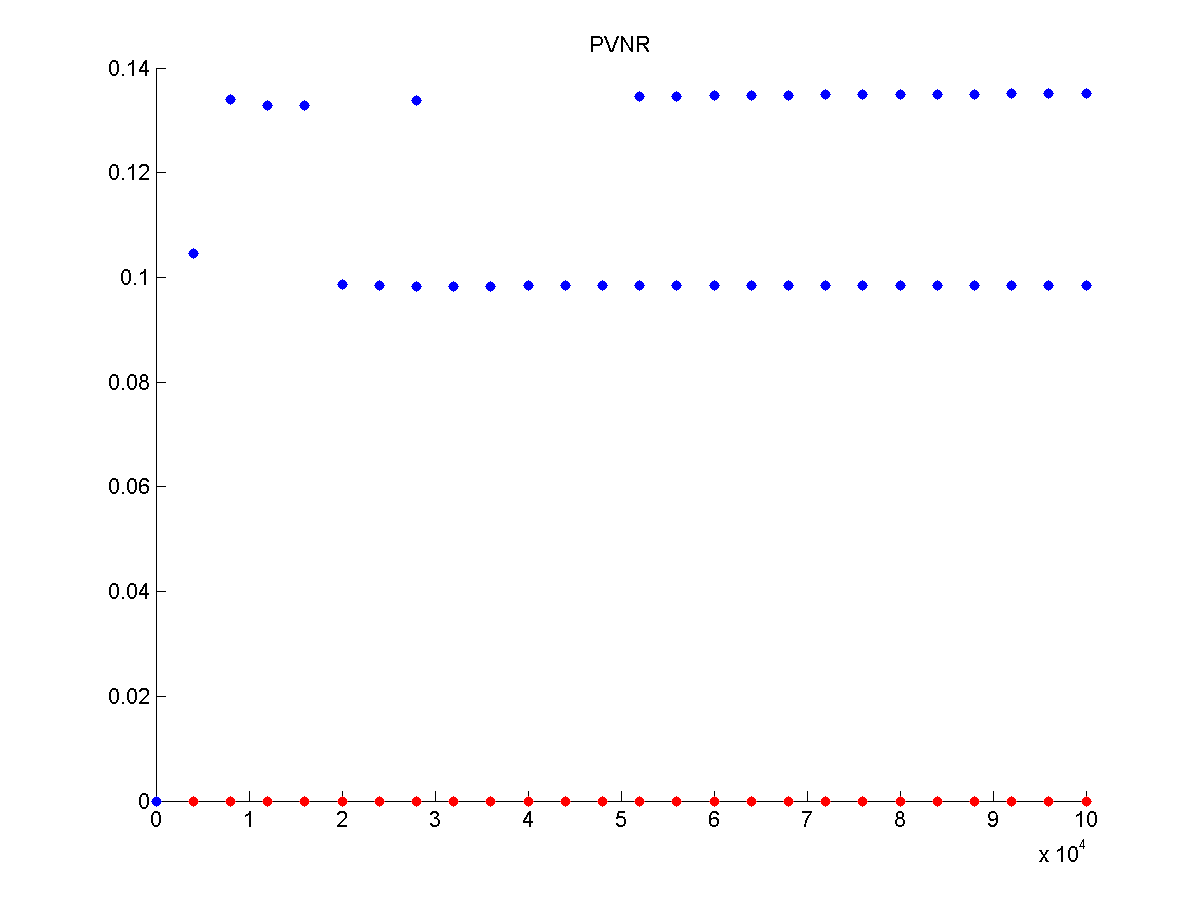

Supplement: Supplementary file 2 [file Presentation2.ZIP › PVNR.png]

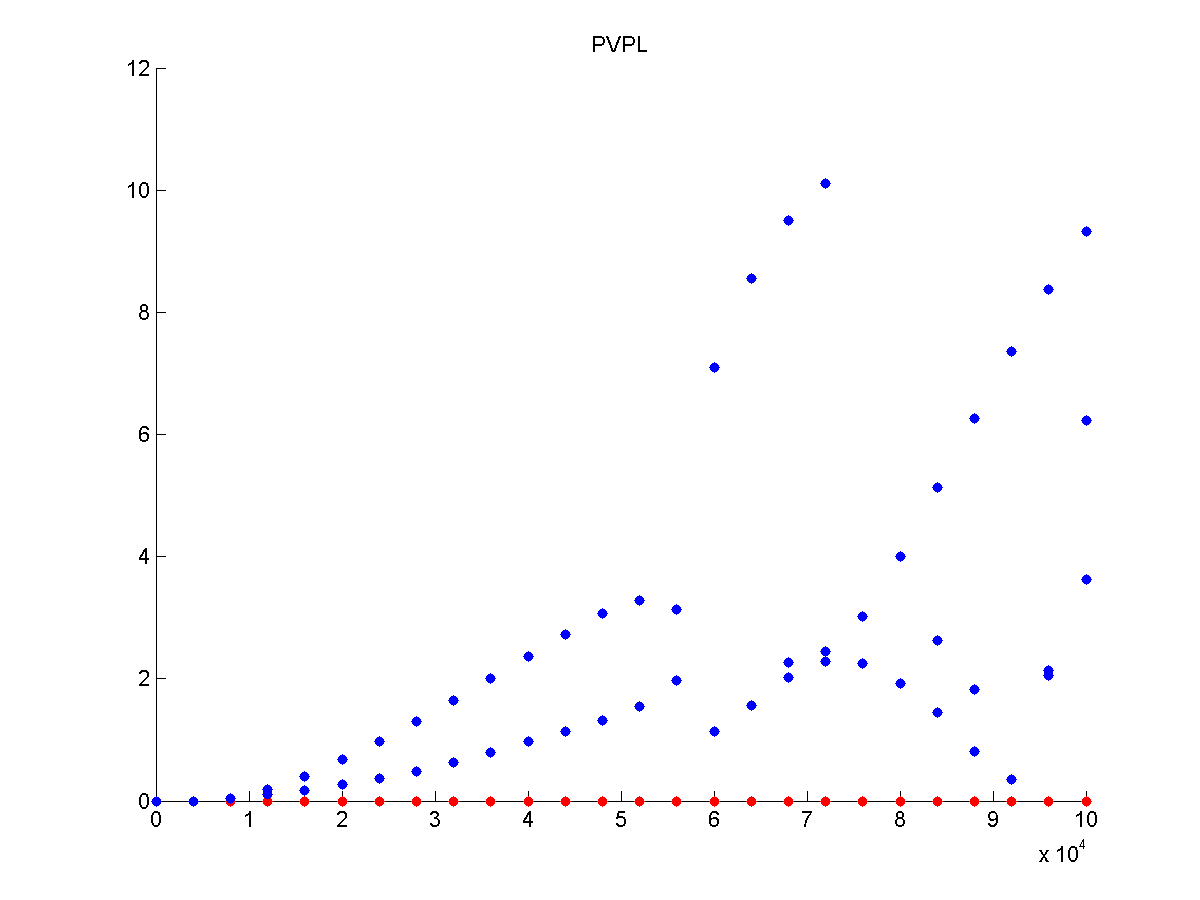

Supplement: Supplementary file 2 [file Presentation2.ZIP › PVPL.png]

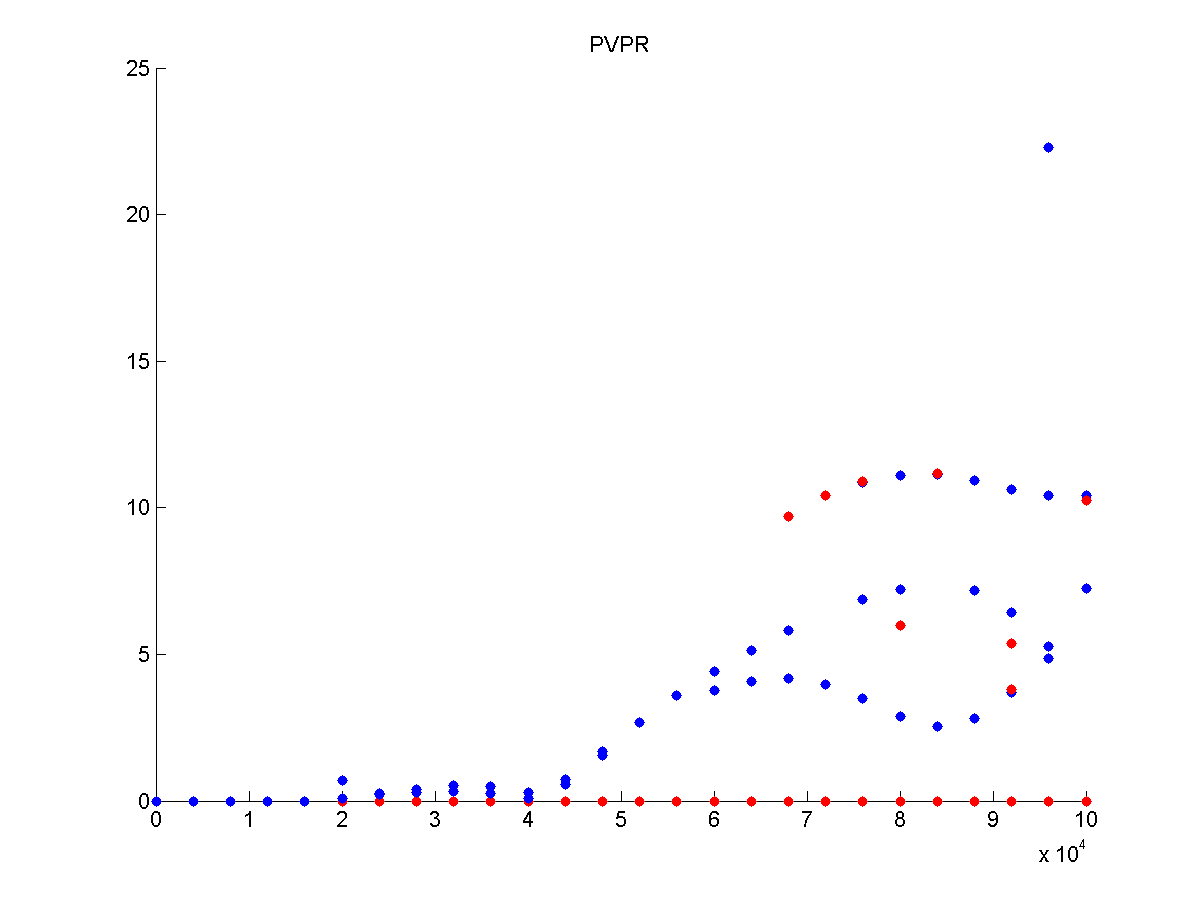

Supplement: Supplementary file 2 [file Presentation2.ZIP › PVPR.png]

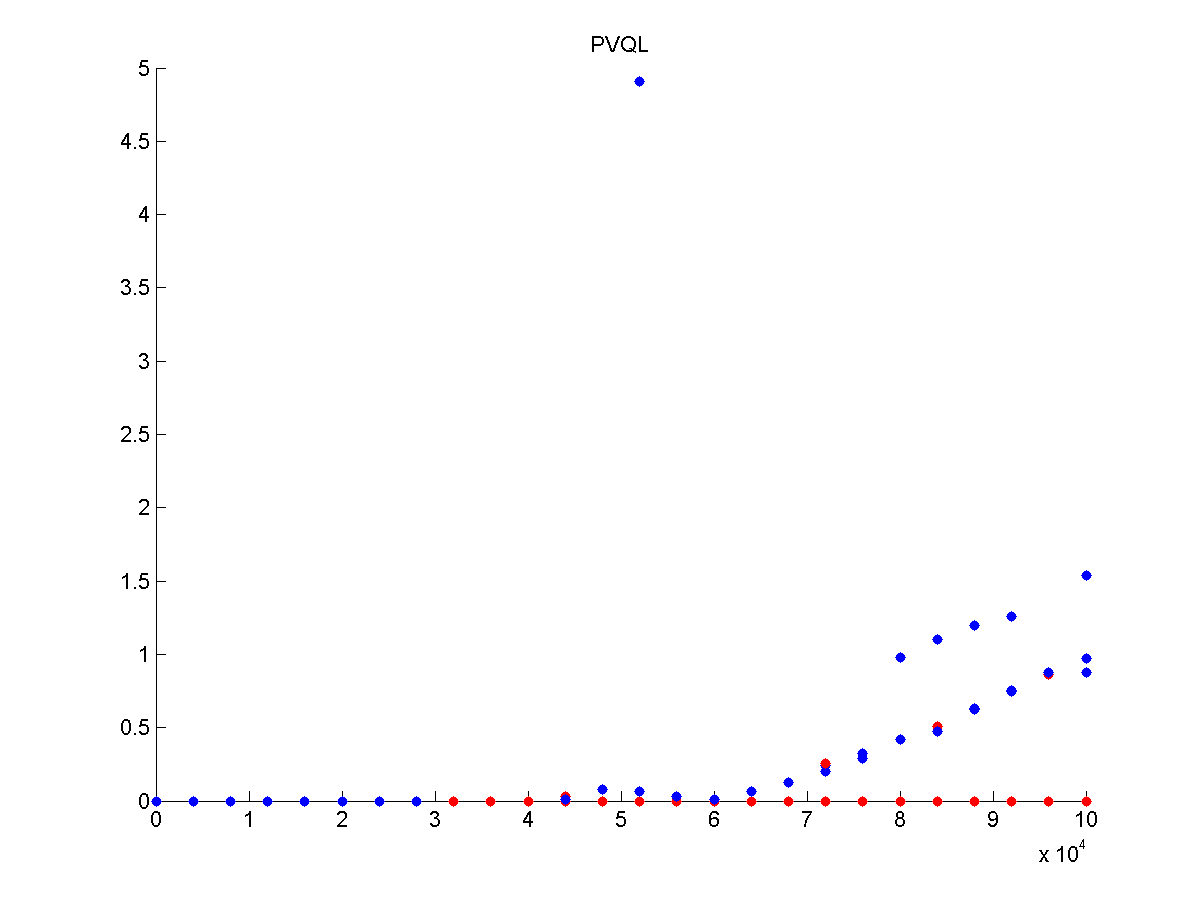

Supplement: Supplementary file 2 [file Presentation2.ZIP › PVQL.png]

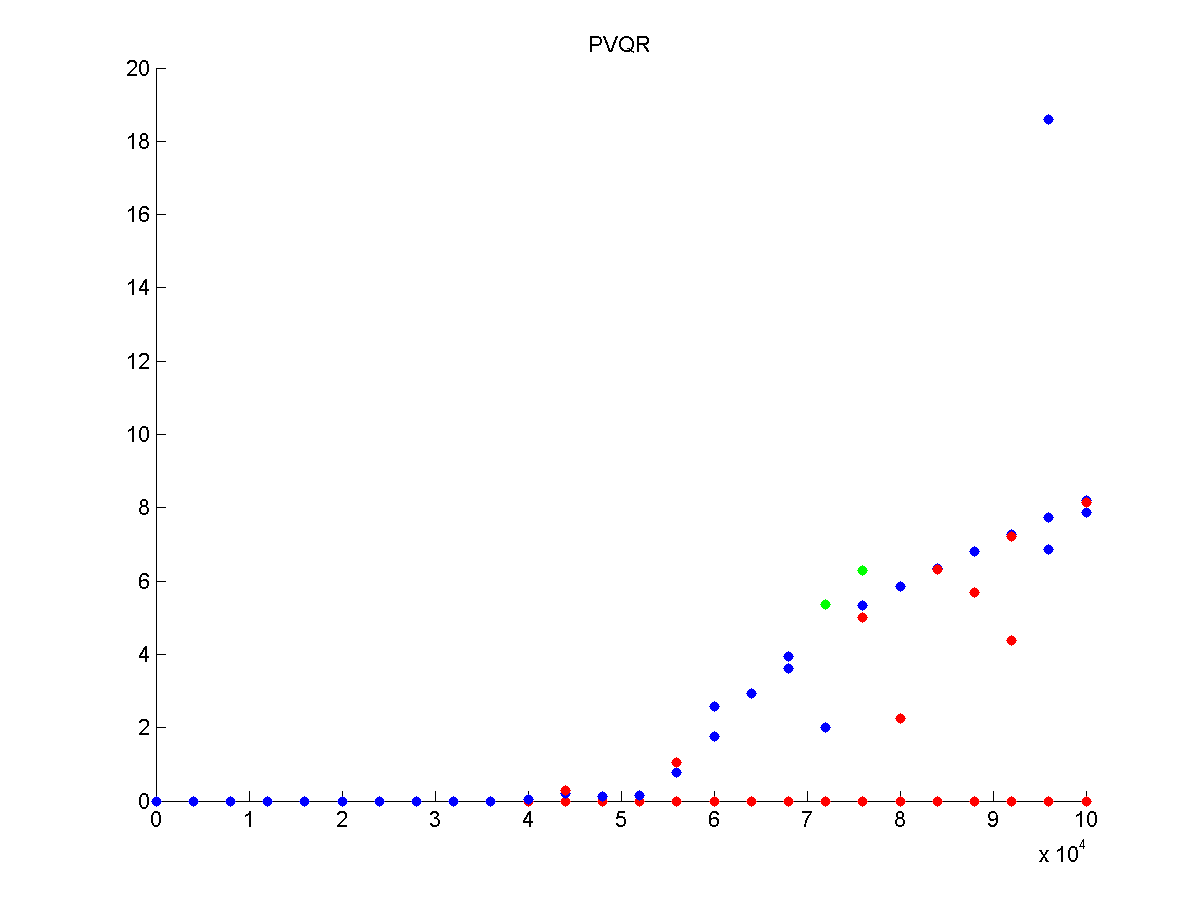

Supplement: Supplementary file 2 [file Presentation2.ZIP › PVQR.png]

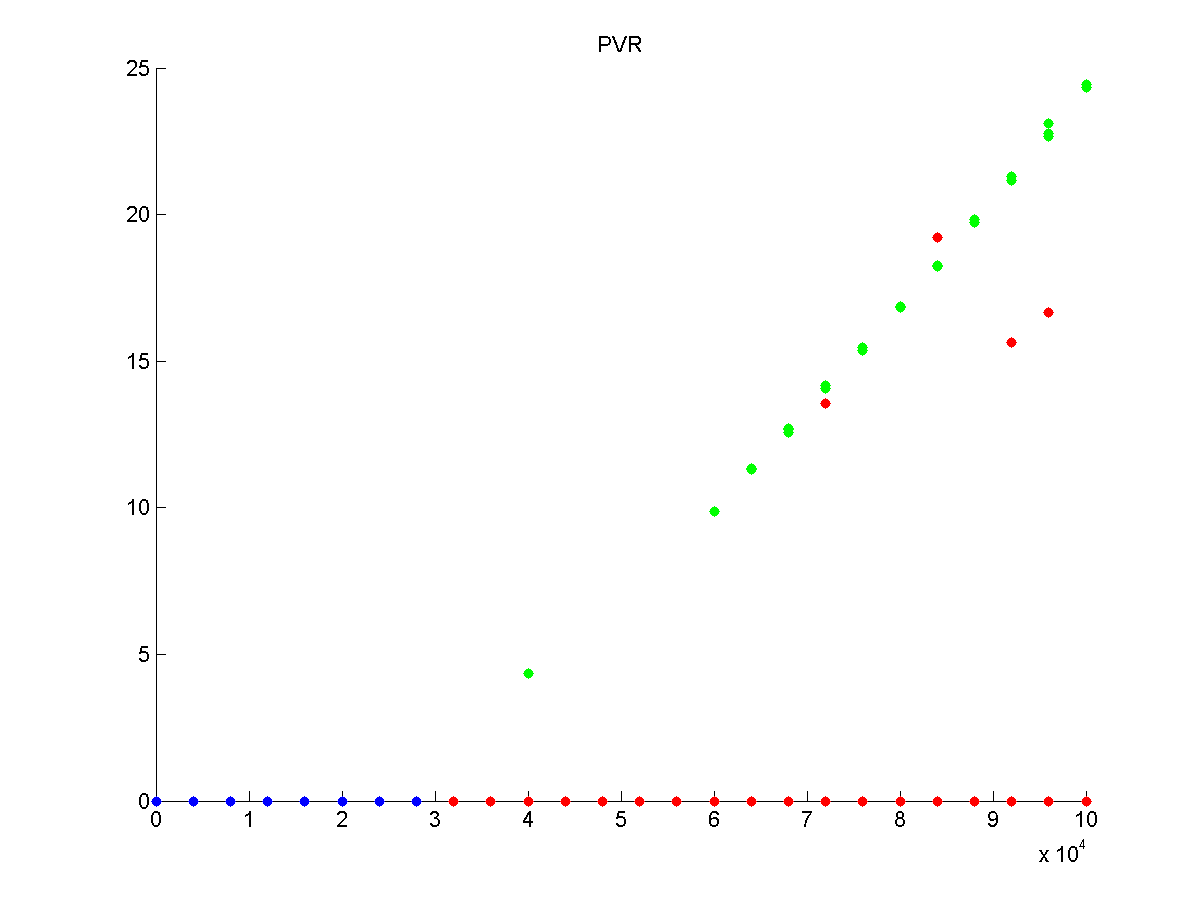

Supplement: Supplementary file 2 [file Presentation2.ZIP › PVR.png]

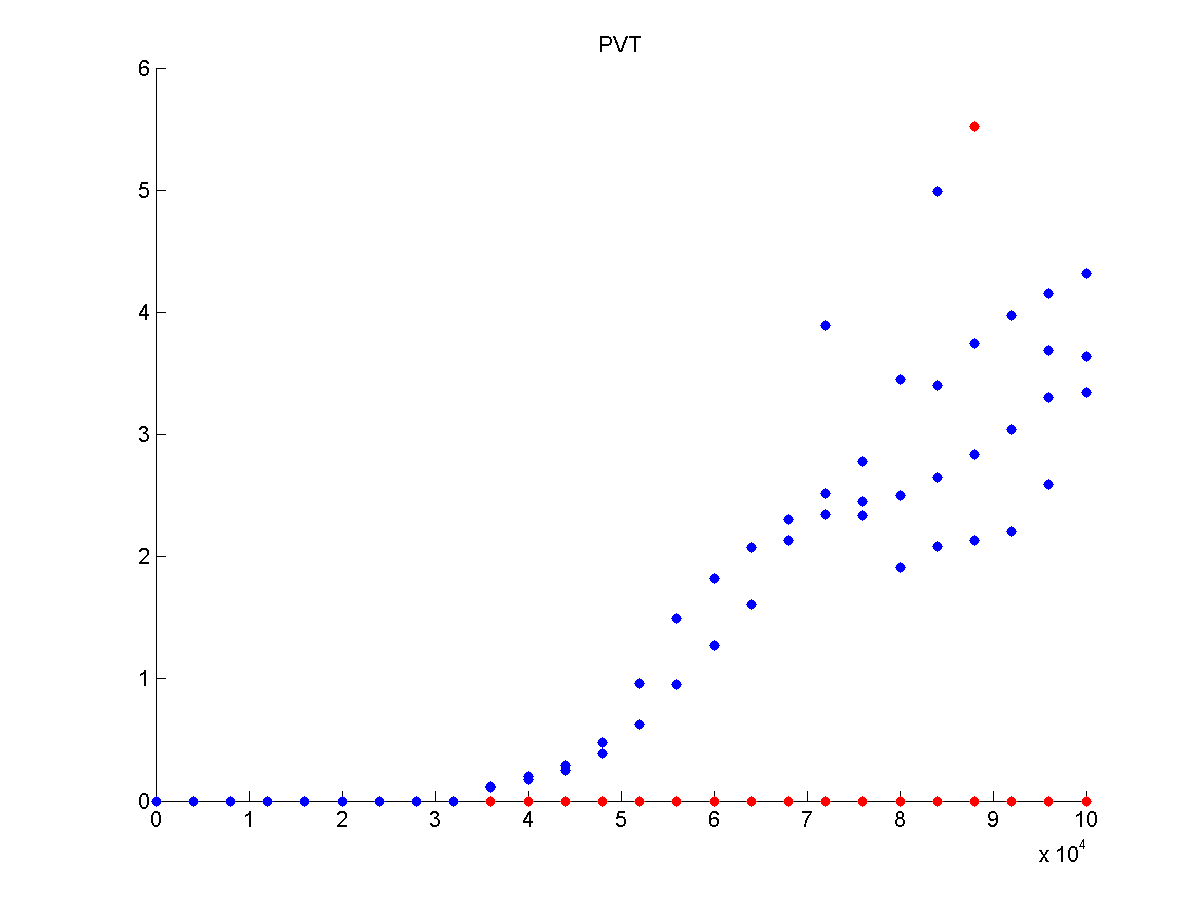

Supplement: Supplementary file 2 [file Presentation2.ZIP › PVT.png]

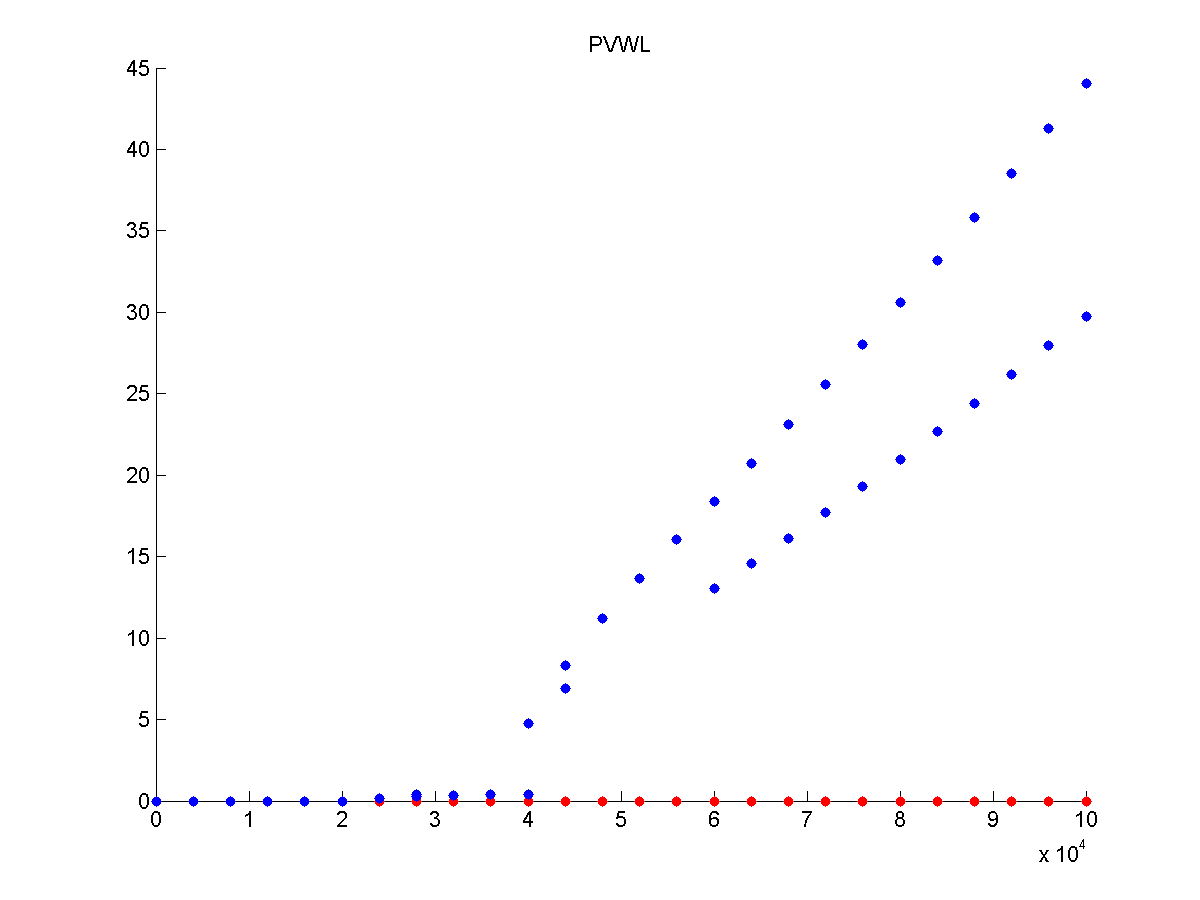

Supplement: Supplementary file 2 [file Presentation2.ZIP › PVWL.png]

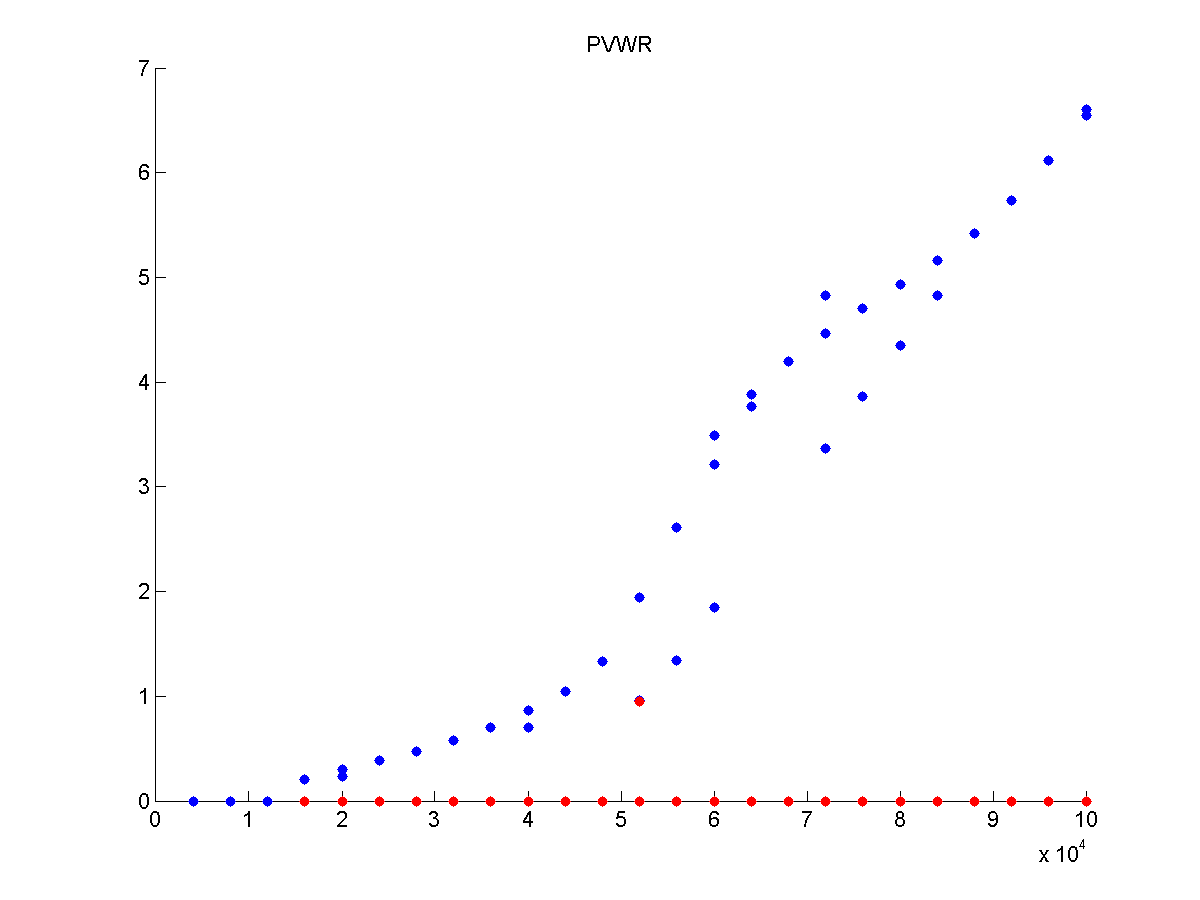

Supplement: Supplementary file 2 [file Presentation2.ZIP › PVWR.png]

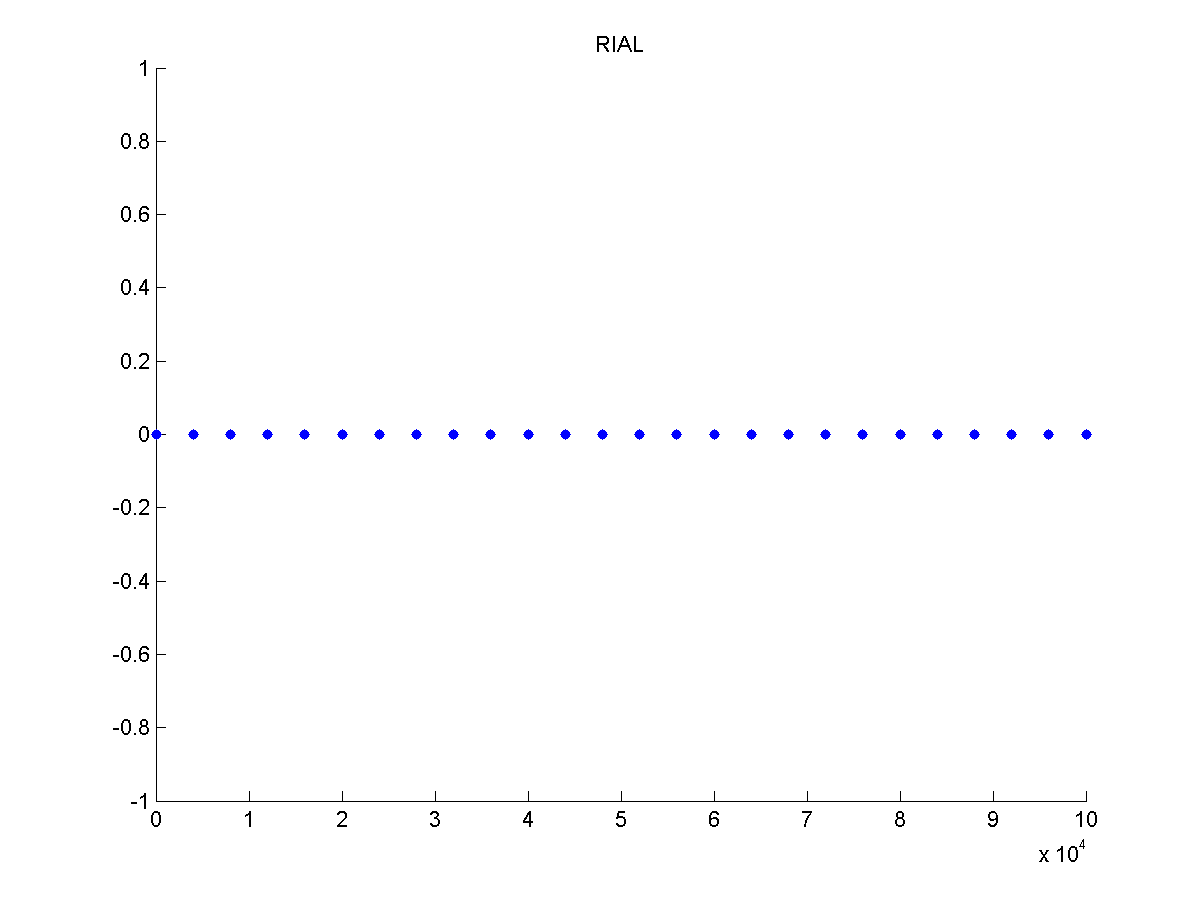

Supplement: Supplementary file 2 [file Presentation2.ZIP › RIAL.png]

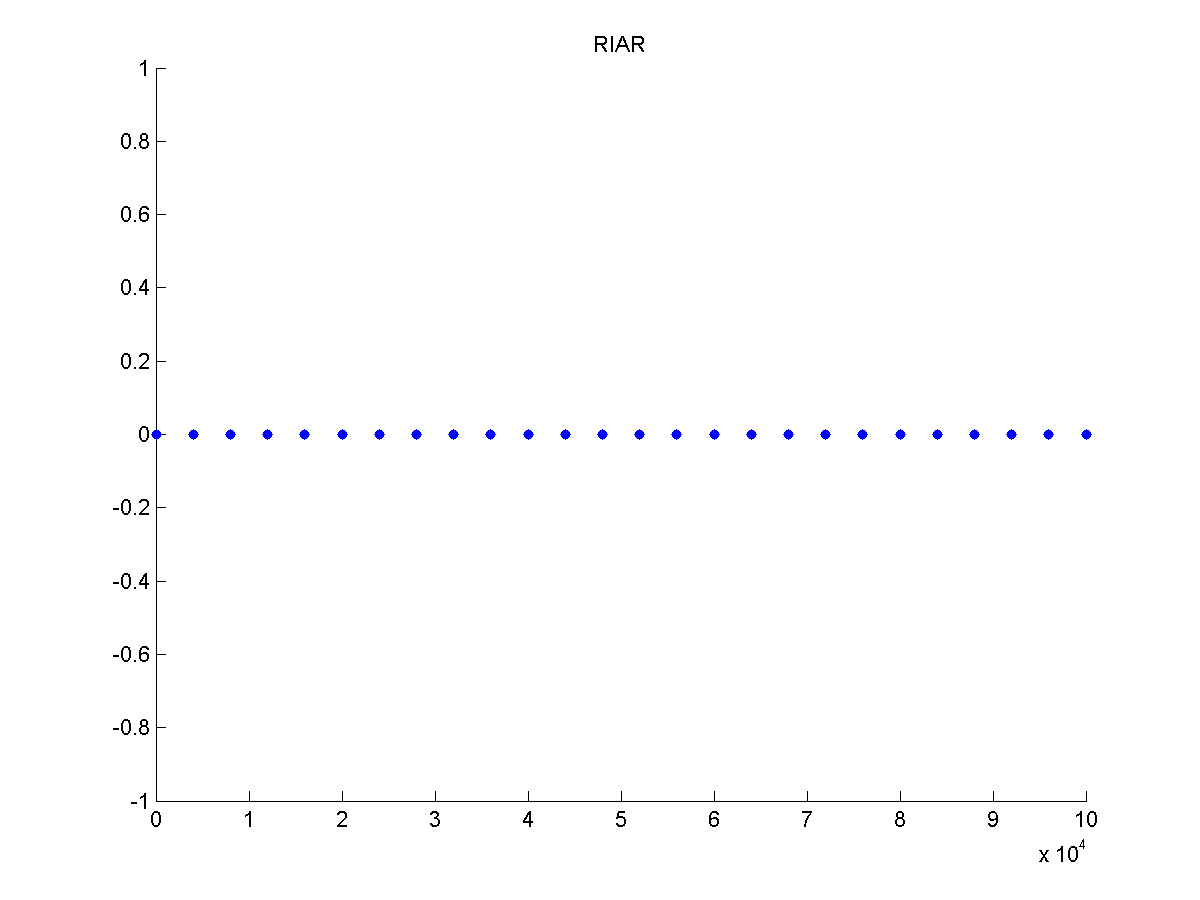

Supplement: Supplementary file 2 [file Presentation2.ZIP › RIAR.png]

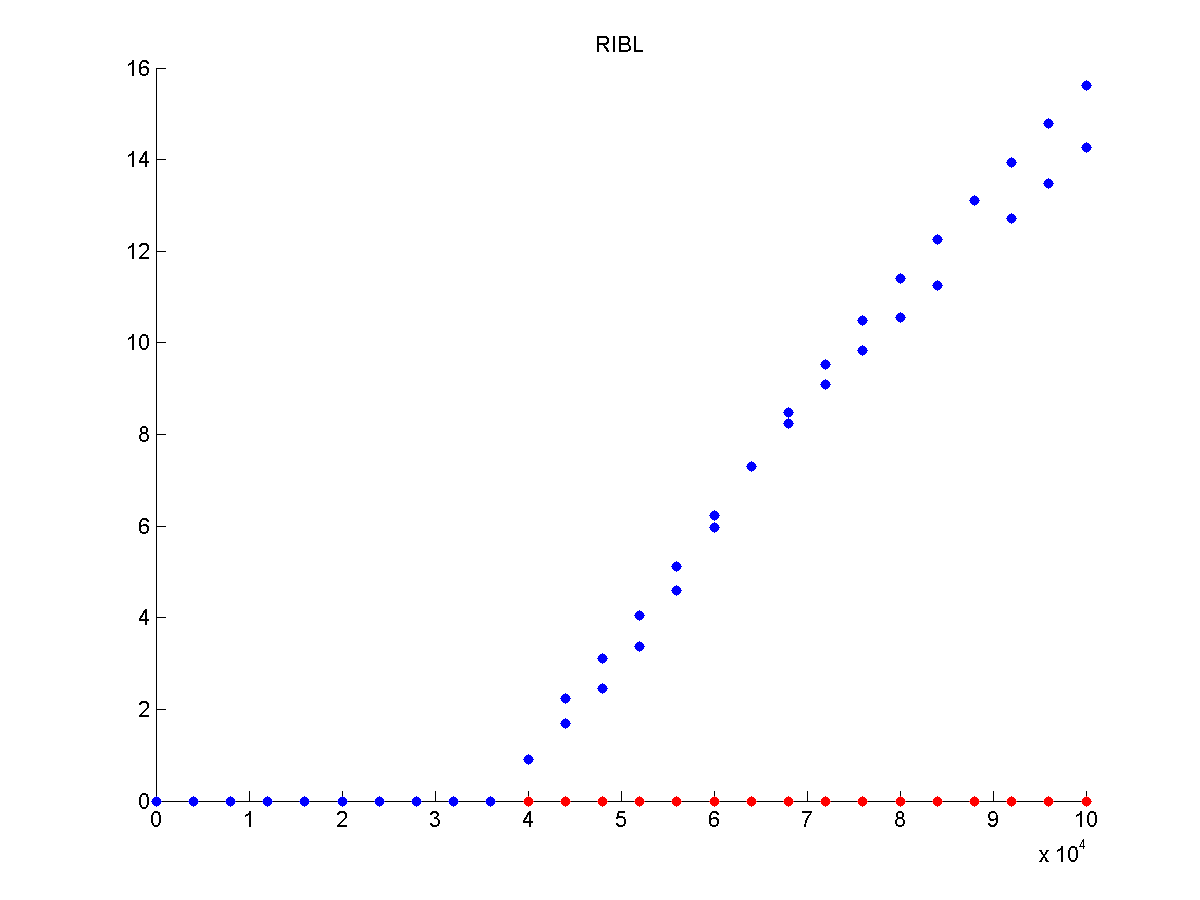

Supplement: Supplementary file 2 [file Presentation2.ZIP › RIBL.png]

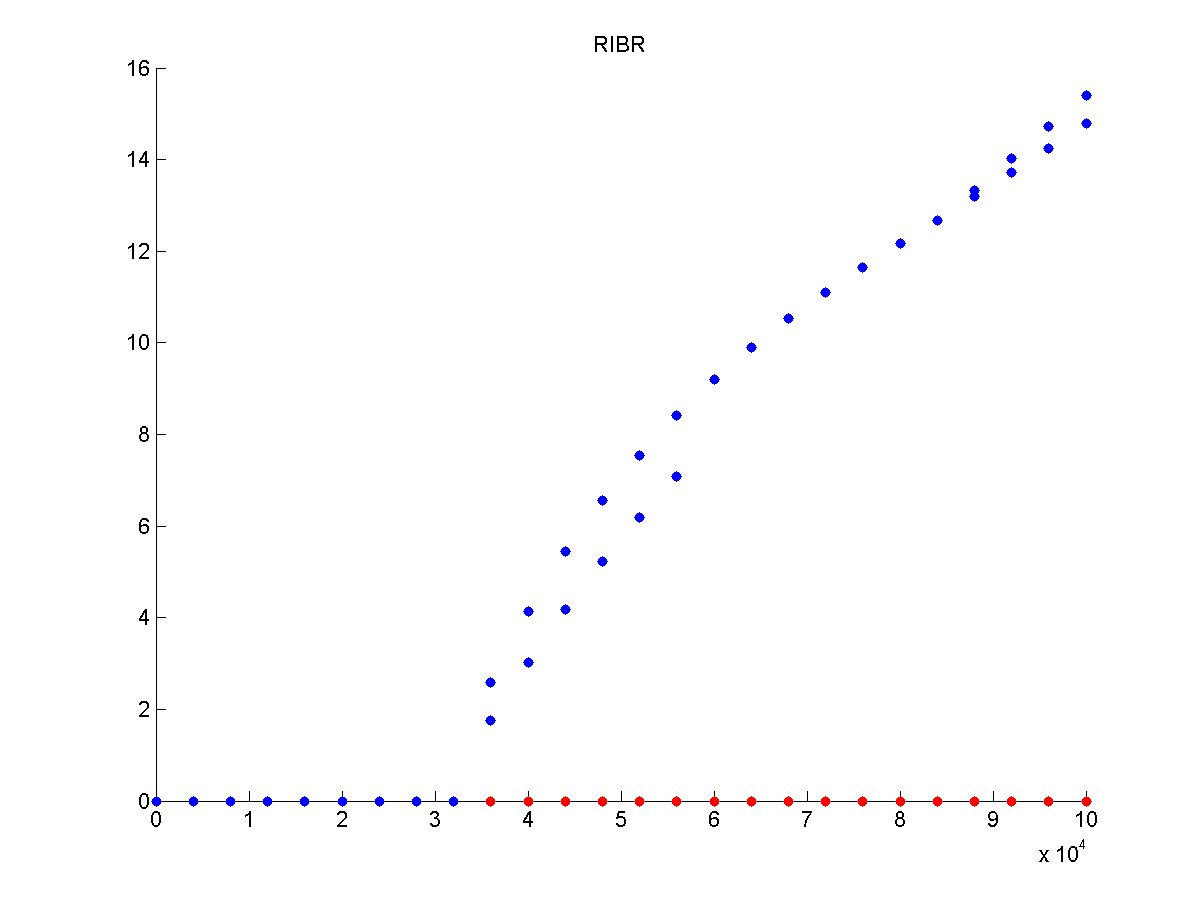

Supplement: Supplementary file 2 [file Presentation2.ZIP › RIBR.png]

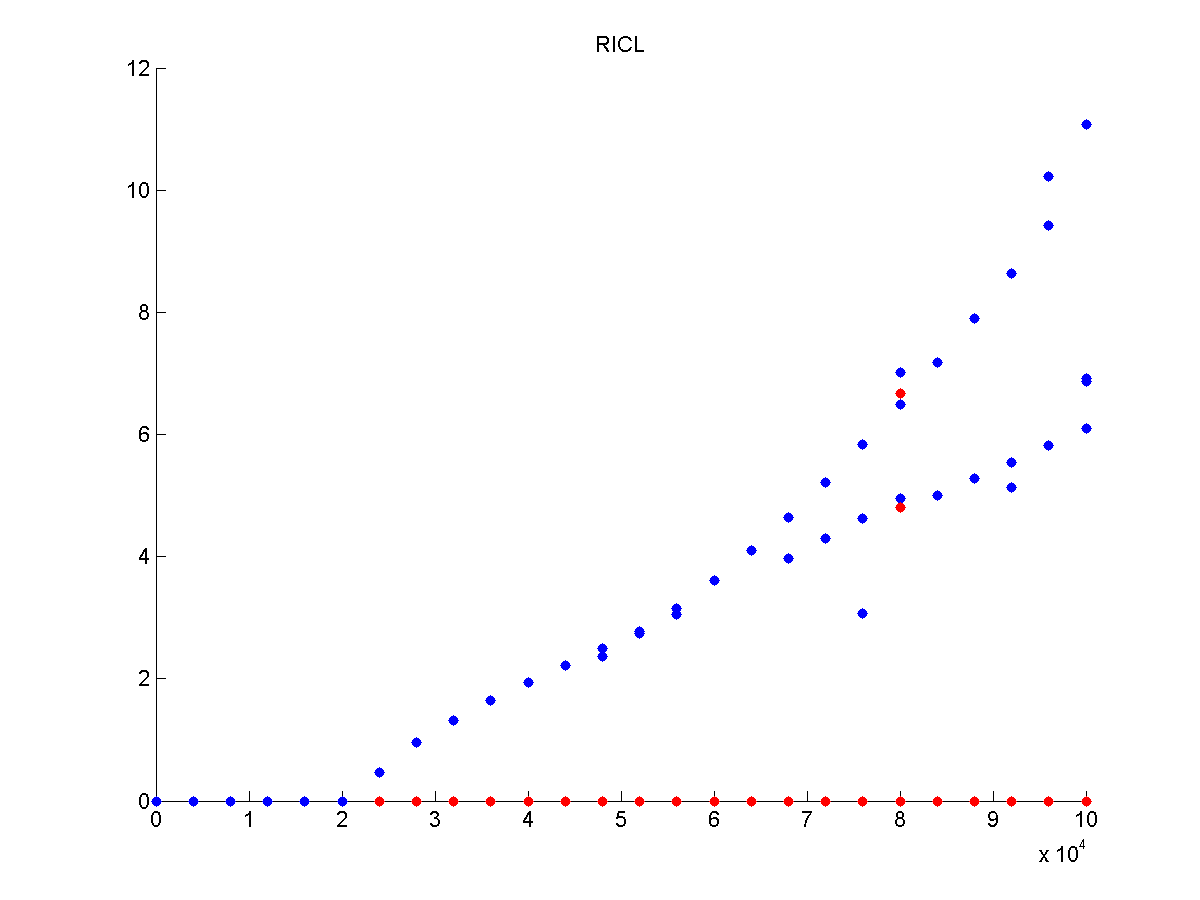

Supplement: Supplementary file 2 [file Presentation2.ZIP › RICL.png]

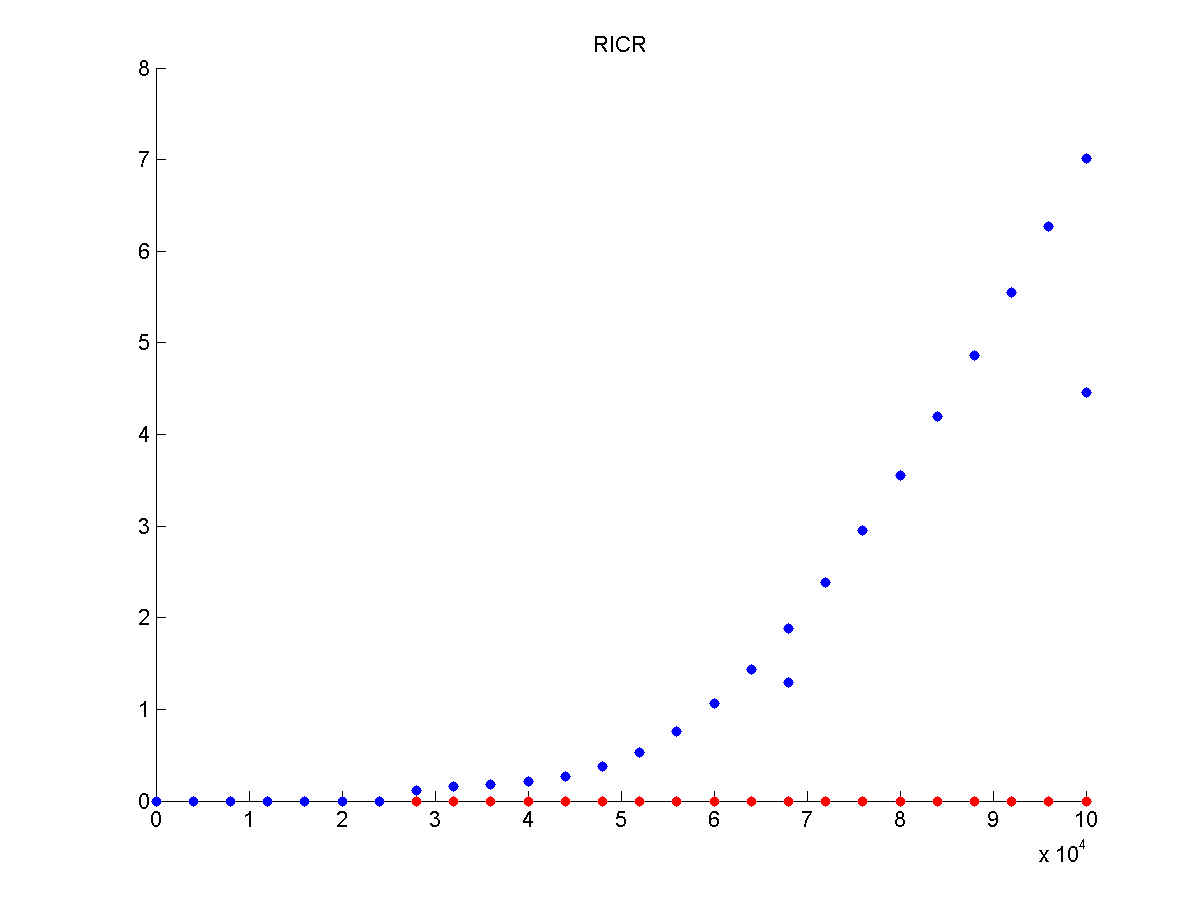

Supplement: Supplementary file 2 [file Presentation2.ZIP › RICR.png]

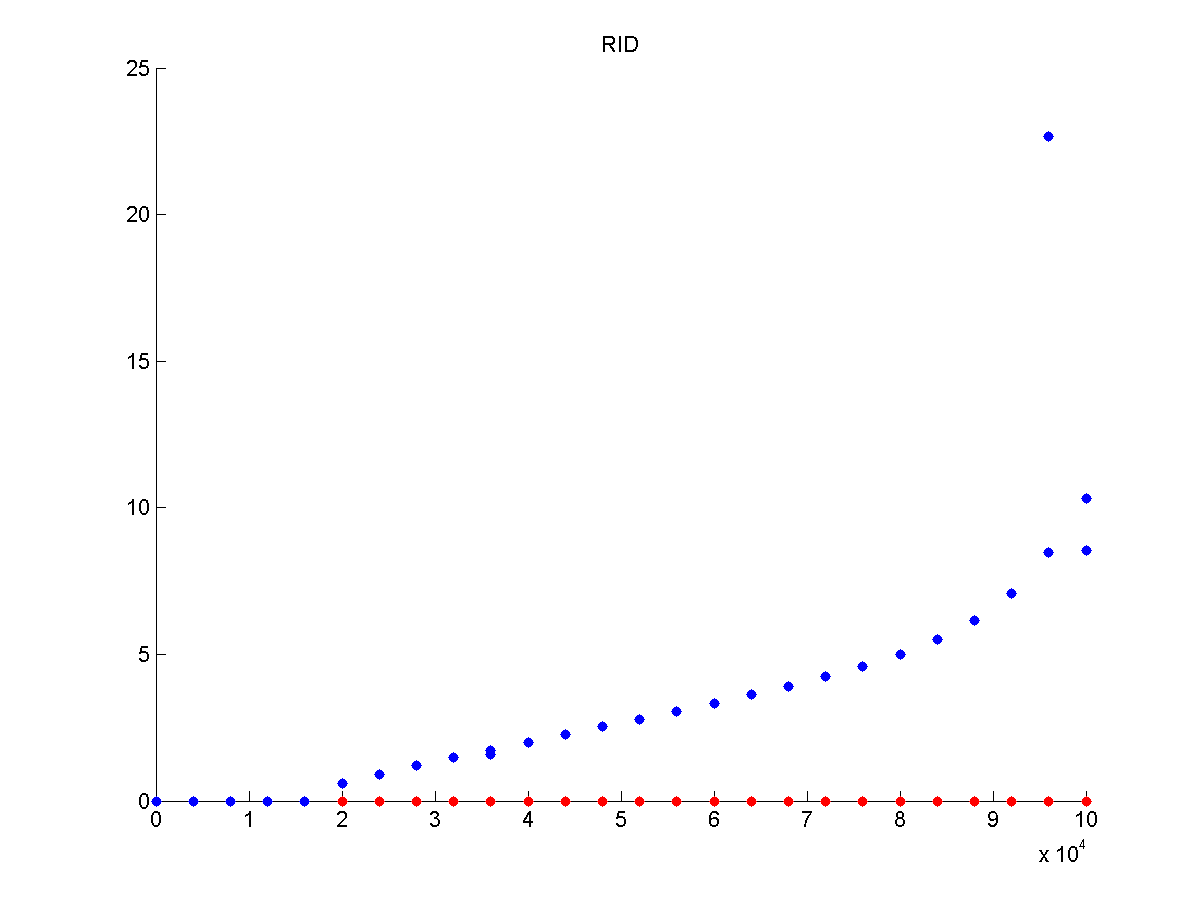

Supplement: Supplementary file 2 [file Presentation2.ZIP › RID.png]

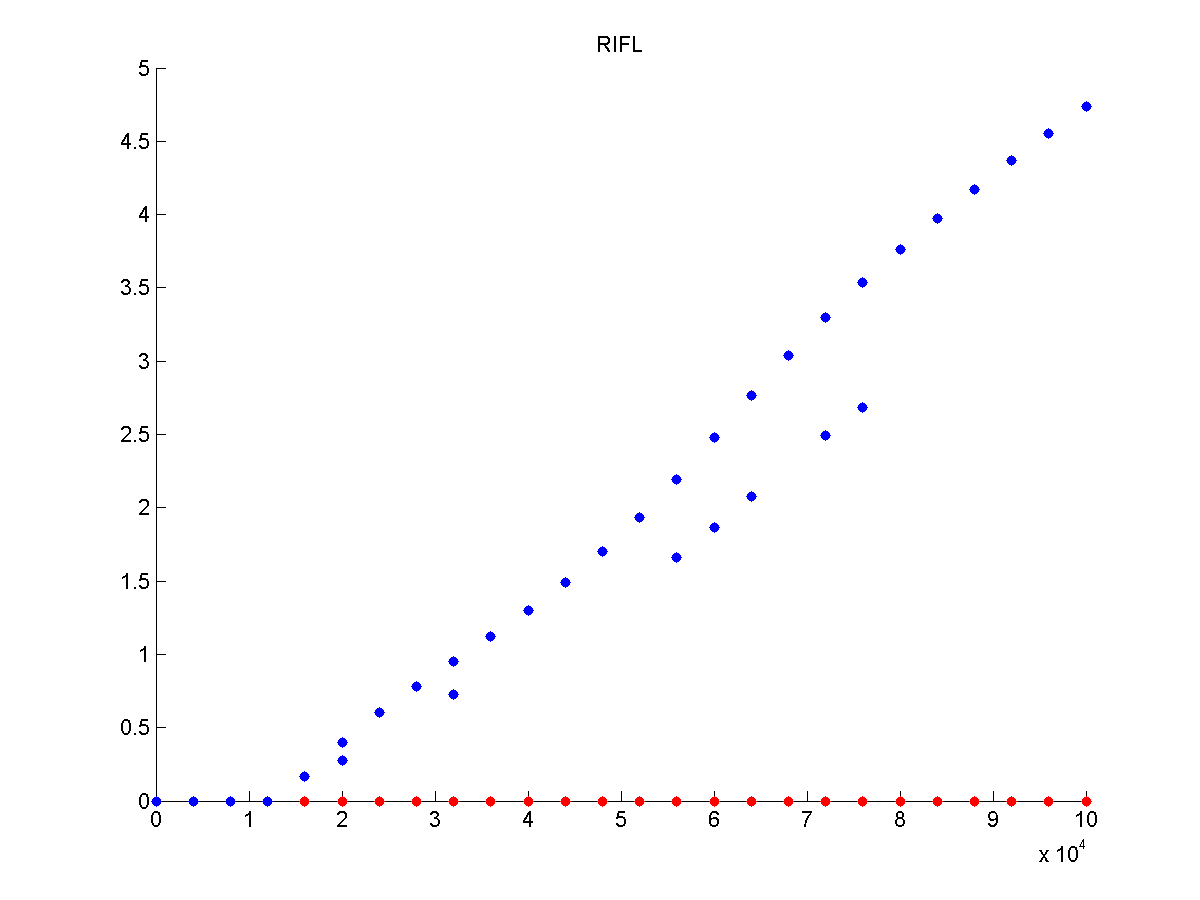

Supplement: Supplementary file 2 [file Presentation2.ZIP › RIFL.png]

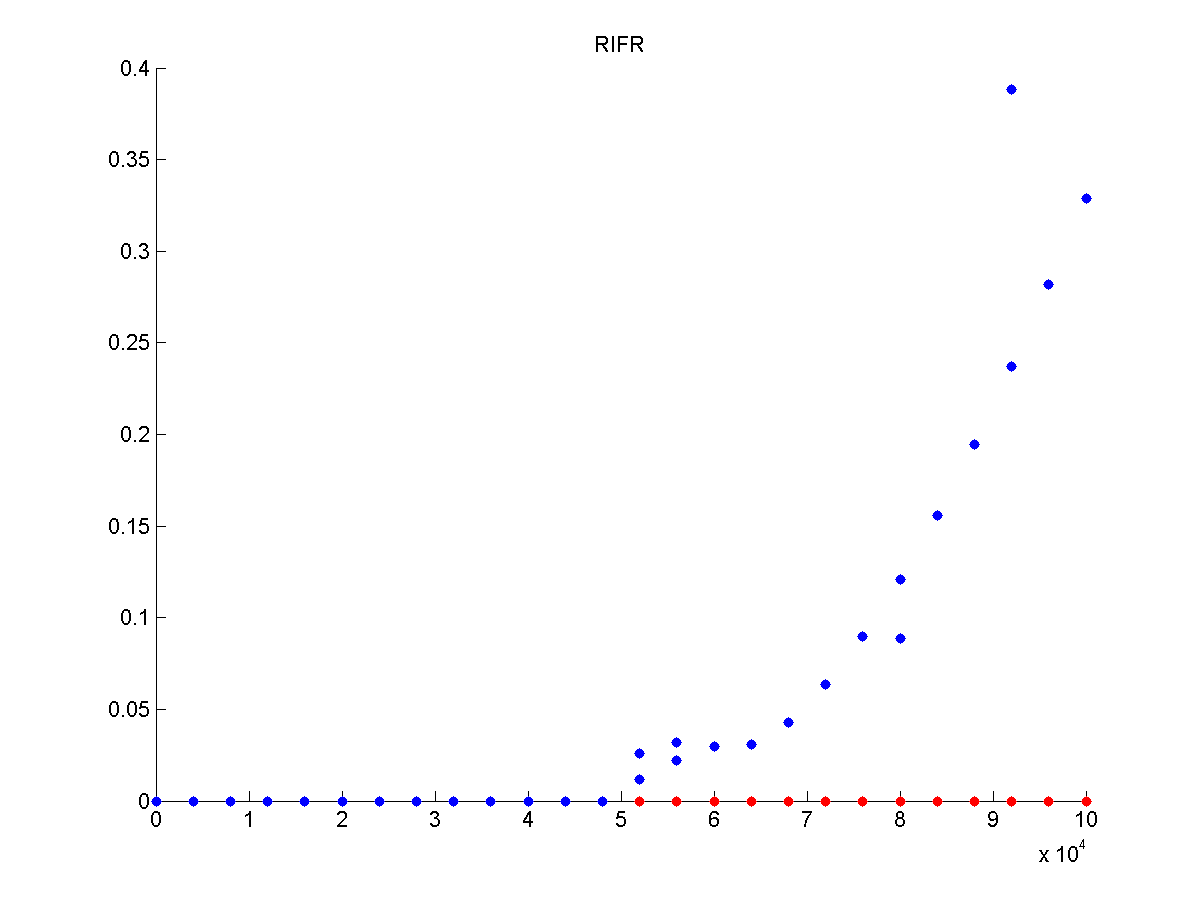

Supplement: Supplementary file 2 [file Presentation2.ZIP › RIFR.png]

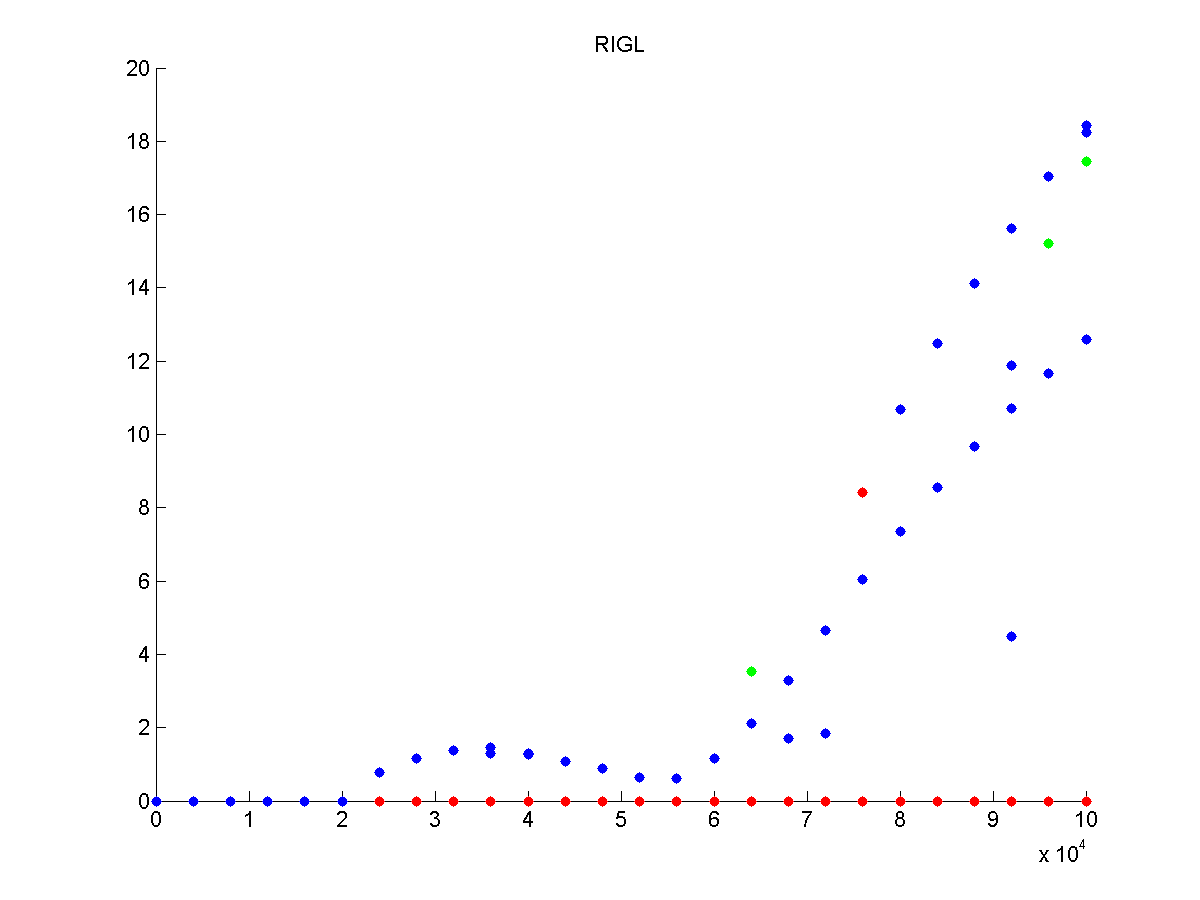

Supplement: Supplementary file 2 [file Presentation2.ZIP › RIGL.png]

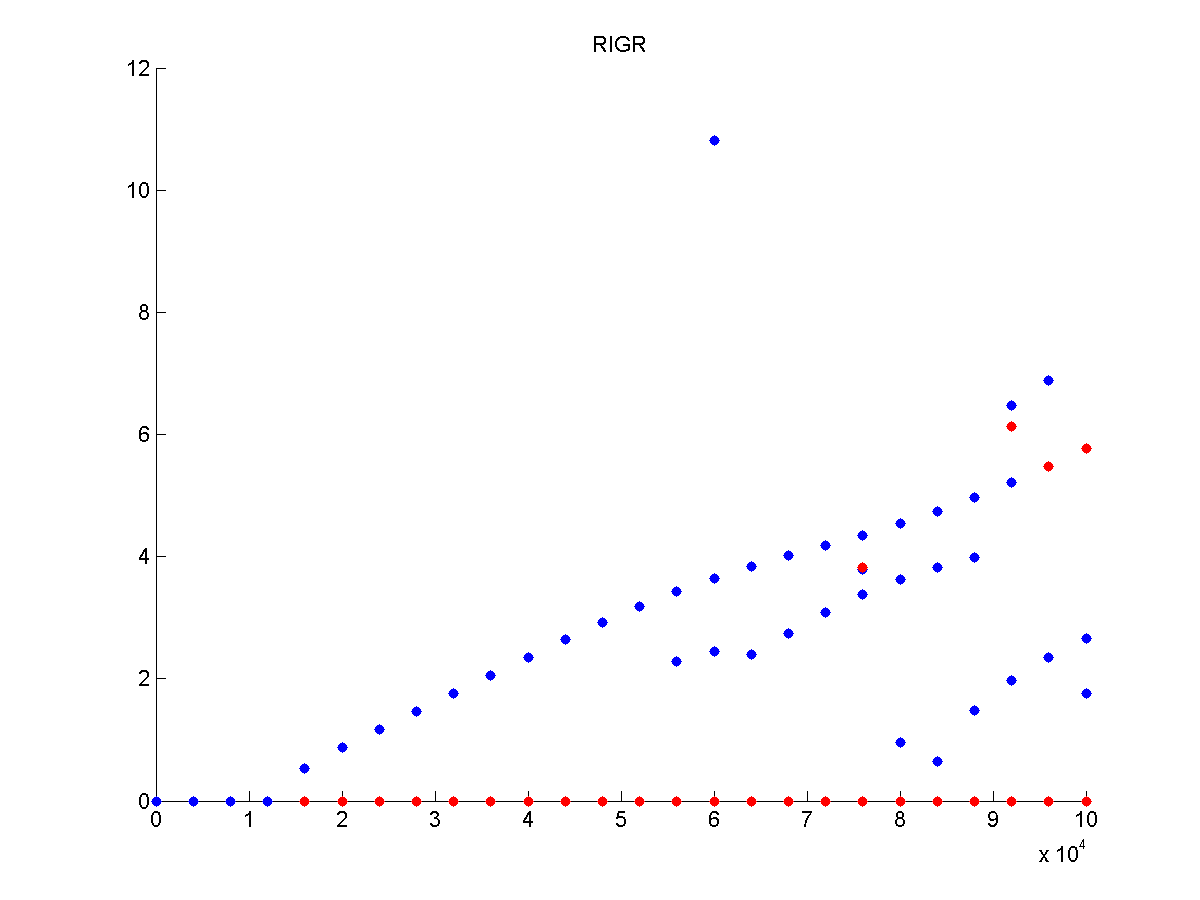

Supplement: Supplementary file 2 [file Presentation2.ZIP › RIGR.png]

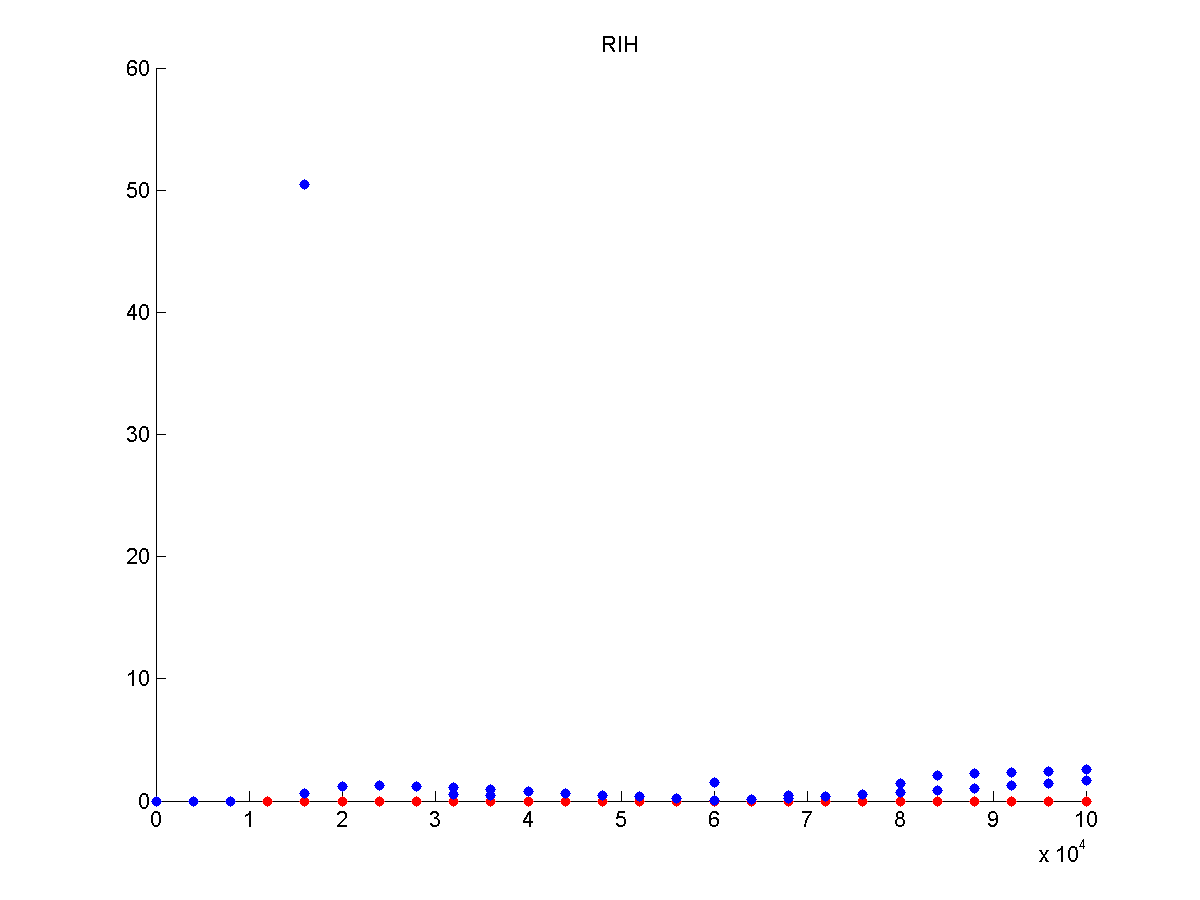

Supplement: Supplementary file 2 [file Presentation2.ZIP › RIH.png]

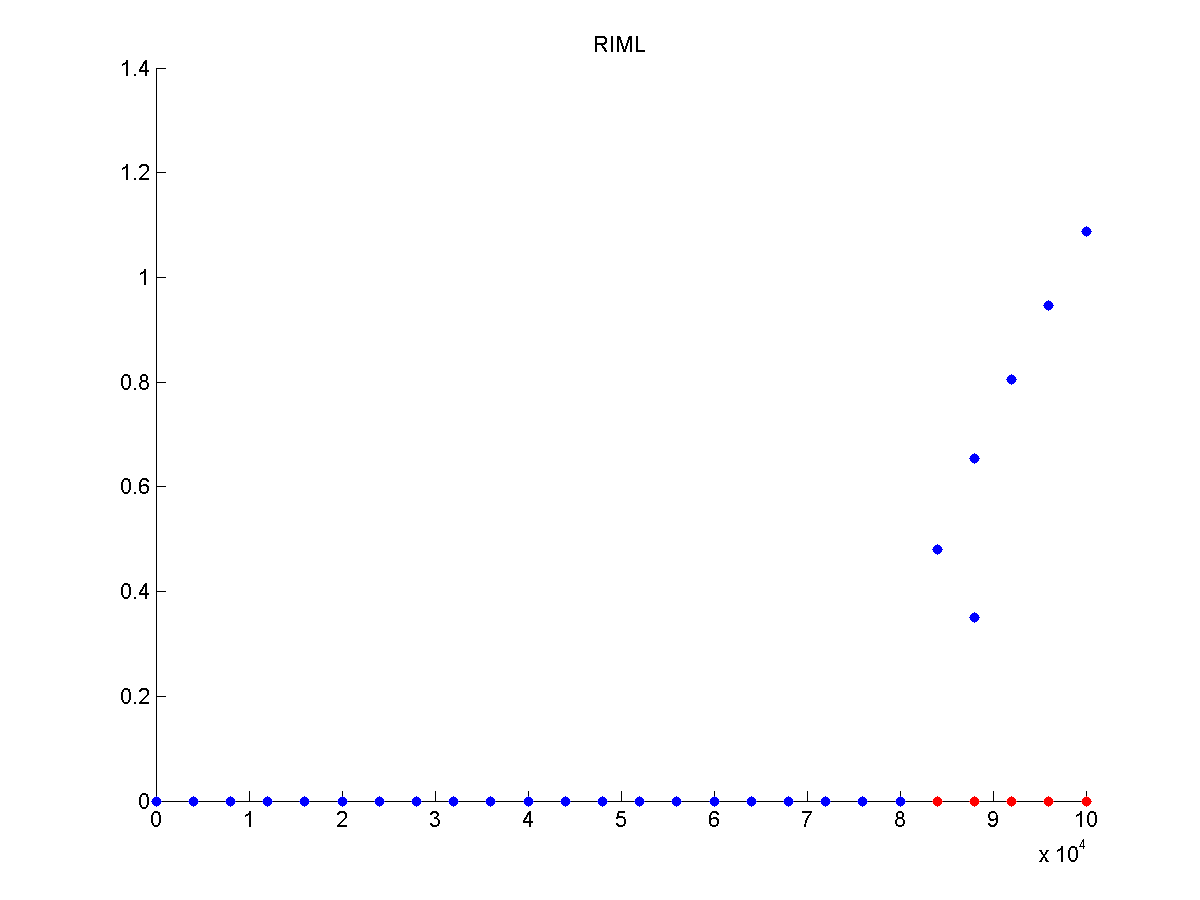

Supplement: Supplementary file 2 [file Presentation2.ZIP › RIML.png]

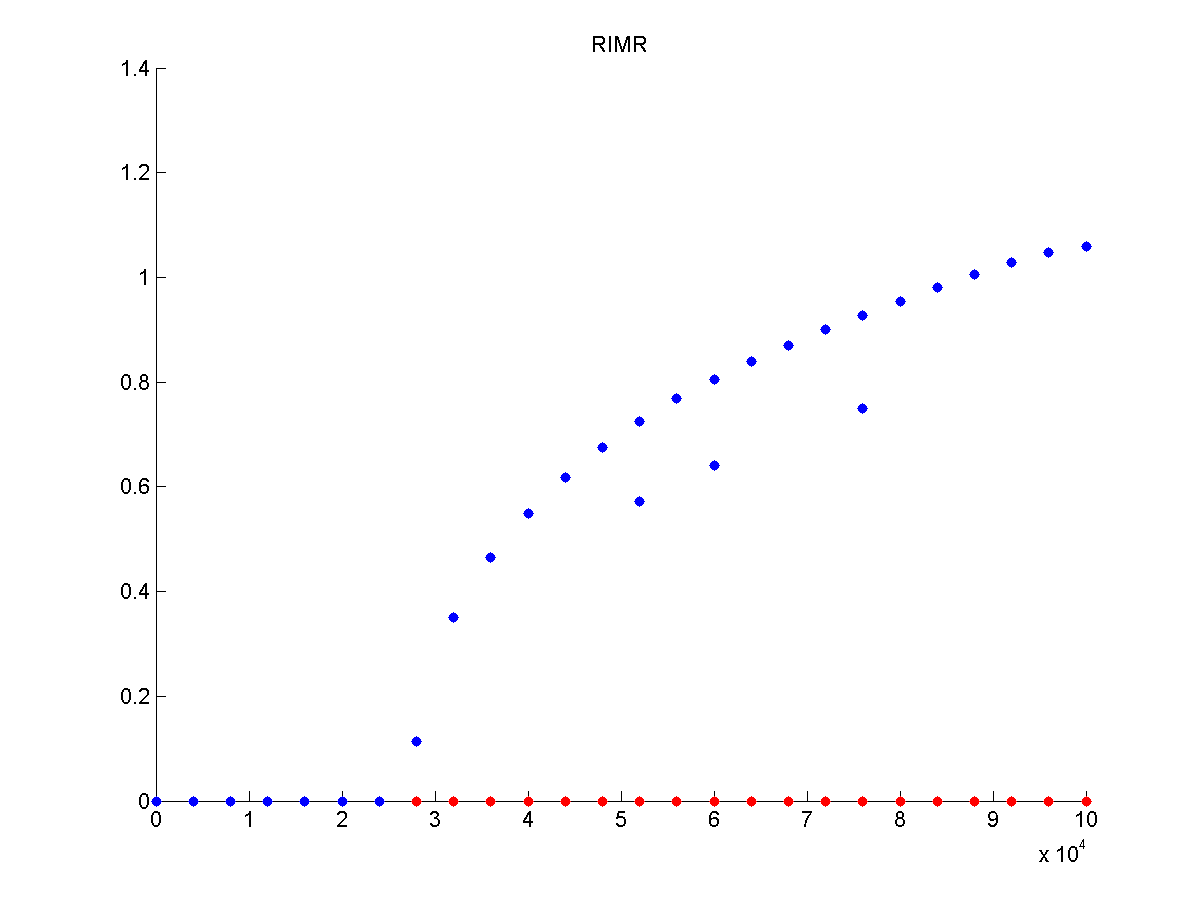

Supplement: Supplementary file 2 [file Presentation2.ZIP › RIMR.png]

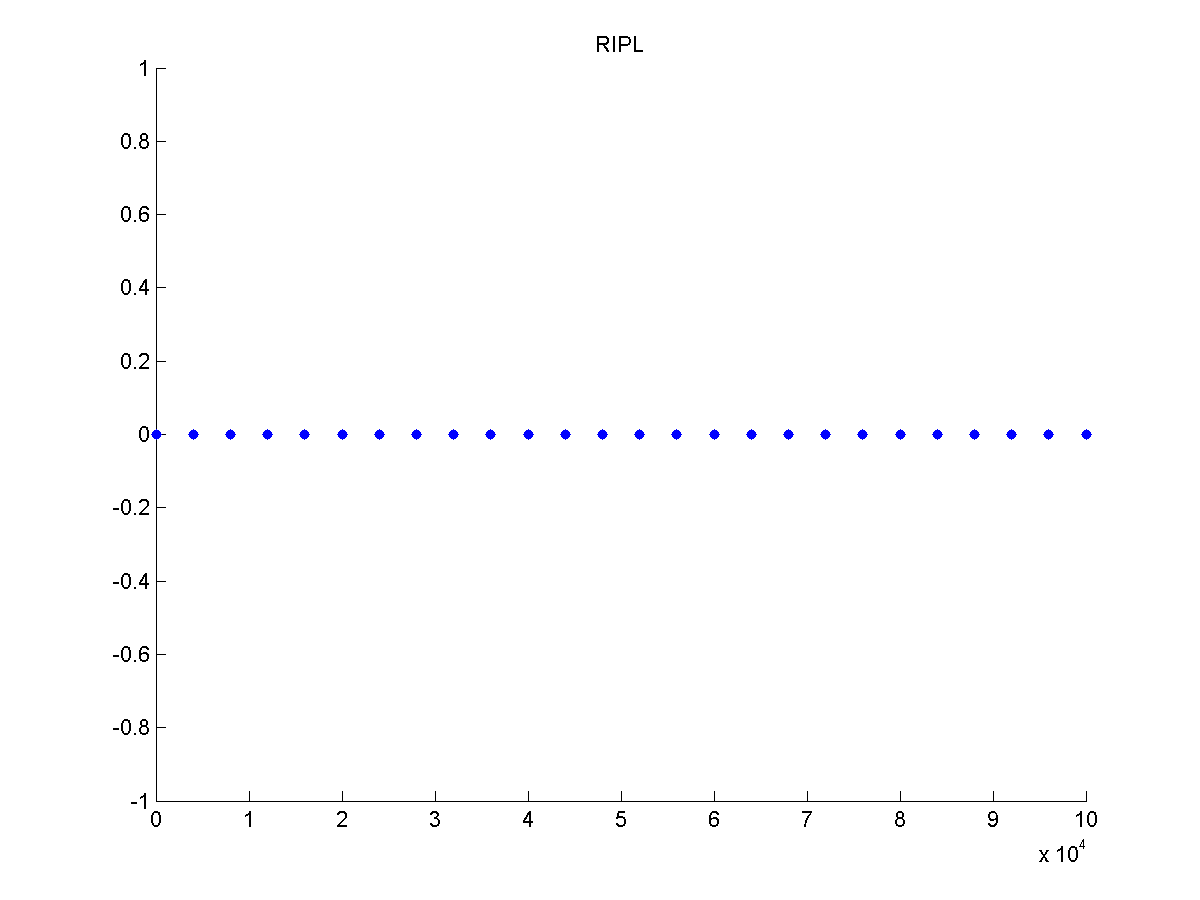

Supplement: Supplementary file 2 [file Presentation2.ZIP › RIPL.png]

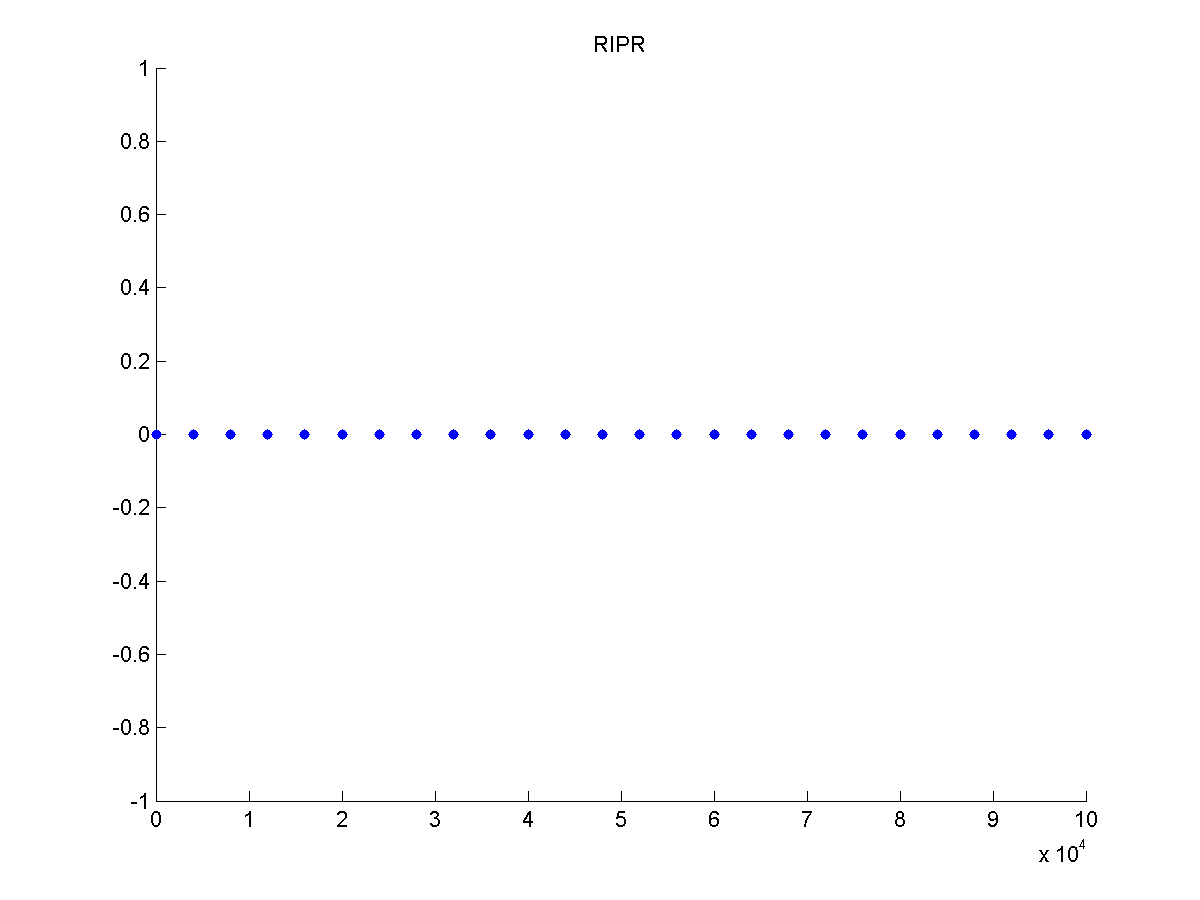

Supplement: Supplementary file 2 [file Presentation2.ZIP › RIPR.png]

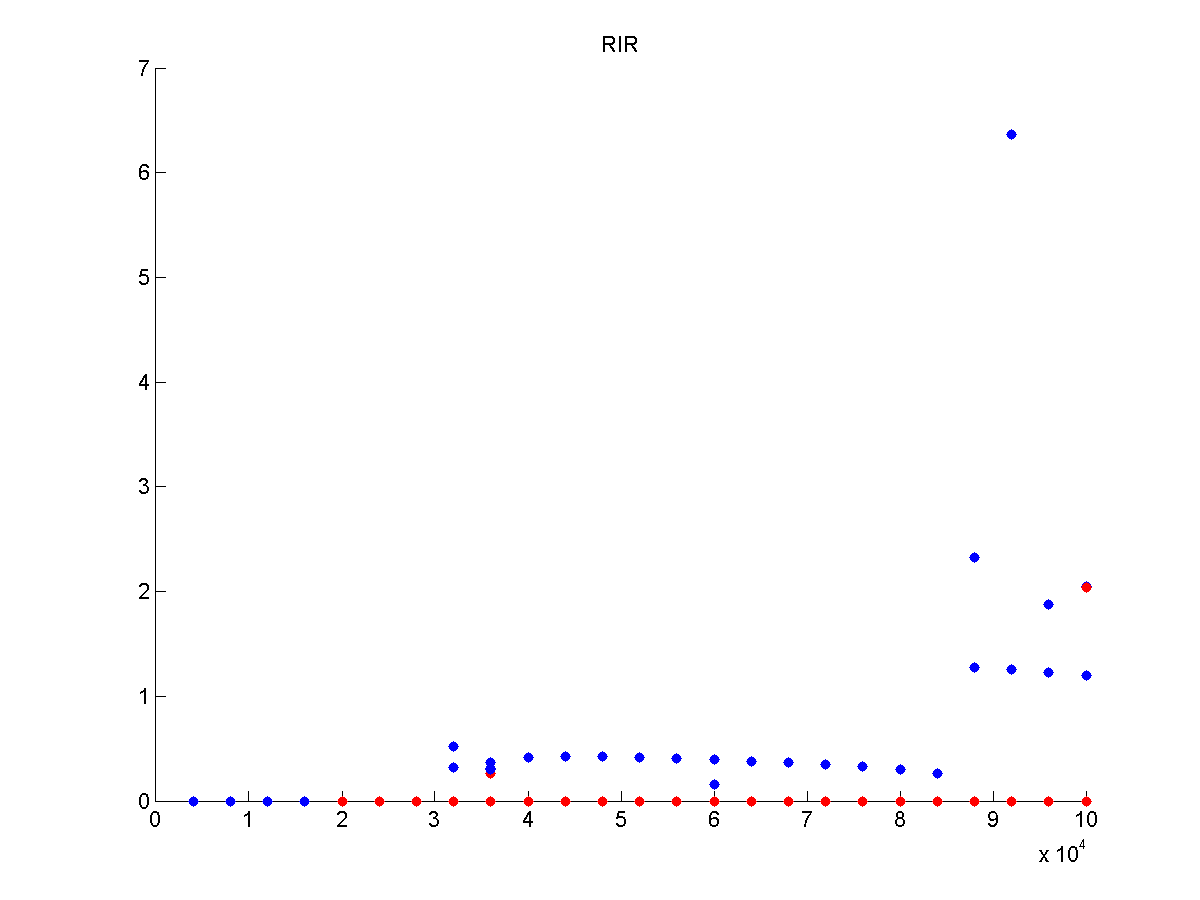

Supplement: Supplementary file 2 [file Presentation2.ZIP › RIR.png]

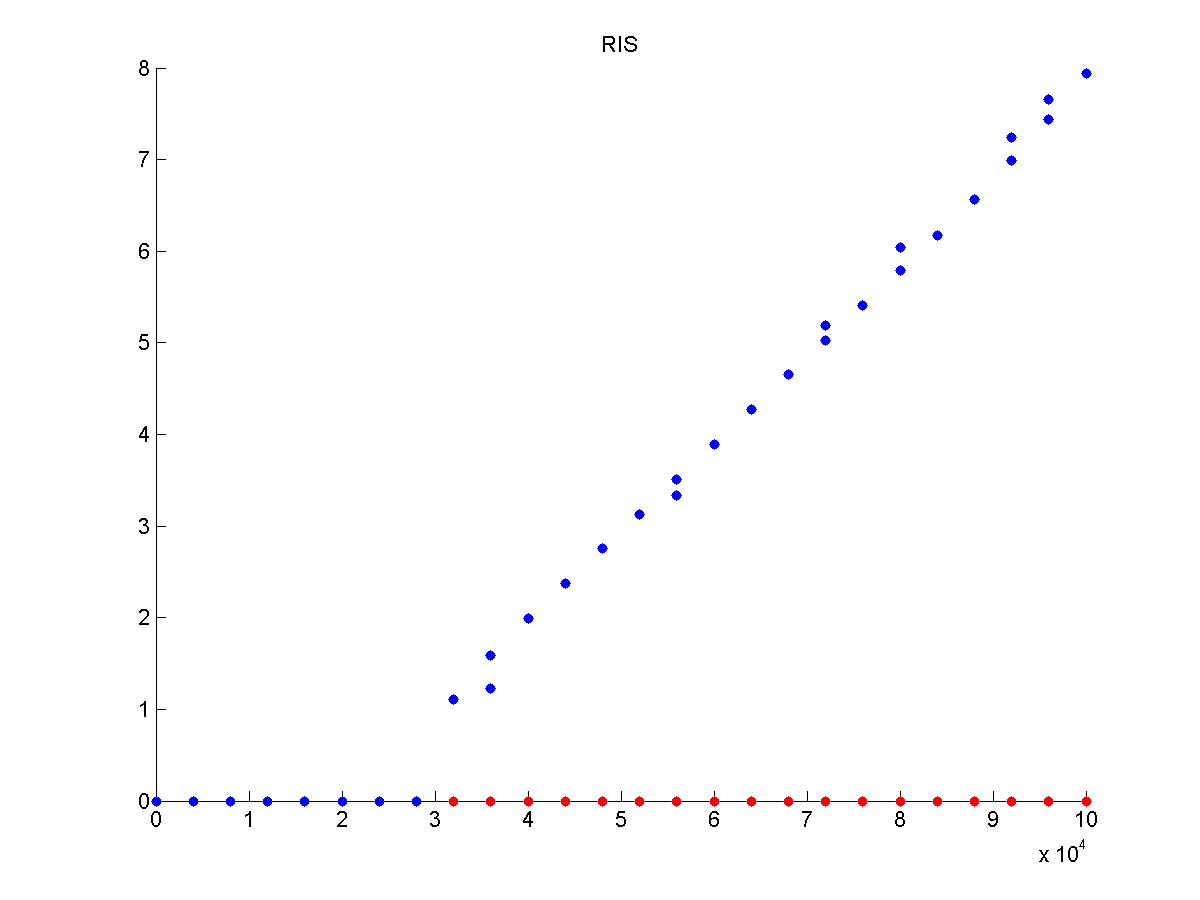

Supplement: Supplementary file 2 [file Presentation2.ZIP › RIS.png]

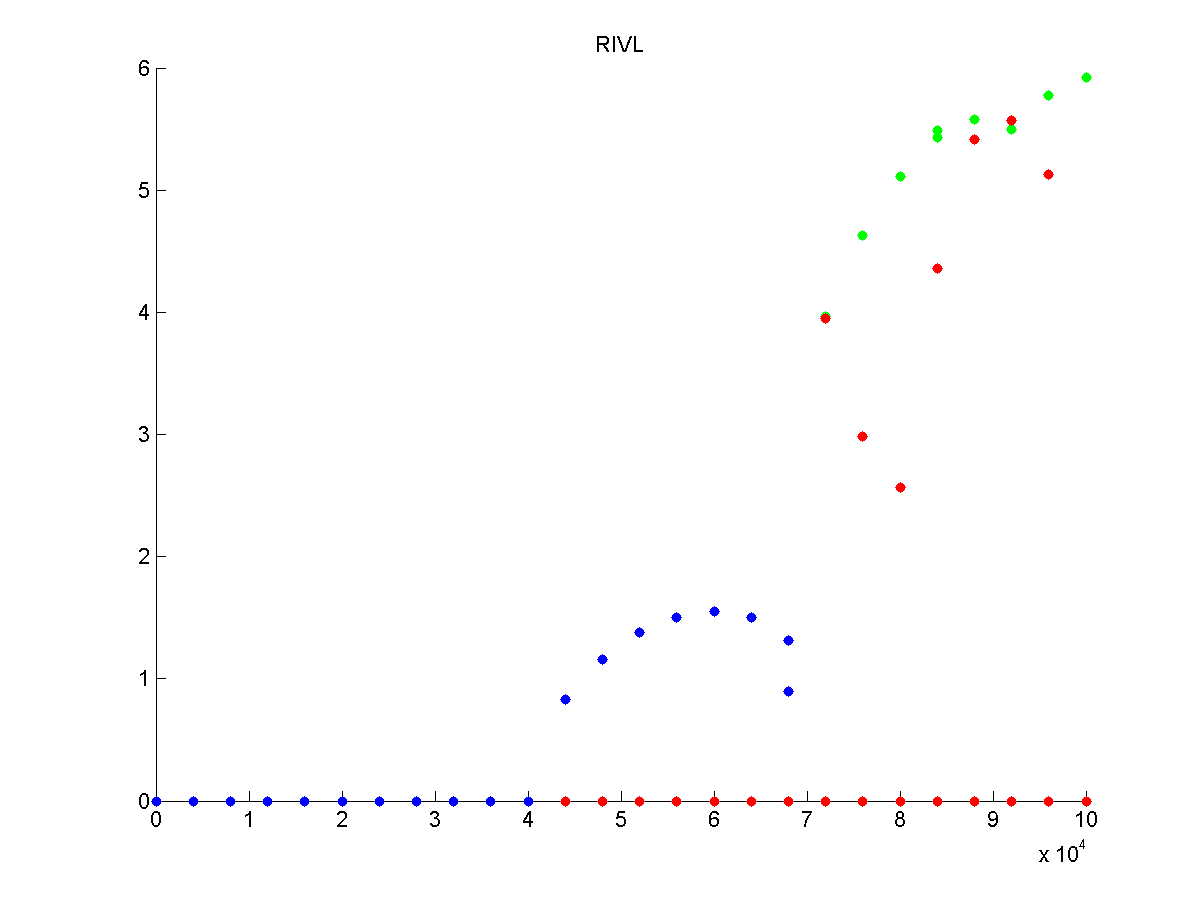

Supplement: Supplementary file 2 [file Presentation2.ZIP › RIVL.png]

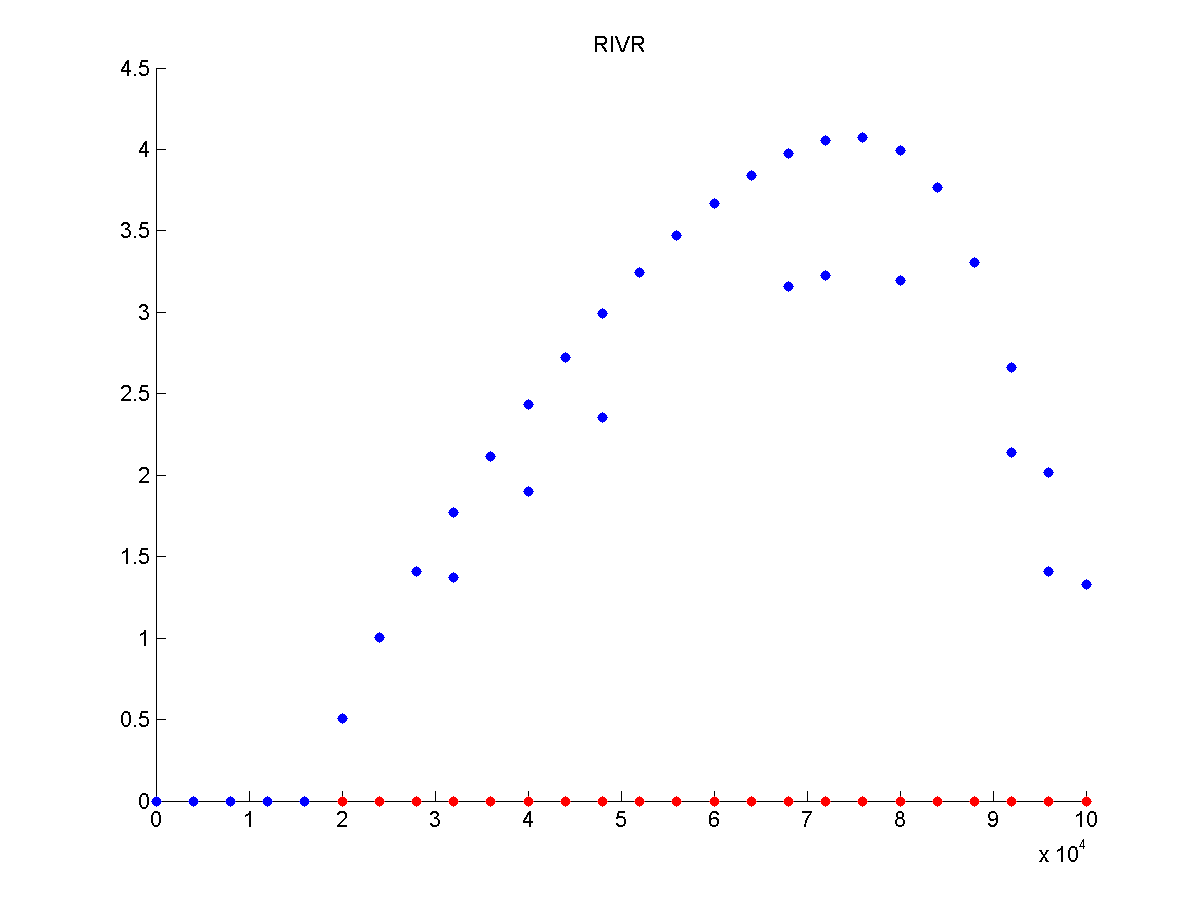

Supplement: Supplementary file 2 [file Presentation2.ZIP › RIVR.png]

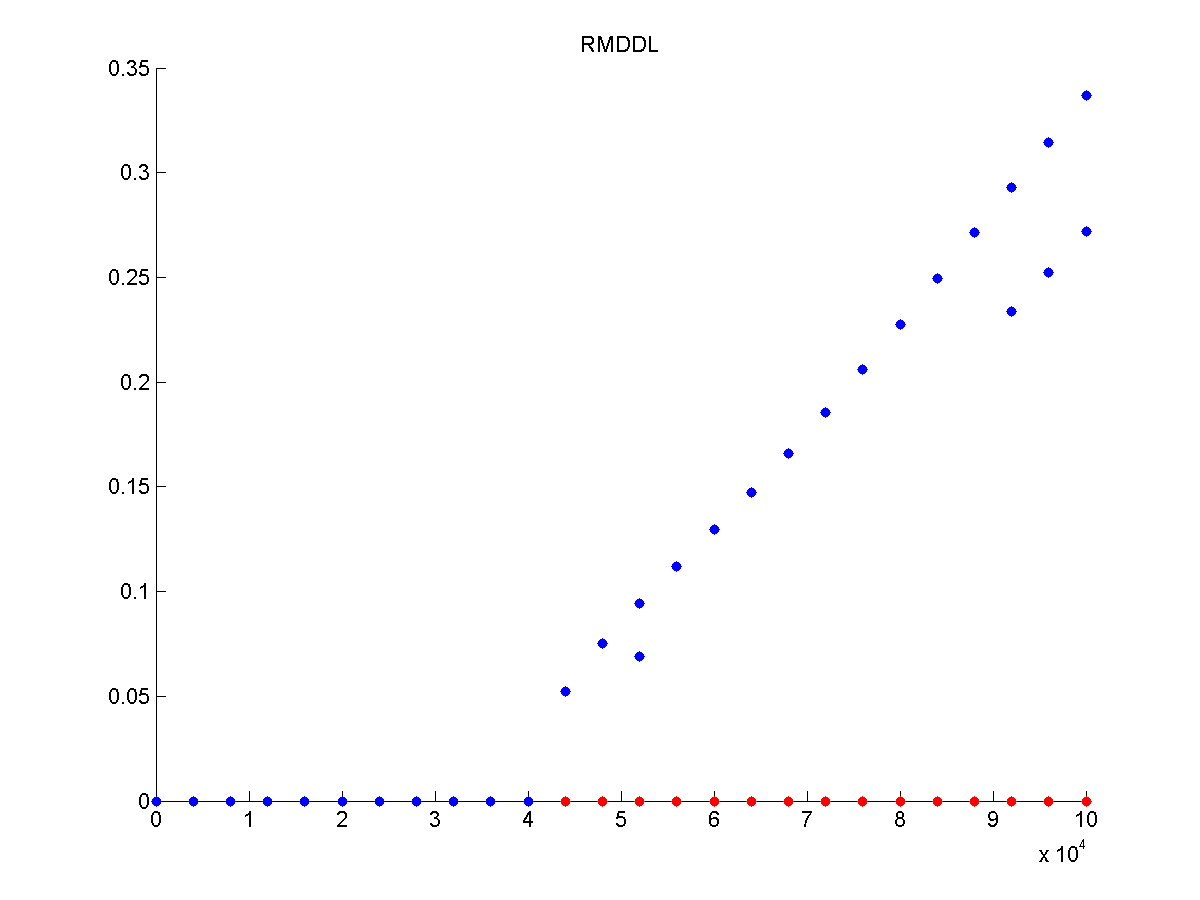

Supplement: Supplementary file 2 [file Presentation2.ZIP › RMDDL.png]

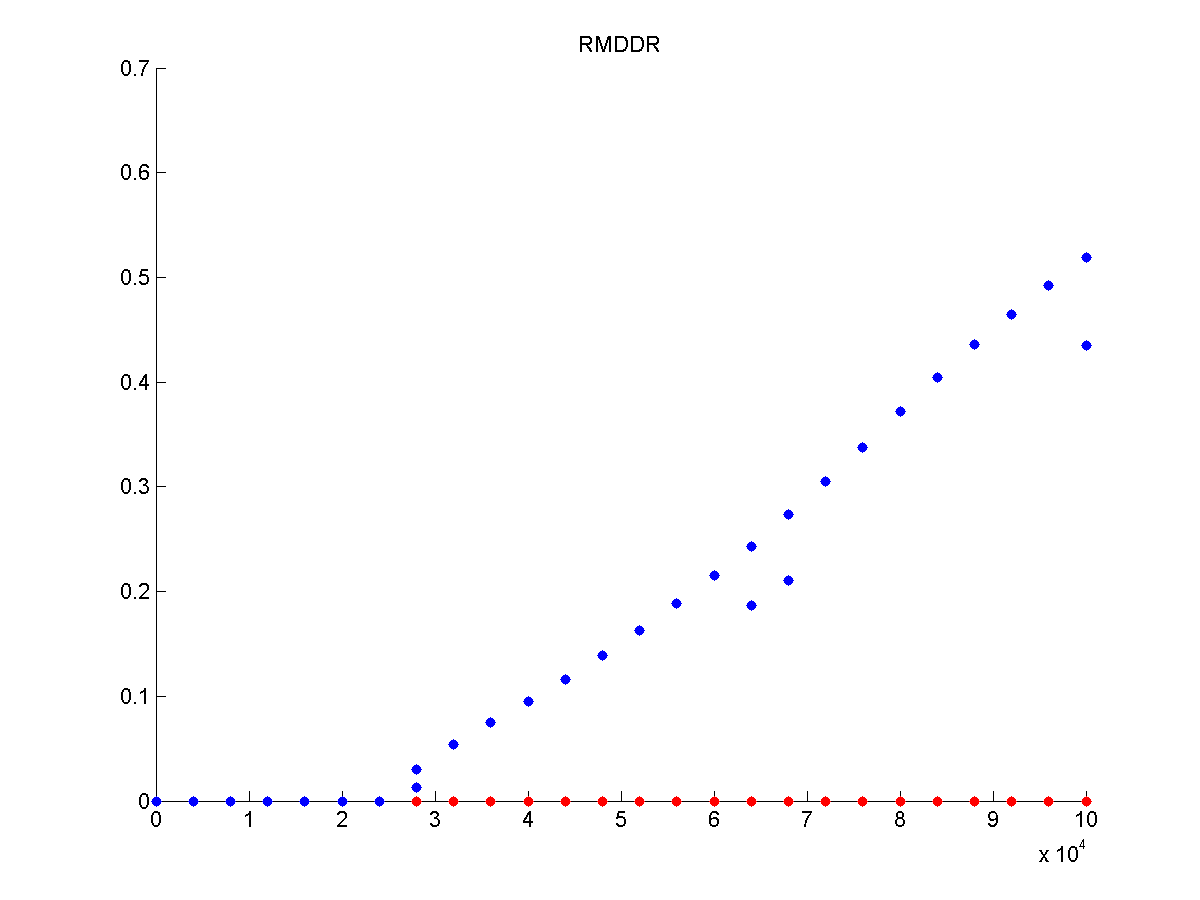

Supplement: Supplementary file 2 [file Presentation2.ZIP › RMDDR.png]

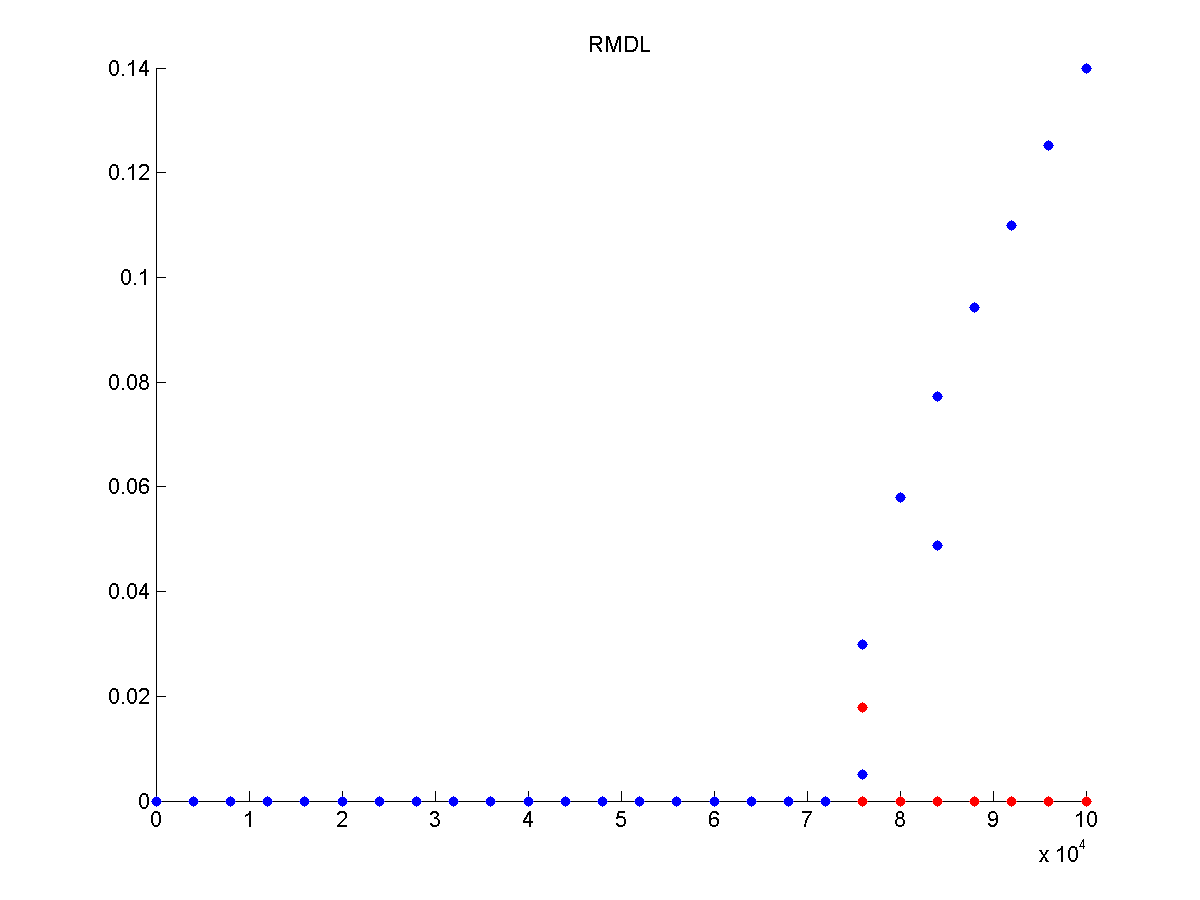

Supplement: Supplementary file 2 [file Presentation2.ZIP › RMDL.png]

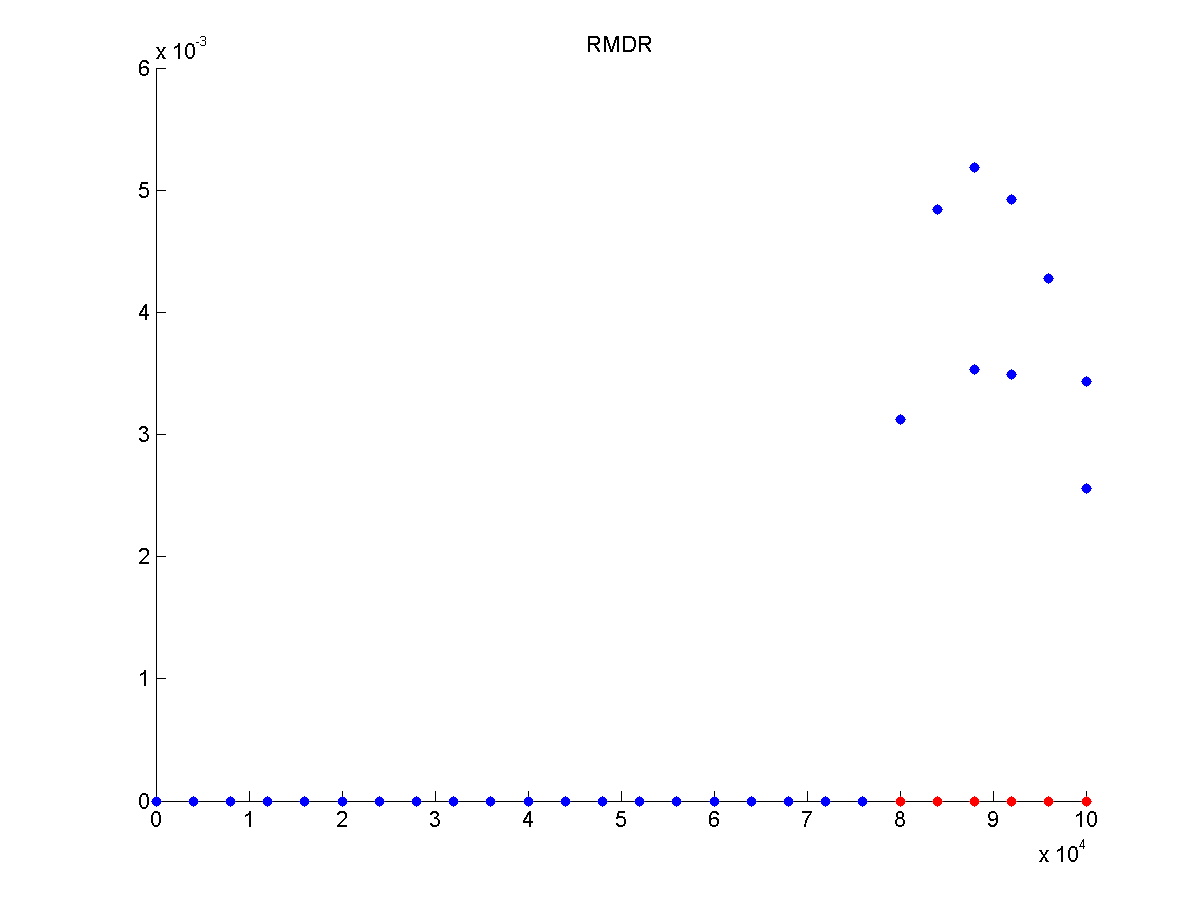

Supplement: Supplementary file 2 [file Presentation2.ZIP › RMDR.png]

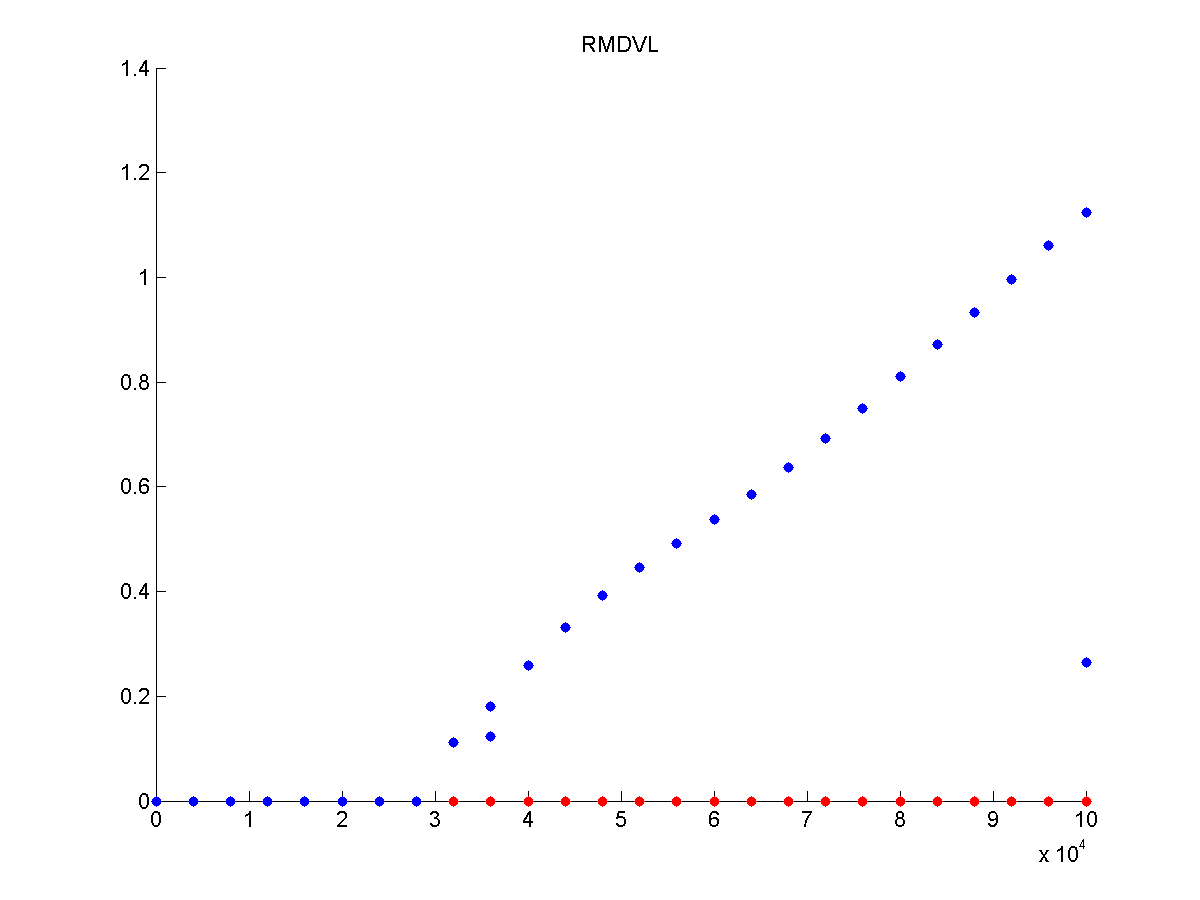

Supplement: Supplementary file 2 [file Presentation2.ZIP › RMDVL.png]

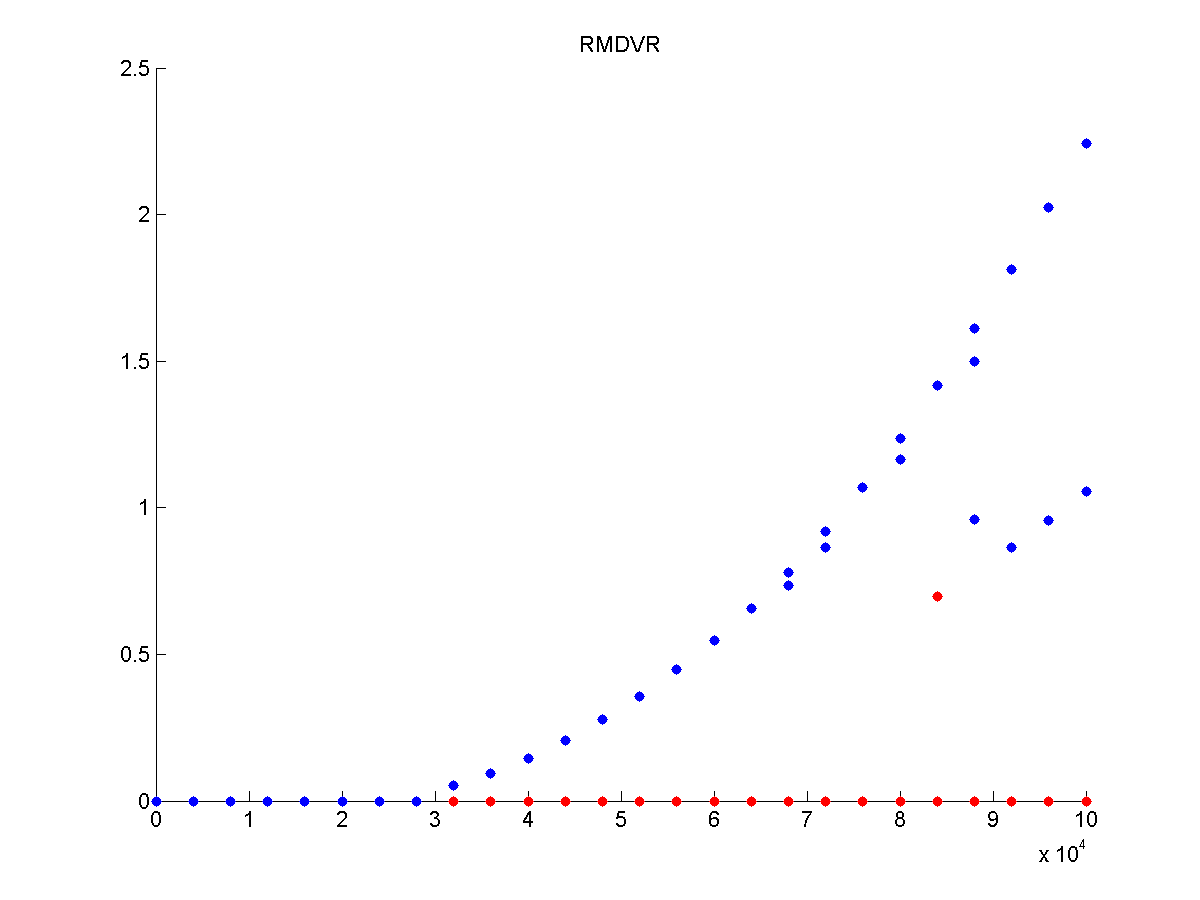

Supplement: Supplementary file 2 [file Presentation2.ZIP › RMDVR.png]

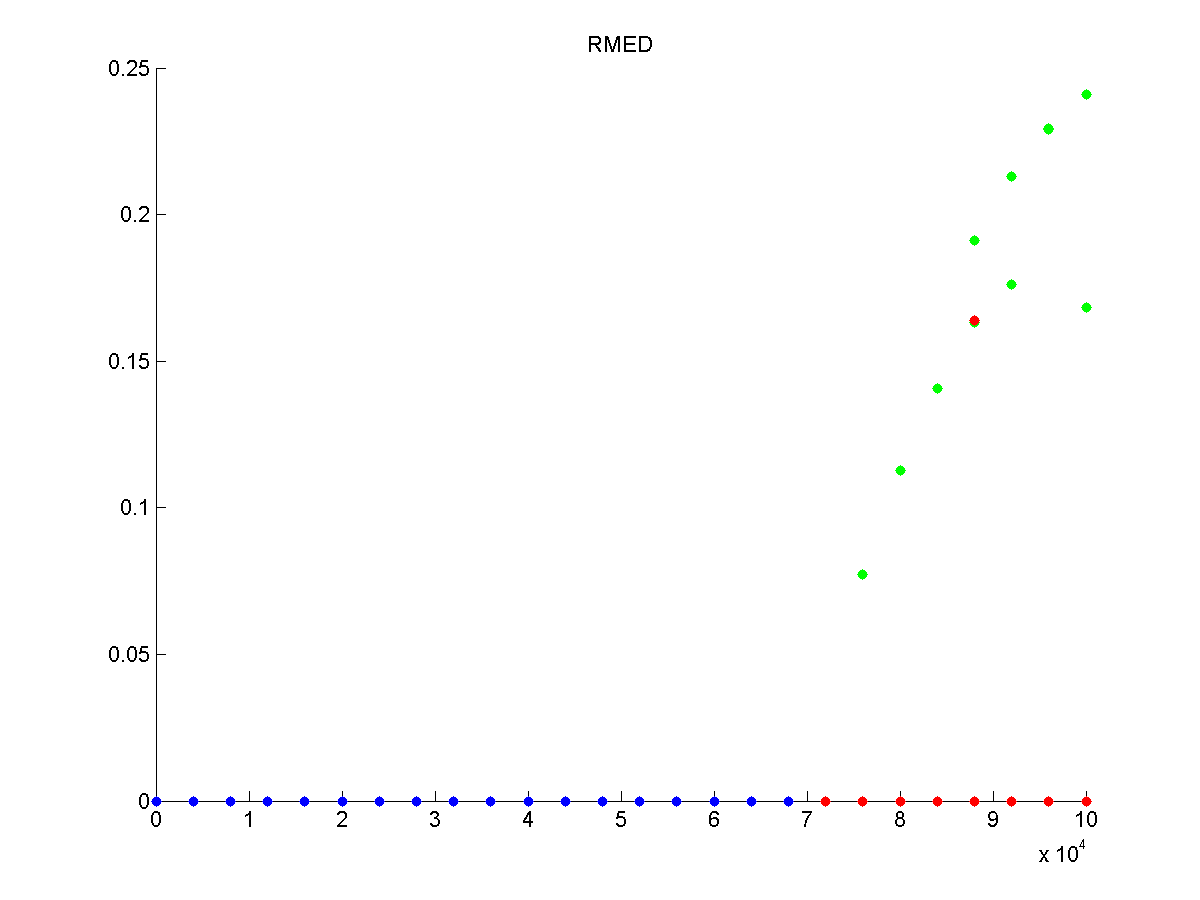

Supplement: Supplementary file 2 [file Presentation2.ZIP › RMED.png]

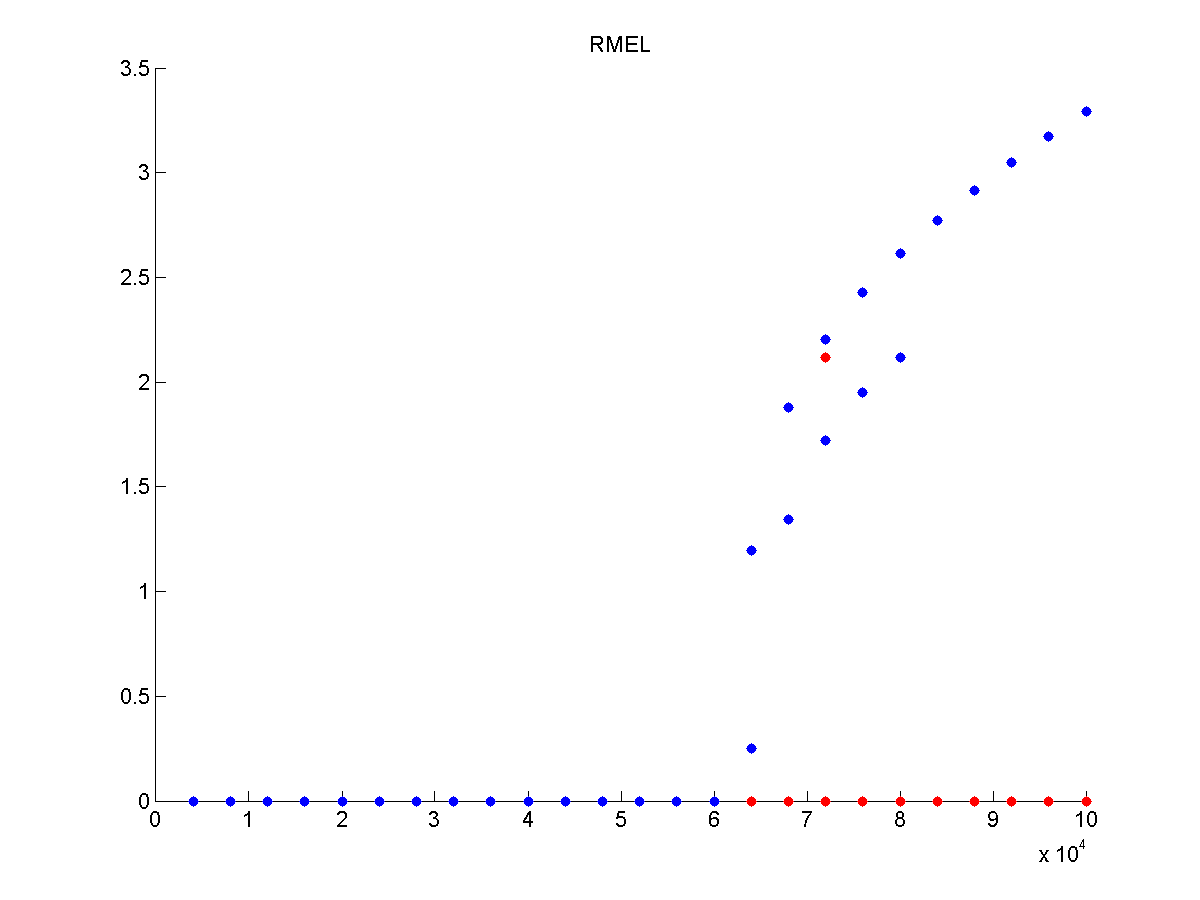

Supplement: Supplementary file 2 [file Presentation2.ZIP › RMEL.png]

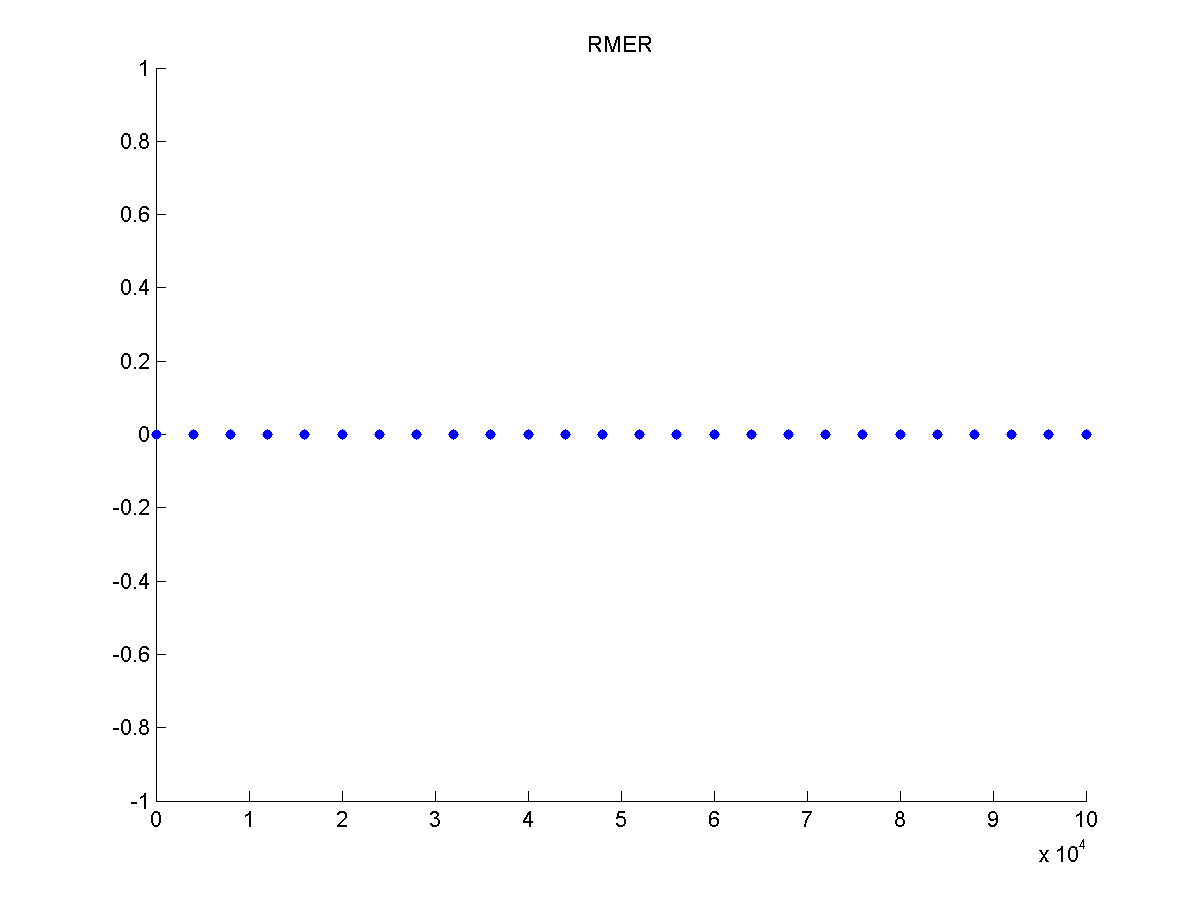

Supplement: Supplementary file 2 [file Presentation2.ZIP › RMER.png]

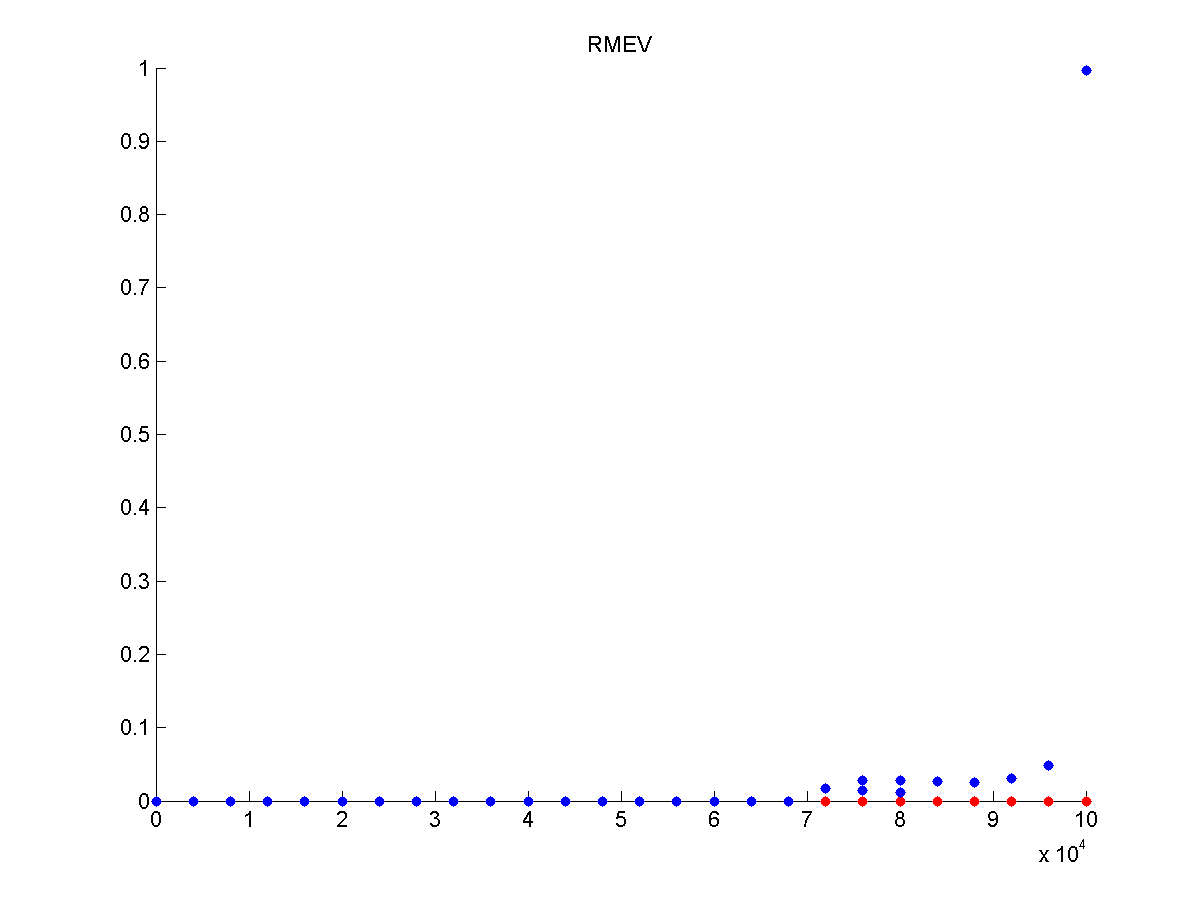

Supplement: Supplementary file 2 [file Presentation2.ZIP › RMEV.png]

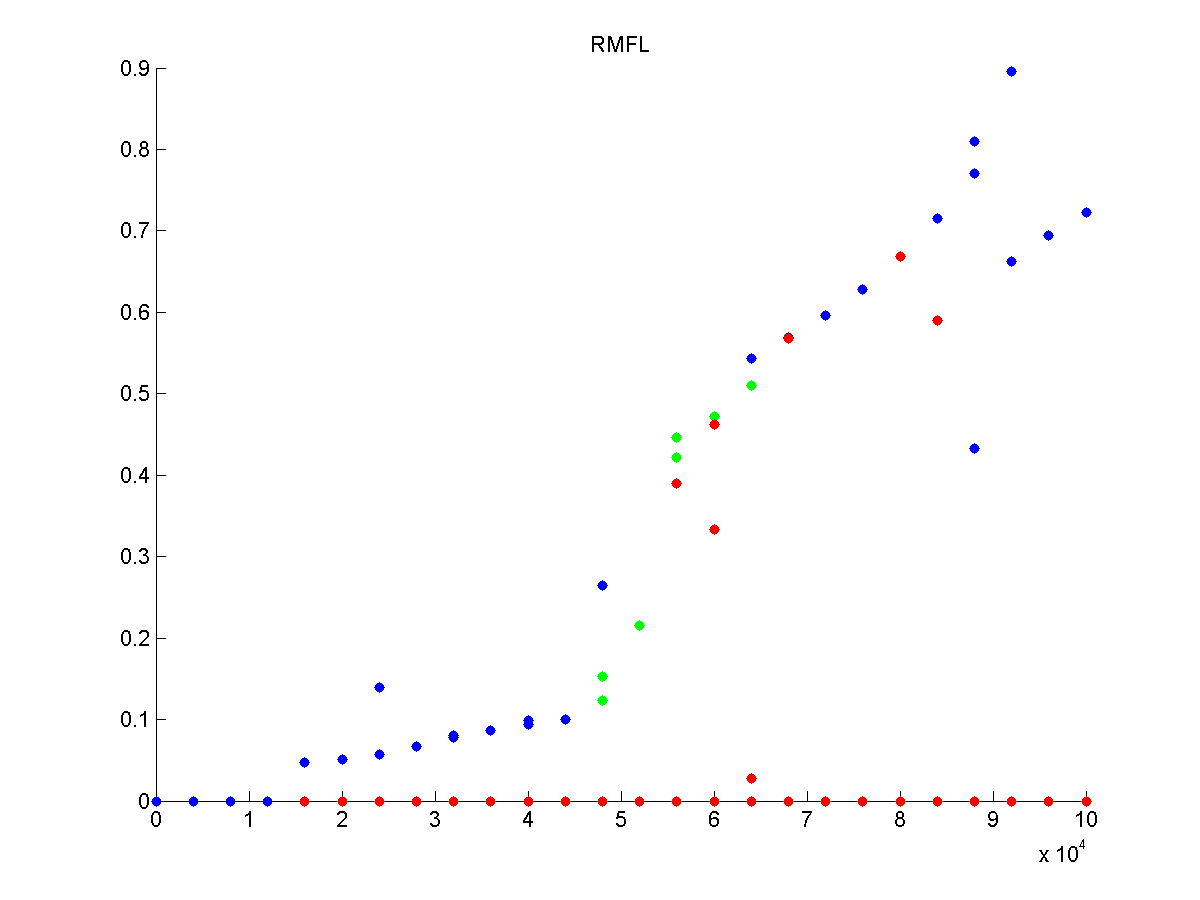

Supplement: Supplementary file 2 [file Presentation2.ZIP › RMFL.png]

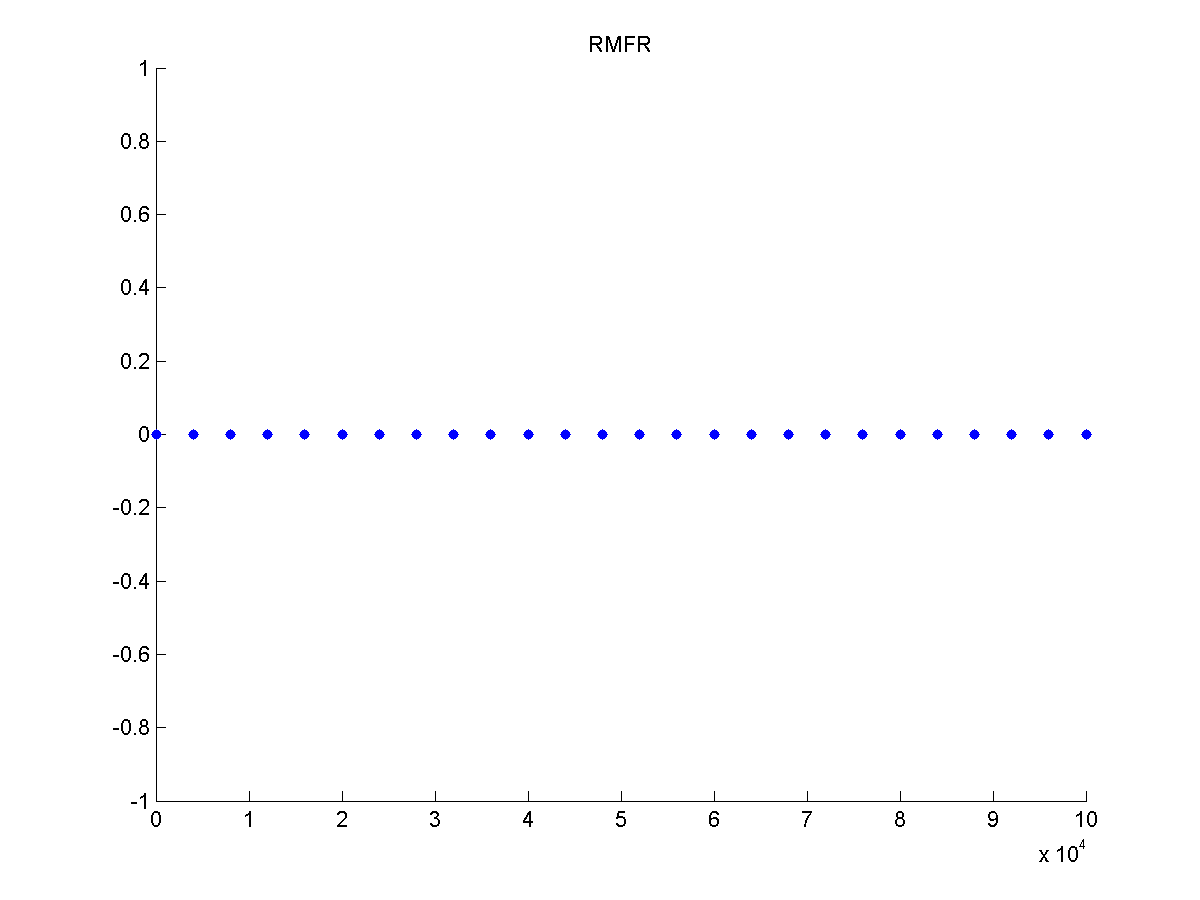

Supplement: Supplementary file 2 [file Presentation2.ZIP › RMFR.png]

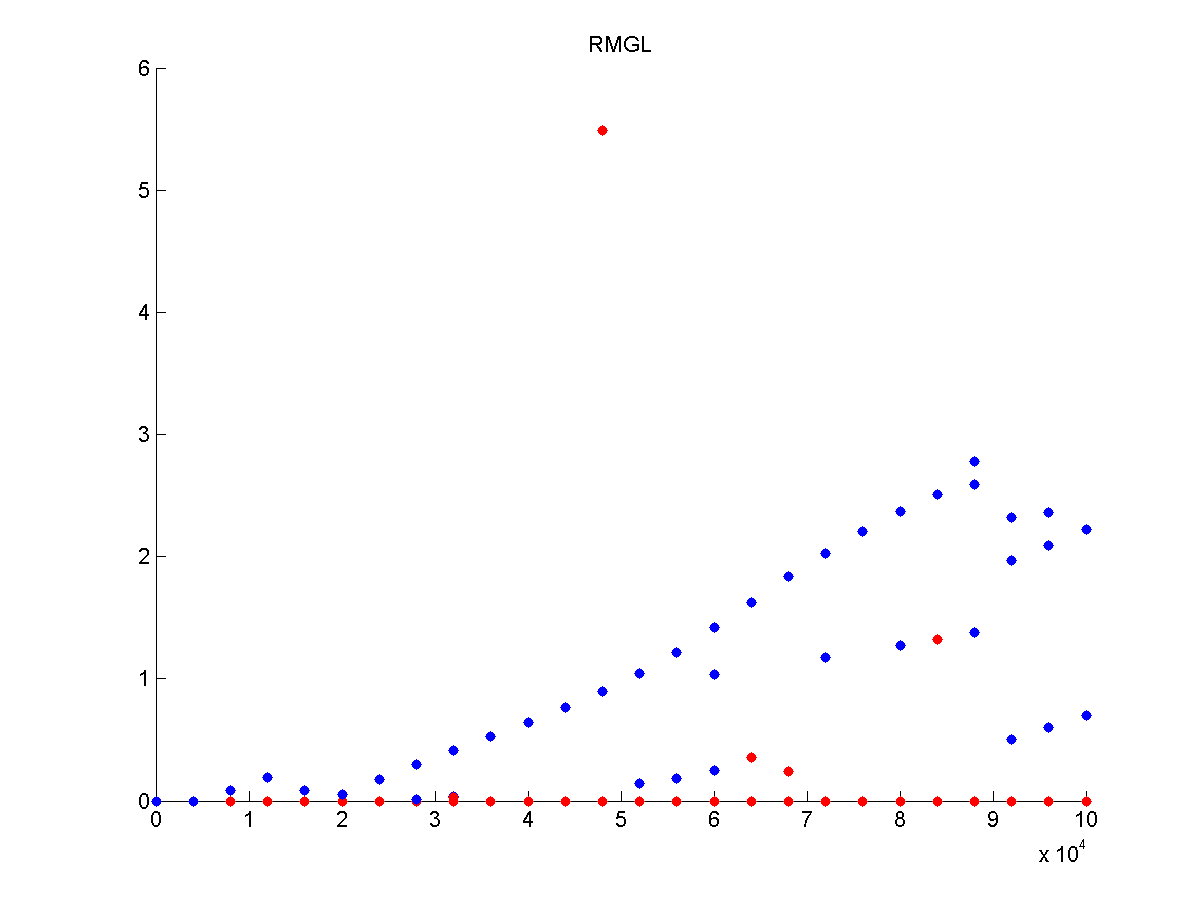

Supplement: Supplementary file 2 [file Presentation2.ZIP › RMGL.png]

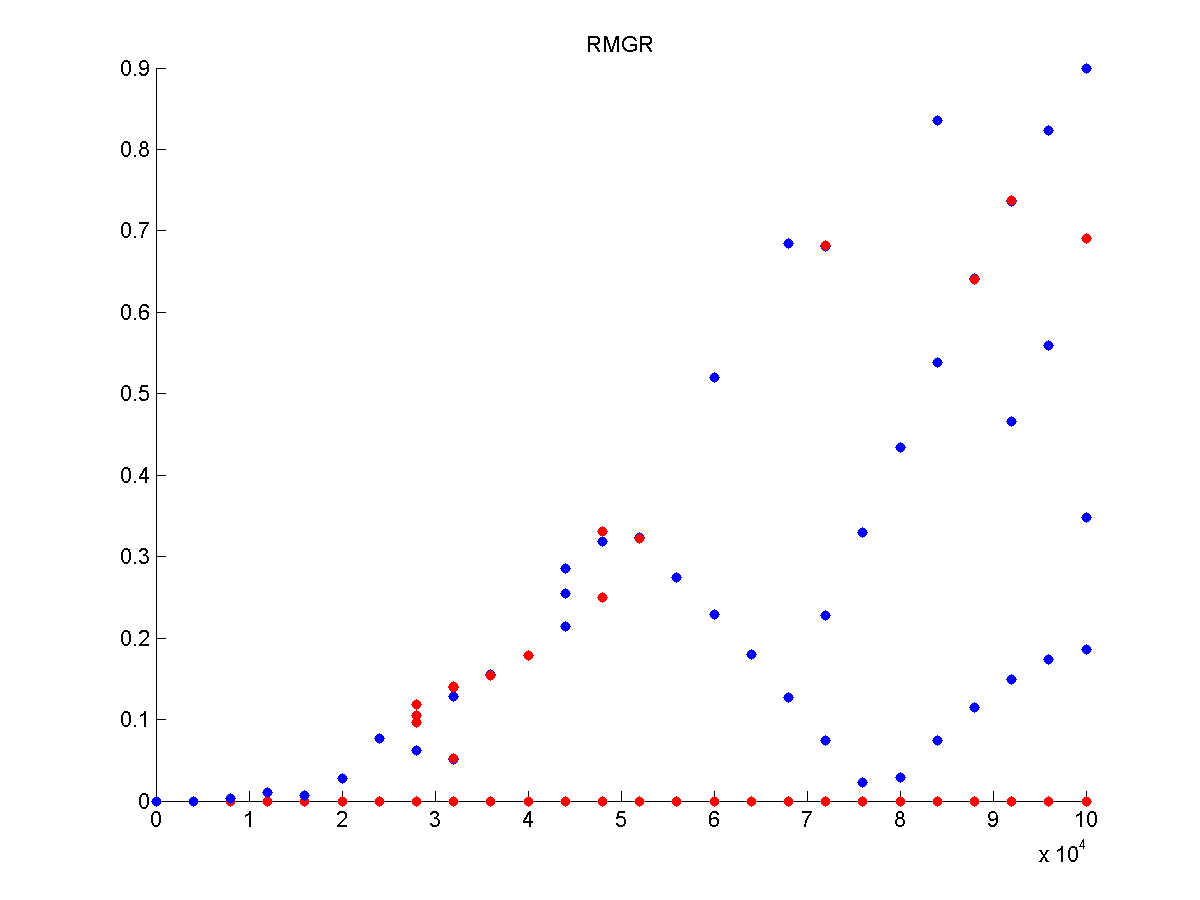

Supplement: Supplementary file 2 [file Presentation2.ZIP › RMGR.png]

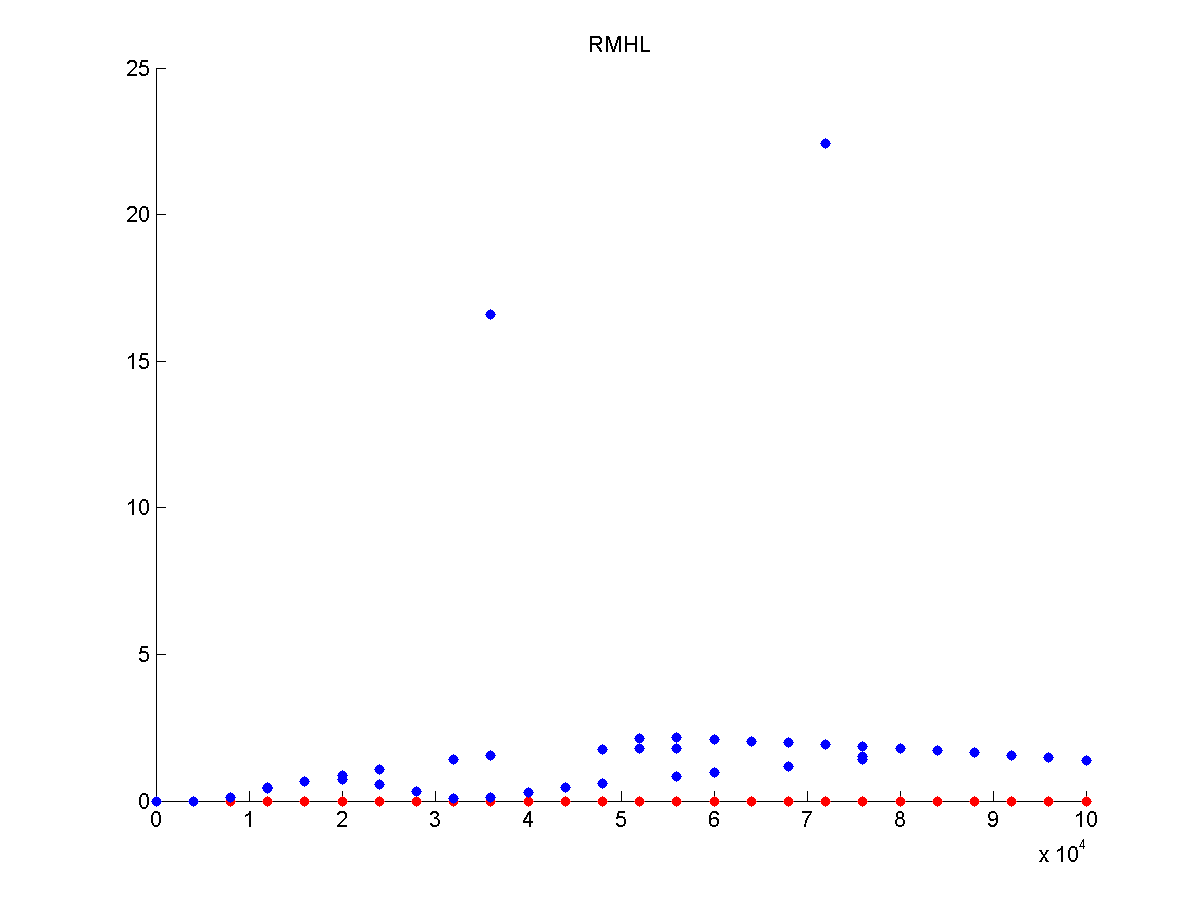

Supplement: Supplementary file 2 [file Presentation2.ZIP › RMHL.png]
