# Supplementary figures and images for: Multistability and Long-Timescale Transients Encoded by Network Structure in a Model of C. elegans Connectome Dynamics (part 1 of 2)
Source: Front Comput Neurosci. 2017 Jun 13;11:53. doi: 10.3389/fncom.2017.00053 (PMC5468412; doi:10.3389/fncom.2017.00053)

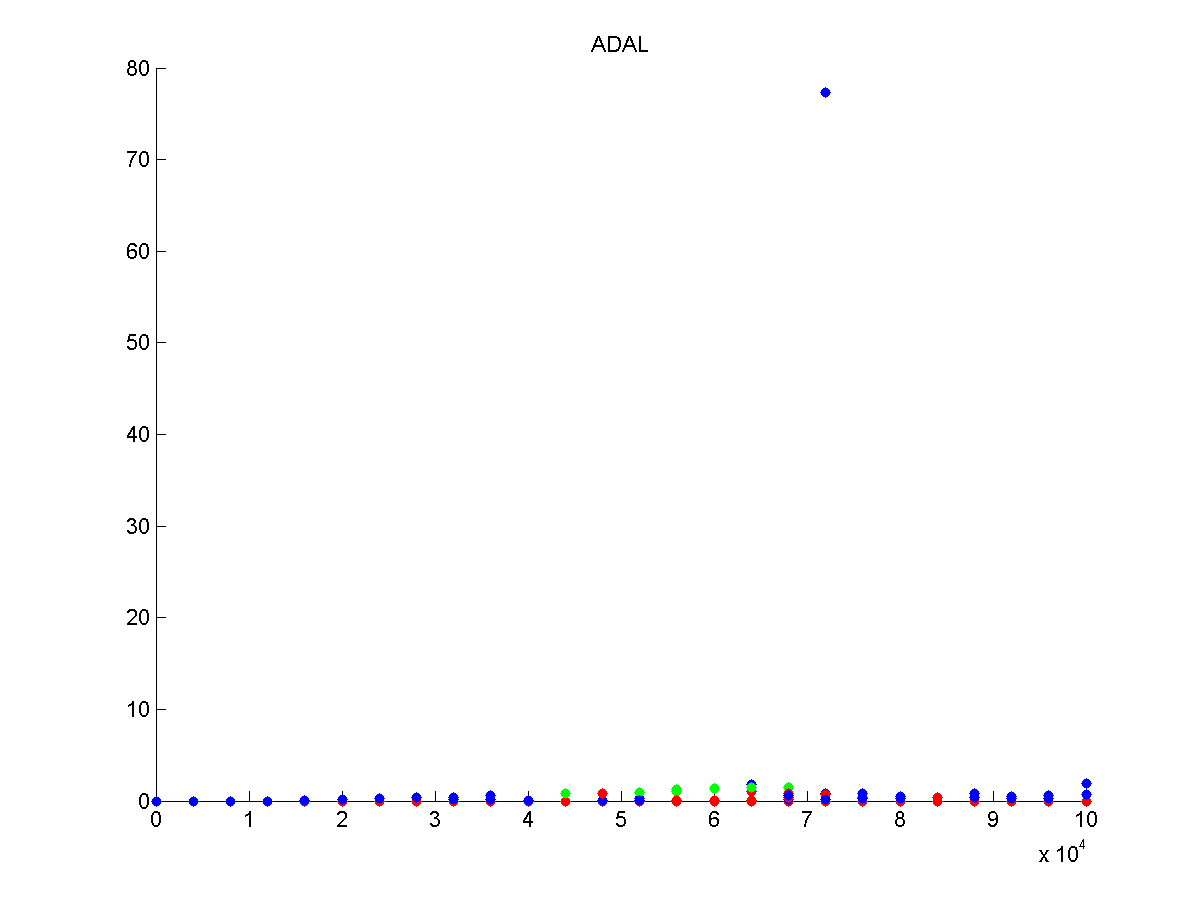

Supplement: Supplementary file 2 [file Presentation2.ZIP › ADAL.png]

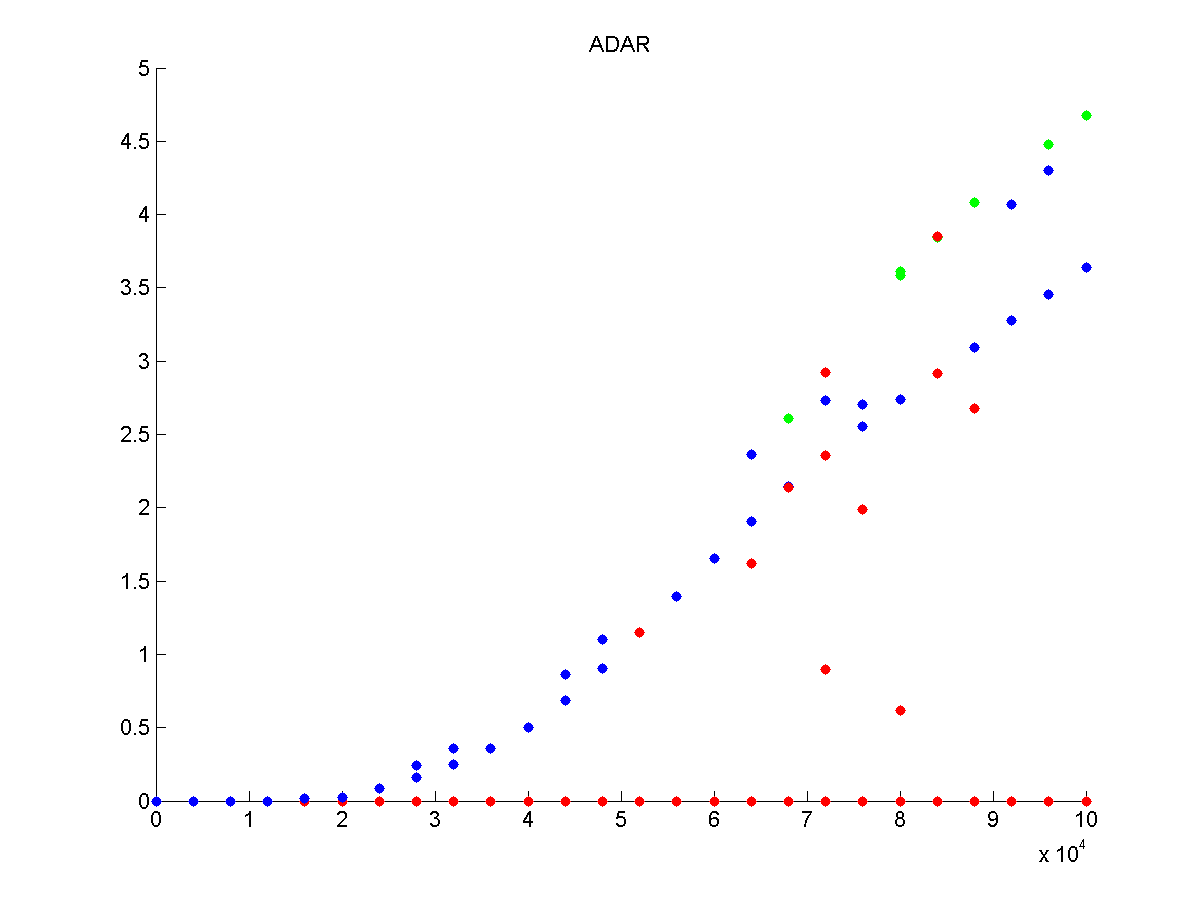

Supplement: Supplementary file 2 [file Presentation2.ZIP › ADAR.png]

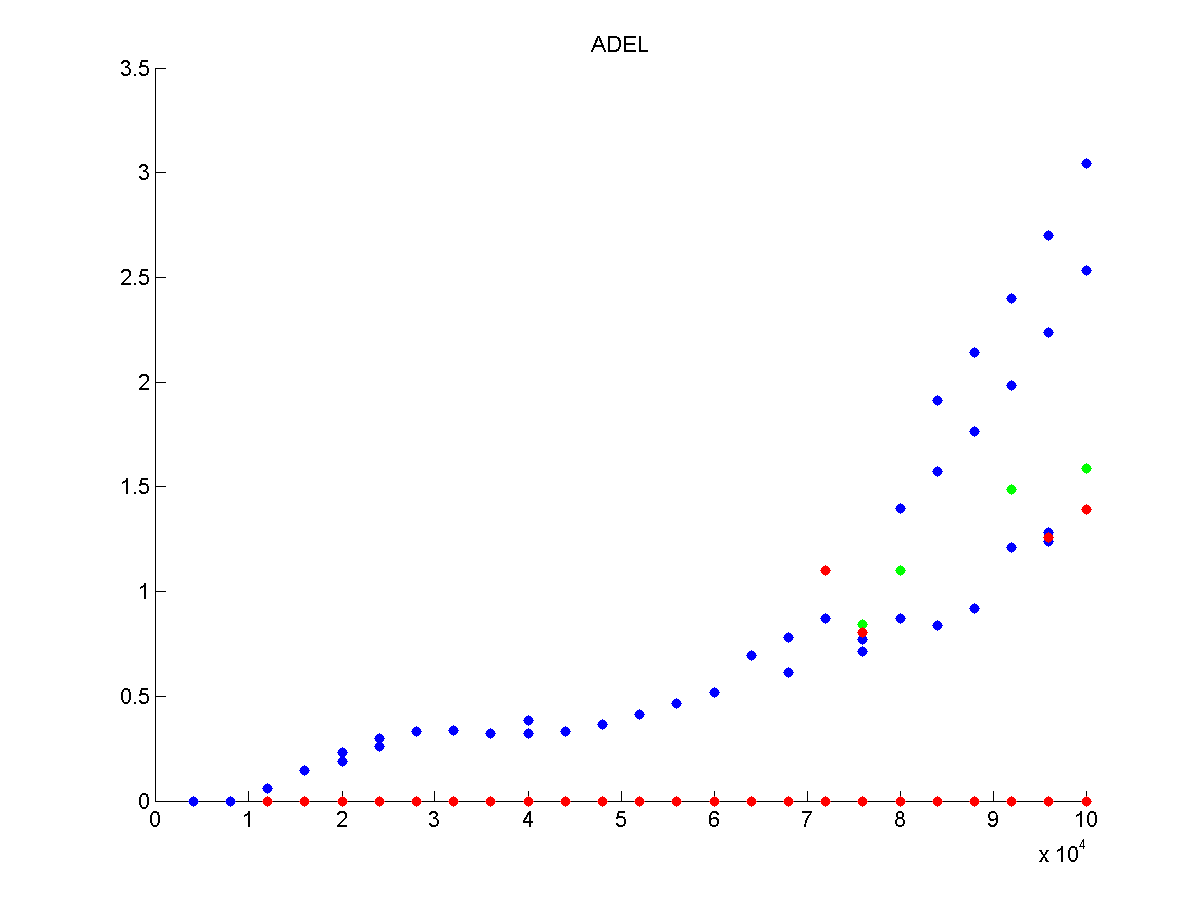

Supplement: Supplementary file 2 [file Presentation2.ZIP › ADEL.png]

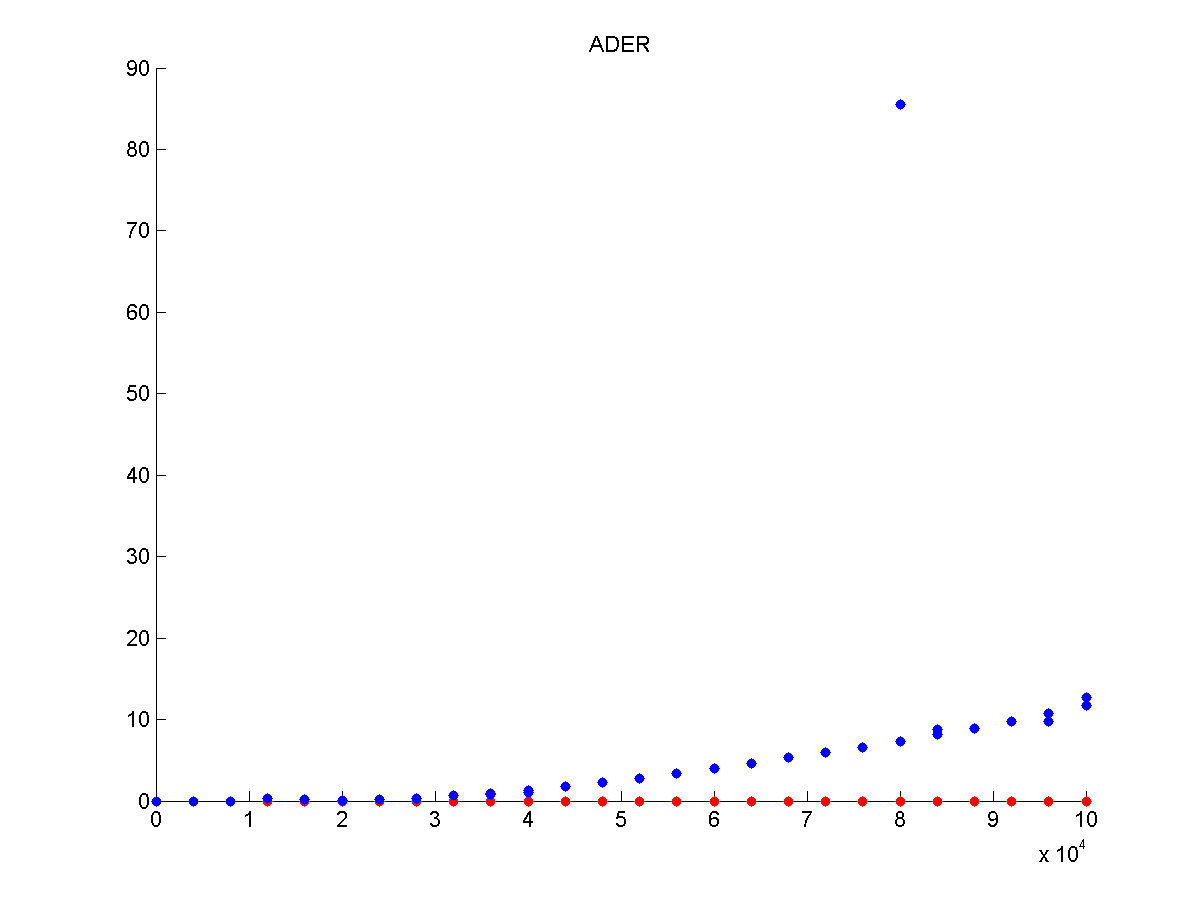

Supplement: Supplementary file 2 [file Presentation2.ZIP › ADER.png]

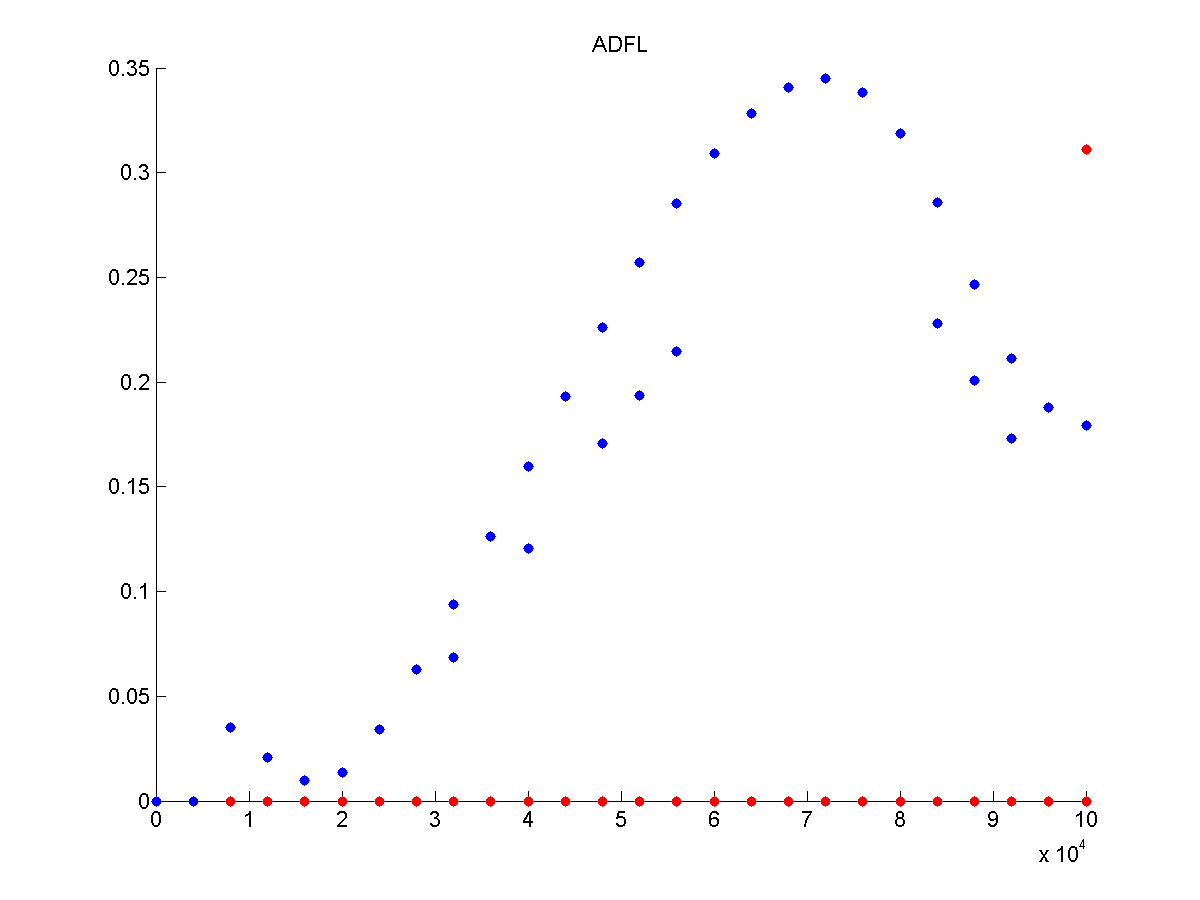

Supplement: Supplementary file 2 [file Presentation2.ZIP › ADFL.png]

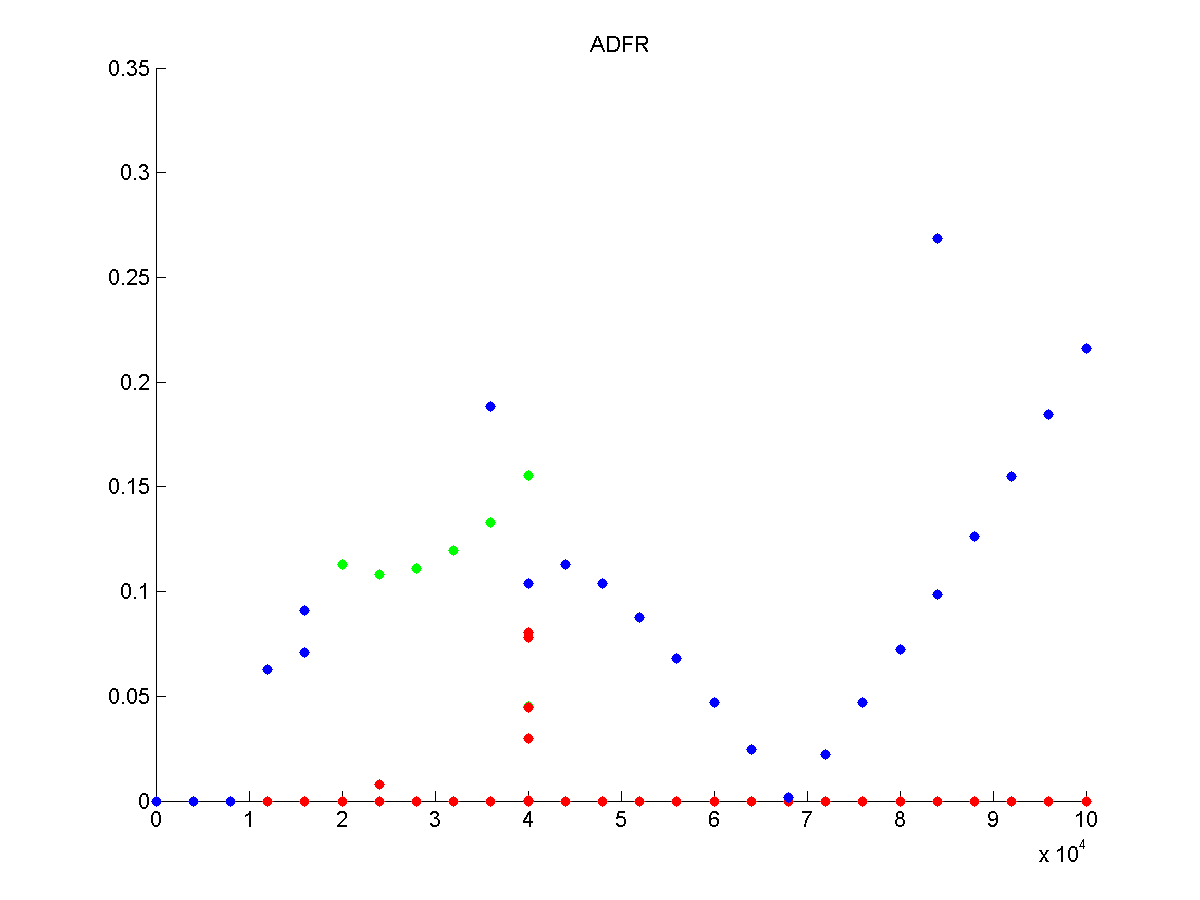

Supplement: Supplementary file 2 [file Presentation2.ZIP › ADFR.png]

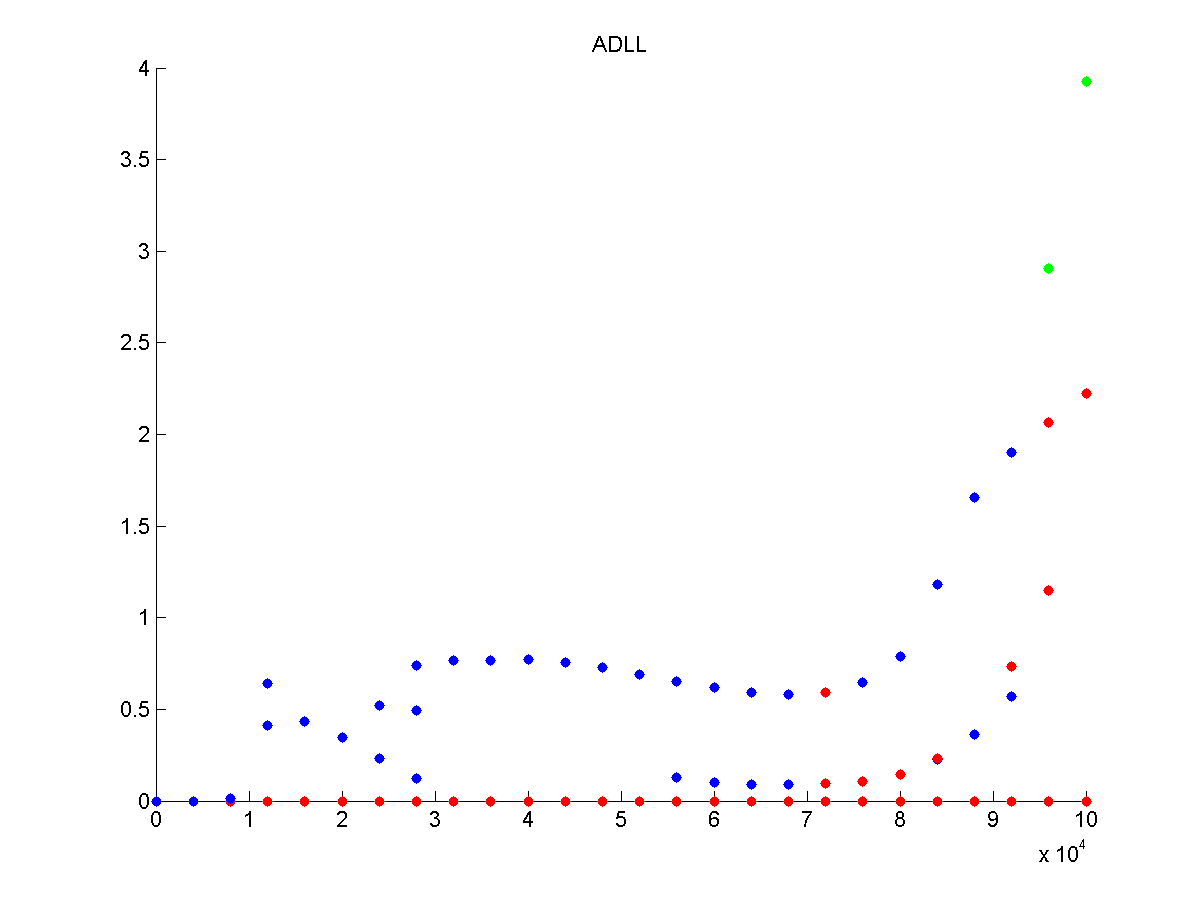

Supplement: Supplementary file 2 [file Presentation2.ZIP › ADLL.png]

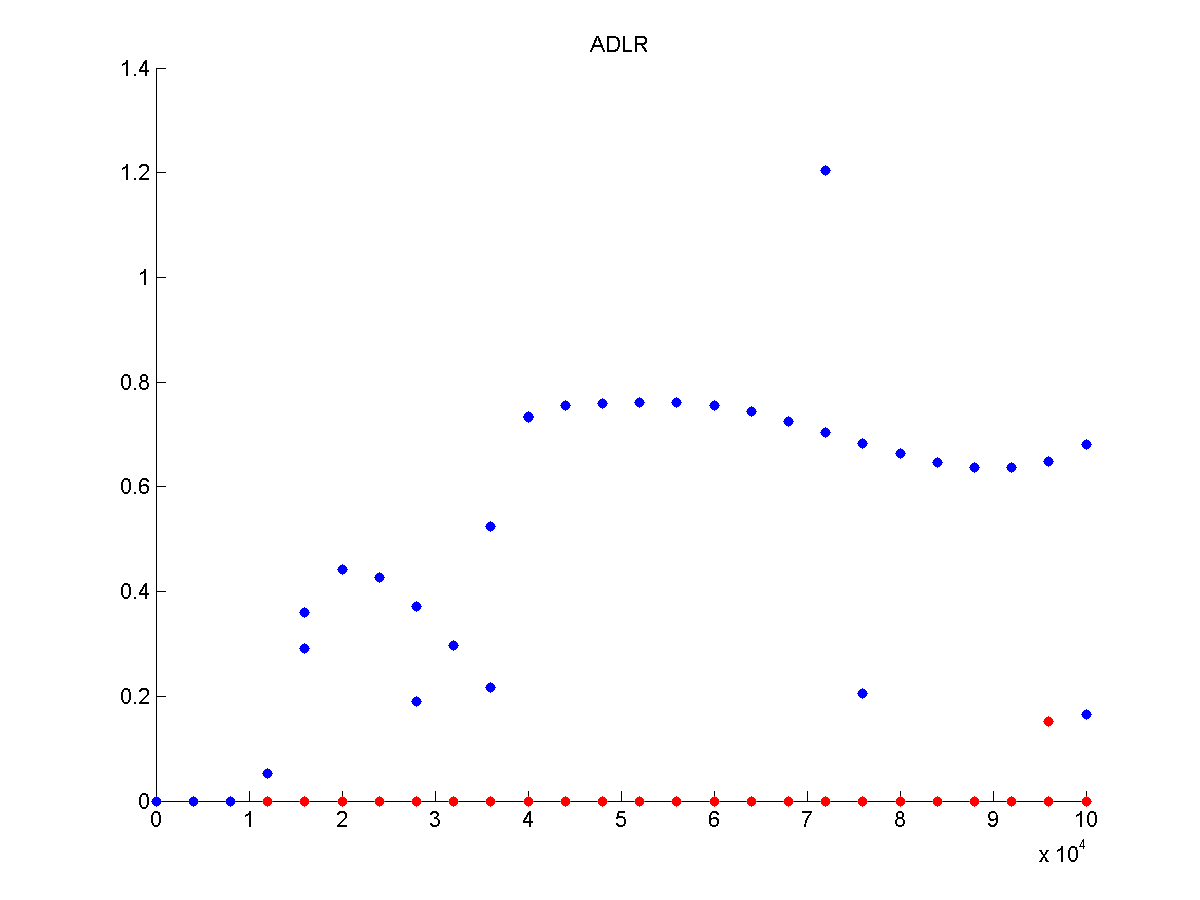

Supplement: Supplementary file 2 [file Presentation2.ZIP › ADLR.png]

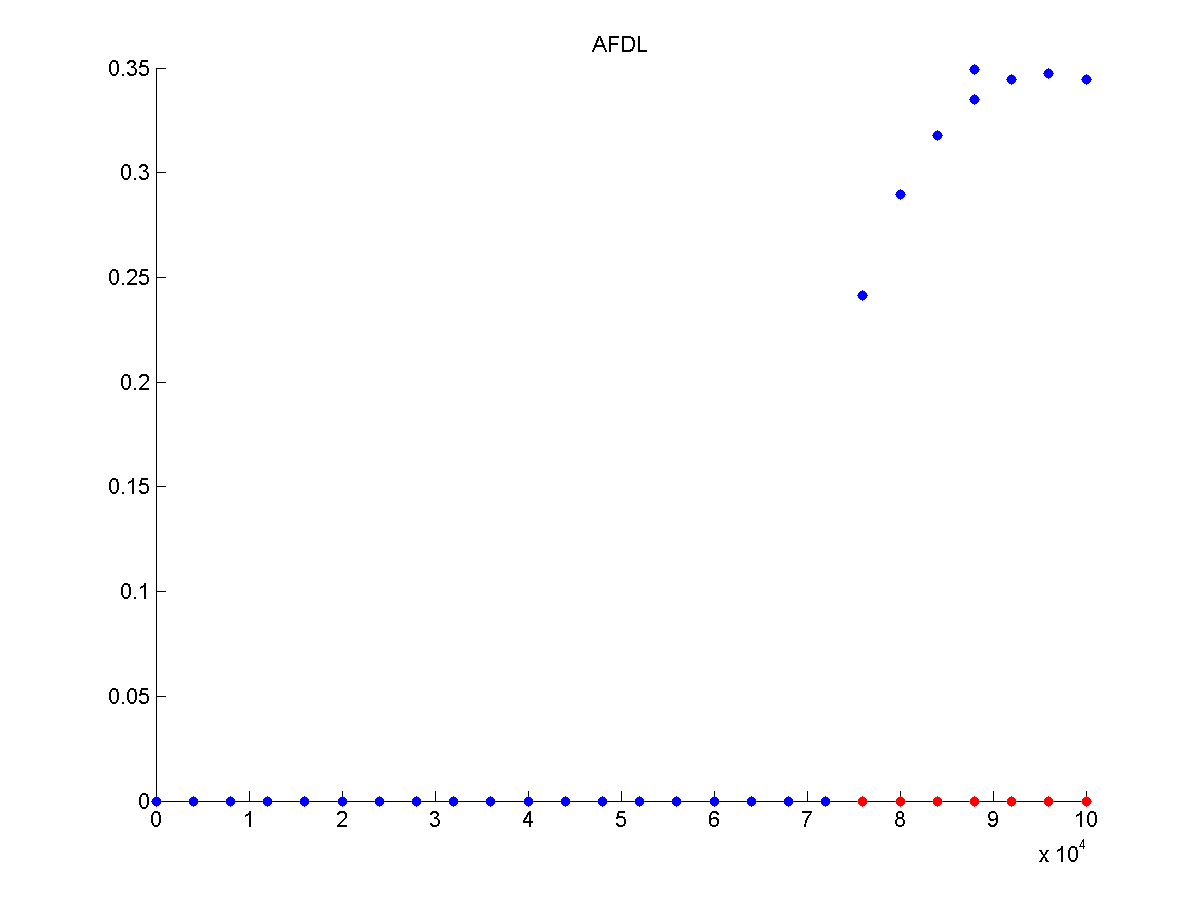

Supplement: Supplementary file 2 [file Presentation2.ZIP › AFDL.png]

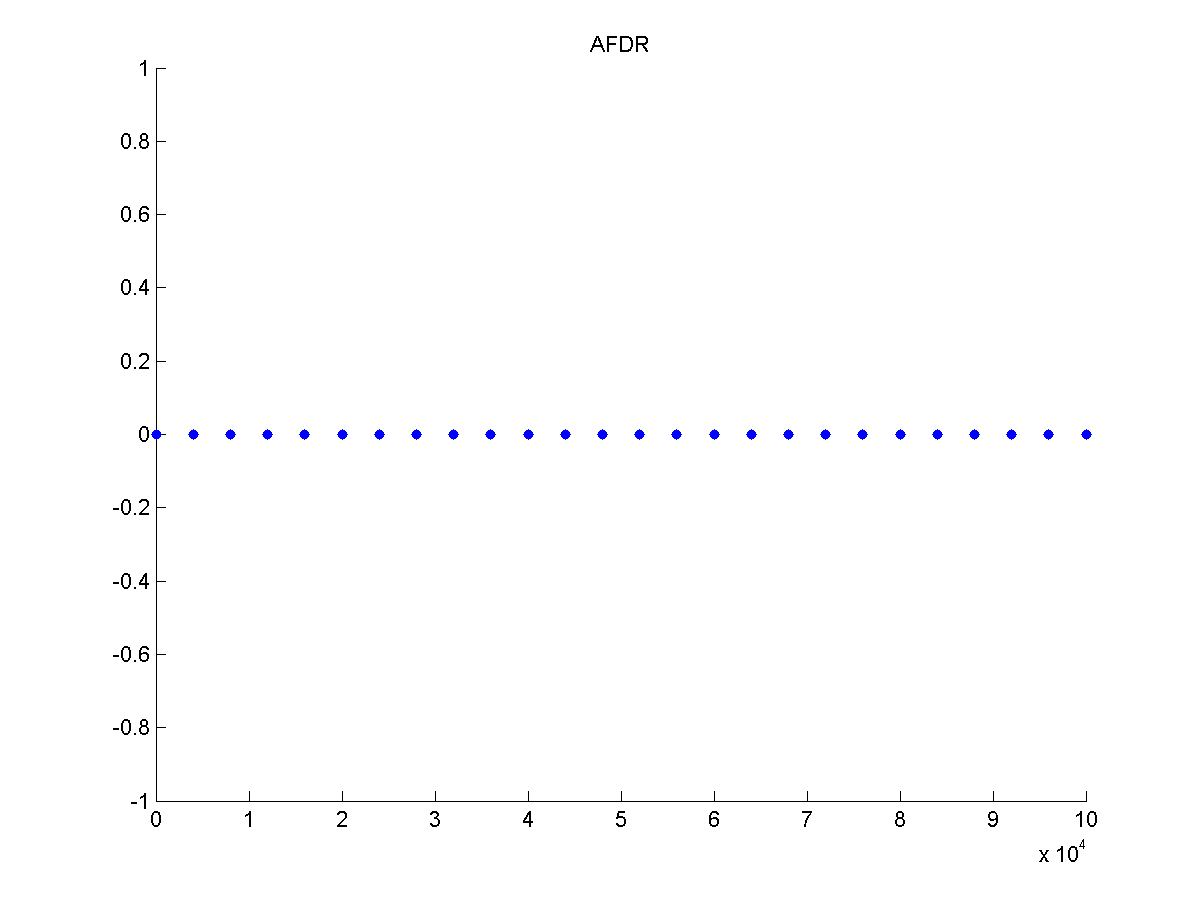

Supplement: Supplementary file 2 [file Presentation2.ZIP › AFDR.png]

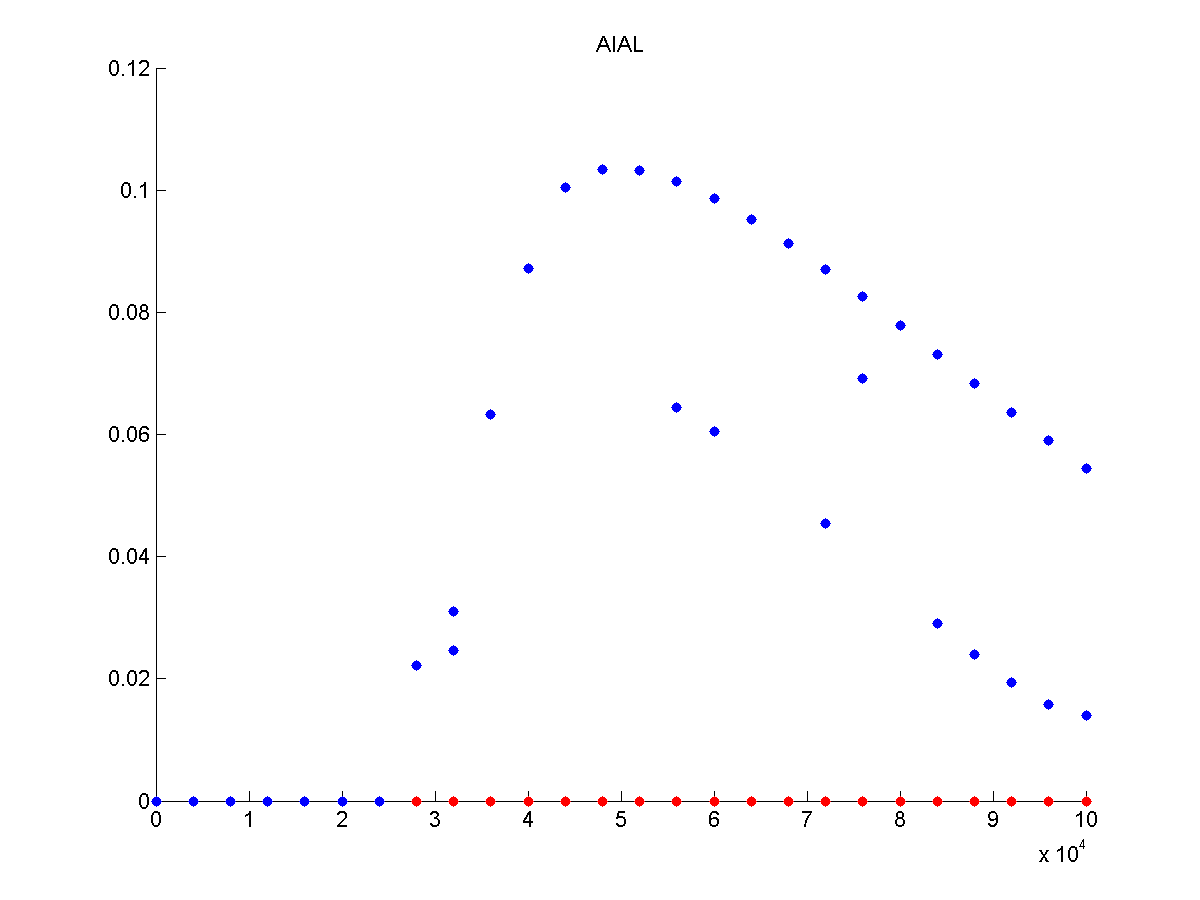

Supplement: Supplementary file 2 [file Presentation2.ZIP › AIAL.png]

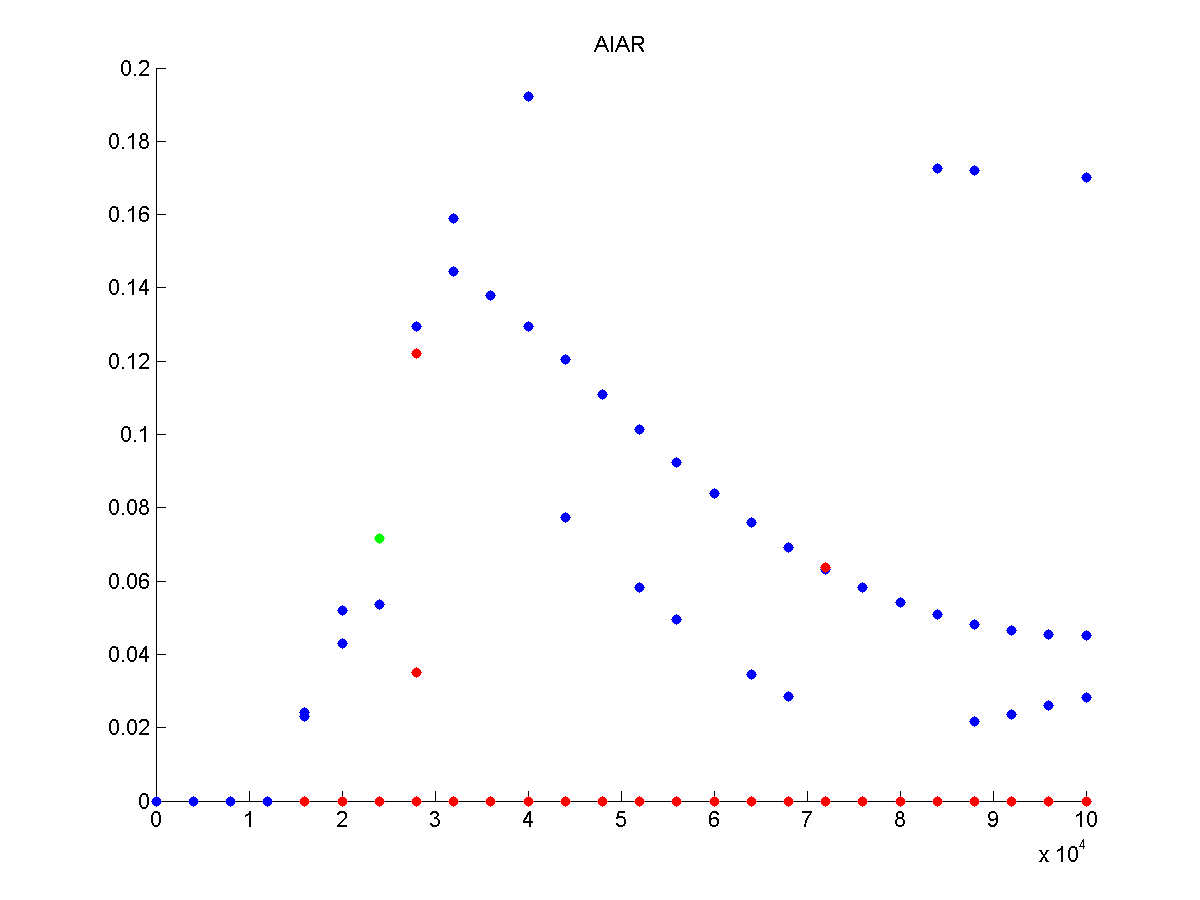

Supplement: Supplementary file 2 [file Presentation2.ZIP › AIAR.png]

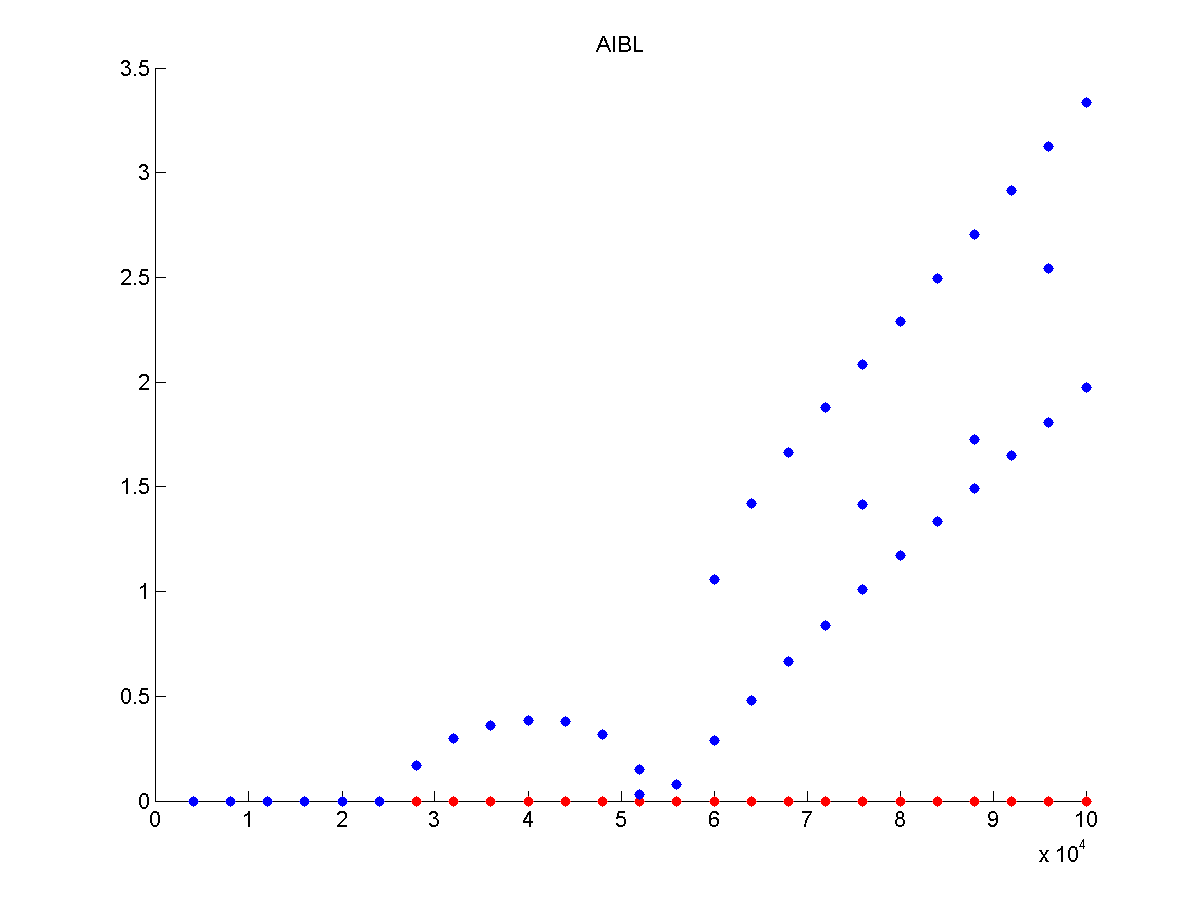

Supplement: Supplementary file 2 [file Presentation2.ZIP › AIBL.png]

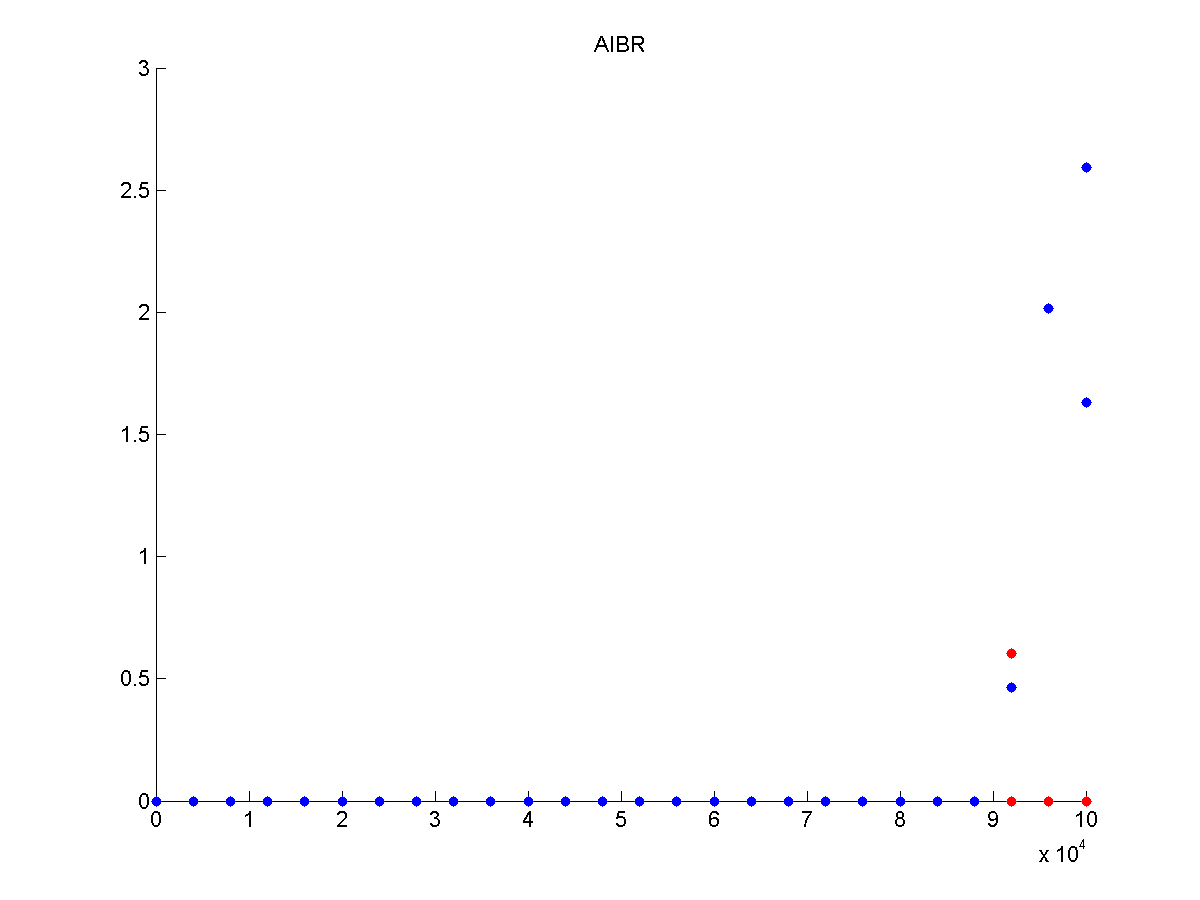

Supplement: Supplementary file 2 [file Presentation2.ZIP › AIBR.png]

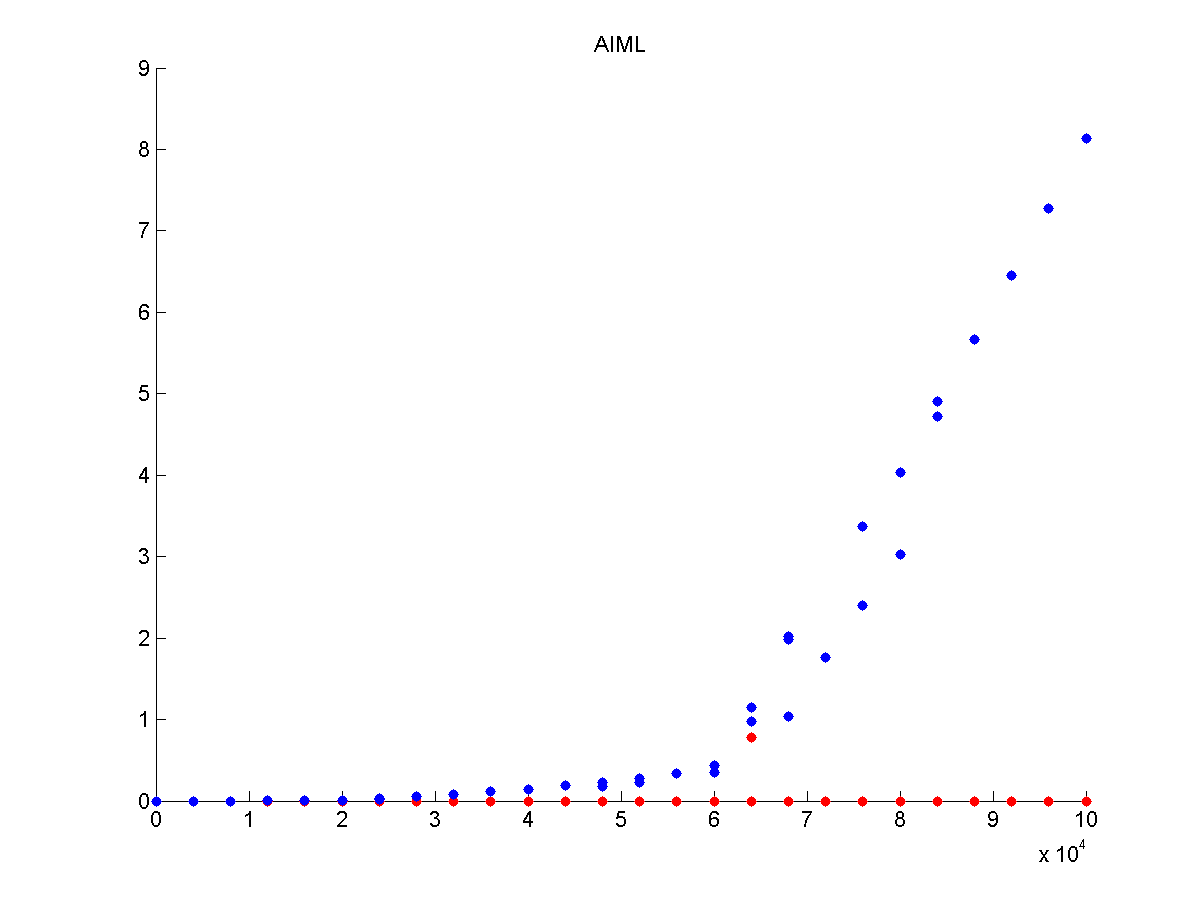

Supplement: Supplementary file 2 [file Presentation2.ZIP › AIML.png]

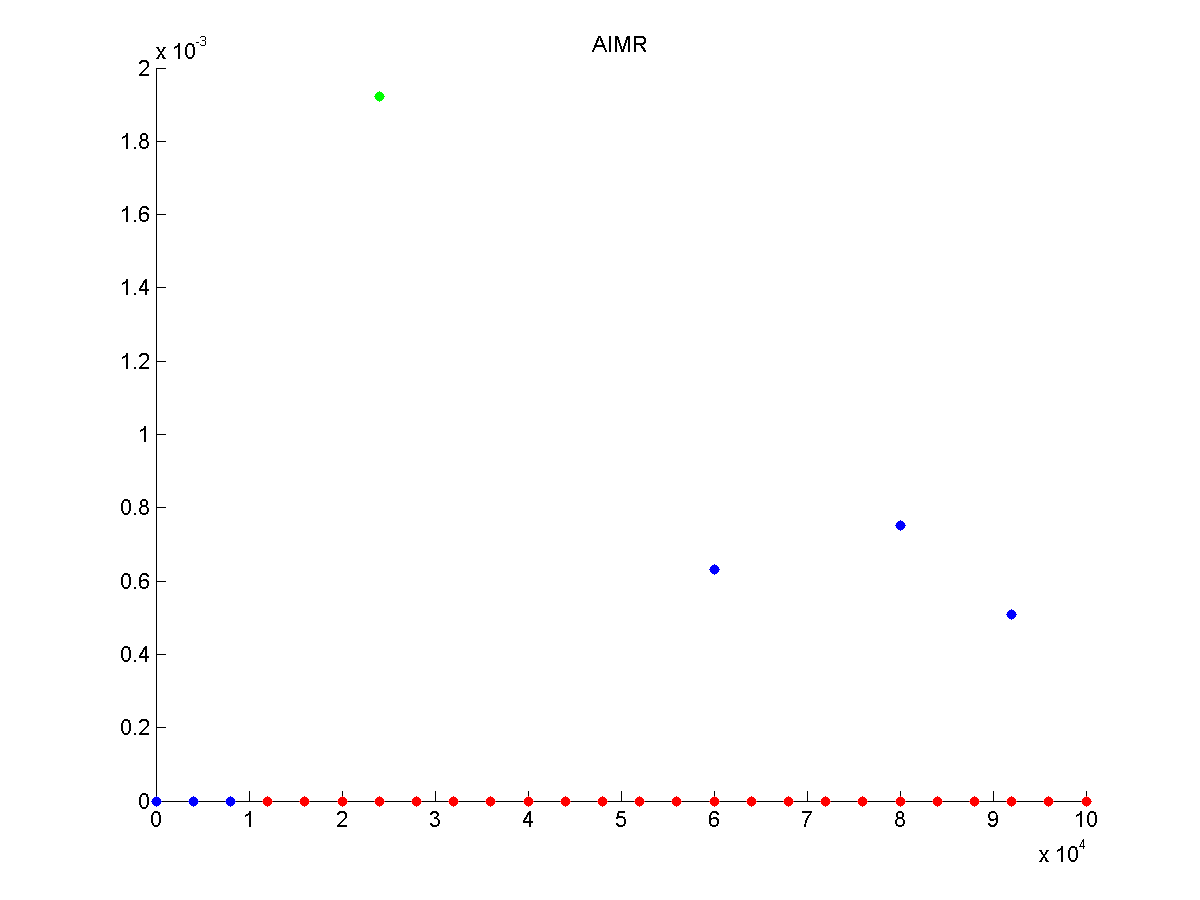

Supplement: Supplementary file 2 [file Presentation2.ZIP › AIMR.png]

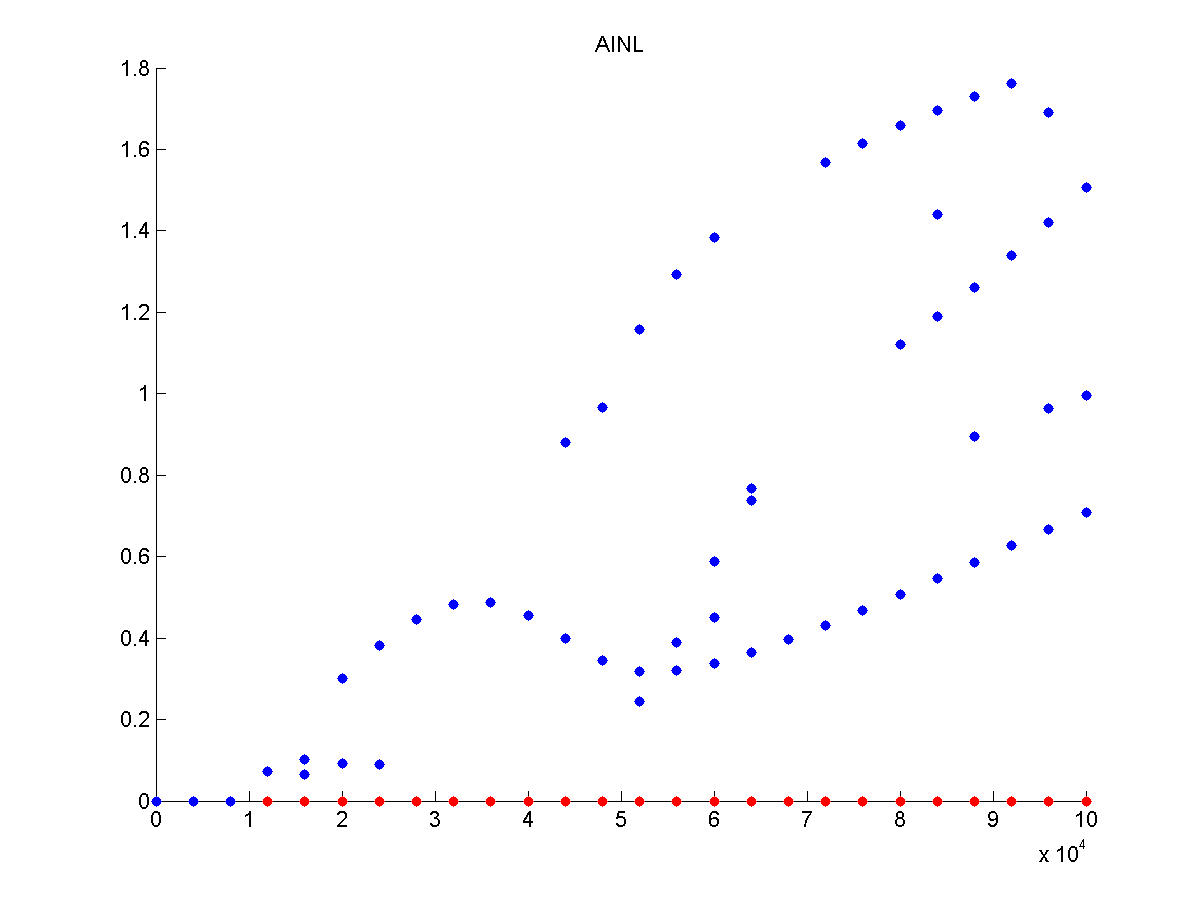

Supplement: Supplementary file 2 [file Presentation2.ZIP › AINL.png]

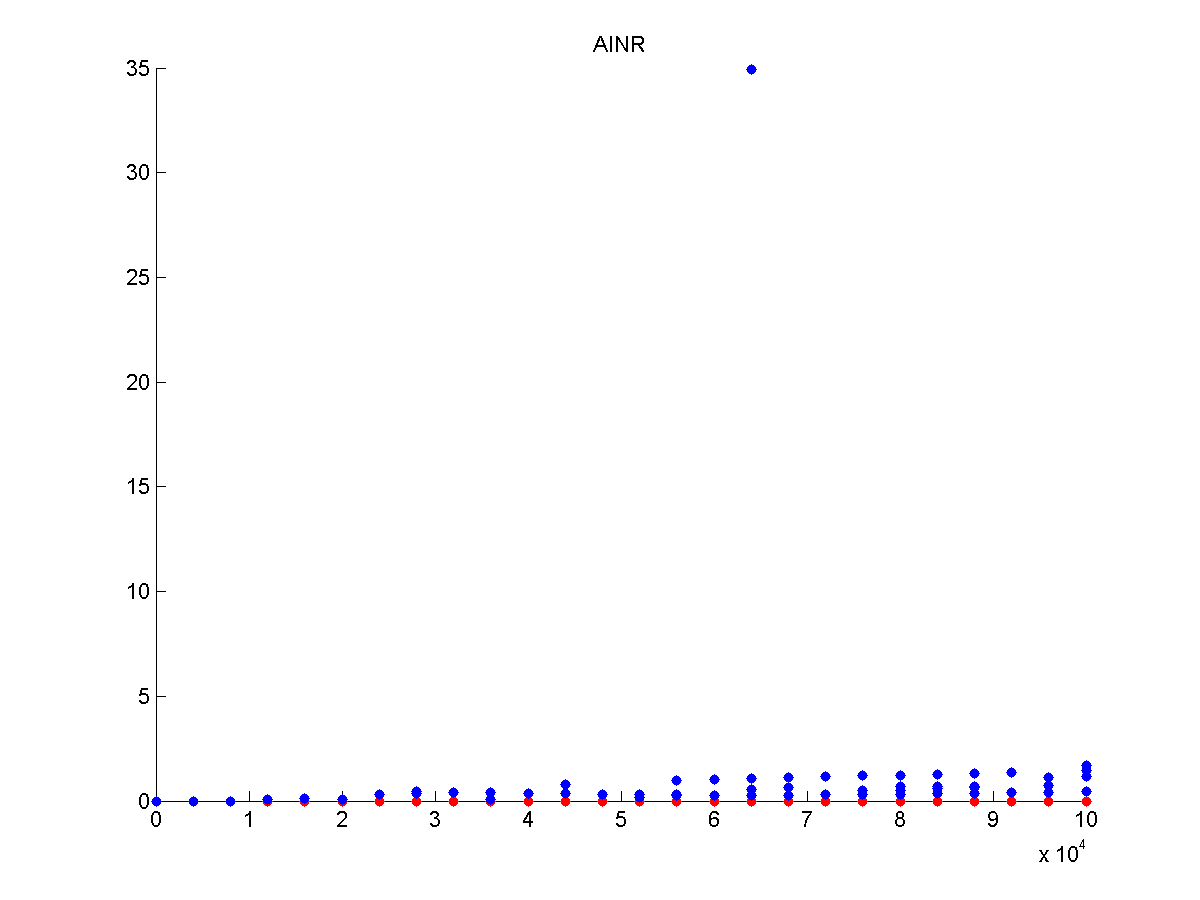

Supplement: Supplementary file 2 [file Presentation2.ZIP › AINR.png]

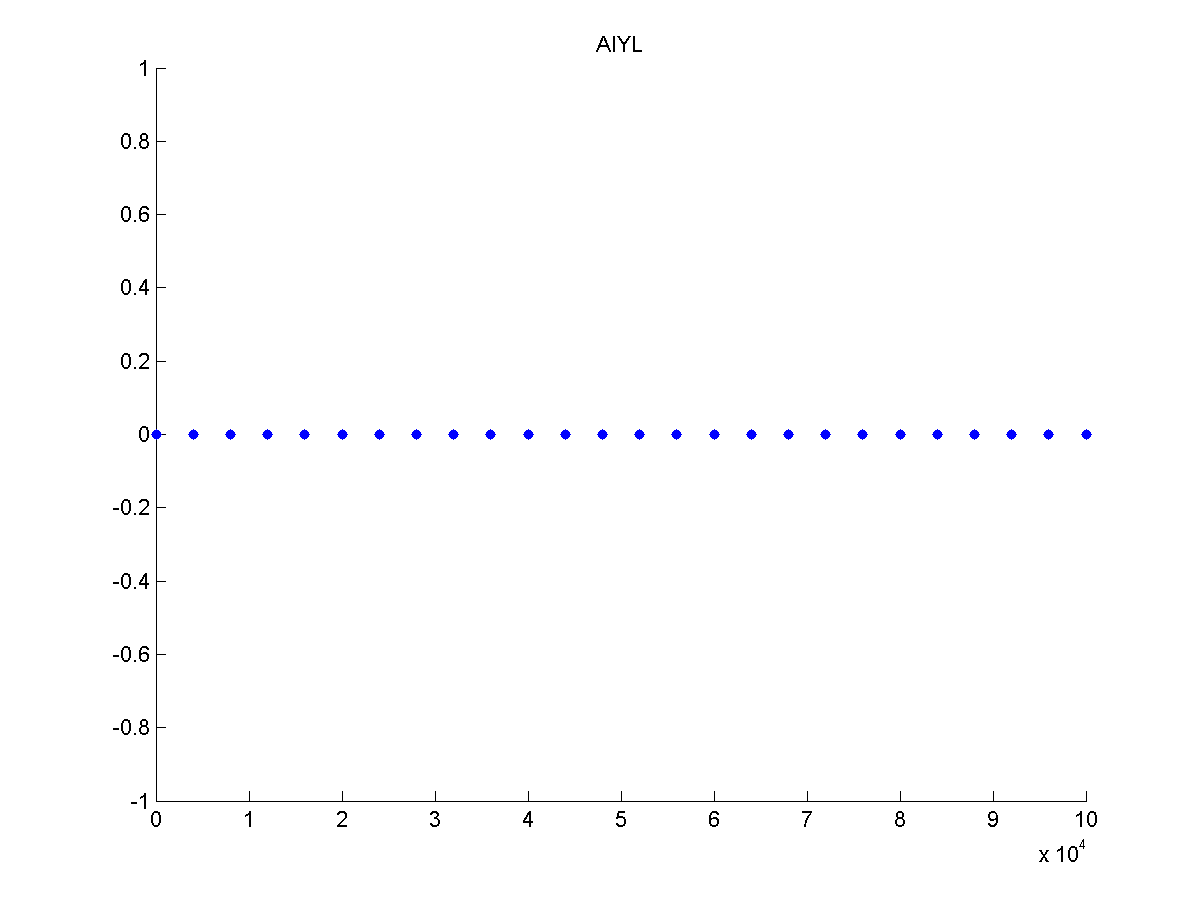

Supplement: Supplementary file 2 [file Presentation2.ZIP › AIYL.png]

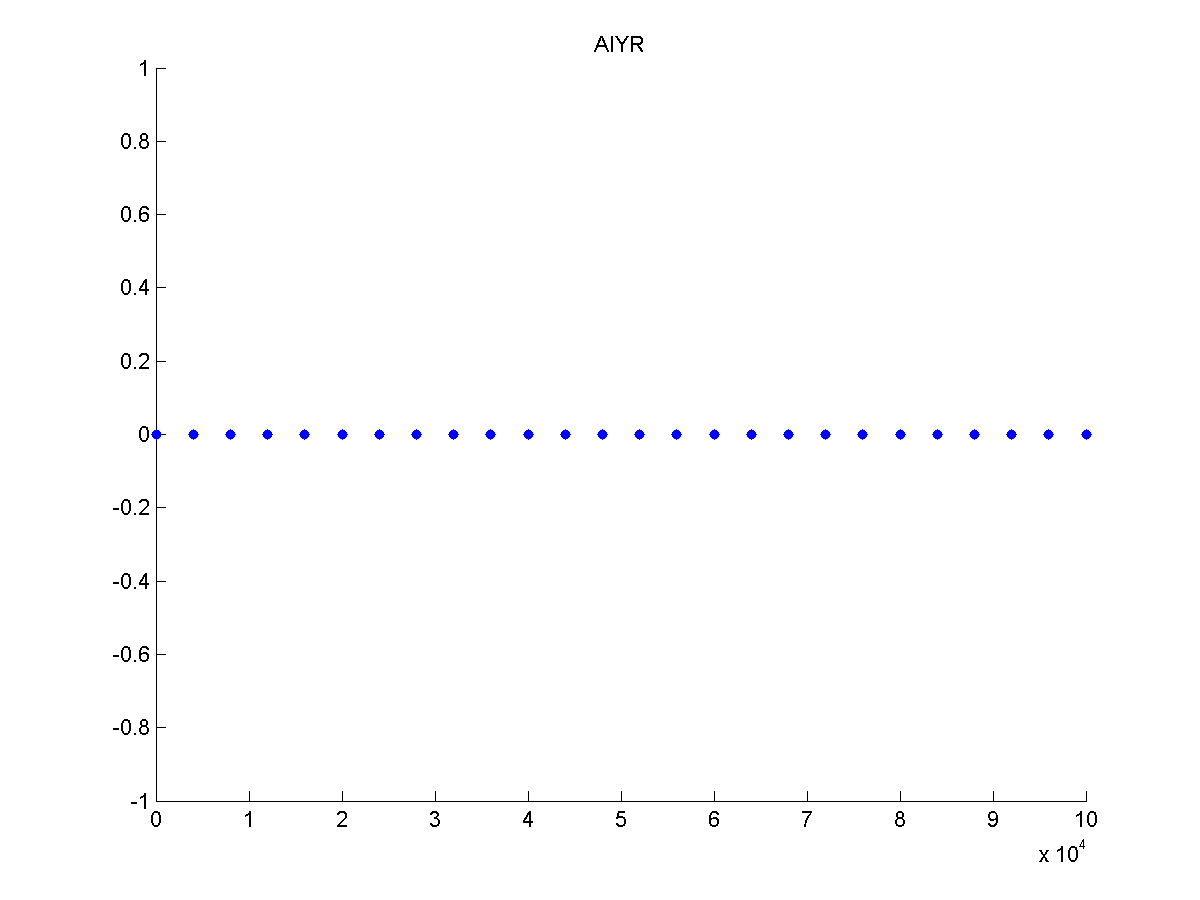

Supplement: Supplementary file 2 [file Presentation2.ZIP › AIYR.png]

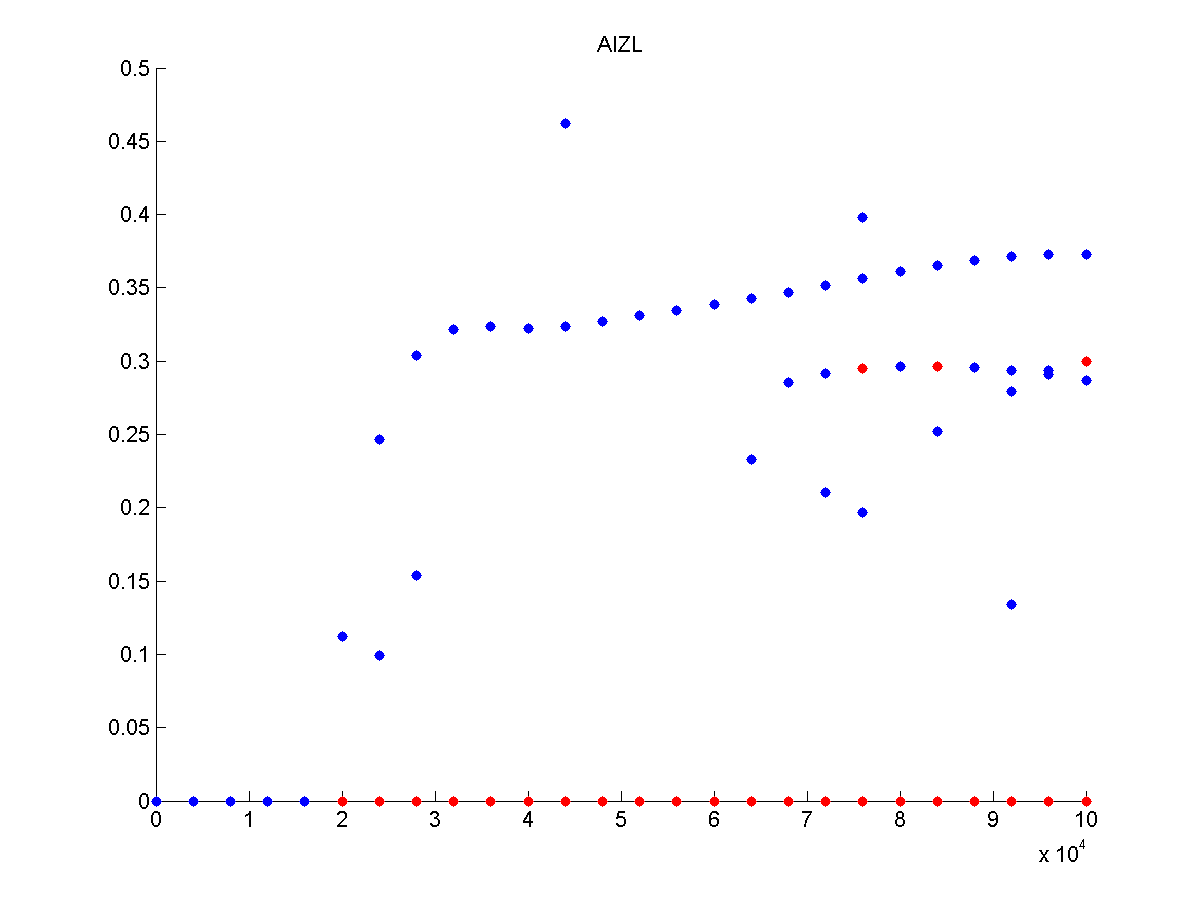

Supplement: Supplementary file 2 [file Presentation2.ZIP › AIZL.png]

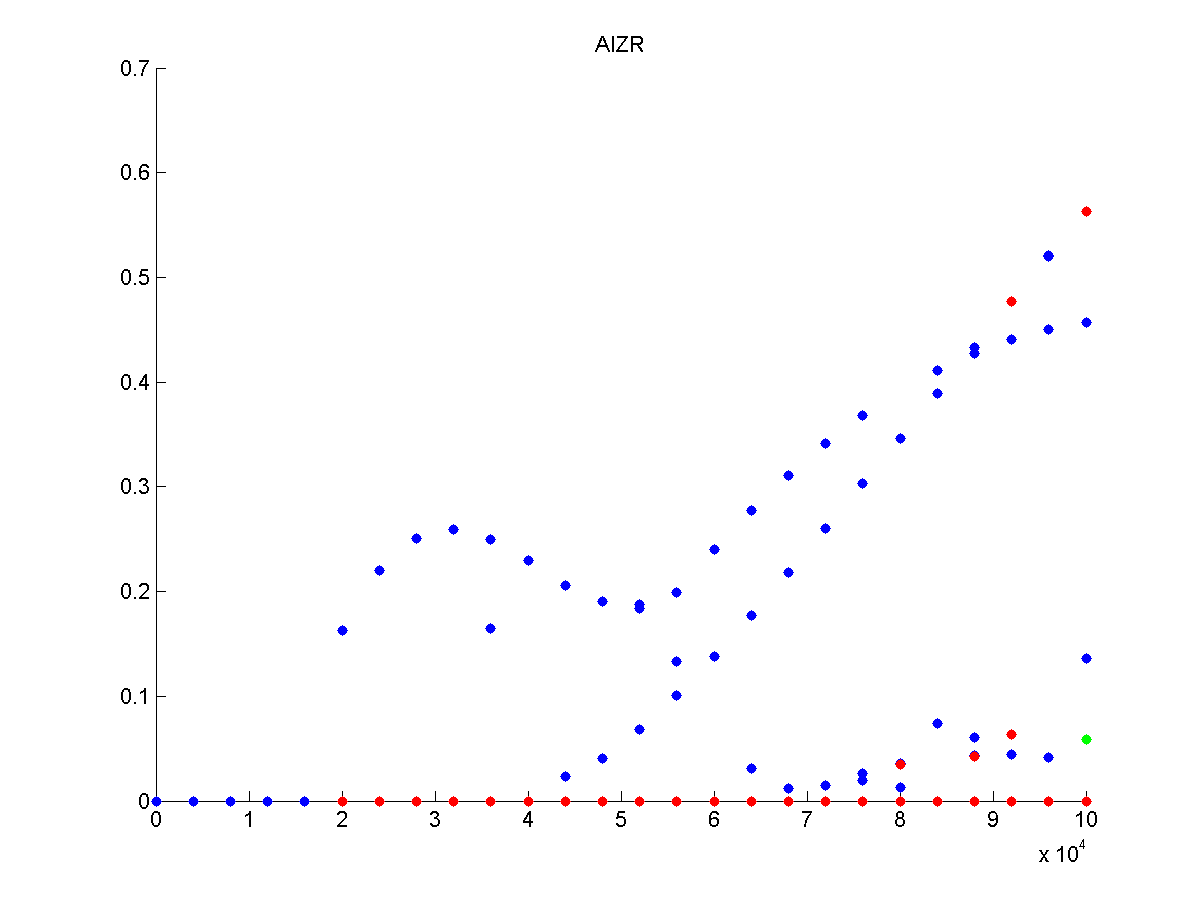

Supplement: Supplementary file 2 [file Presentation2.ZIP › AIZR.png]

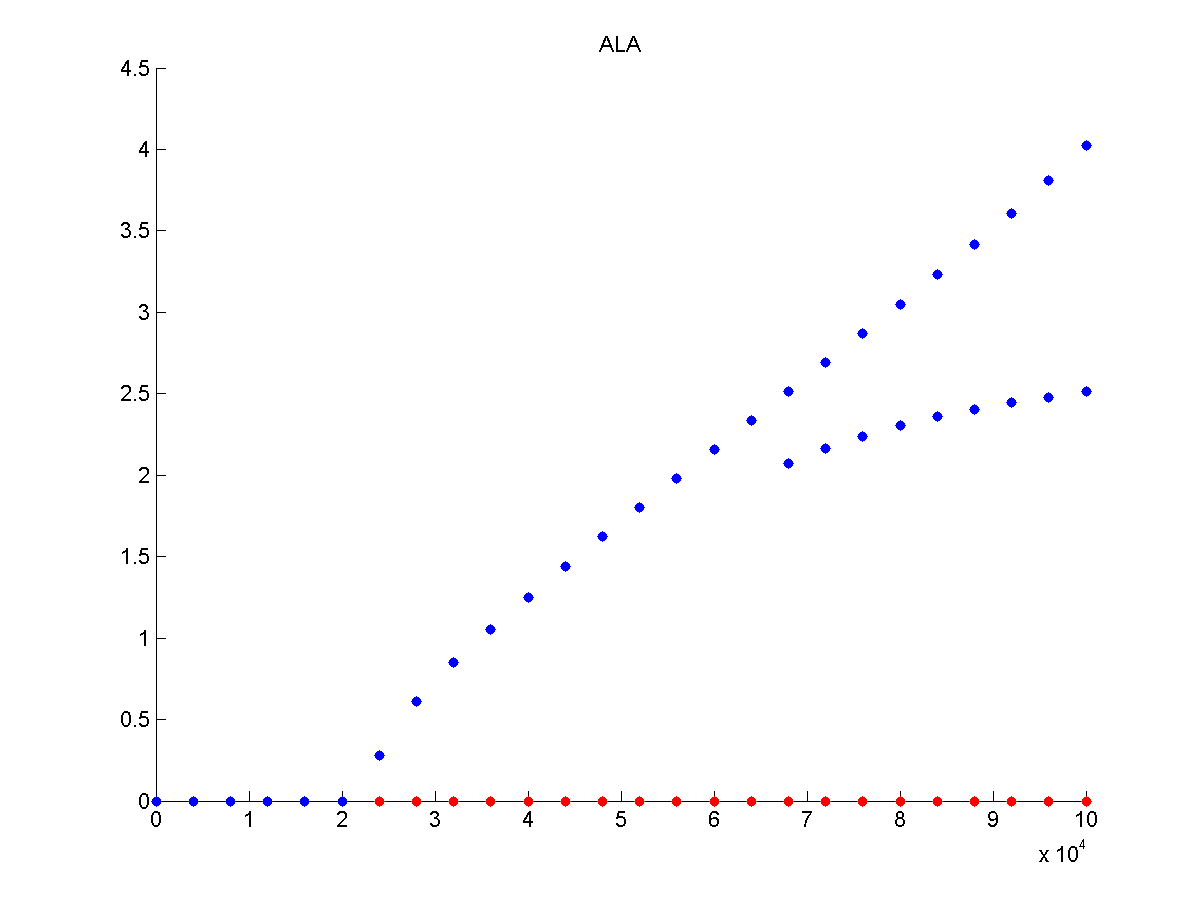

Supplement: Supplementary file 2 [file Presentation2.ZIP › ALA.png]

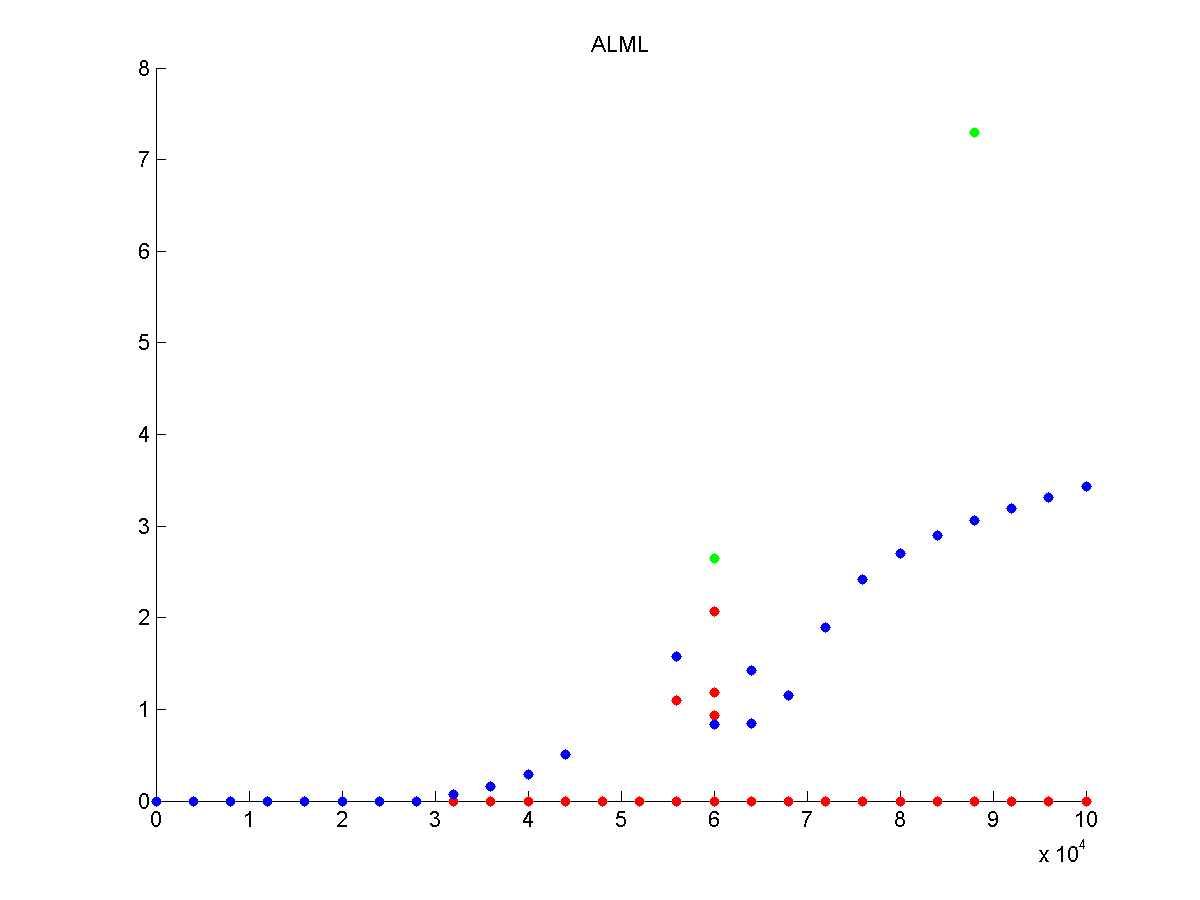

Supplement: Supplementary file 2 [file Presentation2.ZIP › ALML.png]

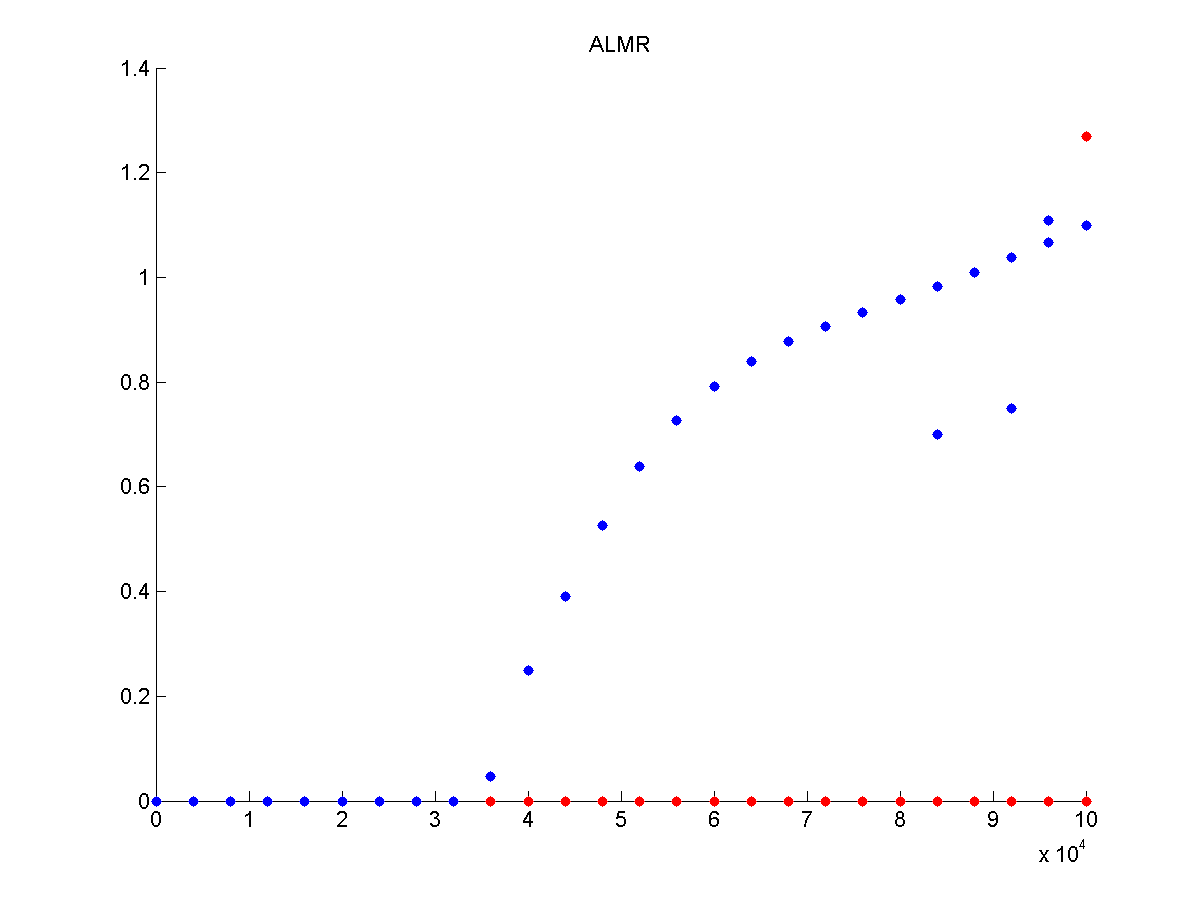

Supplement: Supplementary file 2 [file Presentation2.ZIP › ALMR.png]

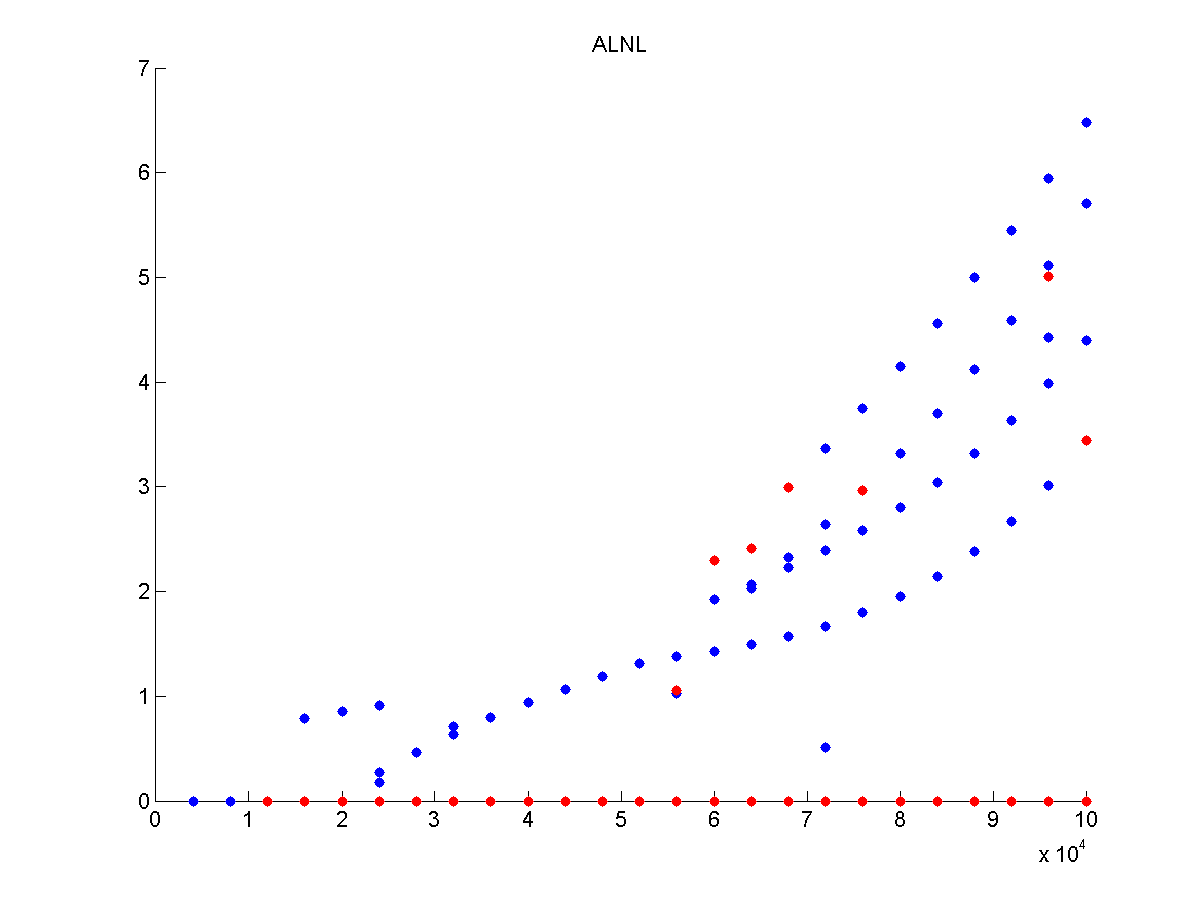

Supplement: Supplementary file 2 [file Presentation2.ZIP › ALNL.png]

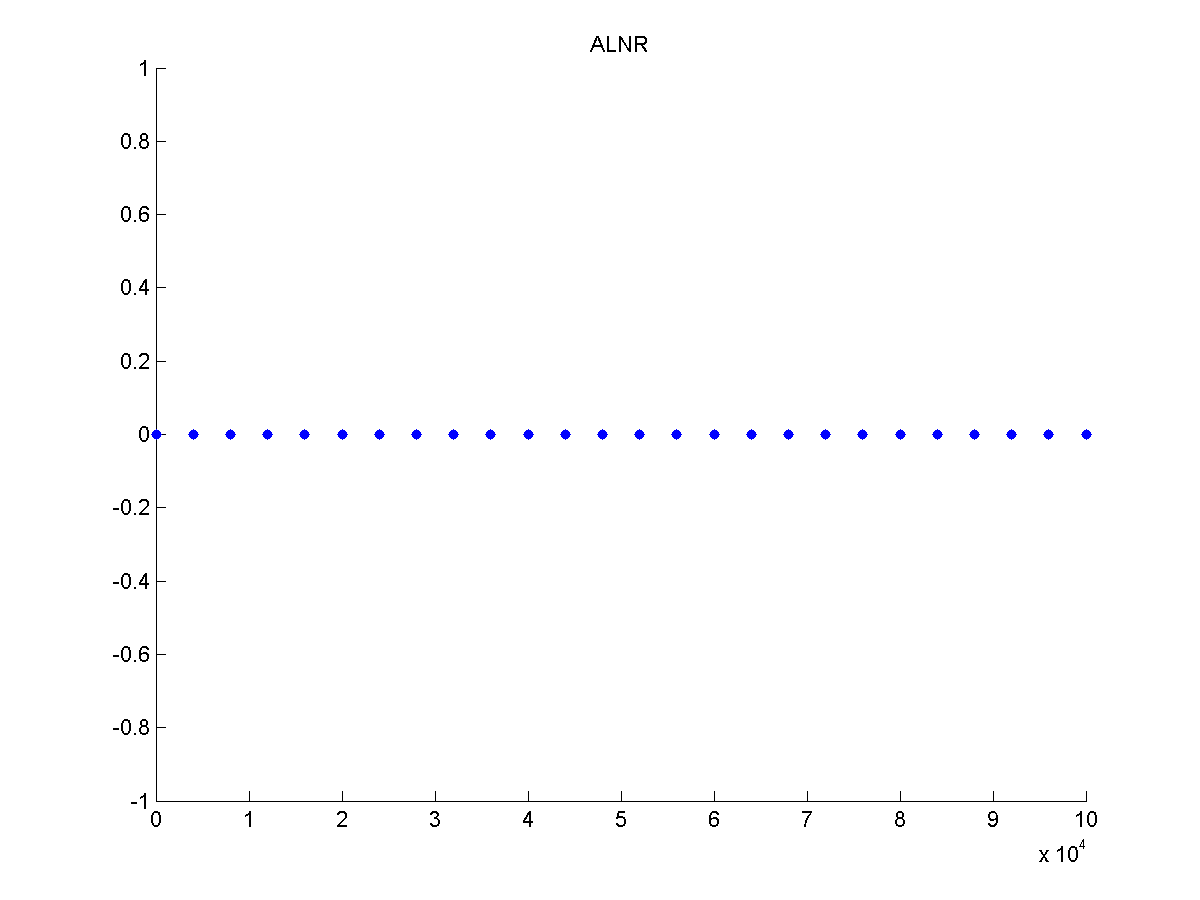

Supplement: Supplementary file 2 [file Presentation2.ZIP › ALNR.png]

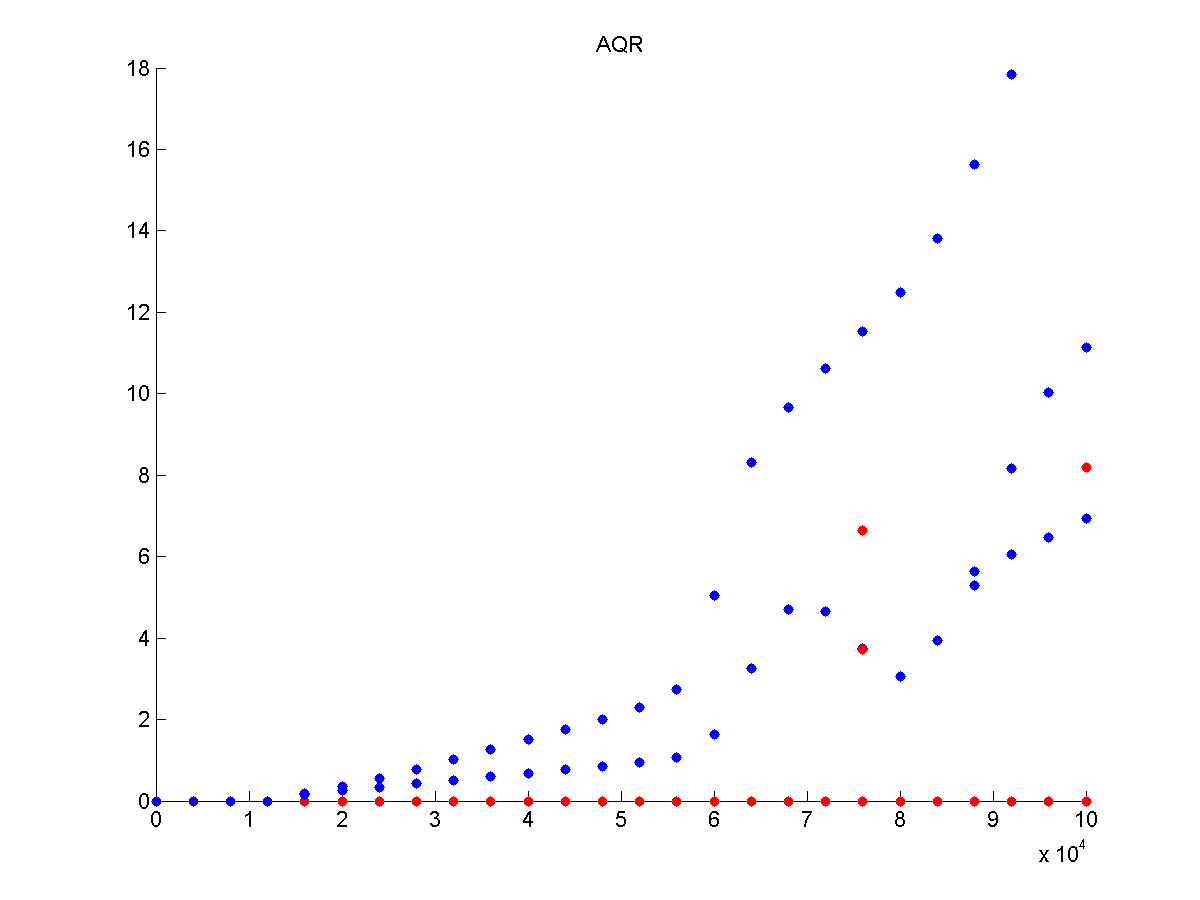

Supplement: Supplementary file 2 [file Presentation2.ZIP › AQR.png]

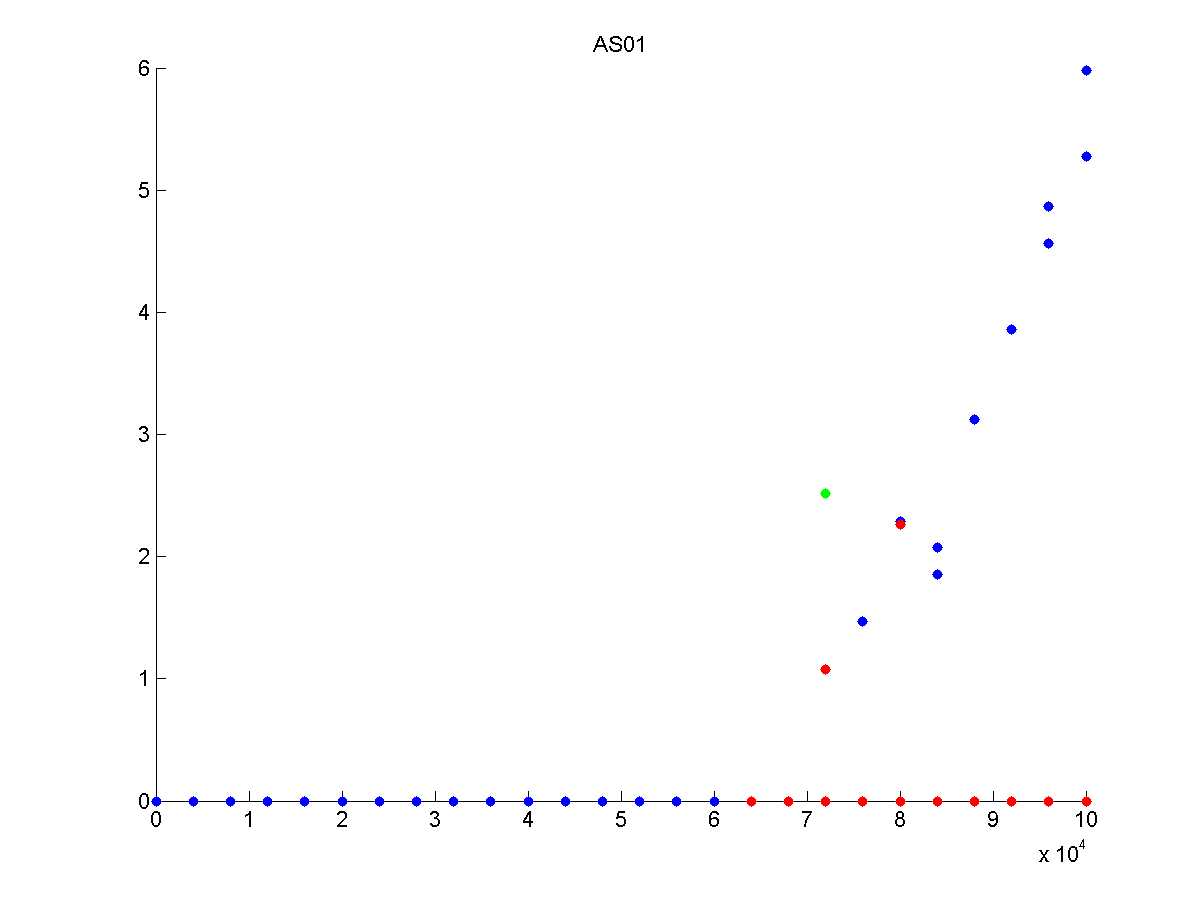

Supplement: Supplementary file 2 [file Presentation2.ZIP › AS01.png]

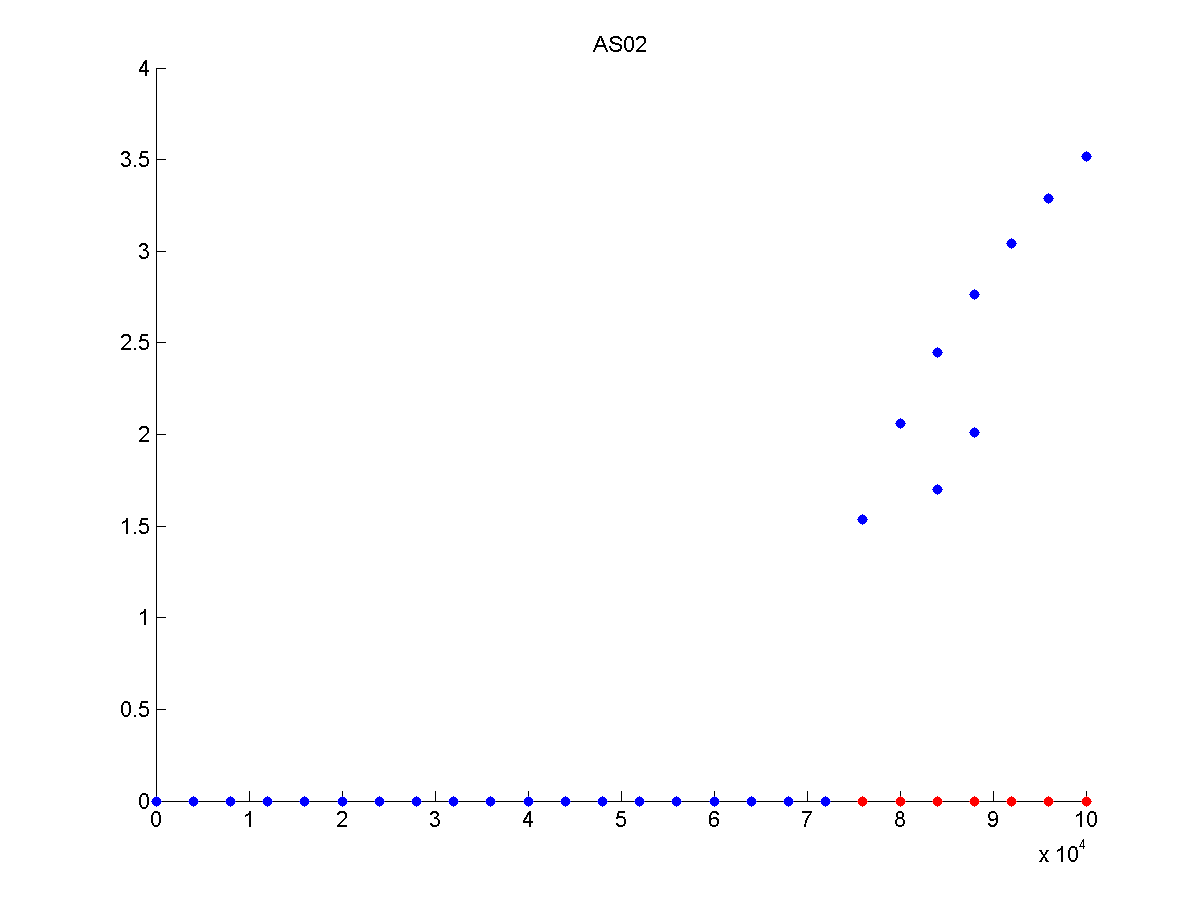

Supplement: Supplementary file 2 [file Presentation2.ZIP › AS02.png]

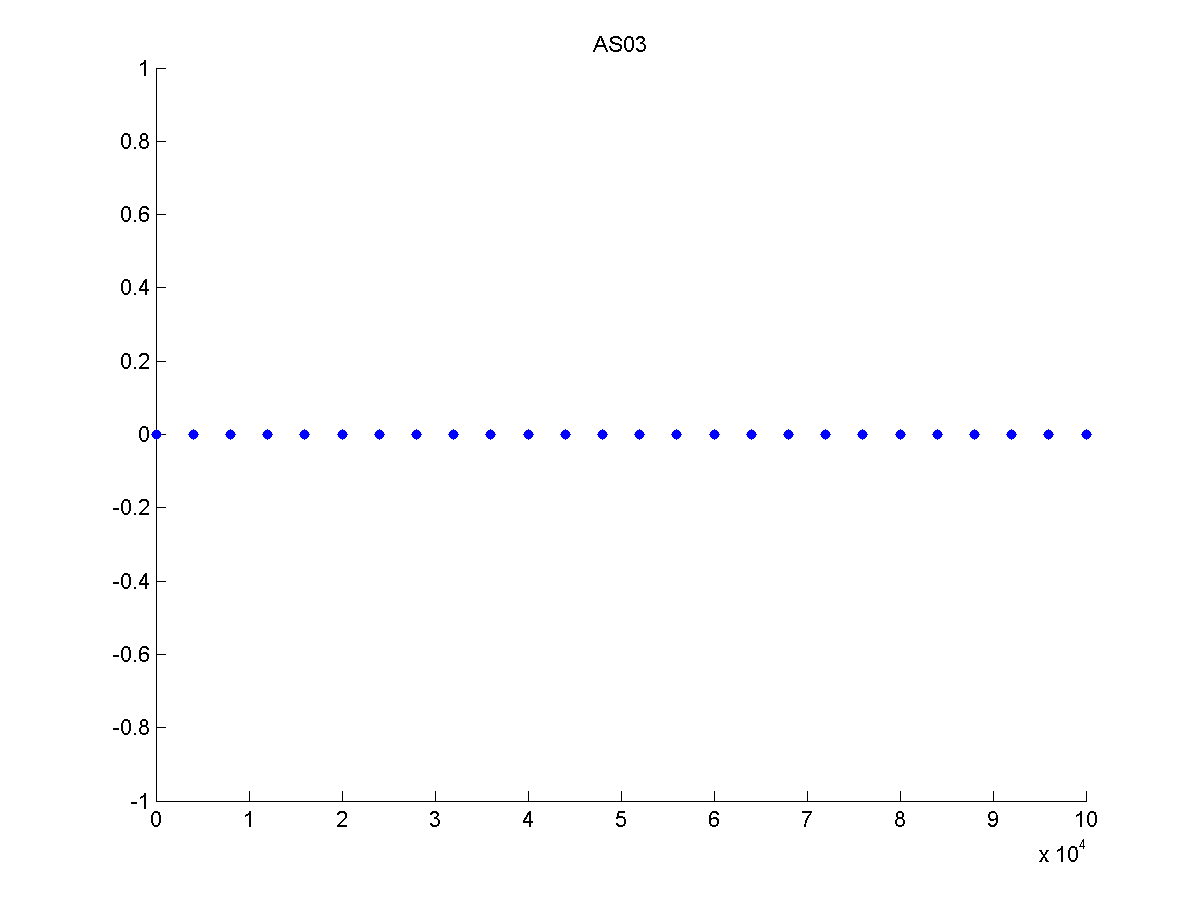

Supplement: Supplementary file 2 [file Presentation2.ZIP › AS03.png]

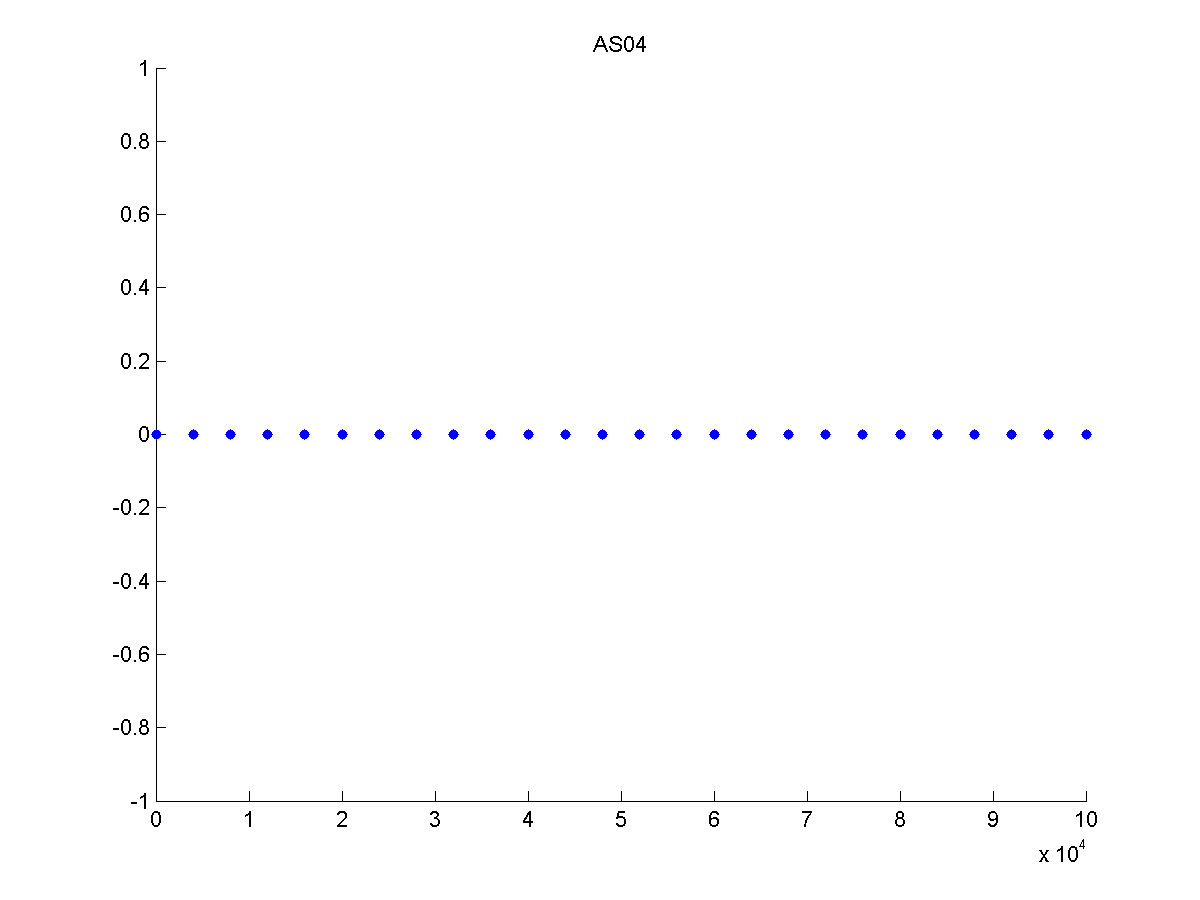

Supplement: Supplementary file 2 [file Presentation2.ZIP › AS04.png]

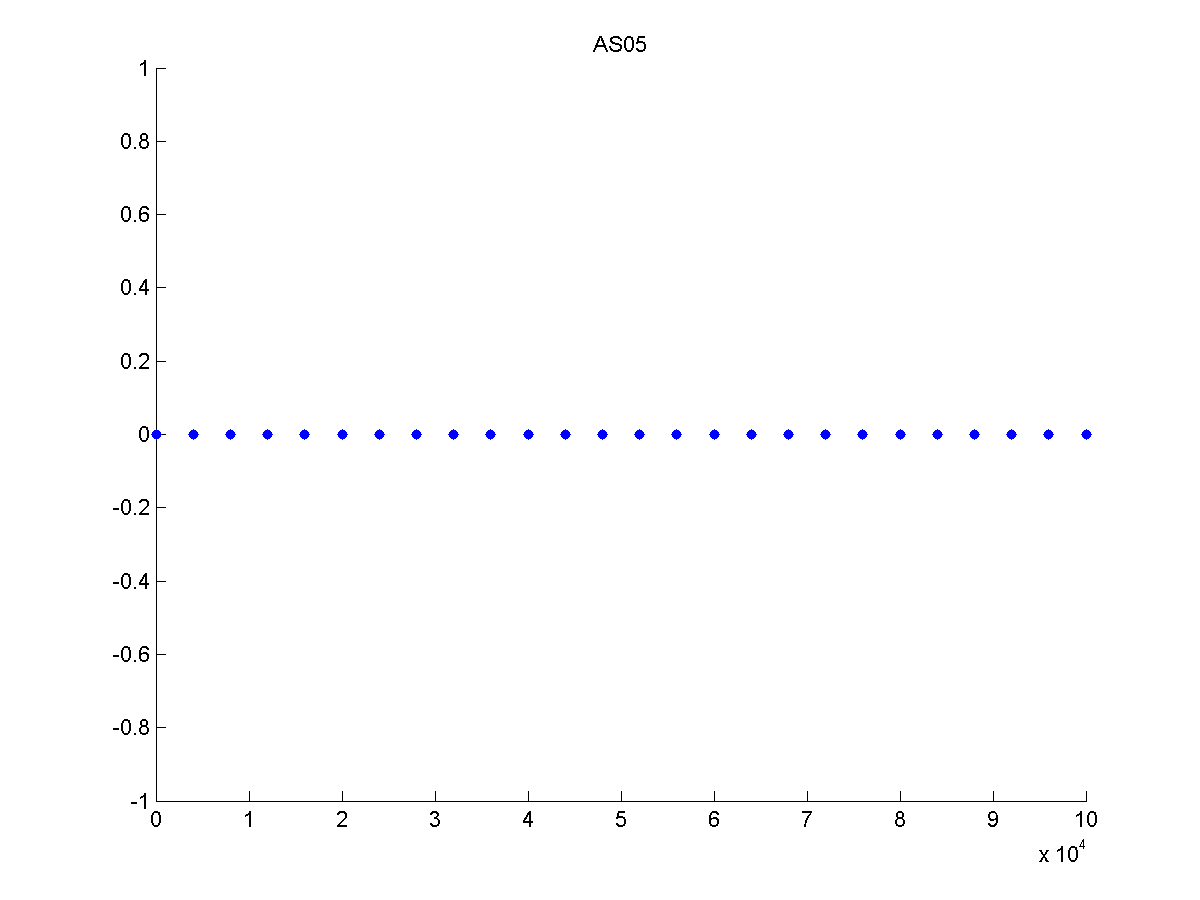

Supplement: Supplementary file 2 [file Presentation2.ZIP › AS05.png]

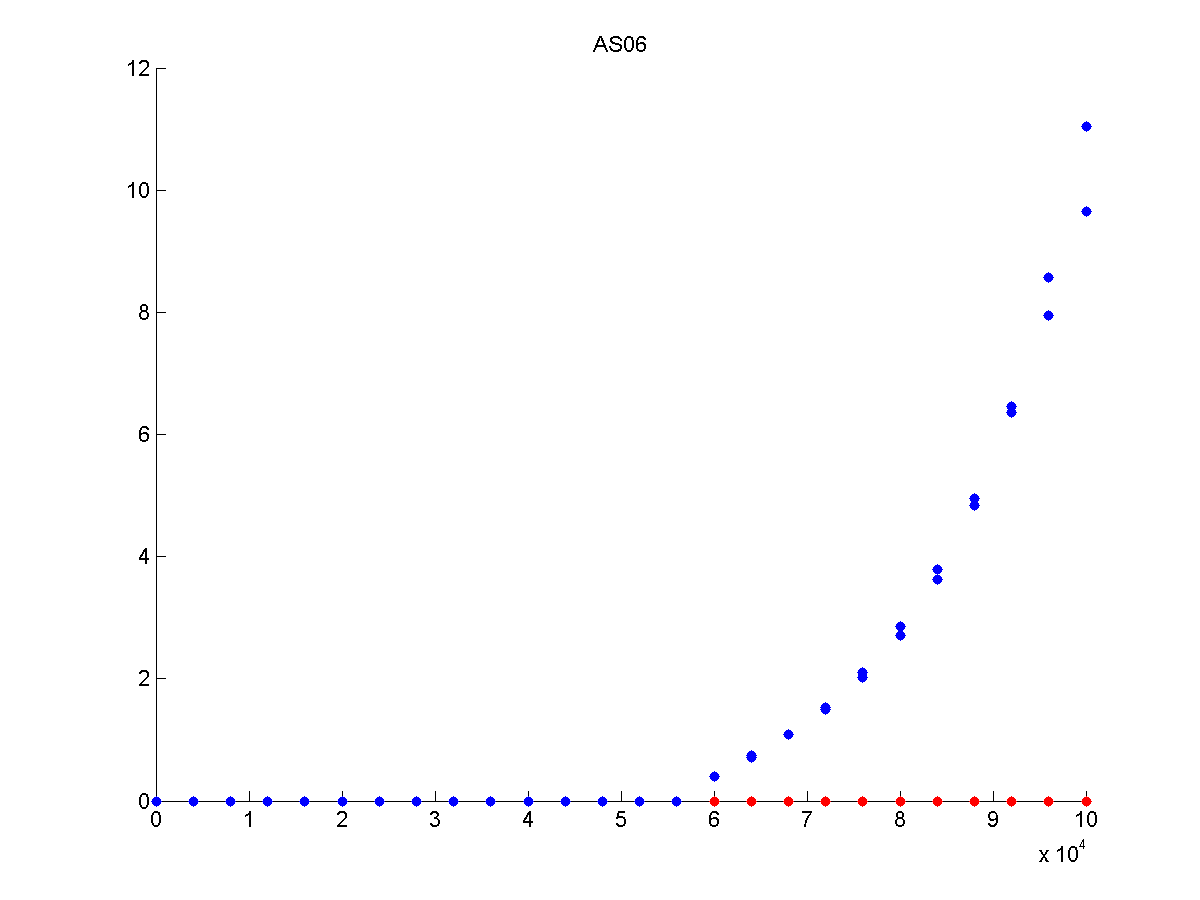

Supplement: Supplementary file 2 [file Presentation2.ZIP › AS06.png]

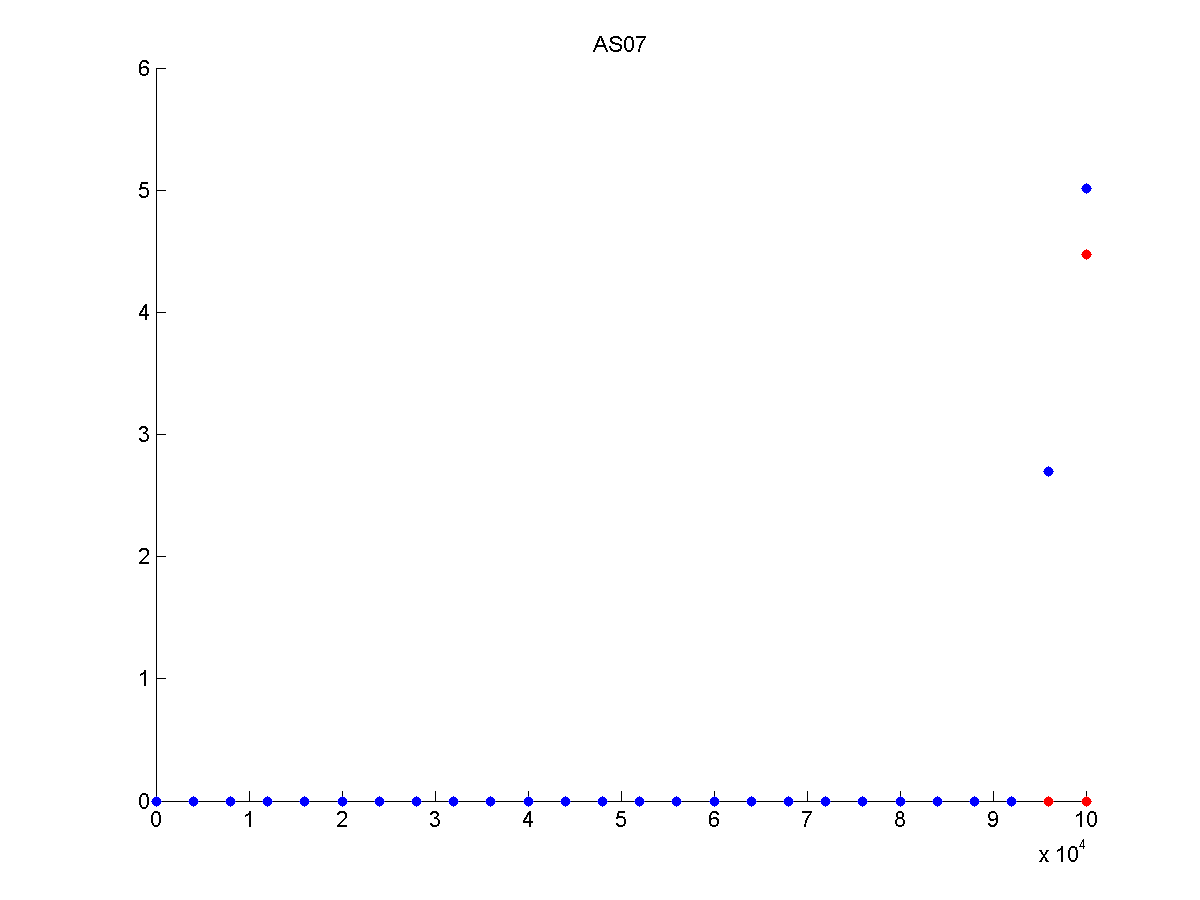

Supplement: Supplementary file 2 [file Presentation2.ZIP › AS07.png]

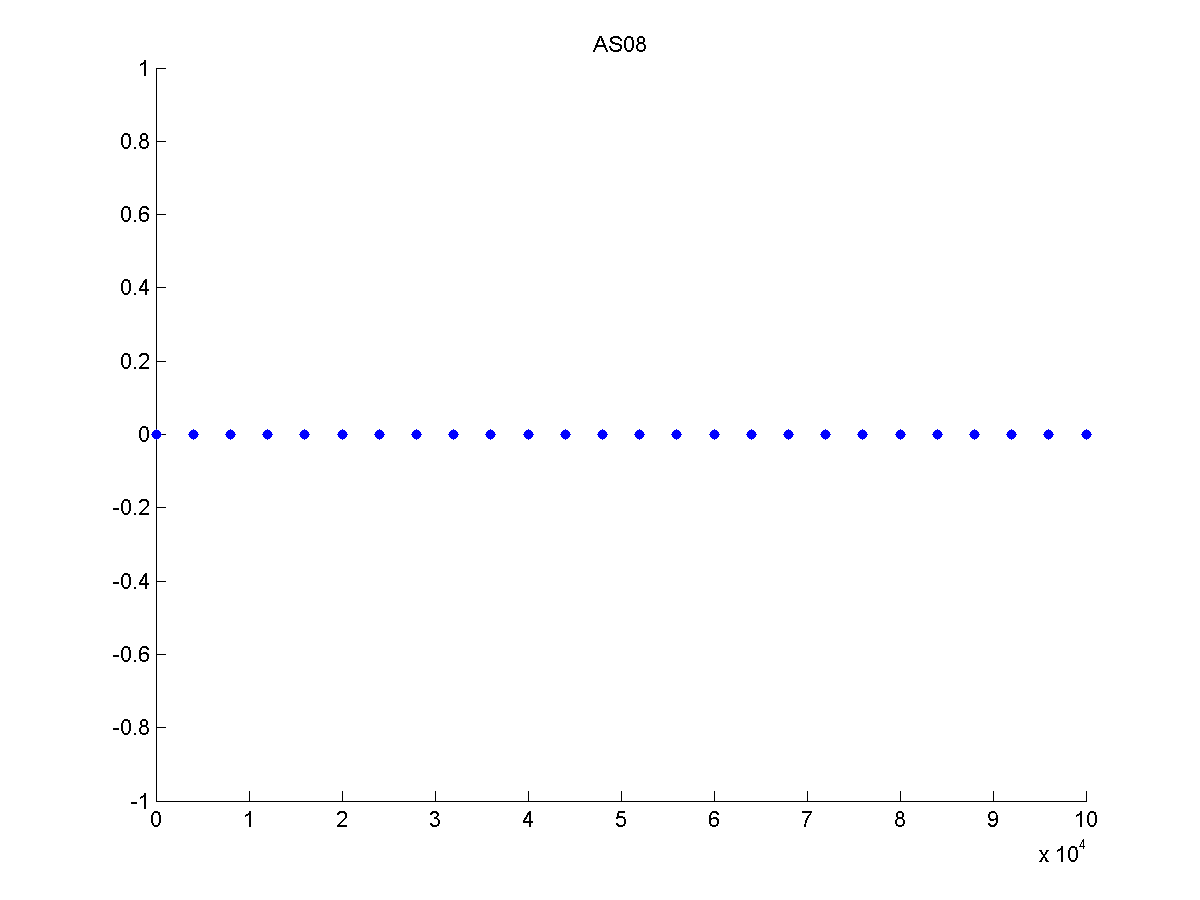

Supplement: Supplementary file 2 [file Presentation2.ZIP › AS08.png]

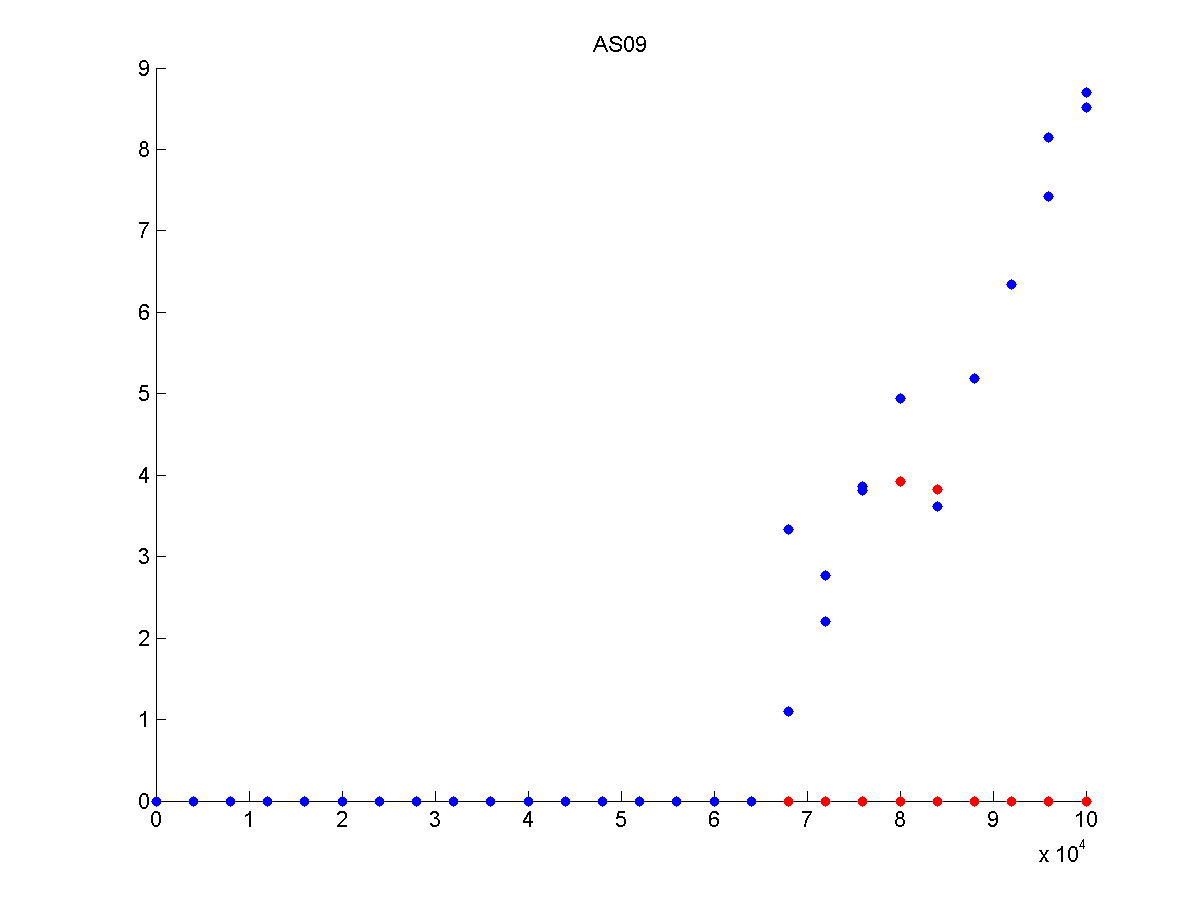

Supplement: Supplementary file 2 [file Presentation2.ZIP › AS09.png]

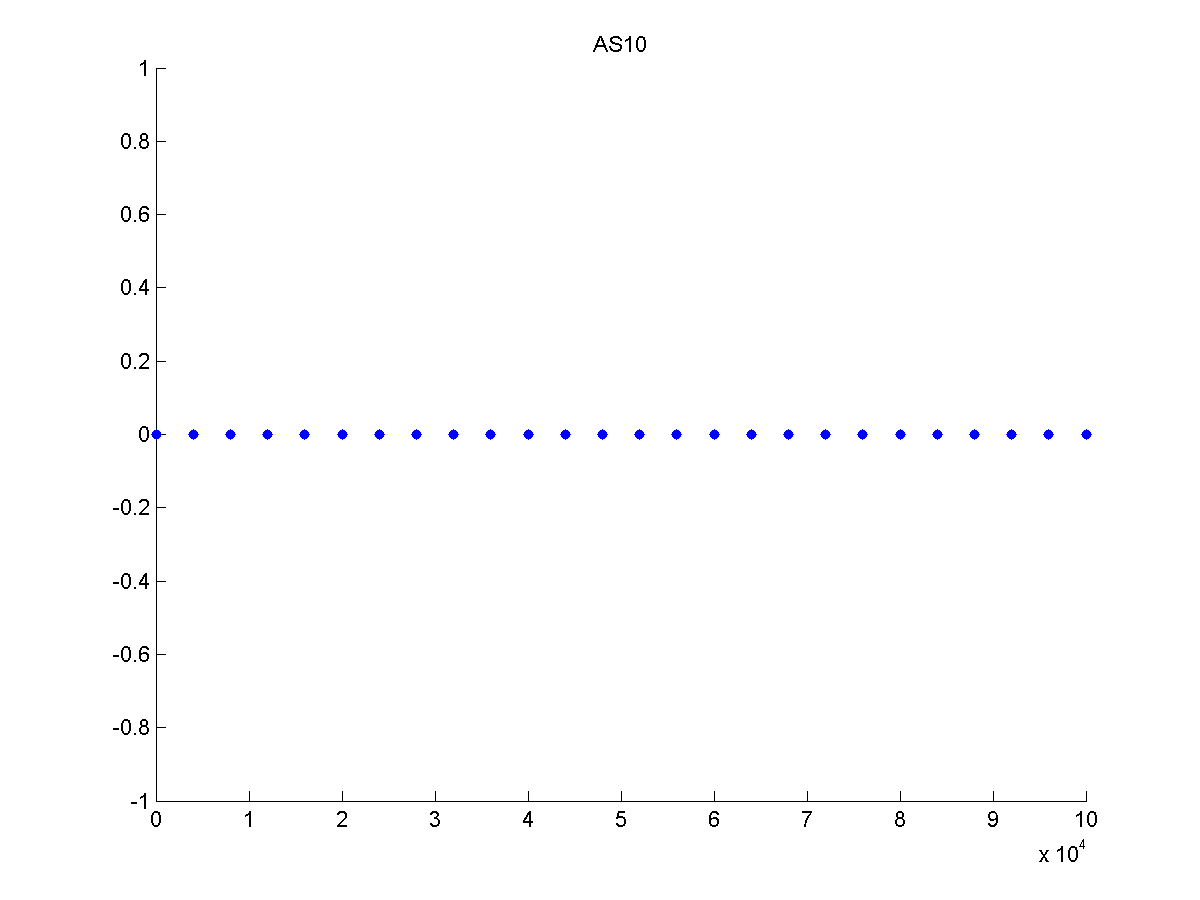

Supplement: Supplementary file 2 [file Presentation2.ZIP › AS10.png]

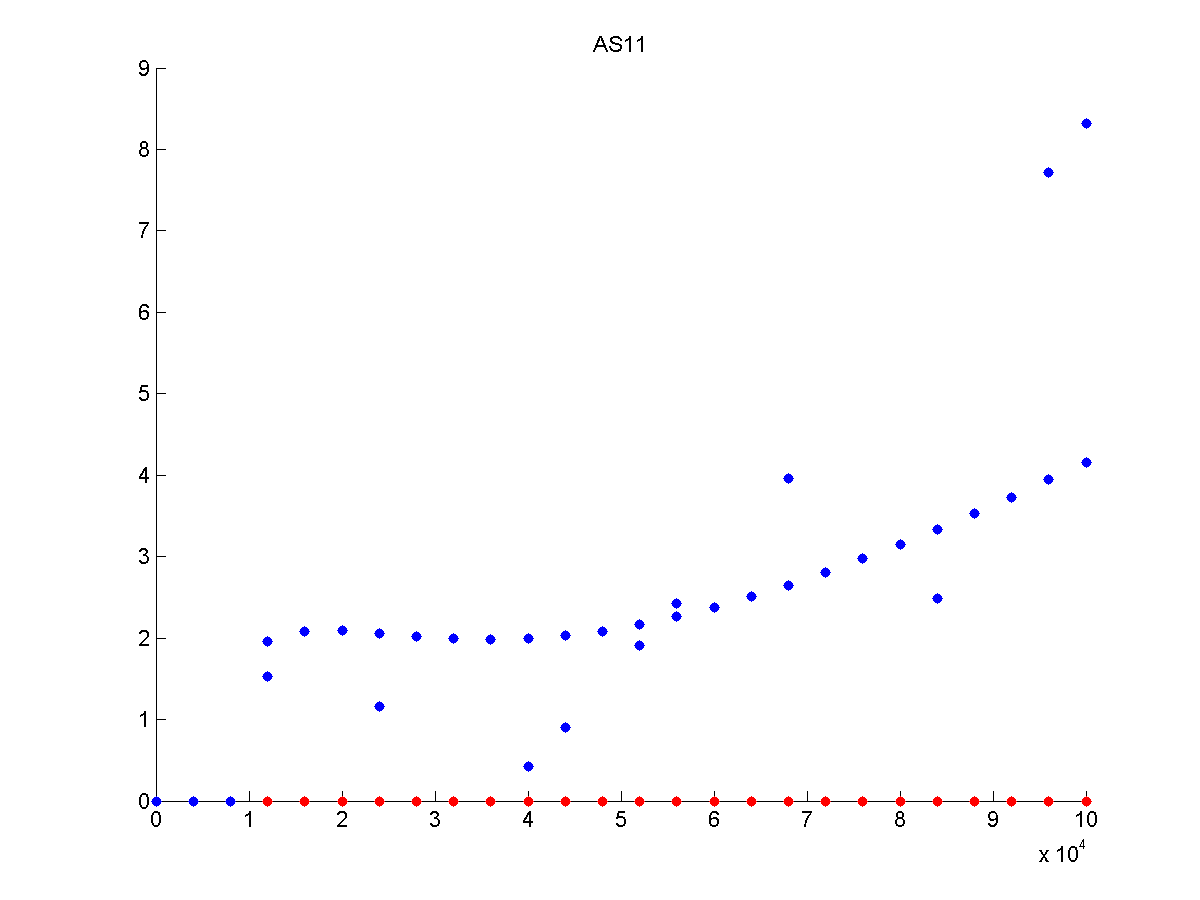

Supplement: Supplementary file 2 [file Presentation2.ZIP › AS11.png]

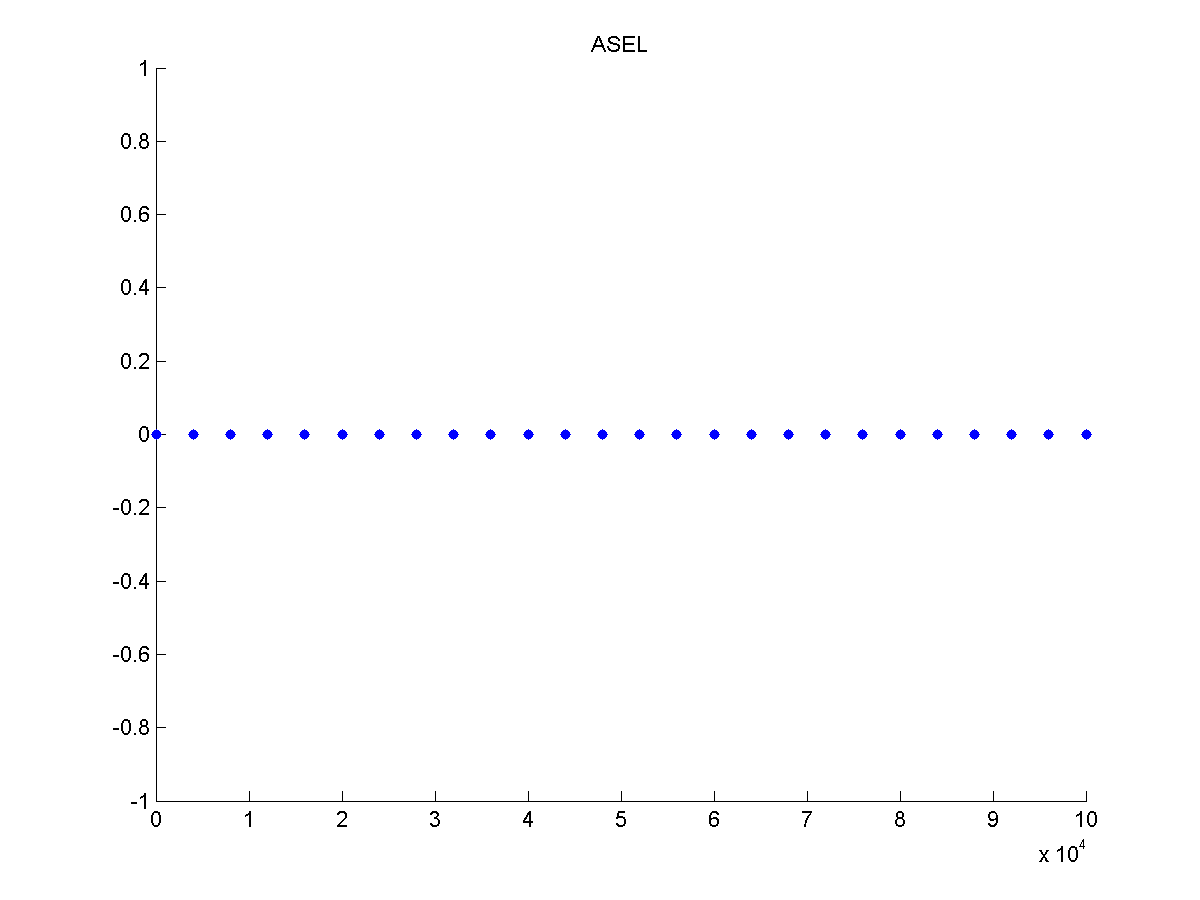

Supplement: Supplementary file 2 [file Presentation2.ZIP › ASEL.png]

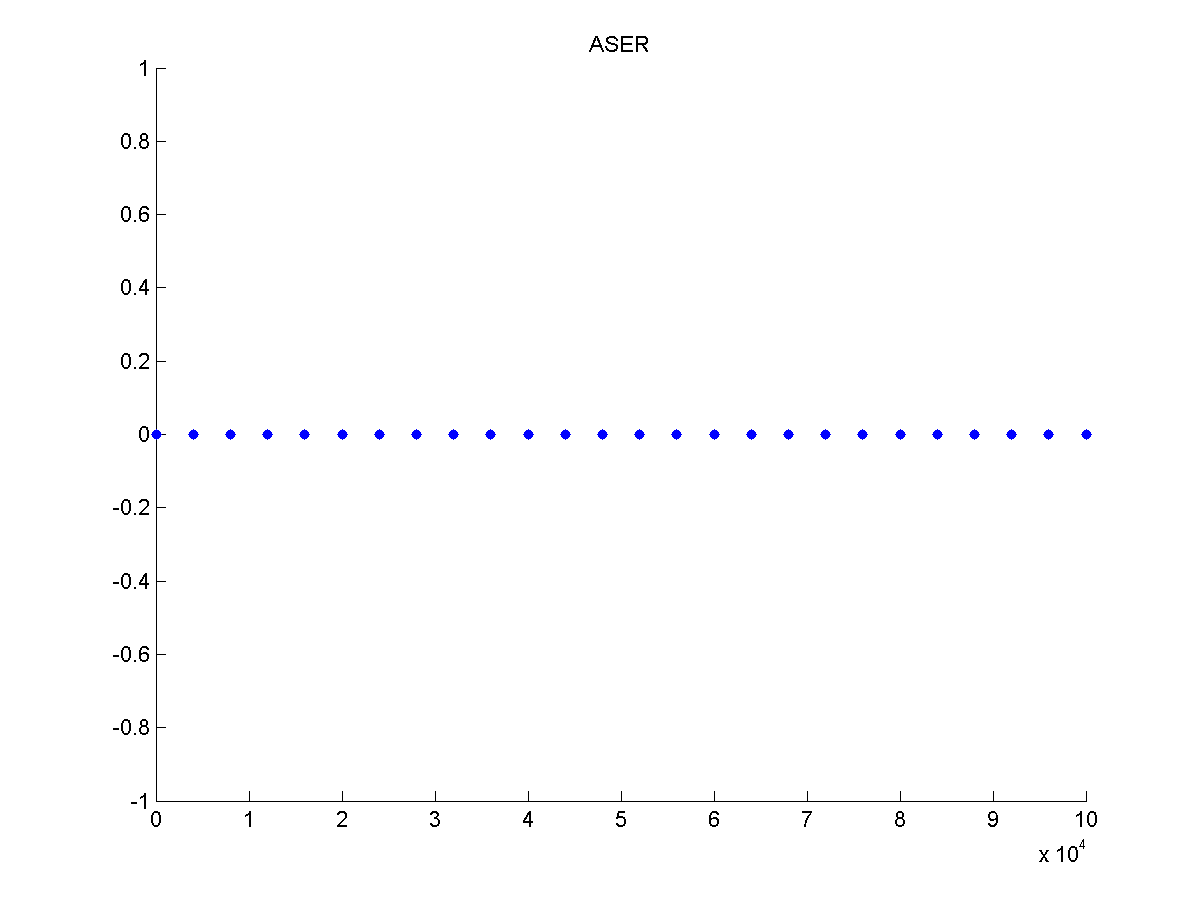

Supplement: Supplementary file 2 [file Presentation2.ZIP › ASER.png]

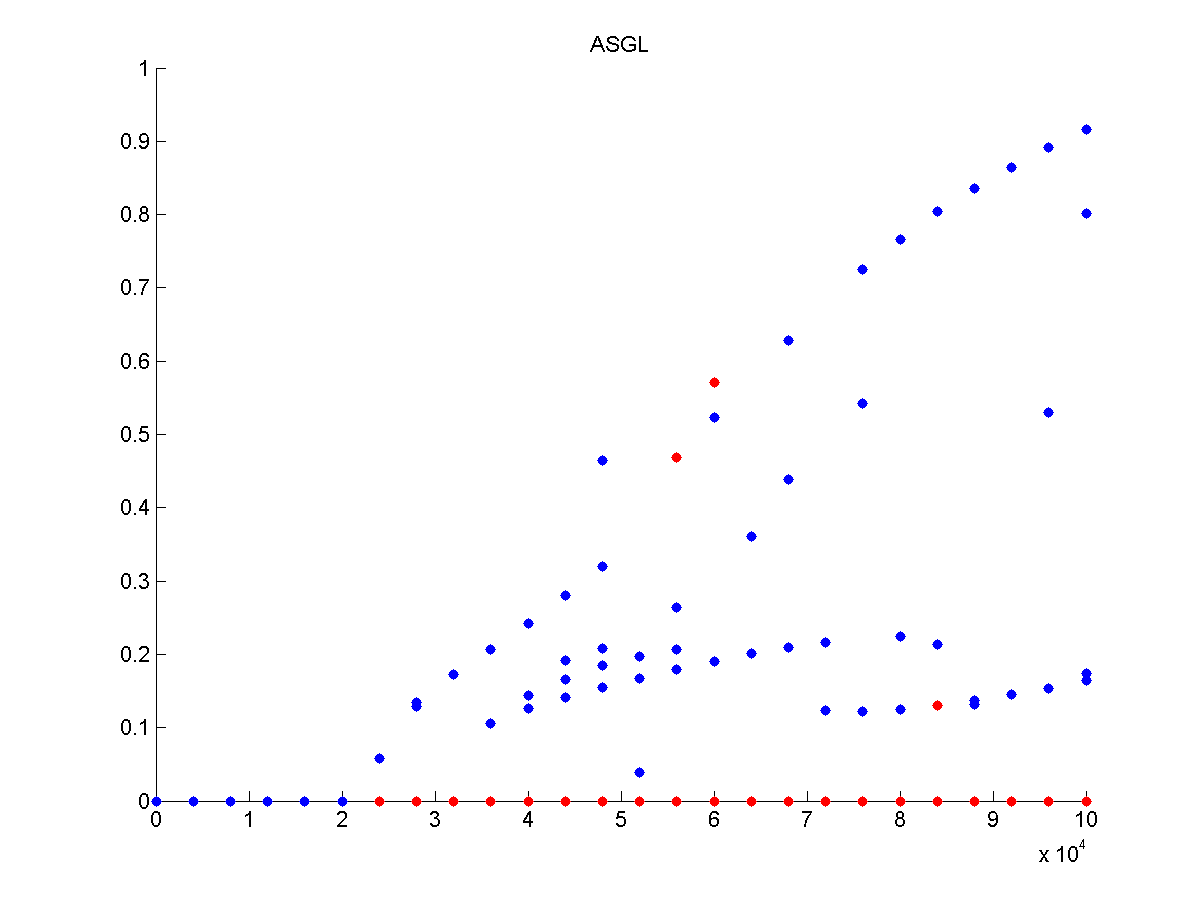

Supplement: Supplementary file 2 [file Presentation2.ZIP › ASGL.png]

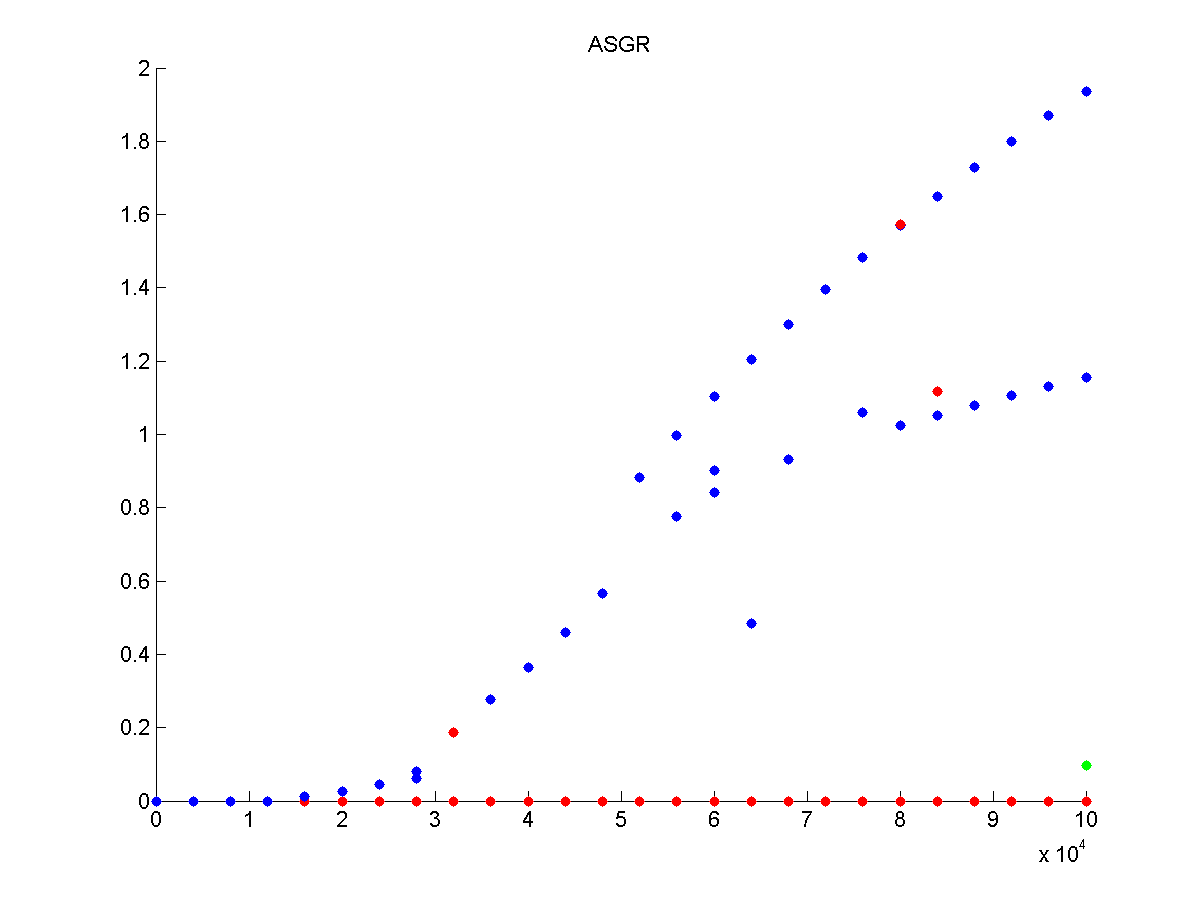

Supplement: Supplementary file 2 [file Presentation2.ZIP › ASGR.png]

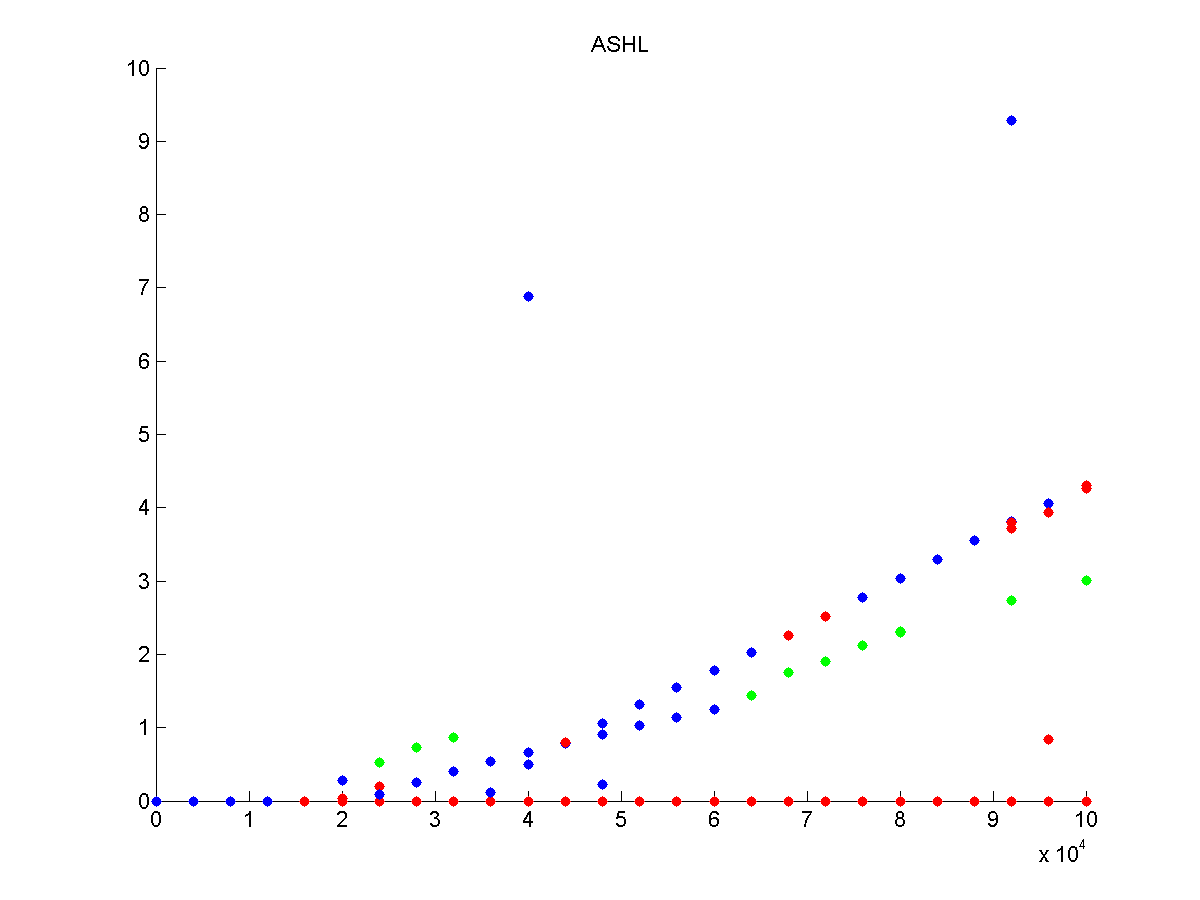

Supplement: Supplementary file 2 [file Presentation2.ZIP › ASHL.png]

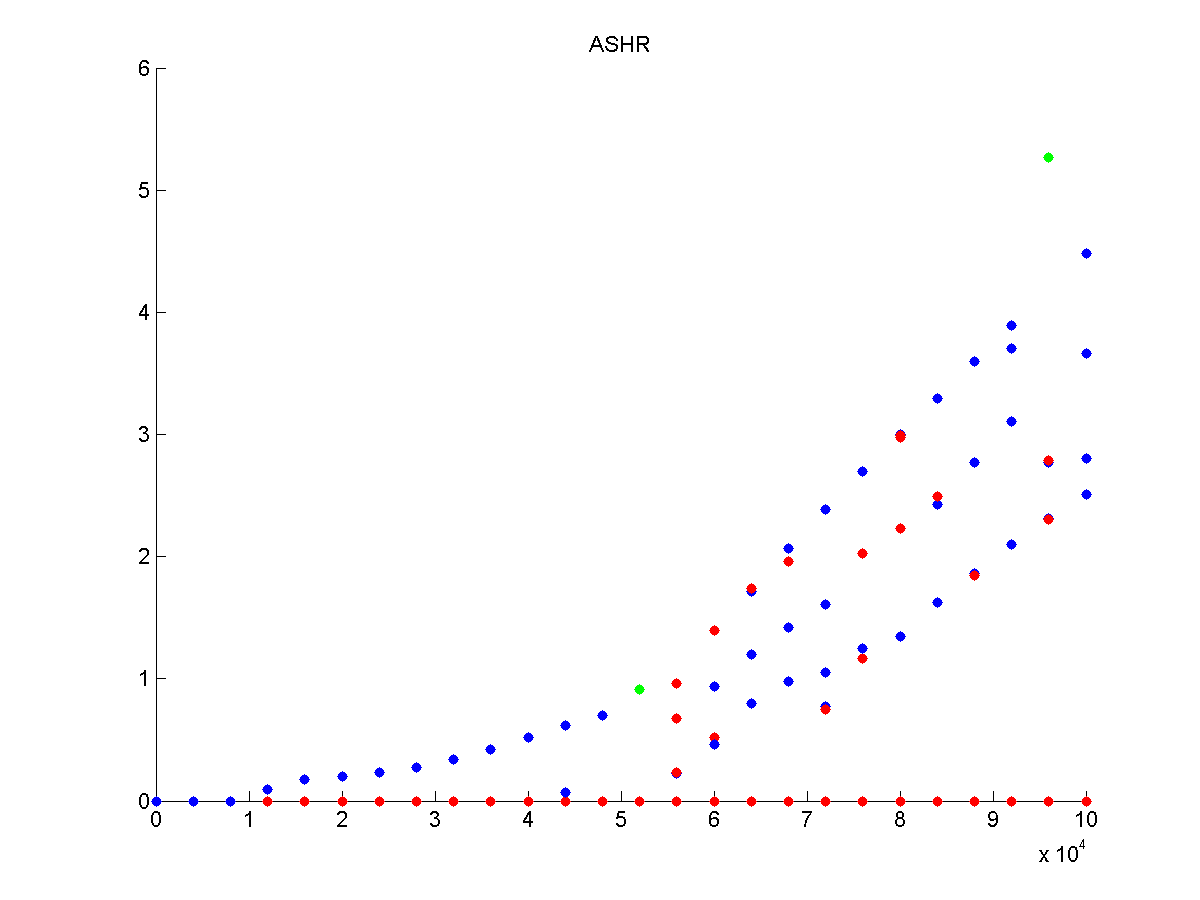

Supplement: Supplementary file 2 [file Presentation2.ZIP › ASHR.png]

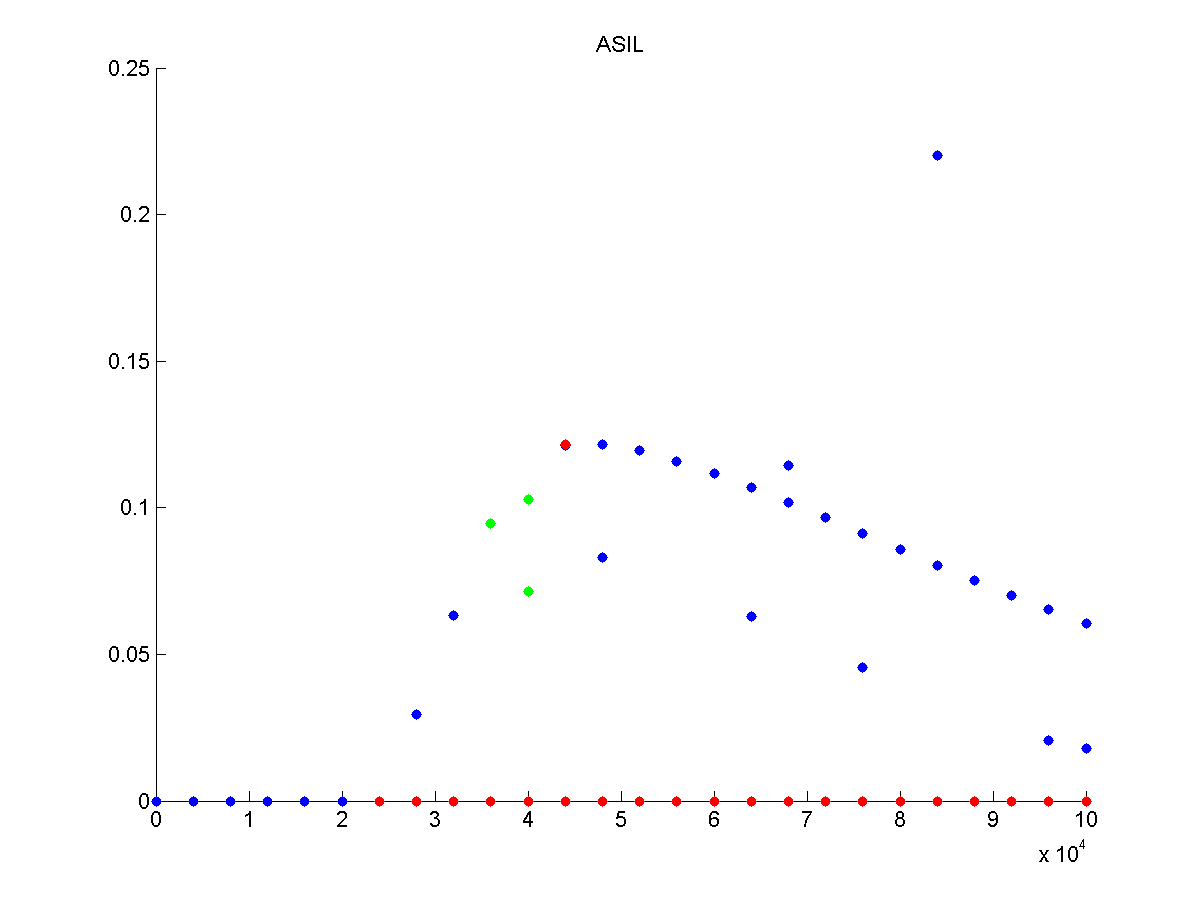

Supplement: Supplementary file 2 [file Presentation2.ZIP › ASIL.png]

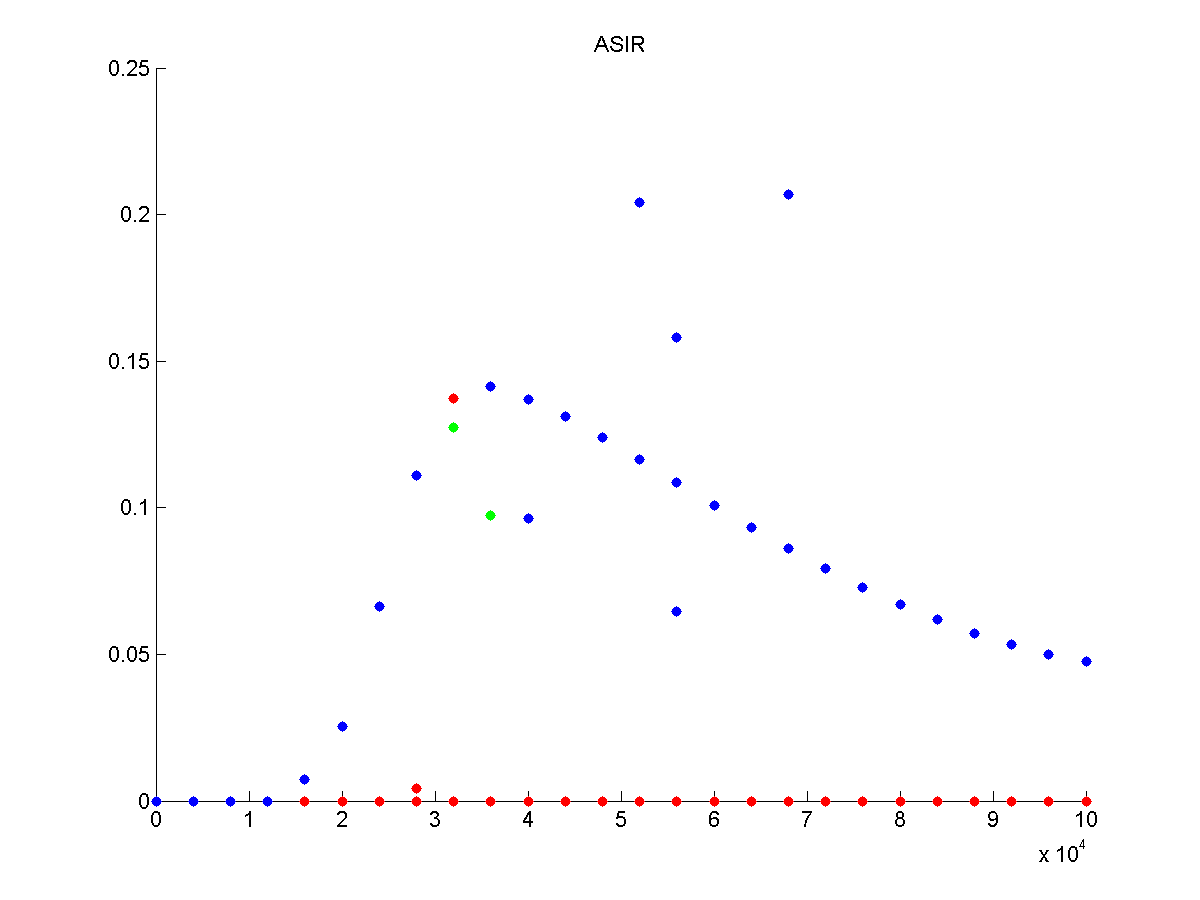

Supplement: Supplementary file 2 [file Presentation2.ZIP › ASIR.png]

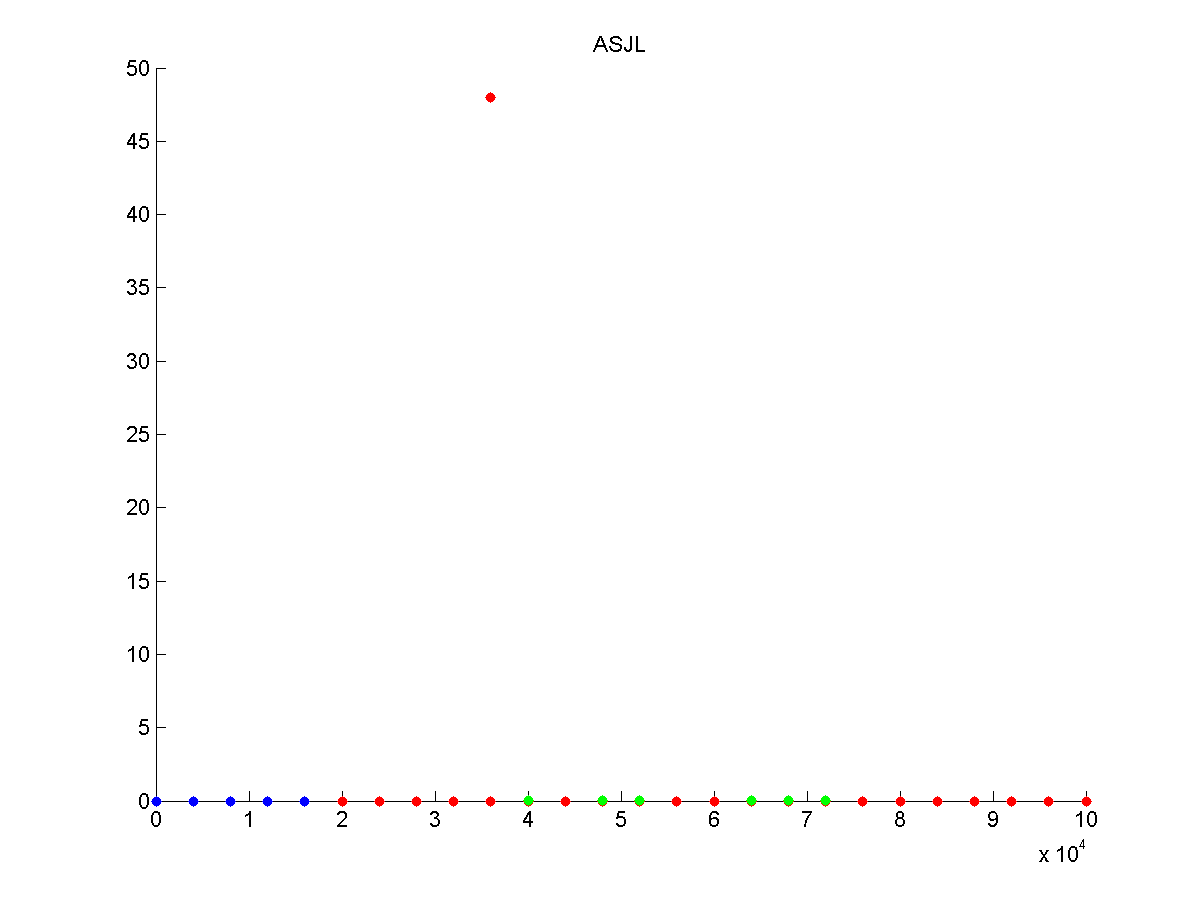

Supplement: Supplementary file 2 [file Presentation2.ZIP › ASJL.png]

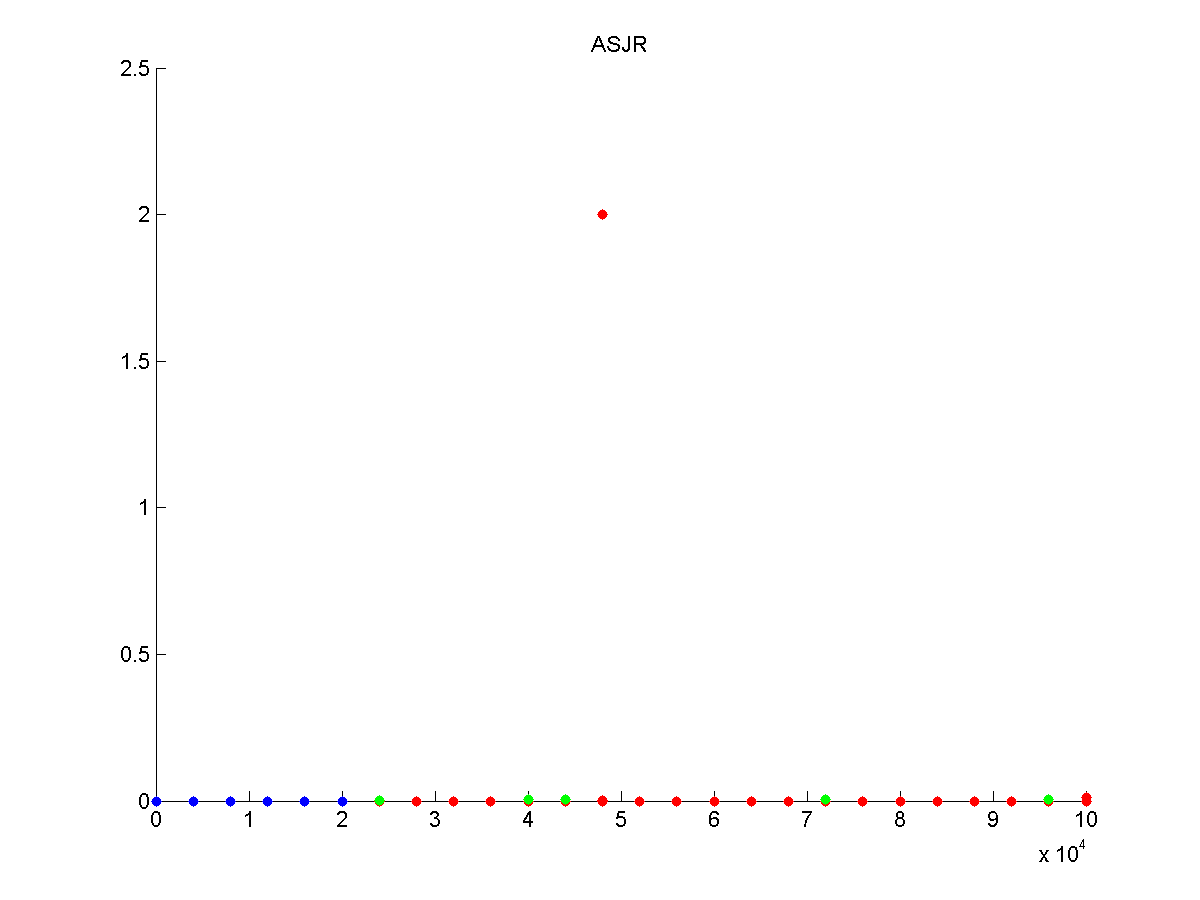

Supplement: Supplementary file 2 [file Presentation2.ZIP › ASJR.png]

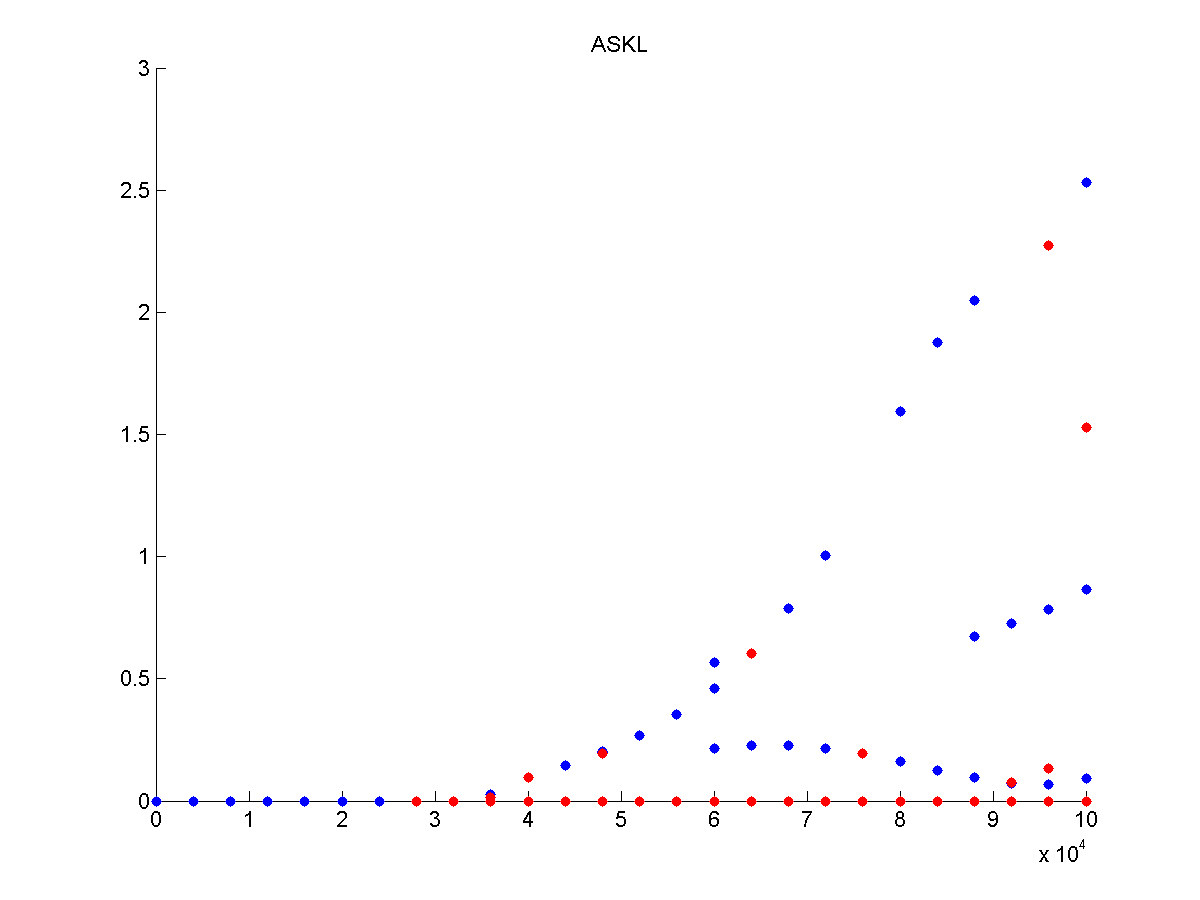

Supplement: Supplementary file 2 [file Presentation2.ZIP › ASKL.png]

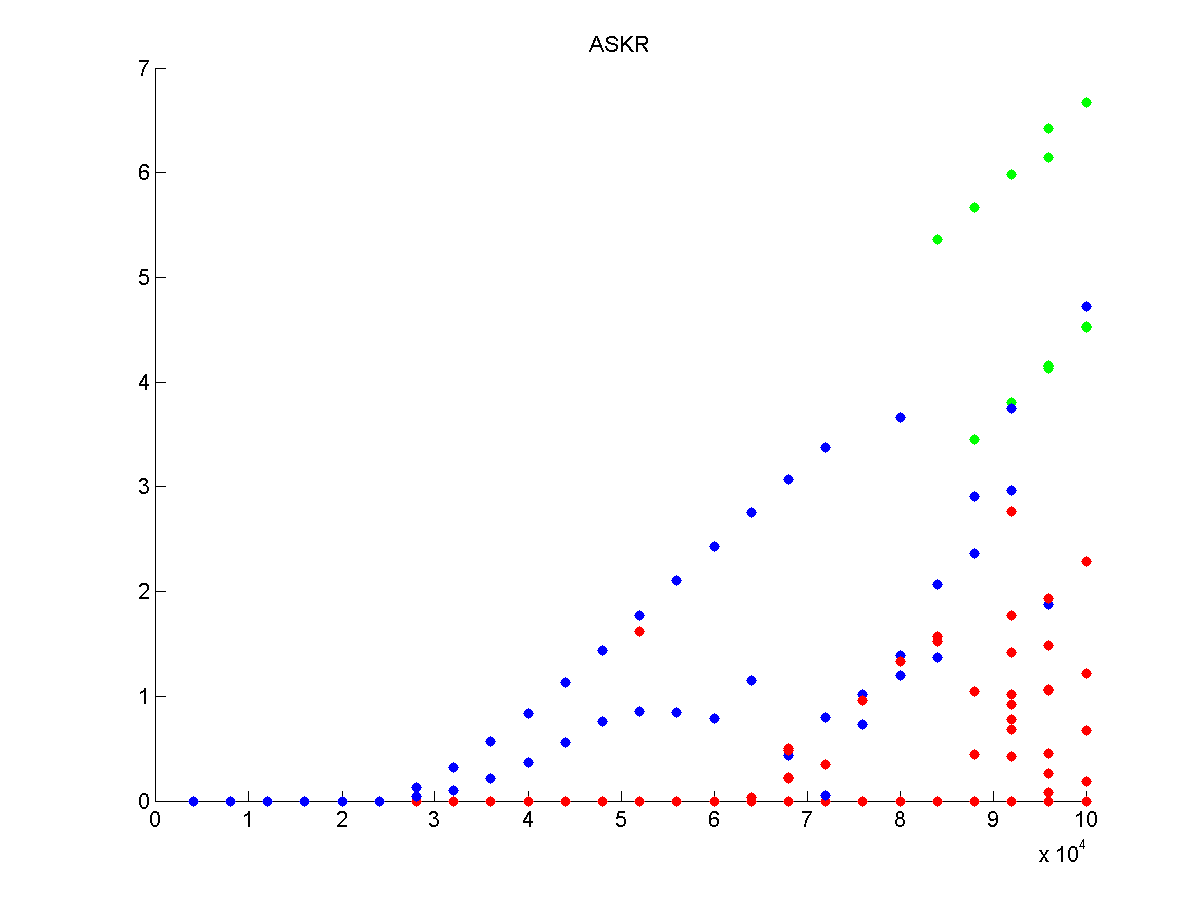

Supplement: Supplementary file 2 [file Presentation2.ZIP › ASKR.png]

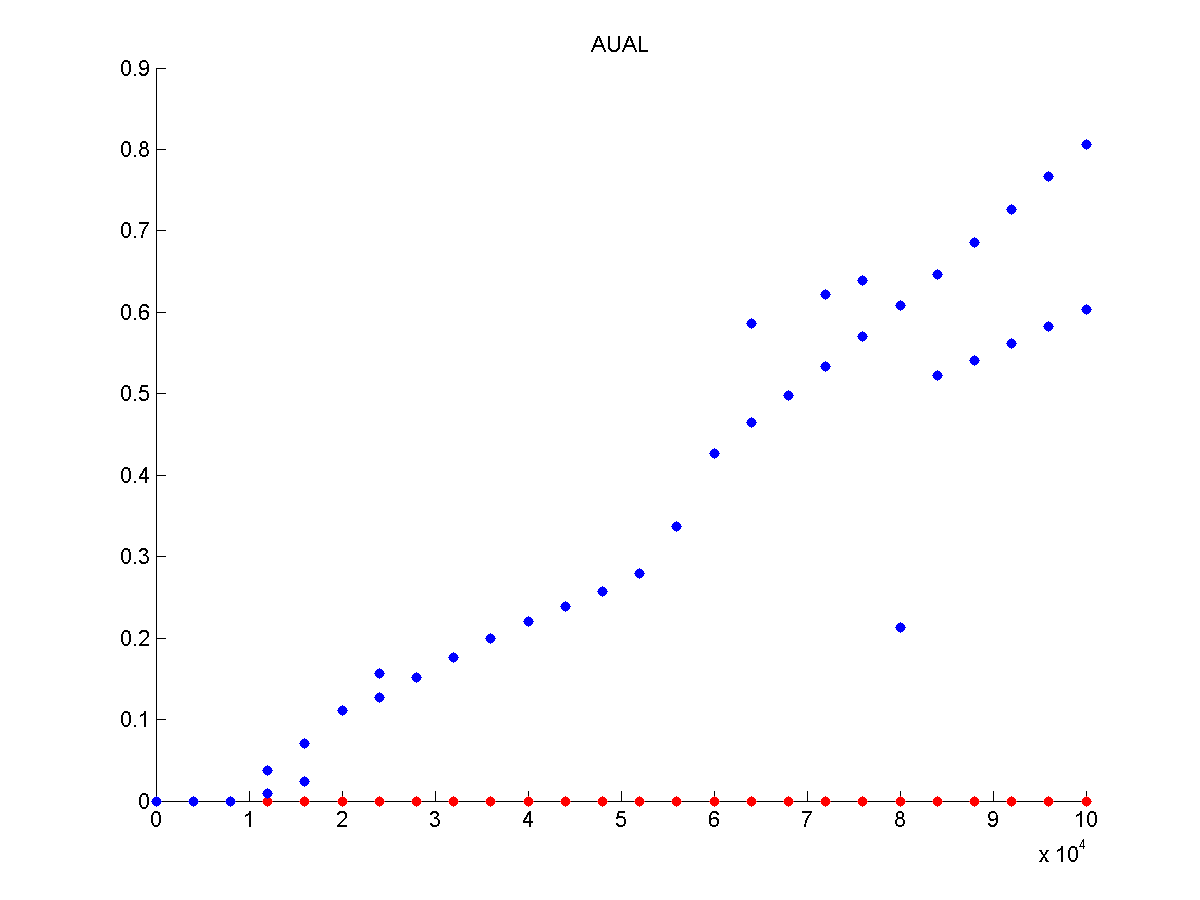

Supplement: Supplementary file 2 [file Presentation2.ZIP › AUAL.png]

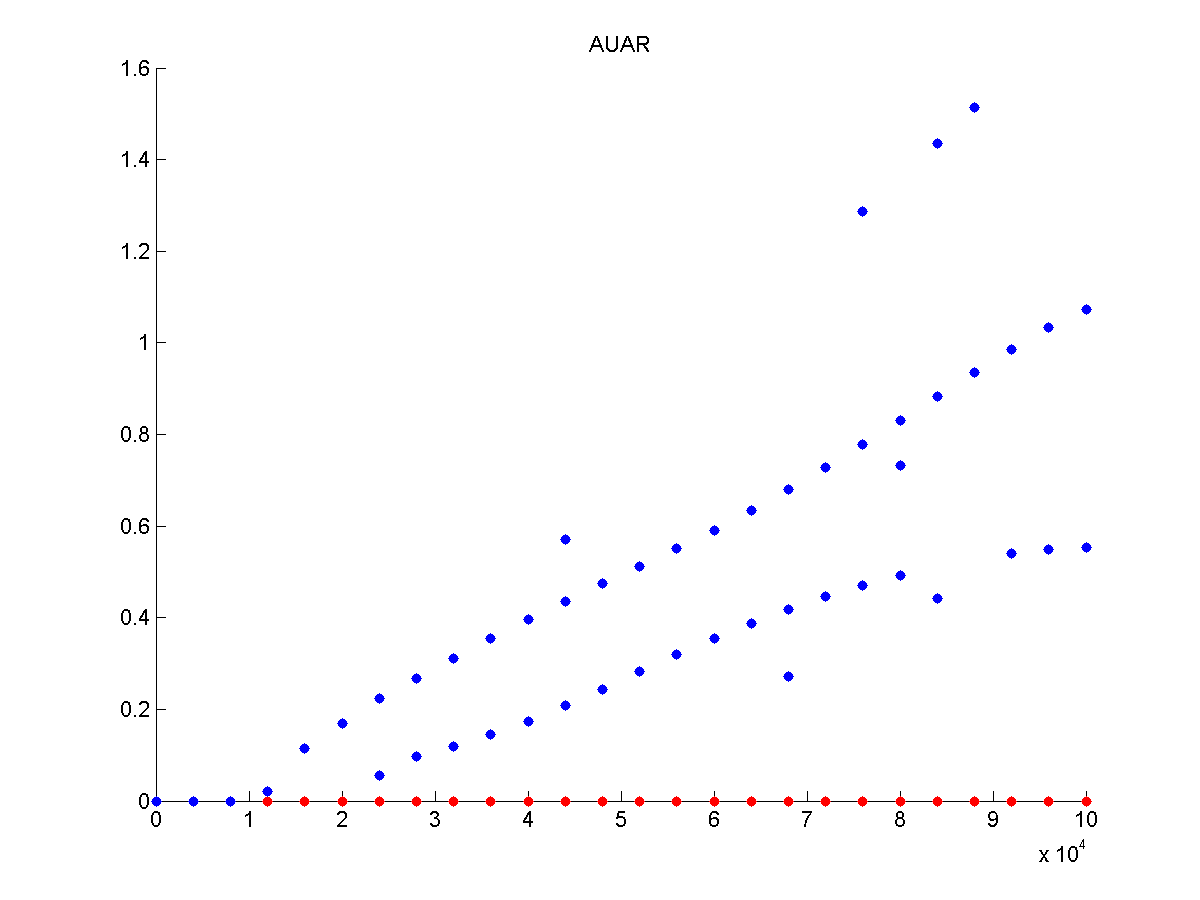

Supplement: Supplementary file 2 [file Presentation2.ZIP › AUAR.png]

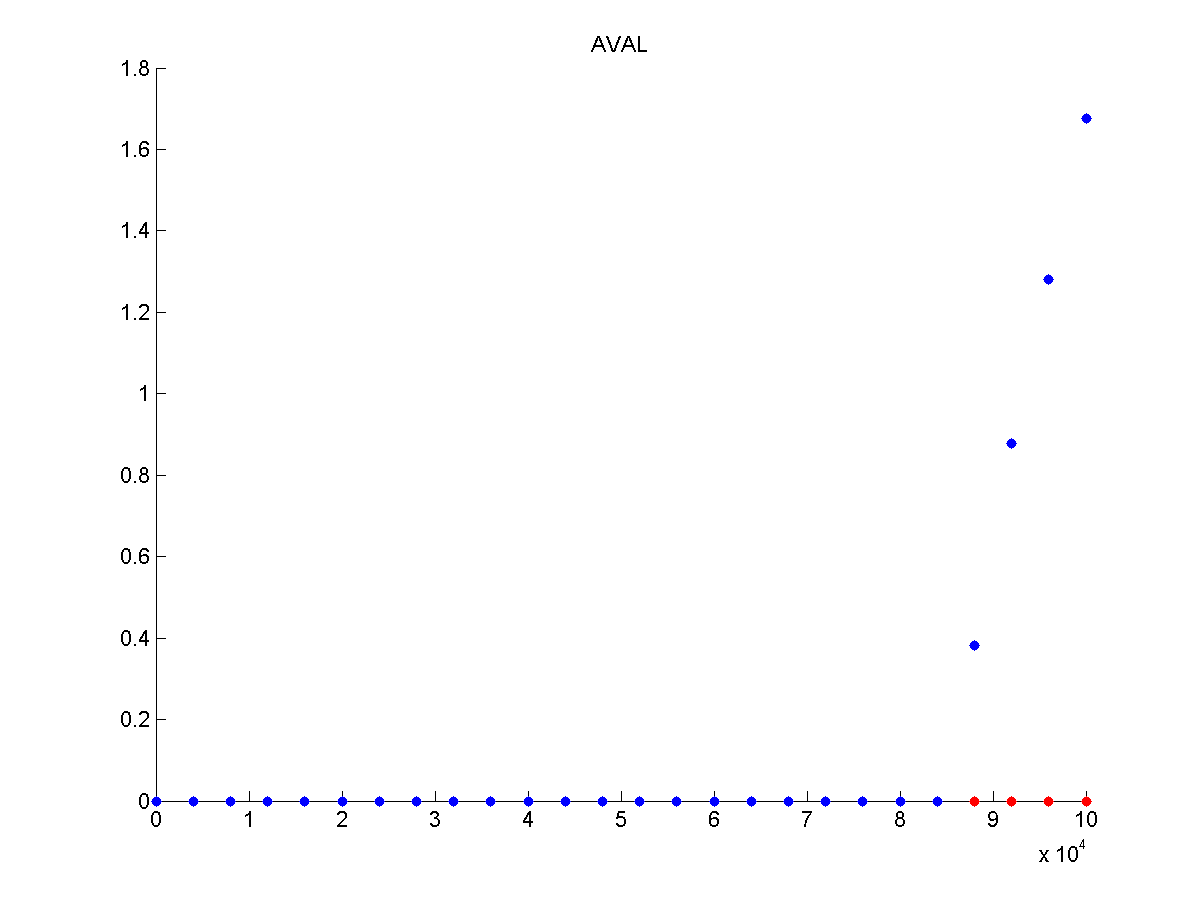

Supplement: Supplementary file 2 [file Presentation2.ZIP › AVAL.png]

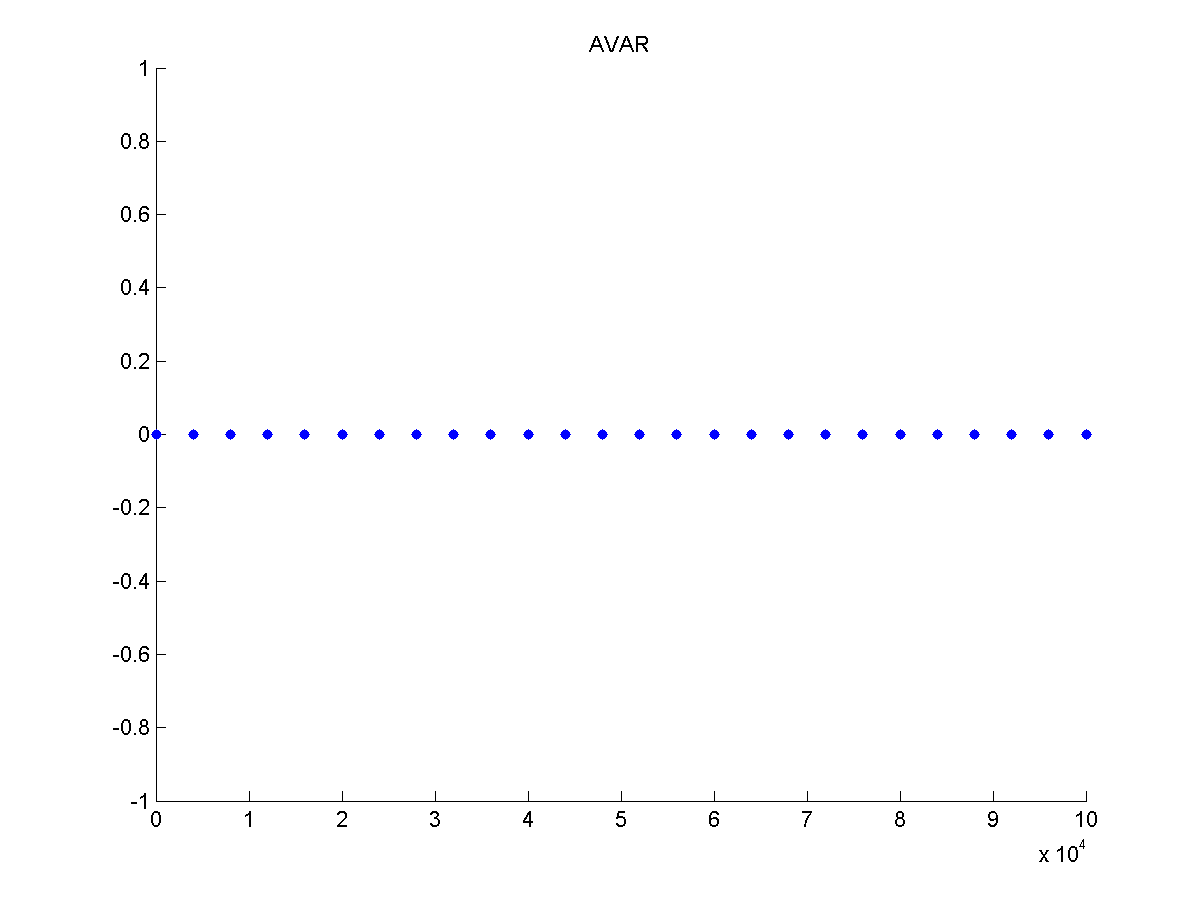

Supplement: Supplementary file 2 [file Presentation2.ZIP › AVAR.png]

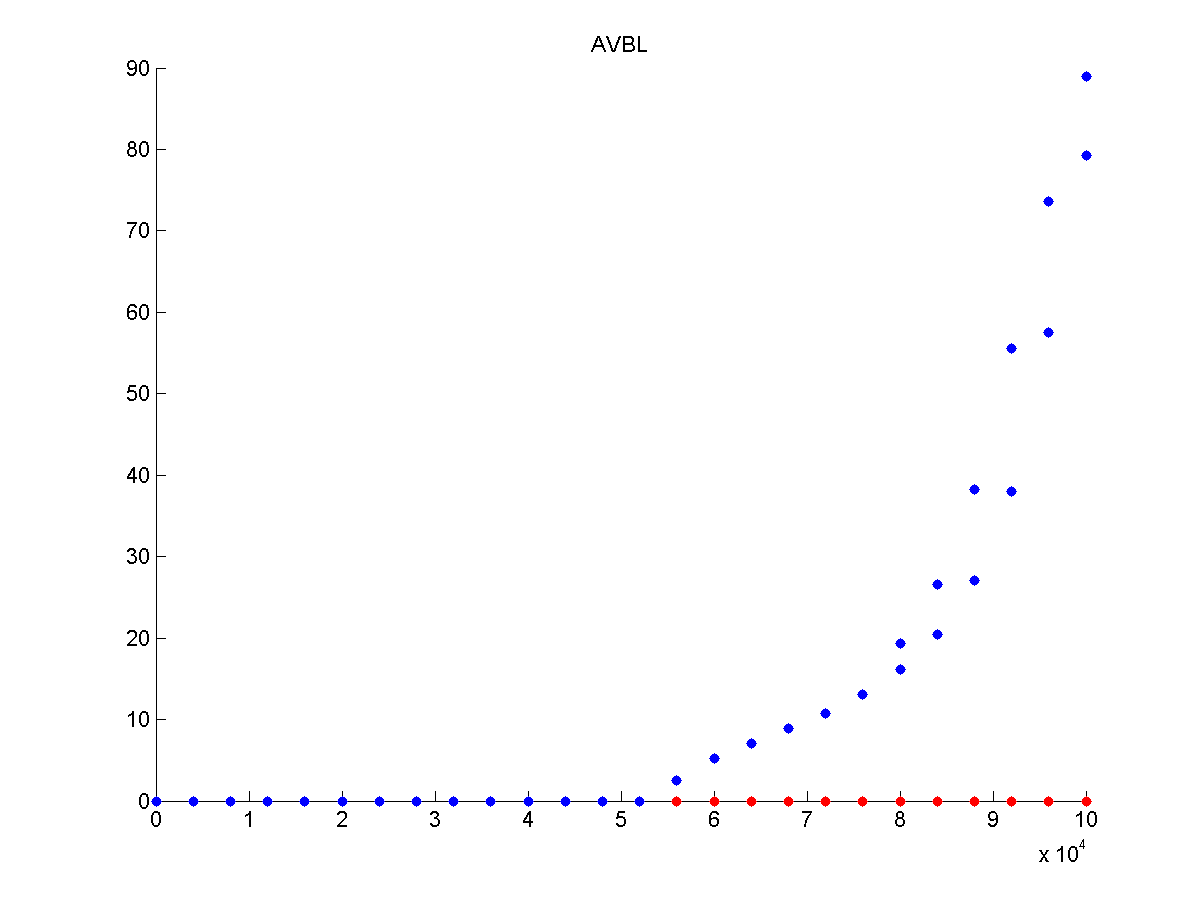

Supplement: Supplementary file 2 [file Presentation2.ZIP › AVBL.png]

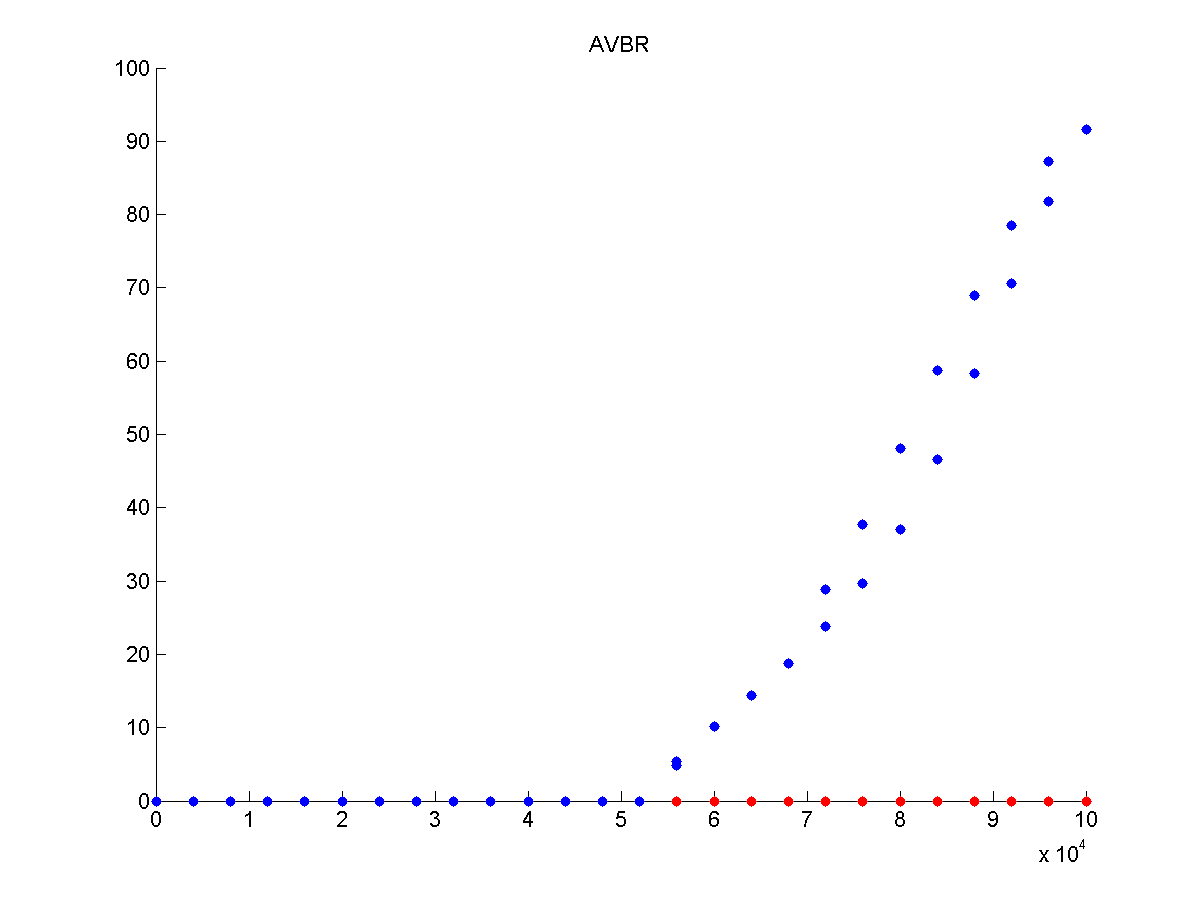

Supplement: Supplementary file 2 [file Presentation2.ZIP › AVBR.png]

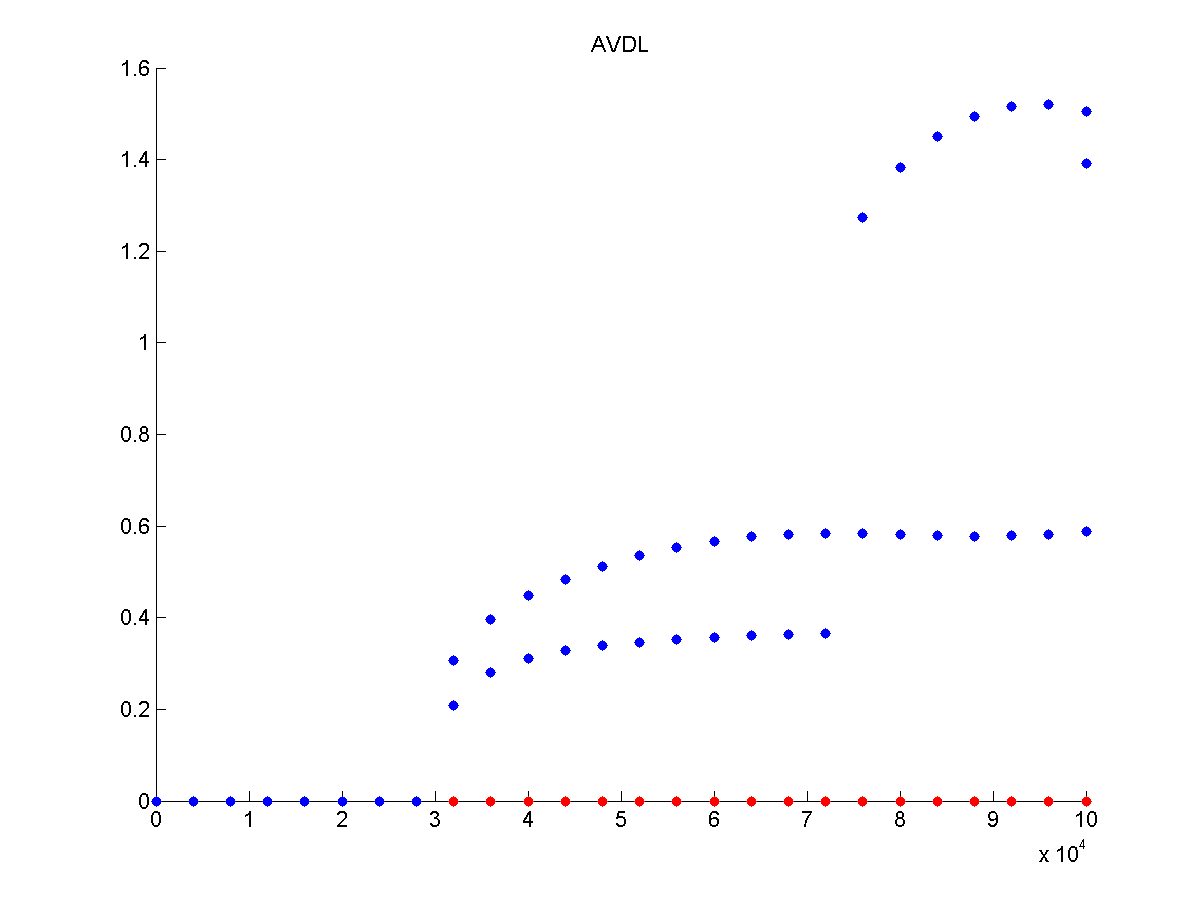

Supplement: Supplementary file 2 [file Presentation2.ZIP › AVDL.png]

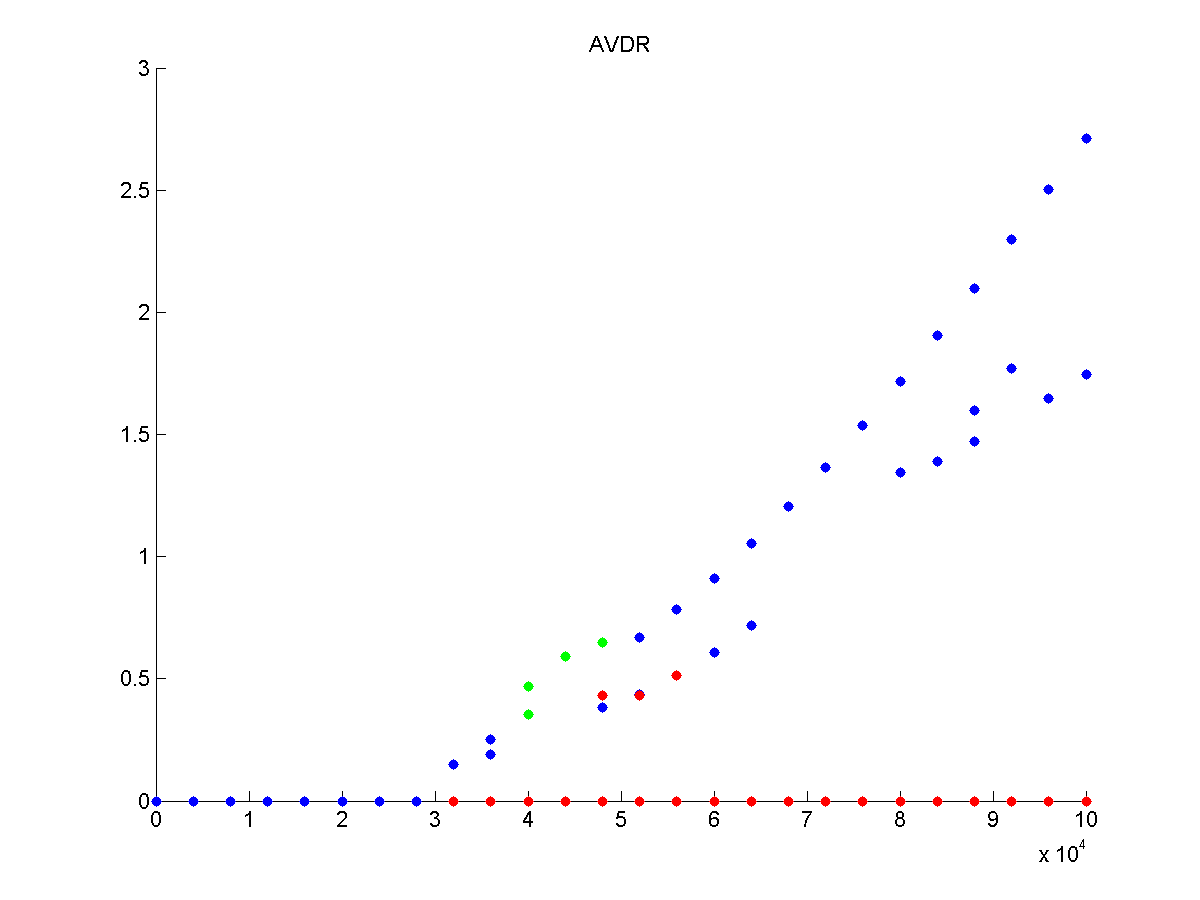

Supplement: Supplementary file 2 [file Presentation2.ZIP › AVDR.png]

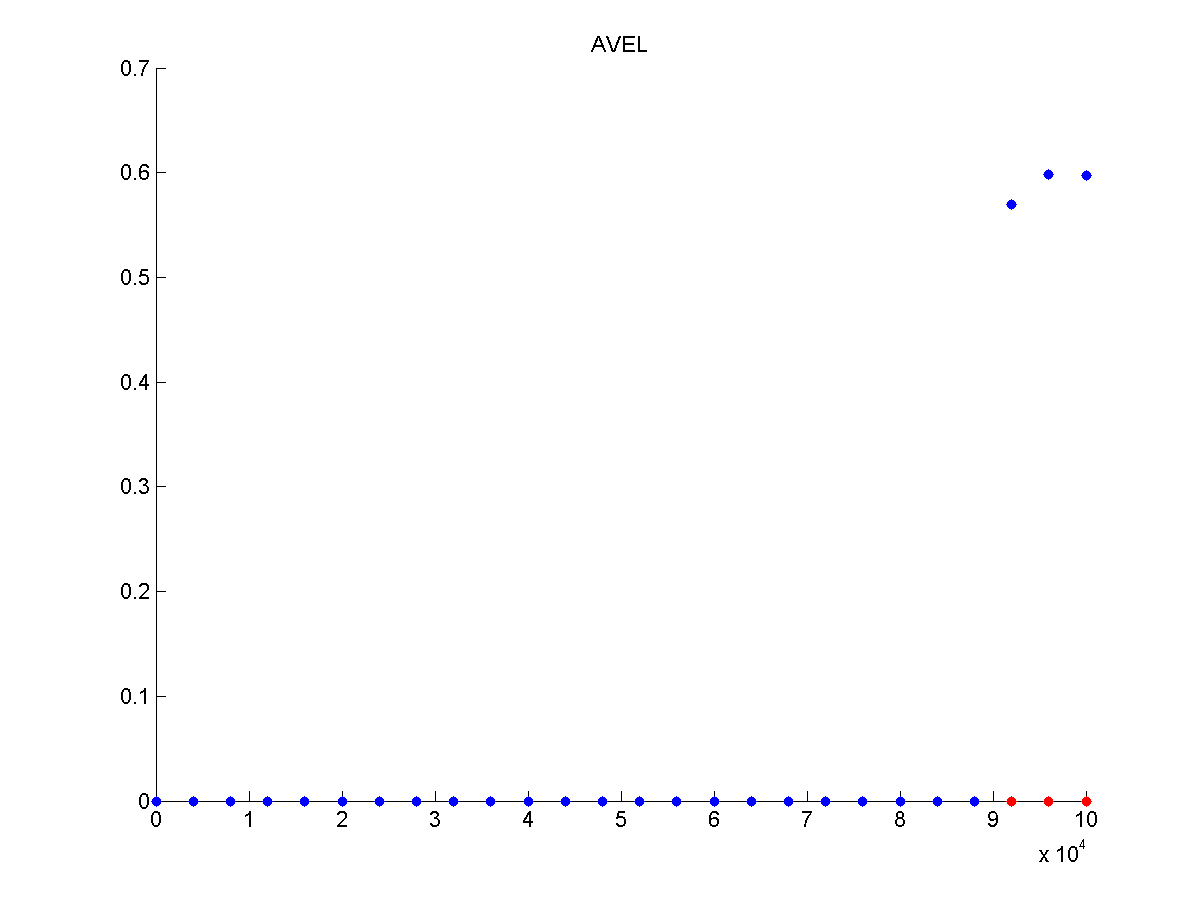

Supplement: Supplementary file 2 [file Presentation2.ZIP › AVEL.png]

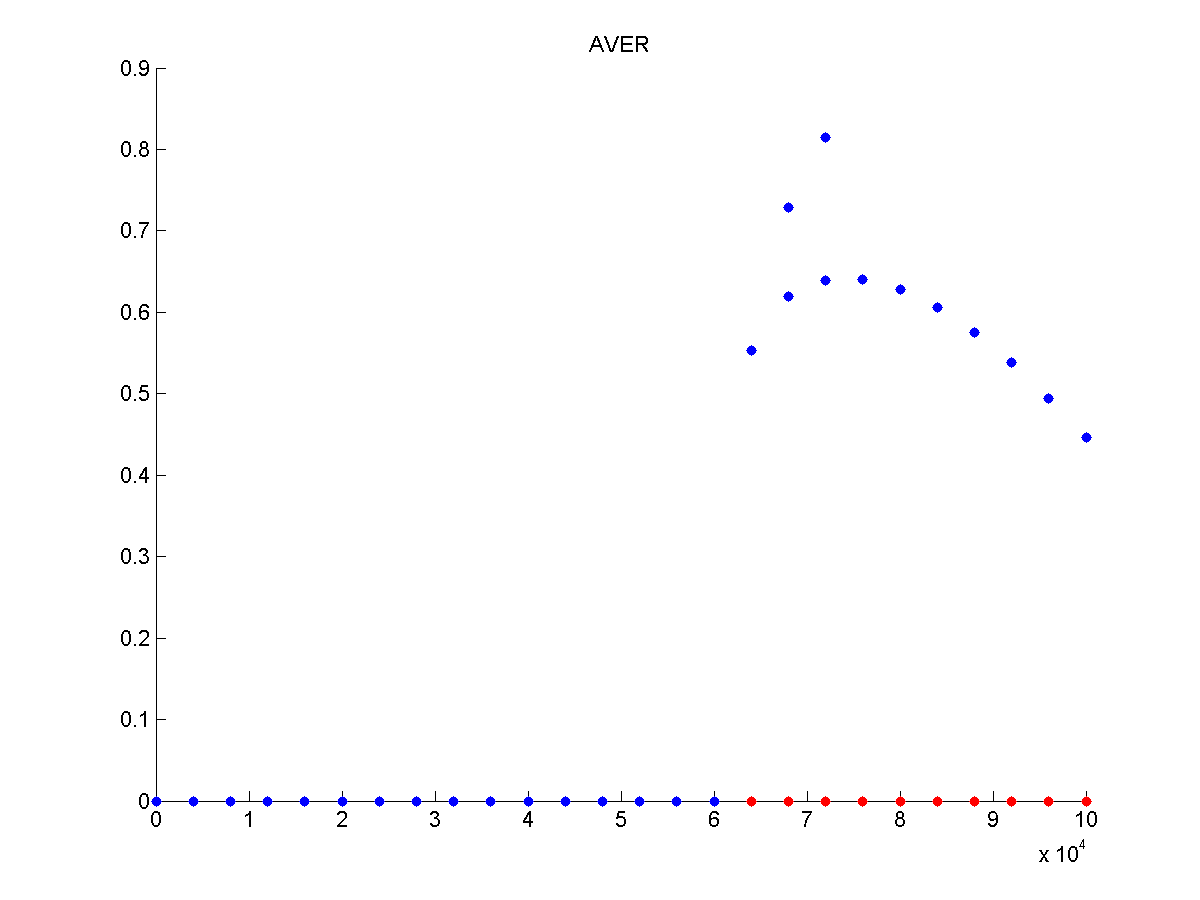

Supplement: Supplementary file 2 [file Presentation2.ZIP › AVER.png]

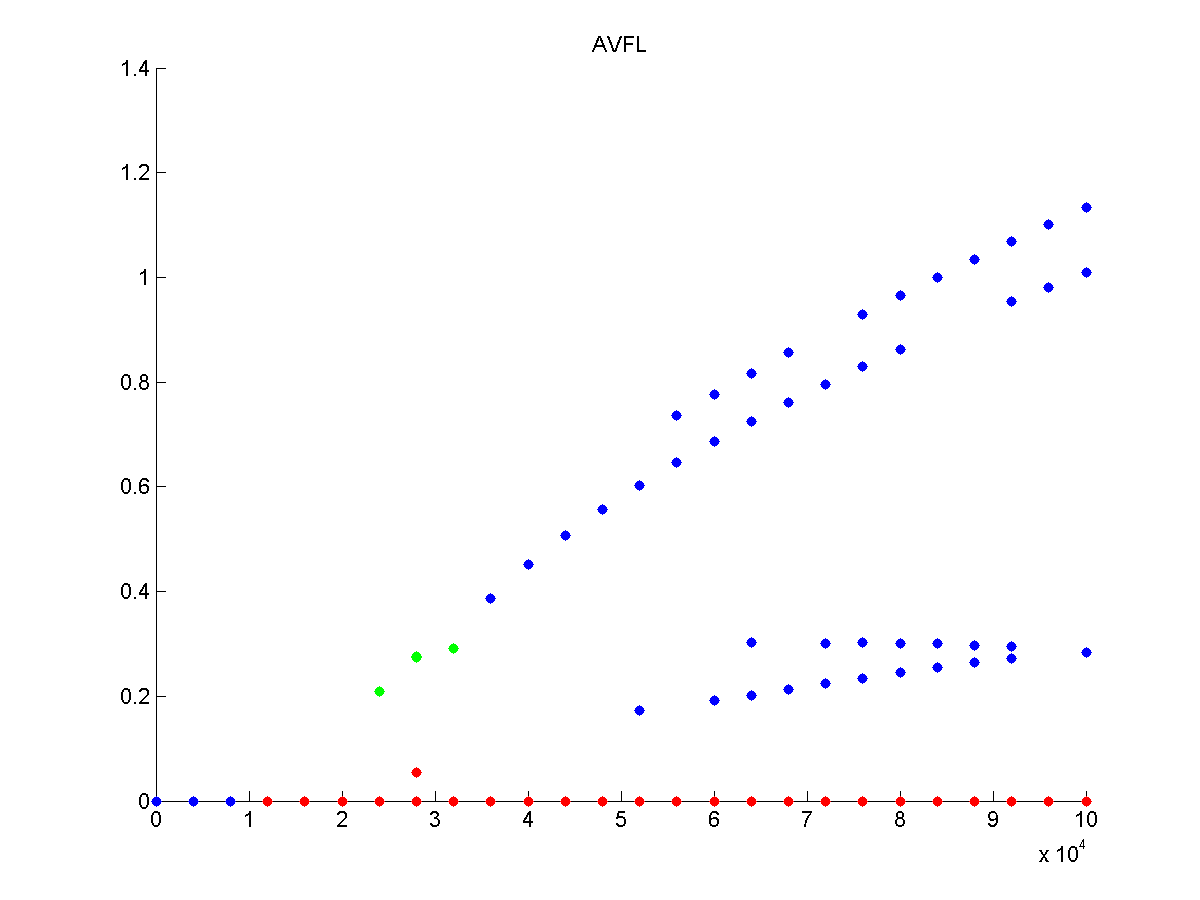

Supplement: Supplementary file 2 [file Presentation2.ZIP › AVFL.png]

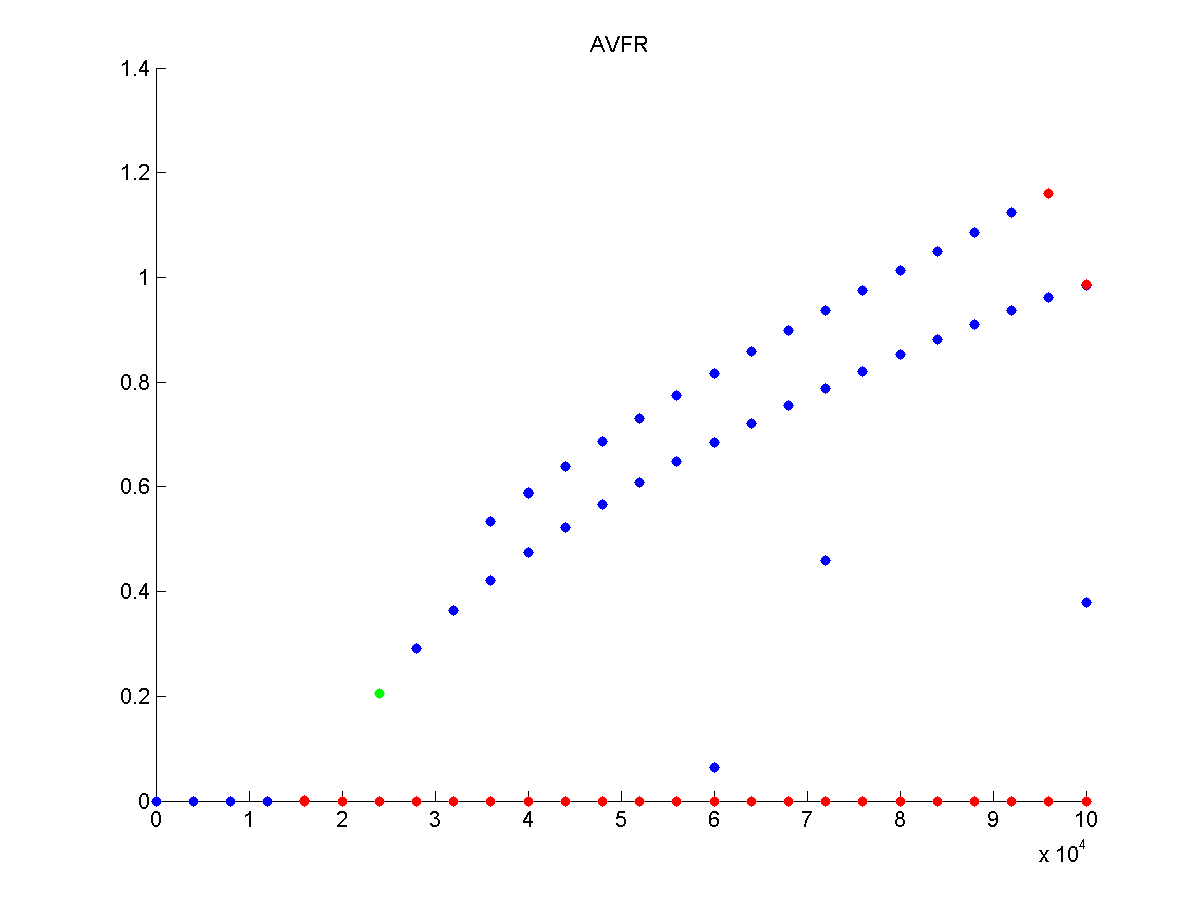

Supplement: Supplementary file 2 [file Presentation2.ZIP › AVFR.png]

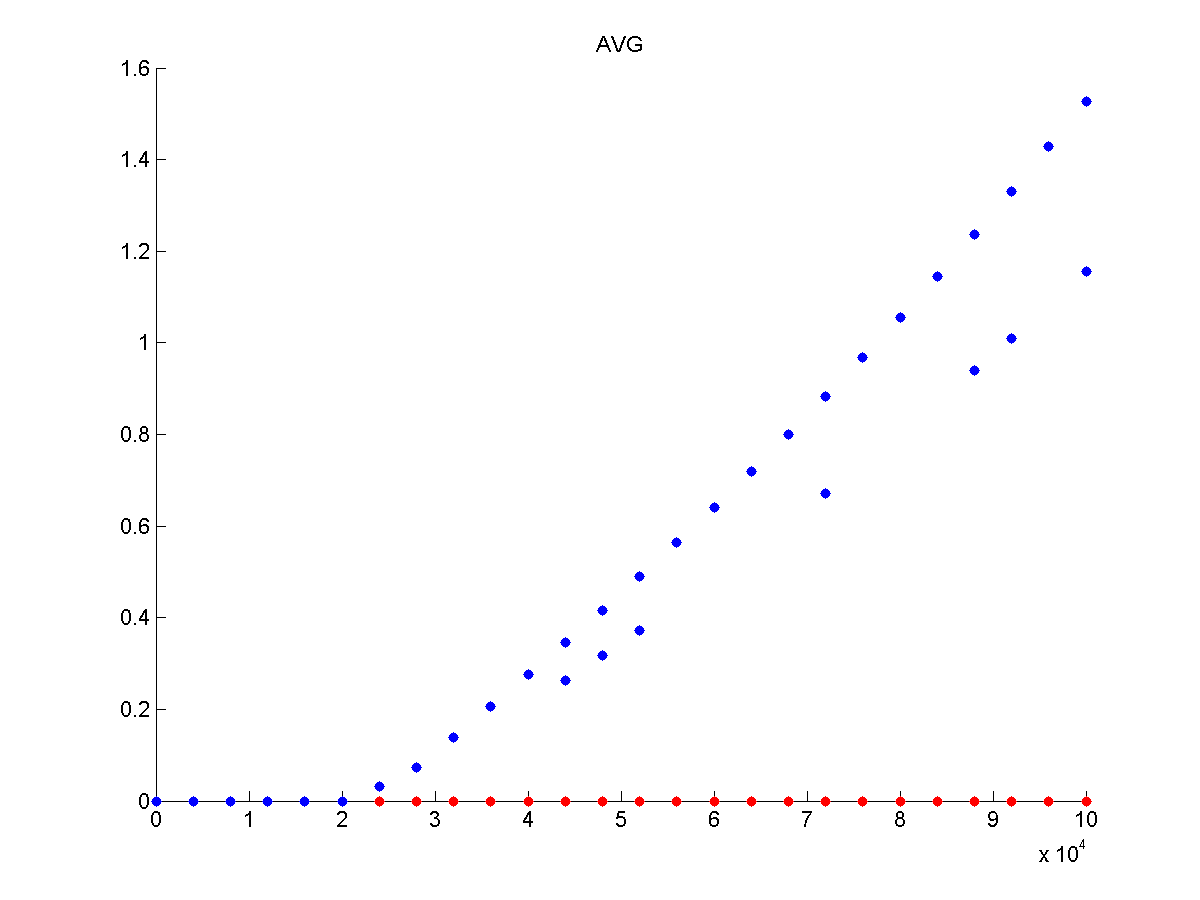

Supplement: Supplementary file 2 [file Presentation2.ZIP › AVG.png]

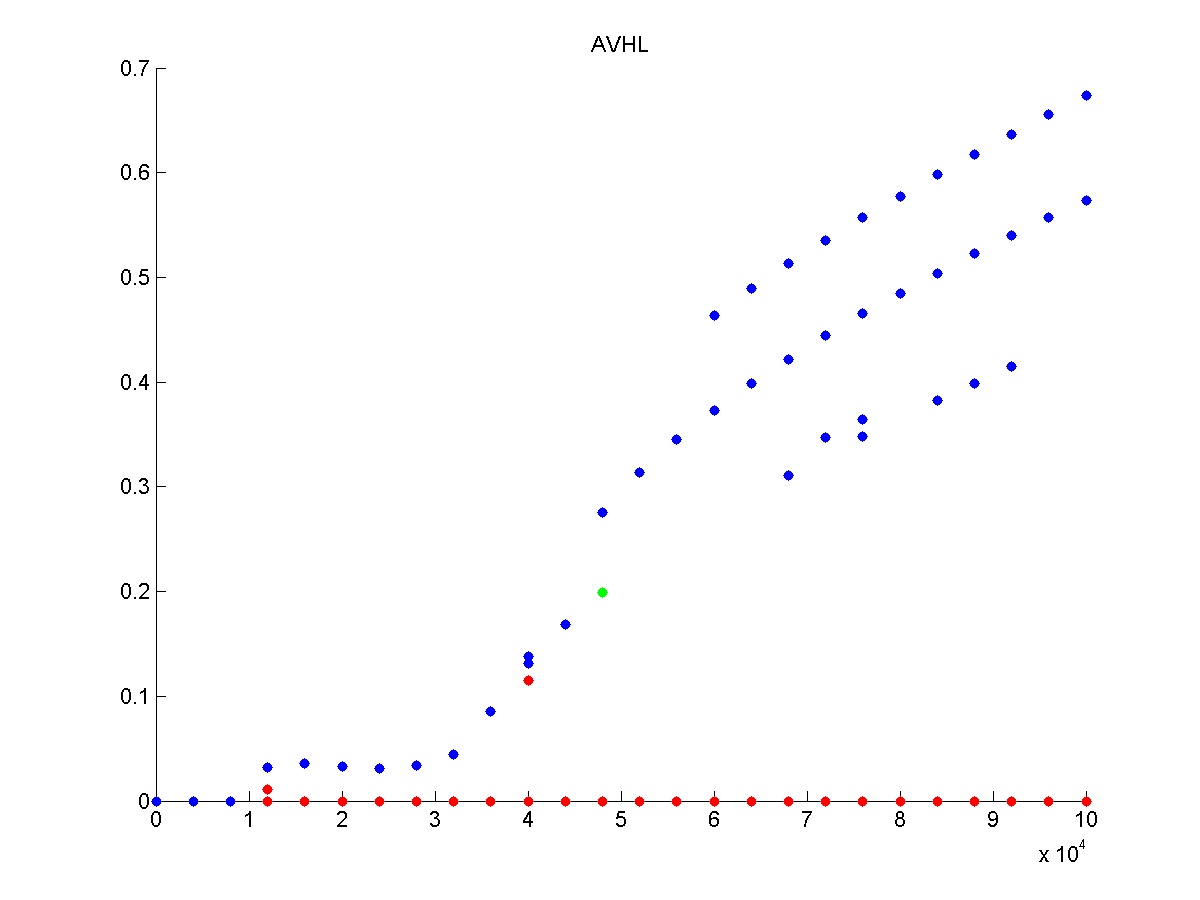

Supplement: Supplementary file 2 [file Presentation2.ZIP › AVHL.png]

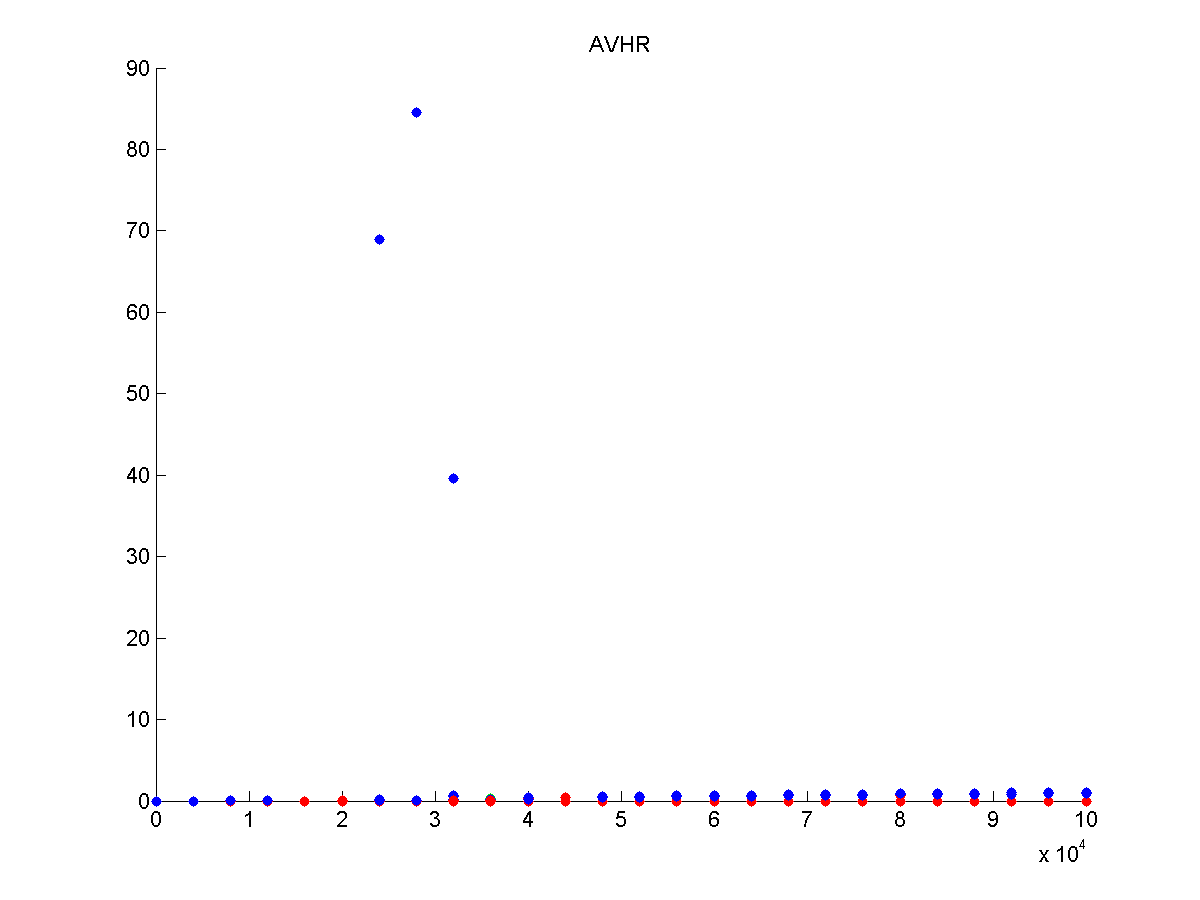

Supplement: Supplementary file 2 [file Presentation2.ZIP › AVHR.png]

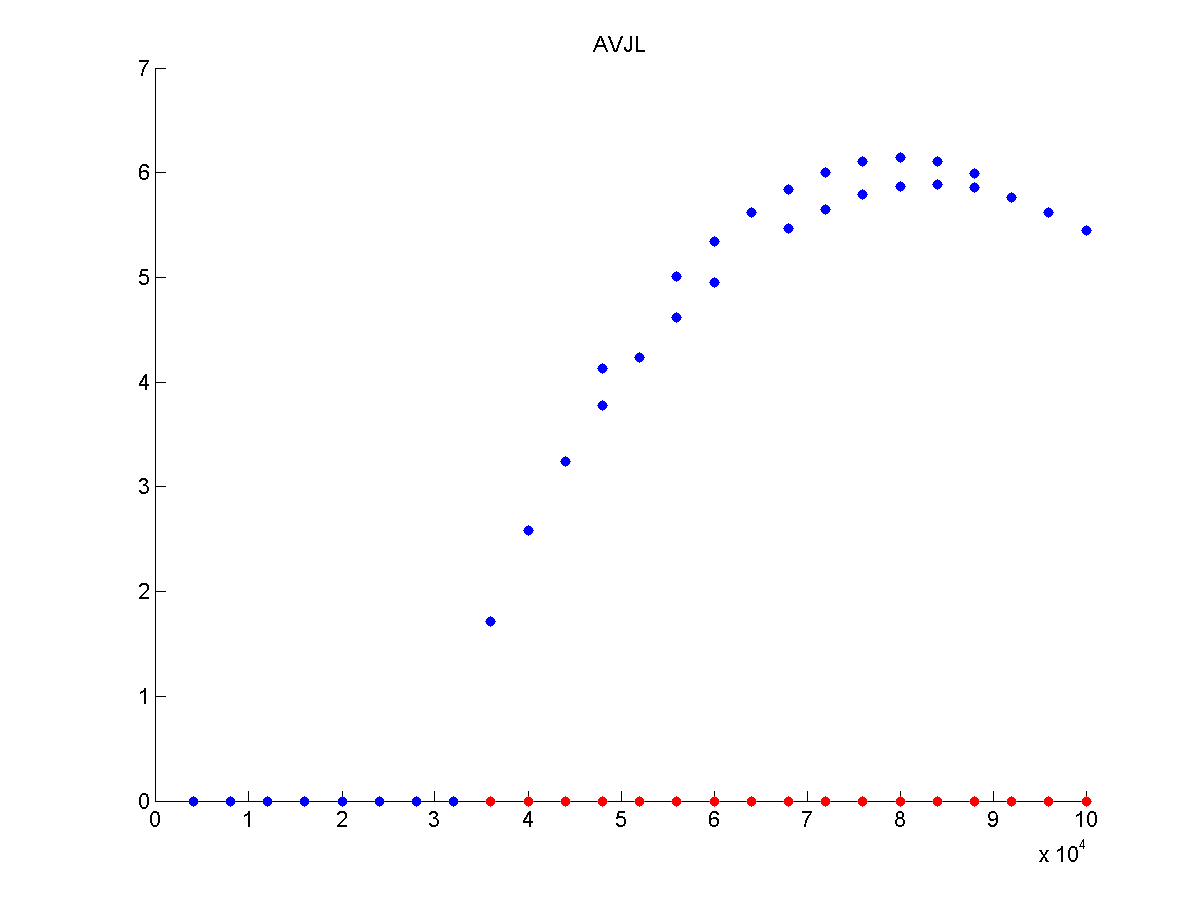

Supplement: Supplementary file 2 [file Presentation2.ZIP › AVJL.png]

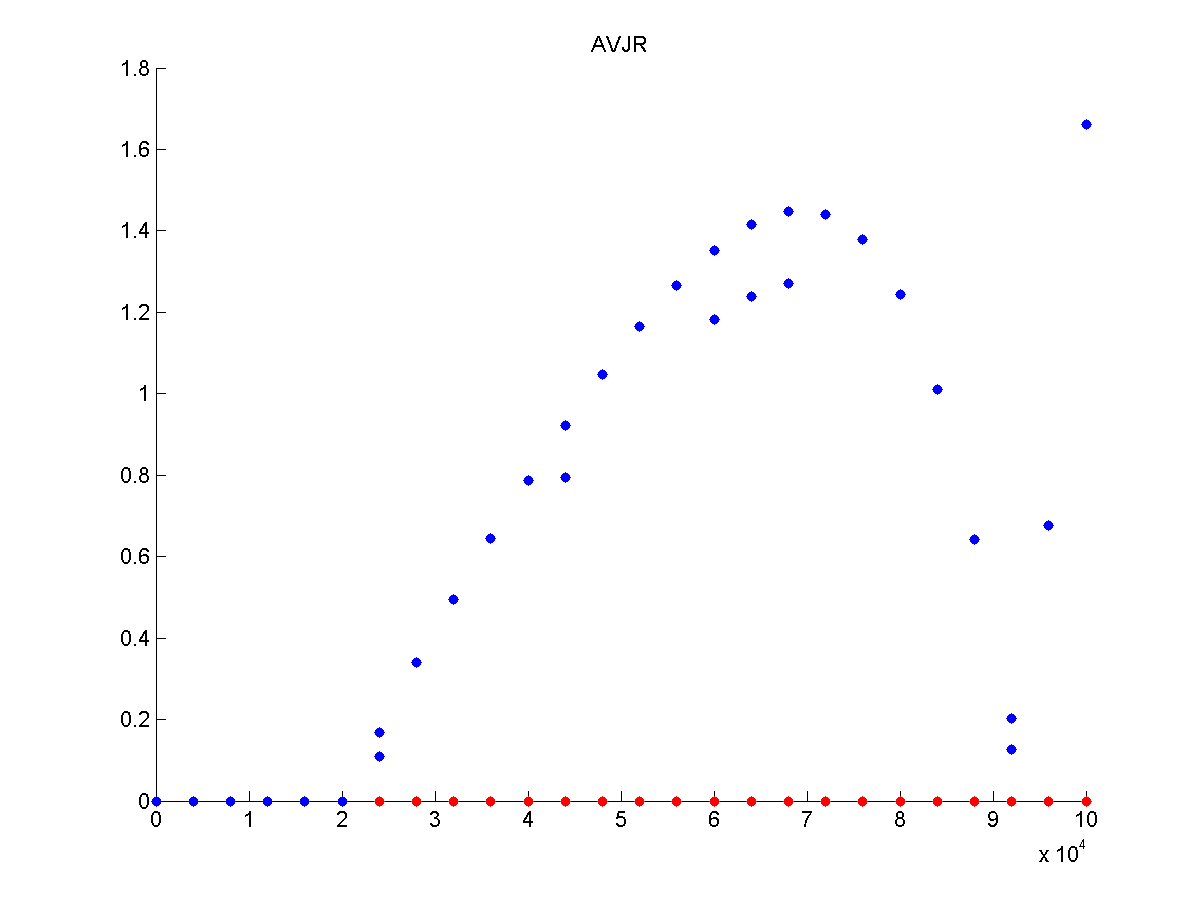

Supplement: Supplementary file 2 [file Presentation2.ZIP › AVJR.png]

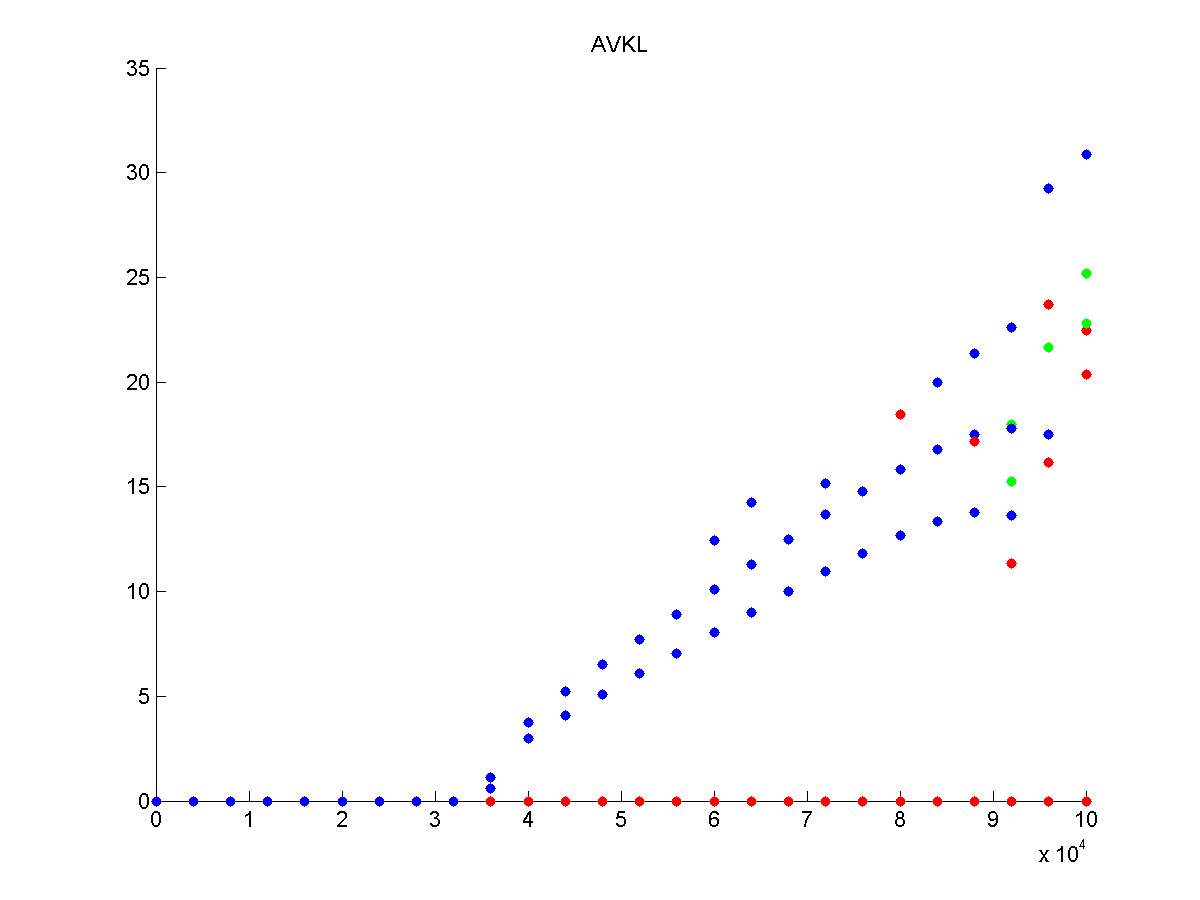

Supplement: Supplementary file 2 [file Presentation2.ZIP › AVKL.png]

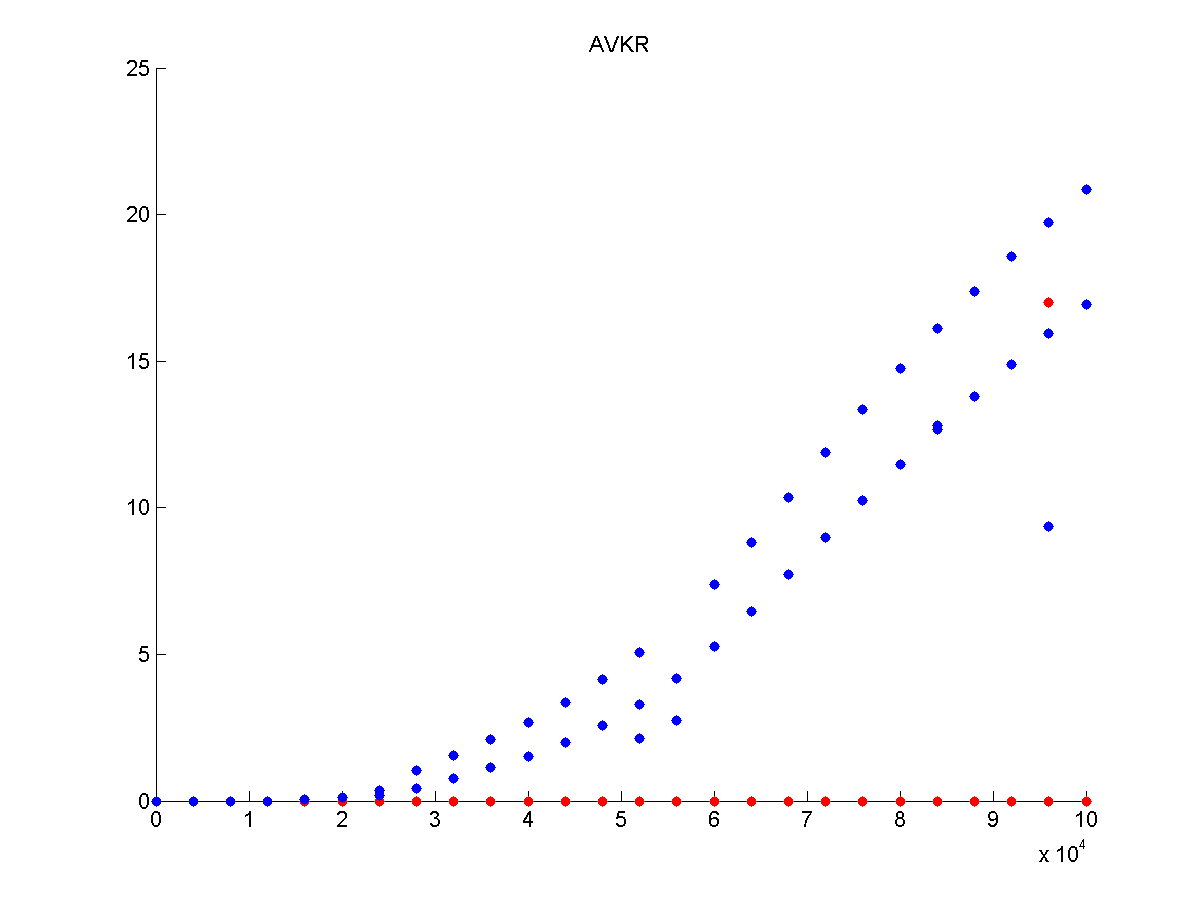

Supplement: Supplementary file 2 [file Presentation2.ZIP › AVKR.png]

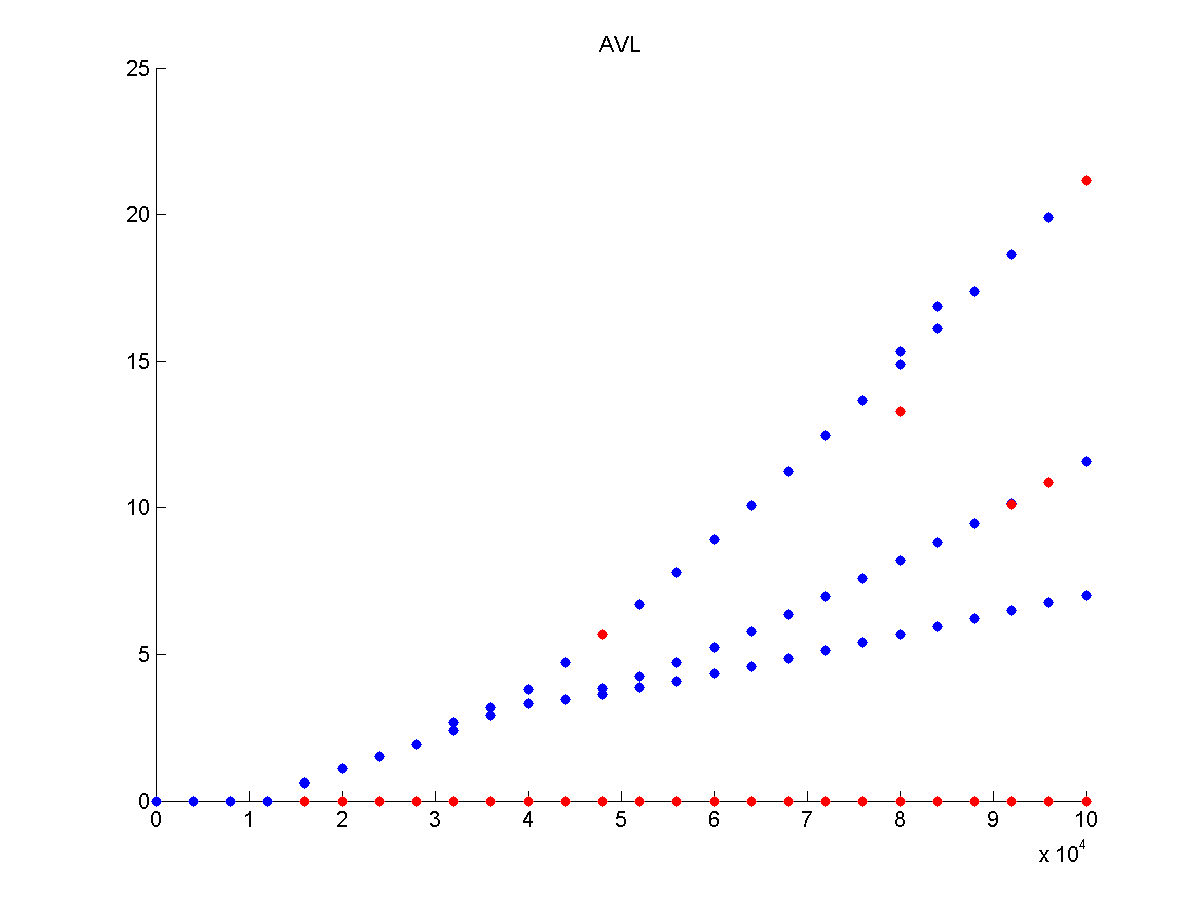

Supplement: Supplementary file 2 [file Presentation2.ZIP › AVL.png]

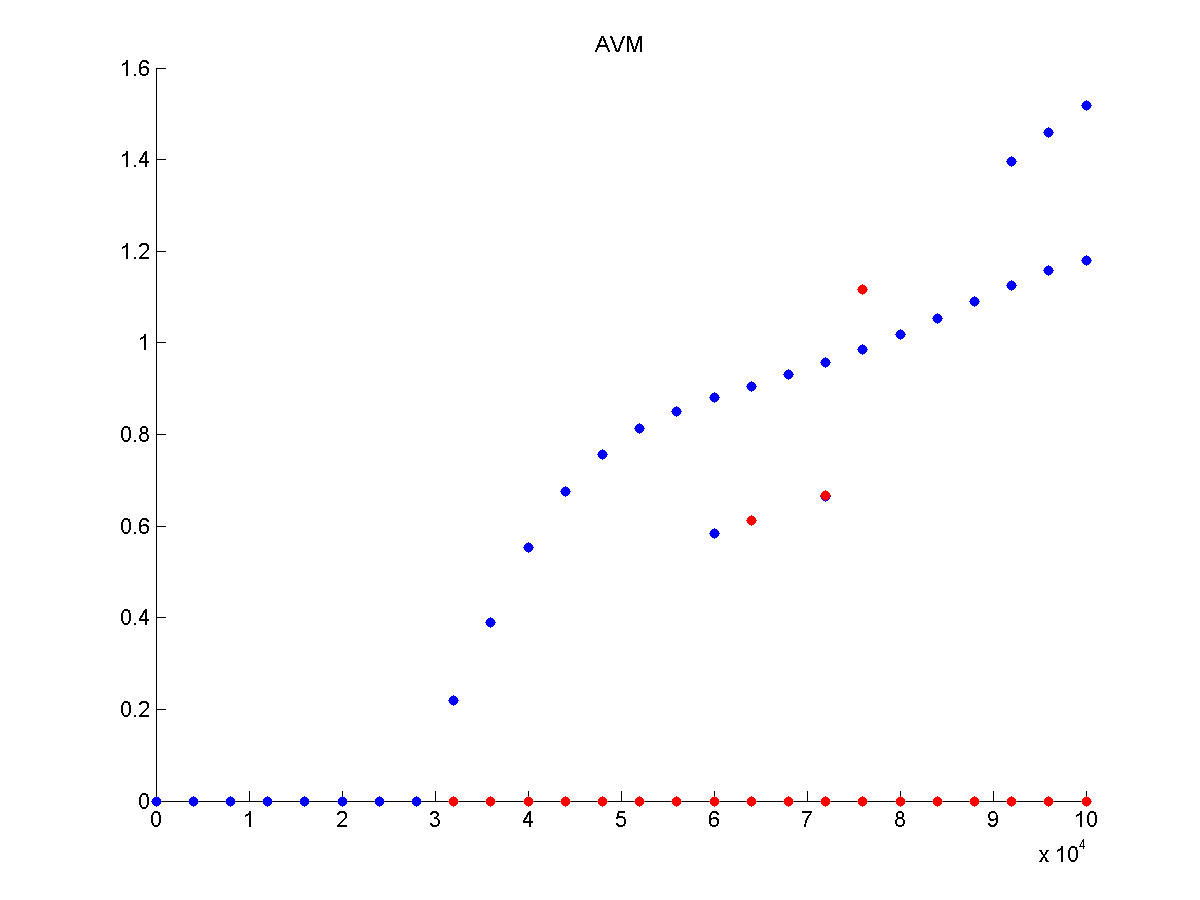

Supplement: Supplementary file 2 [file Presentation2.ZIP › AVM.png]

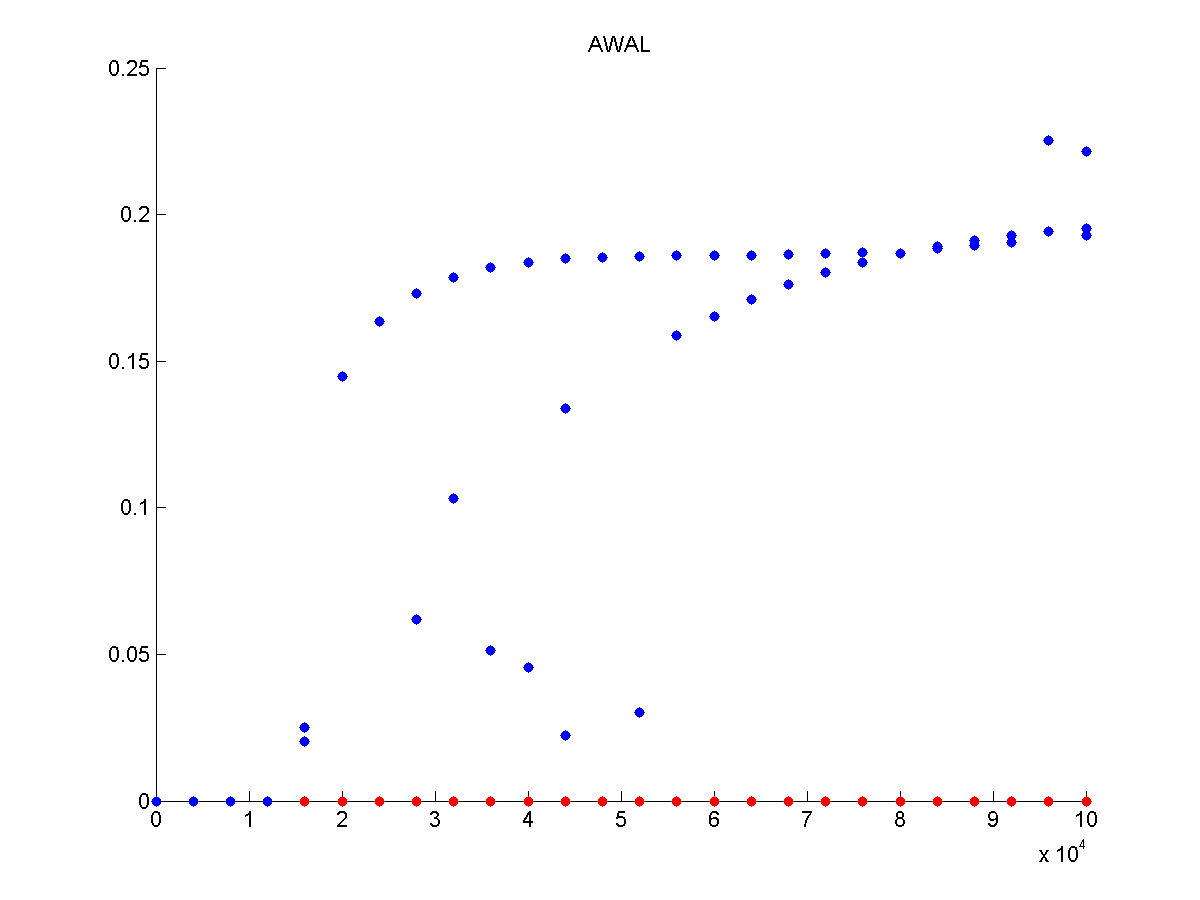

Supplement: Supplementary file 2 [file Presentation2.ZIP › AWAL.png]

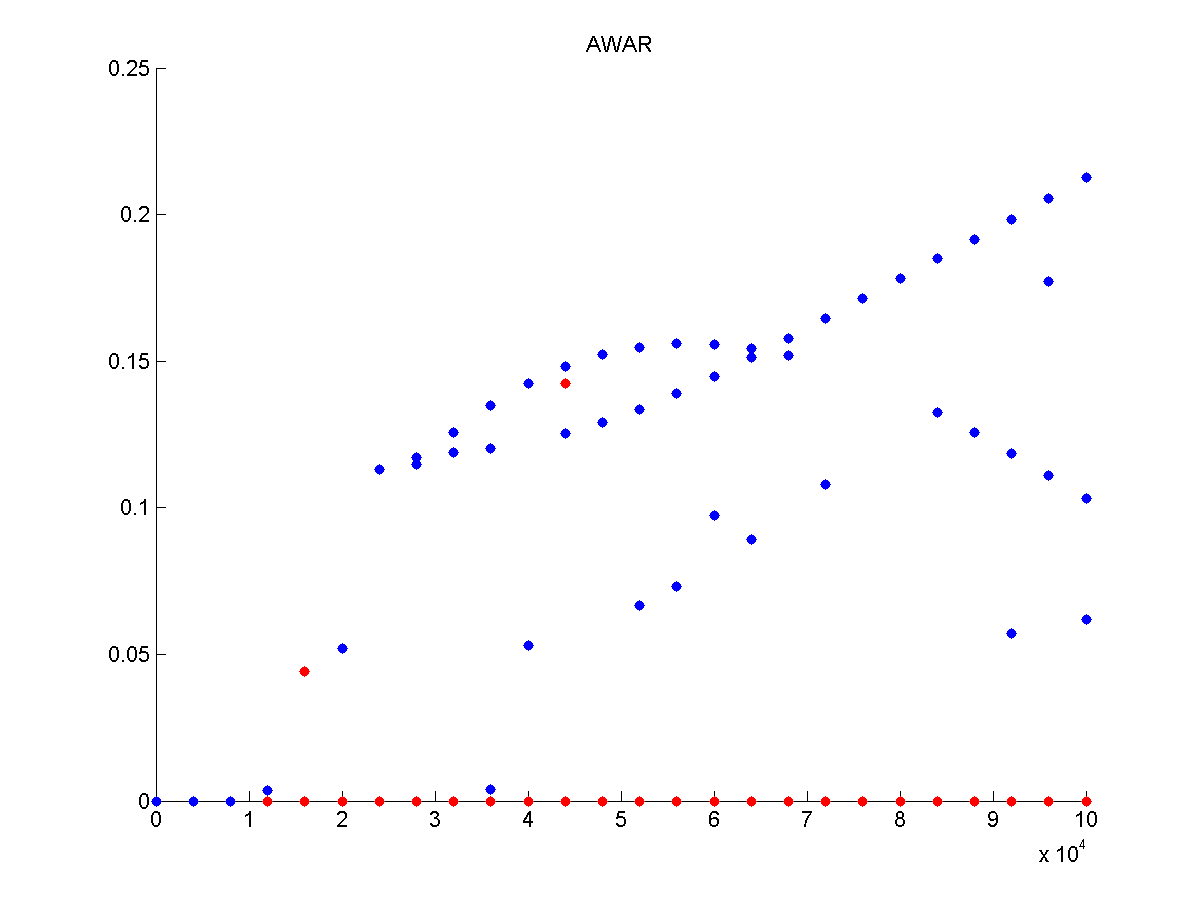

Supplement: Supplementary file 2 [file Presentation2.ZIP › AWAR.png]

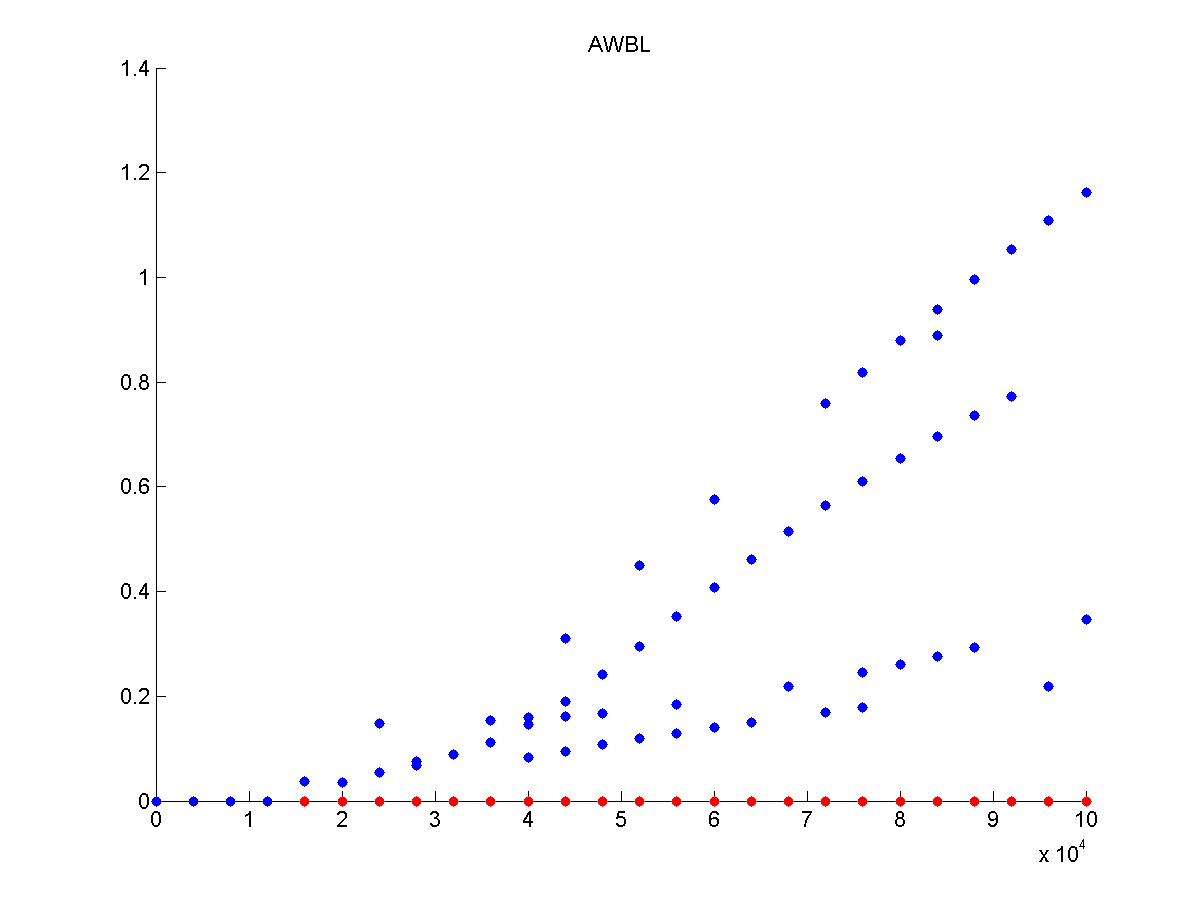

Supplement: Supplementary file 2 [file Presentation2.ZIP › AWBL.png]

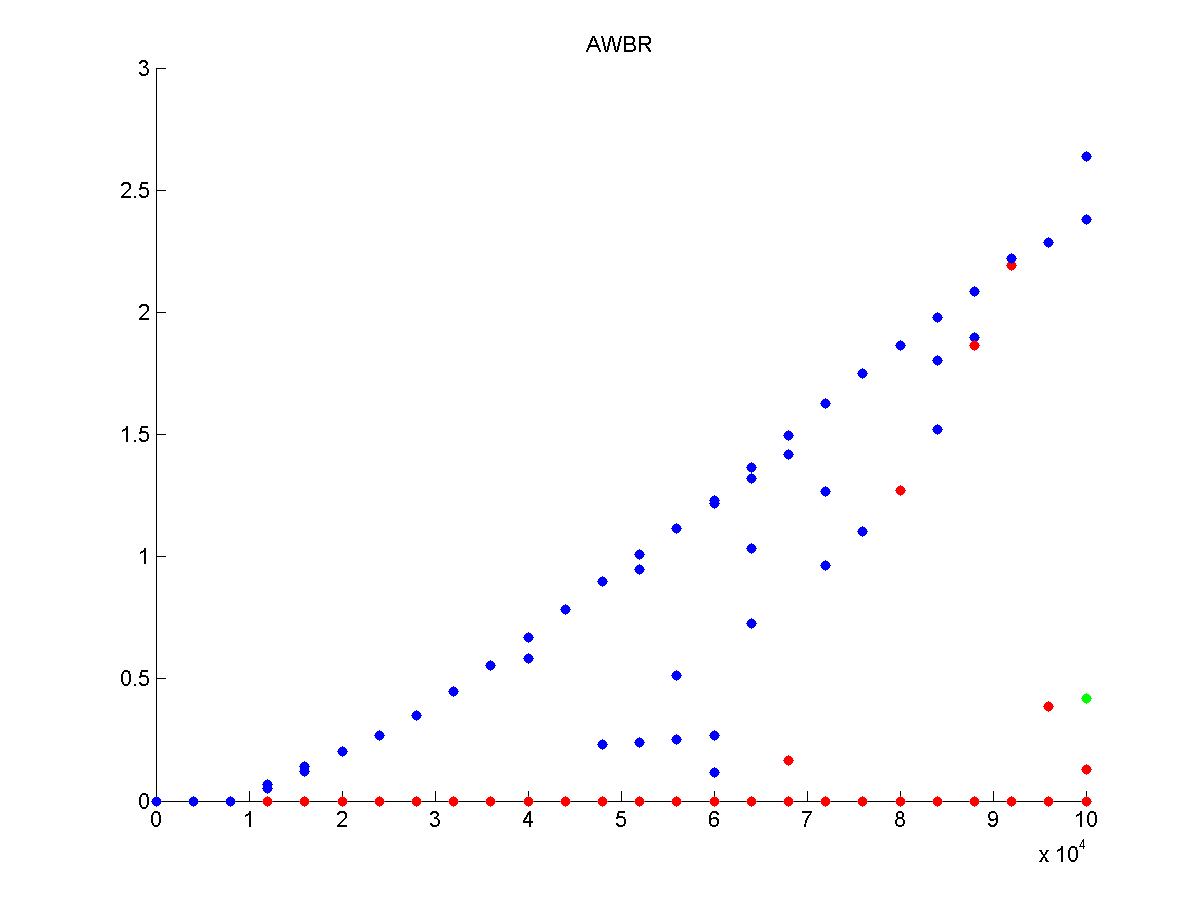

Supplement: Supplementary file 2 [file Presentation2.ZIP › AWBR.png]

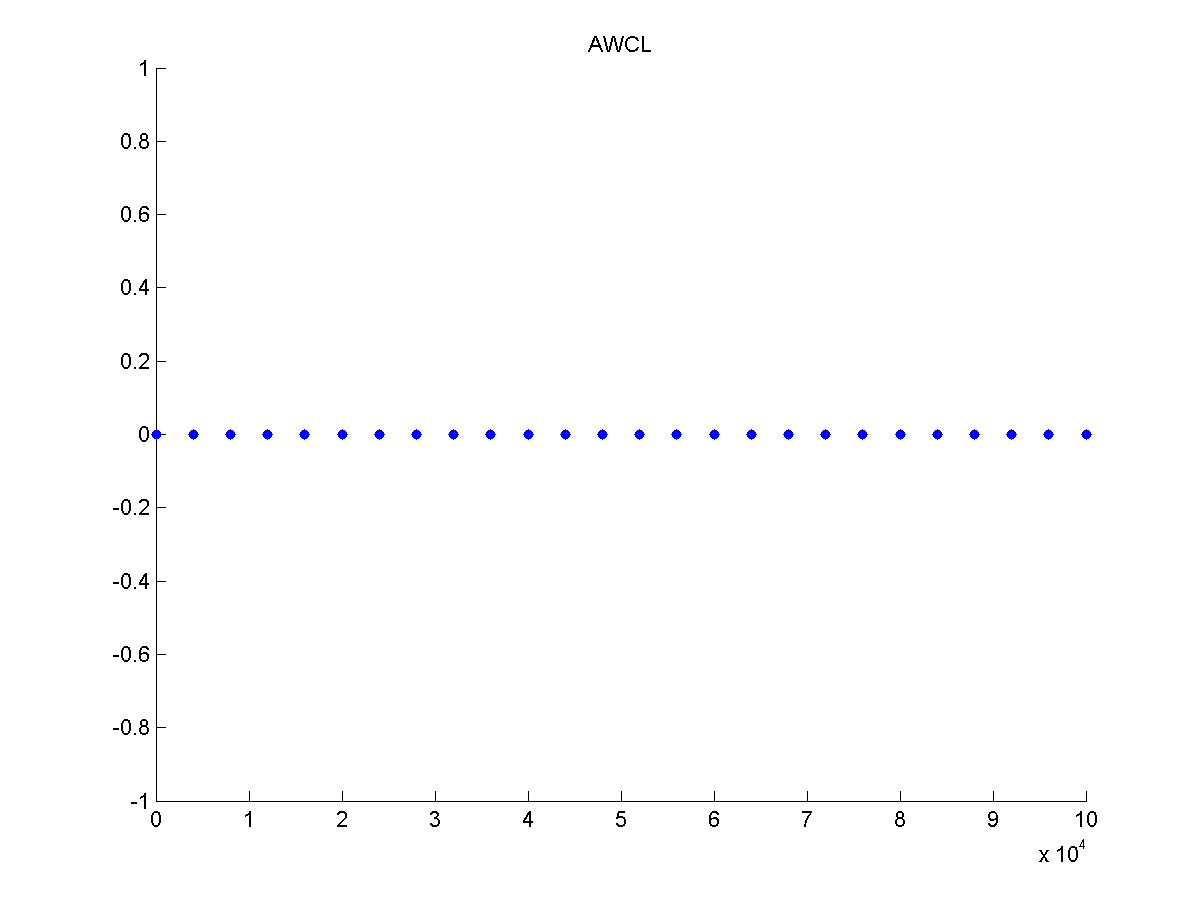

Supplement: Supplementary file 2 [file Presentation2.ZIP › AWCL.png]

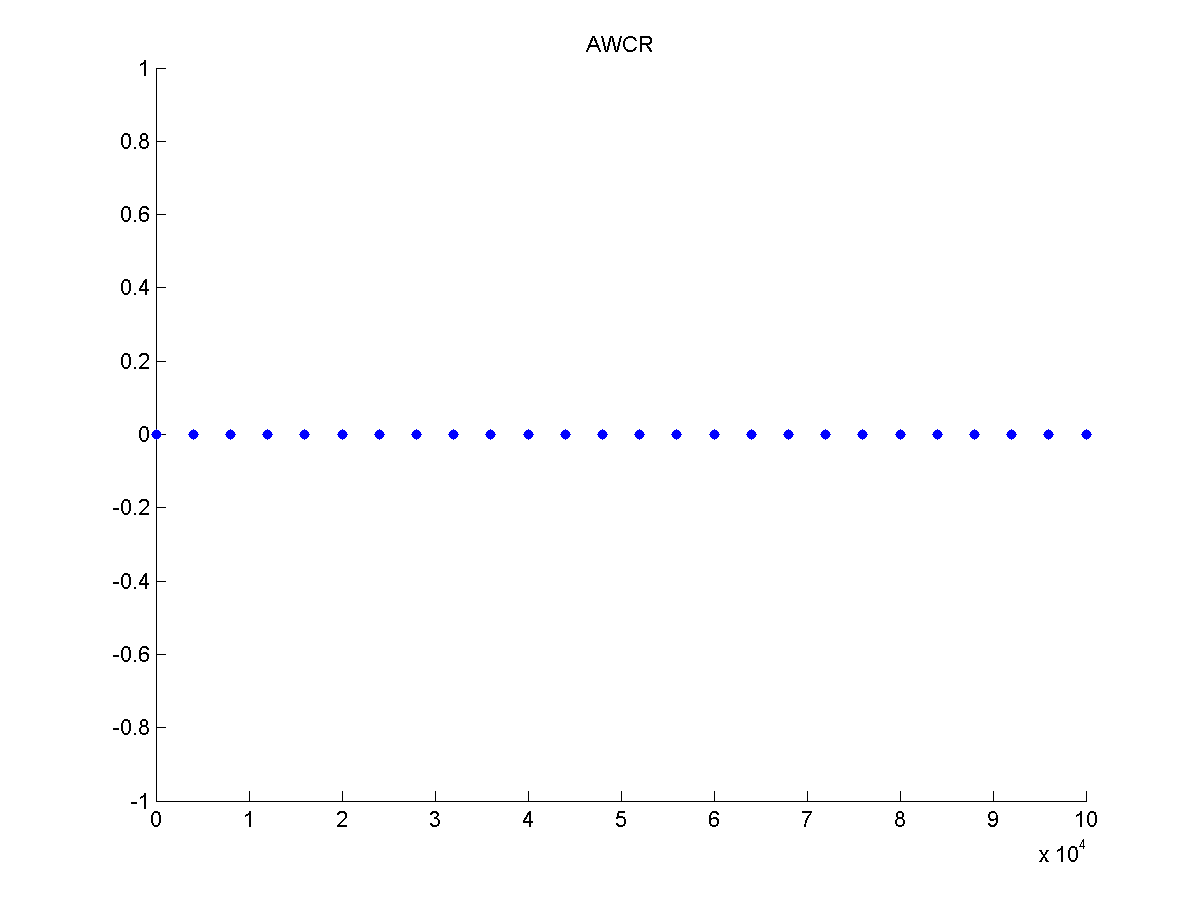

Supplement: Supplementary file 2 [file Presentation2.ZIP › AWCR.png]

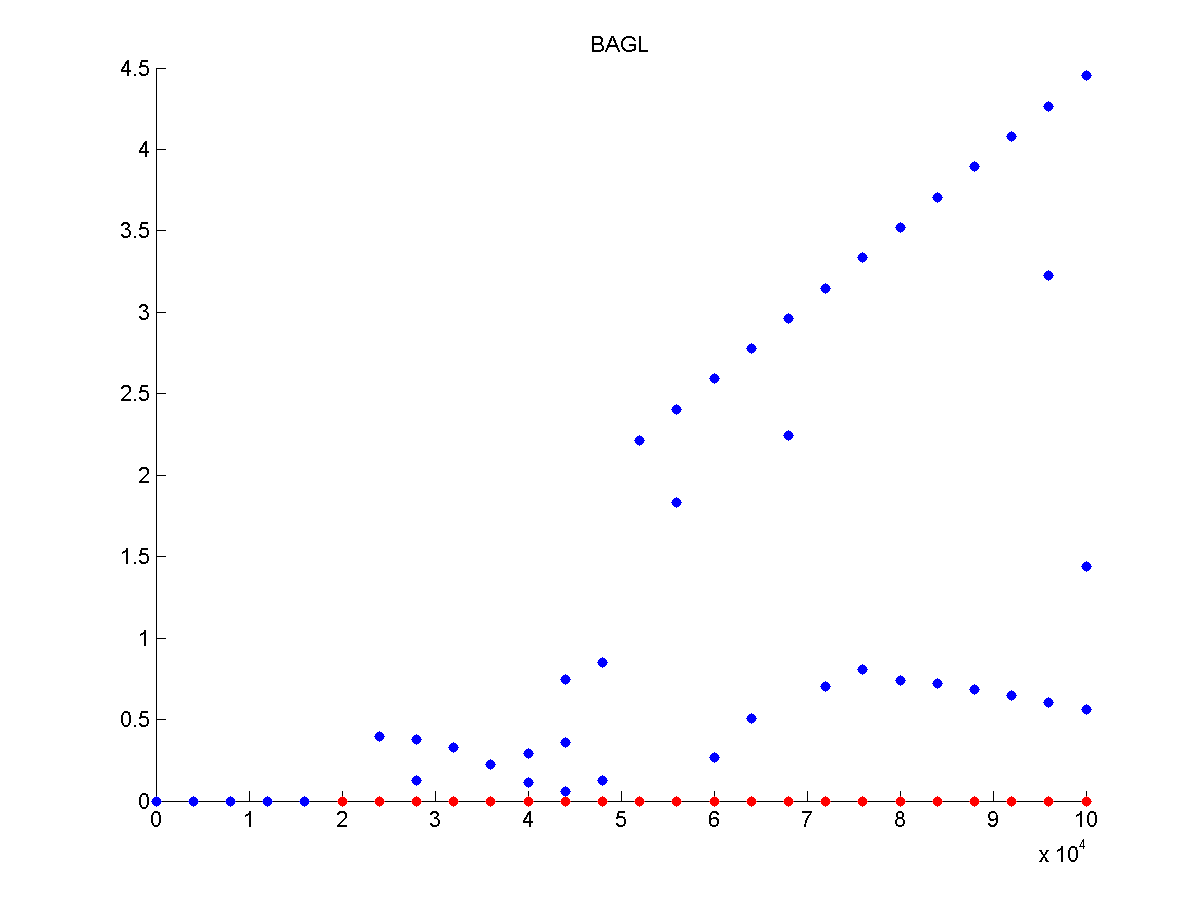

Supplement: Supplementary file 2 [file Presentation2.ZIP › BAGL.png]

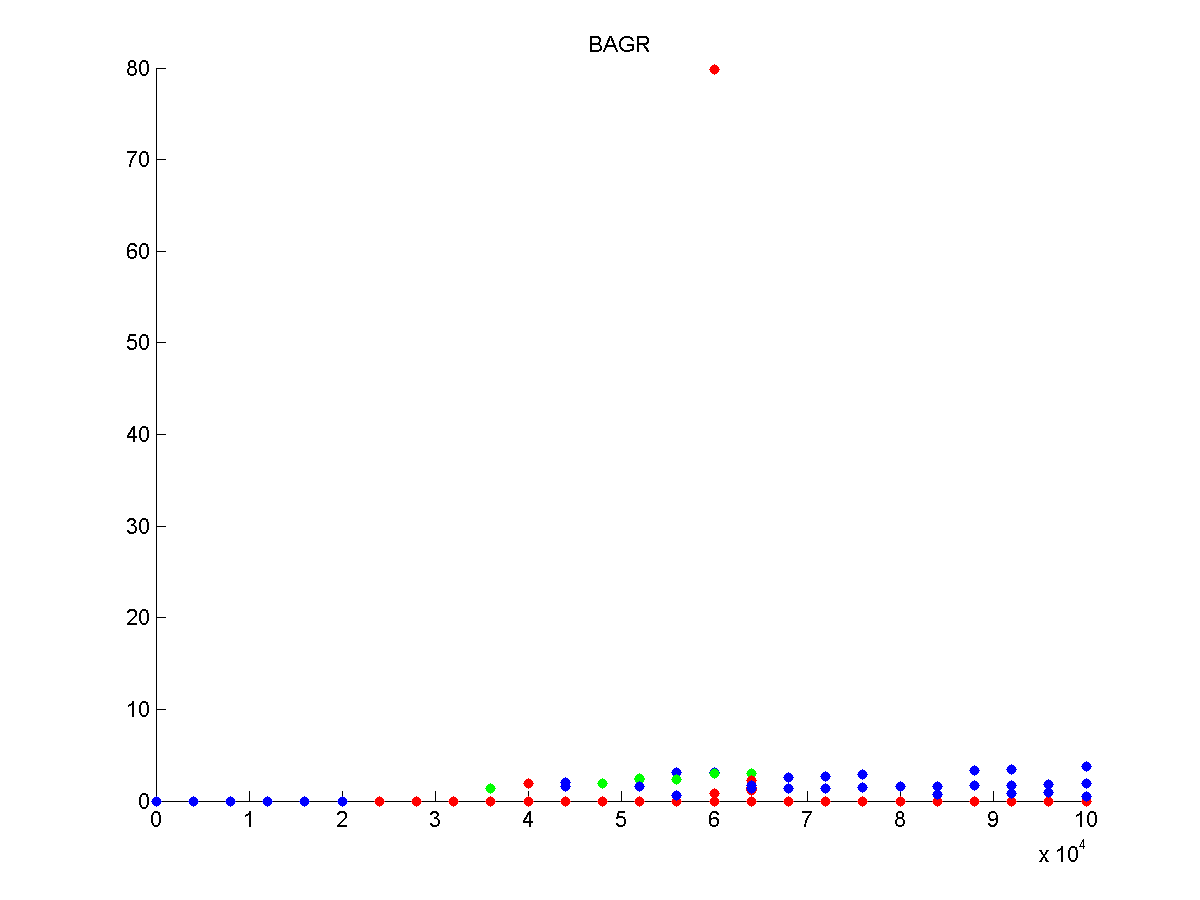

Supplement: Supplementary file 2 [file Presentation2.ZIP › BAGR.png]

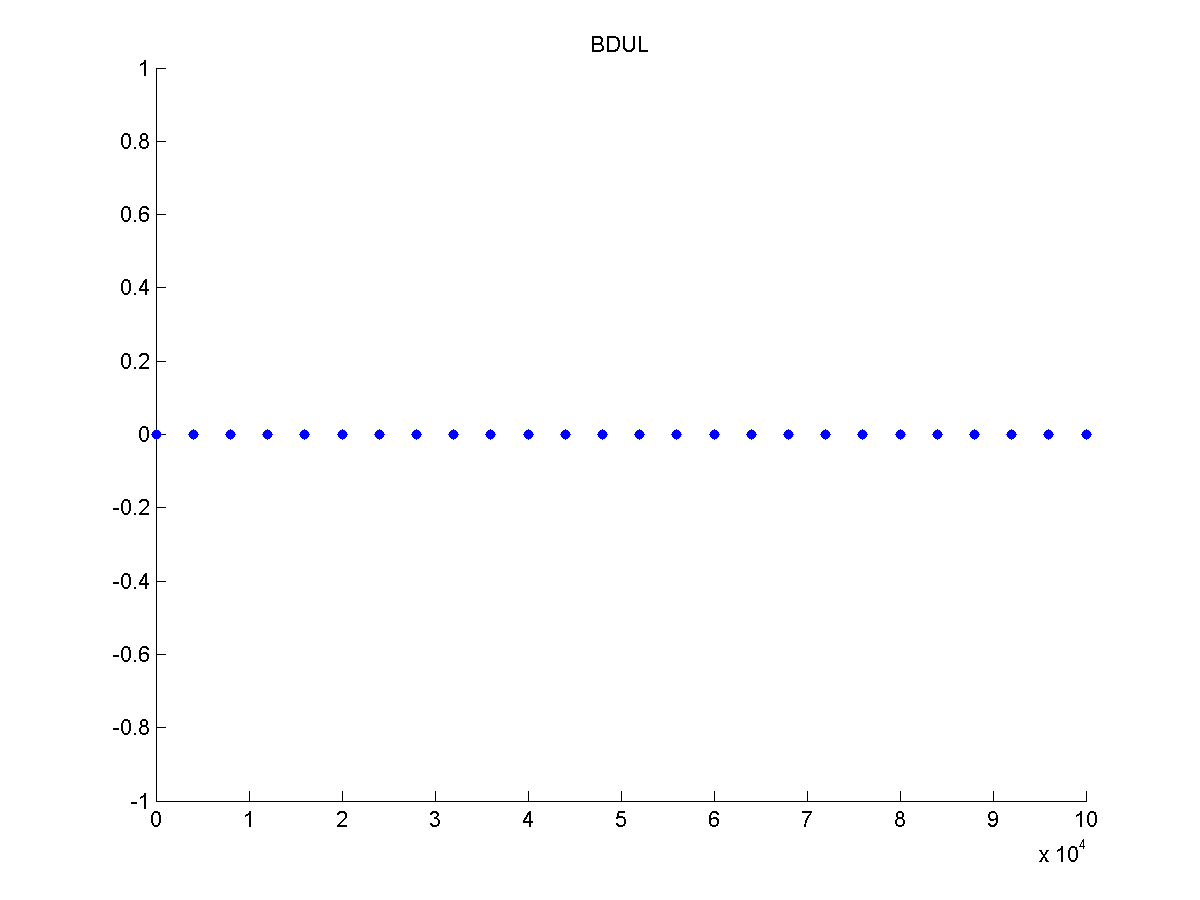

Supplement: Supplementary file 2 [file Presentation2.ZIP › BDUL.png]

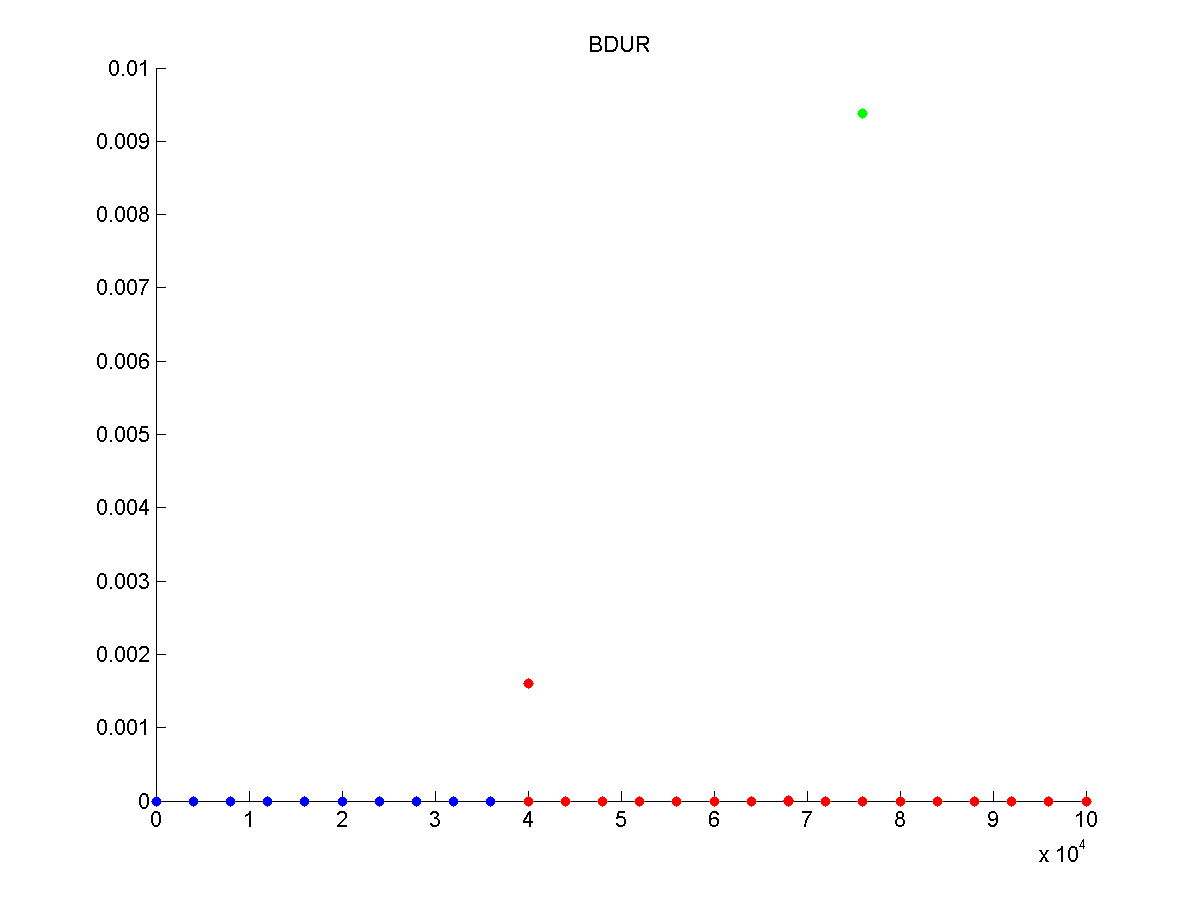

Supplement: Supplementary file 2 [file Presentation2.ZIP › BDUR.png]

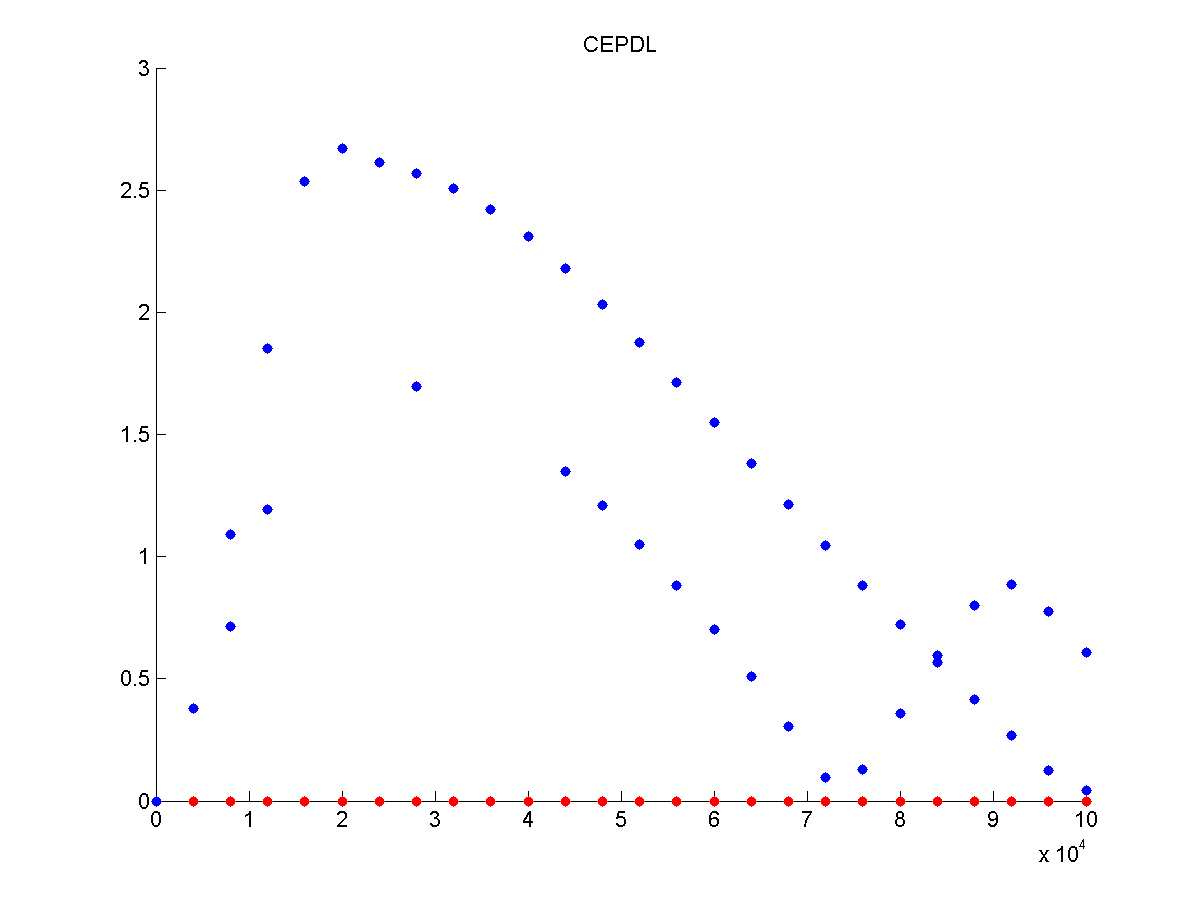

Supplement: Supplementary file 2 [file Presentation2.ZIP › CEPDL.png]

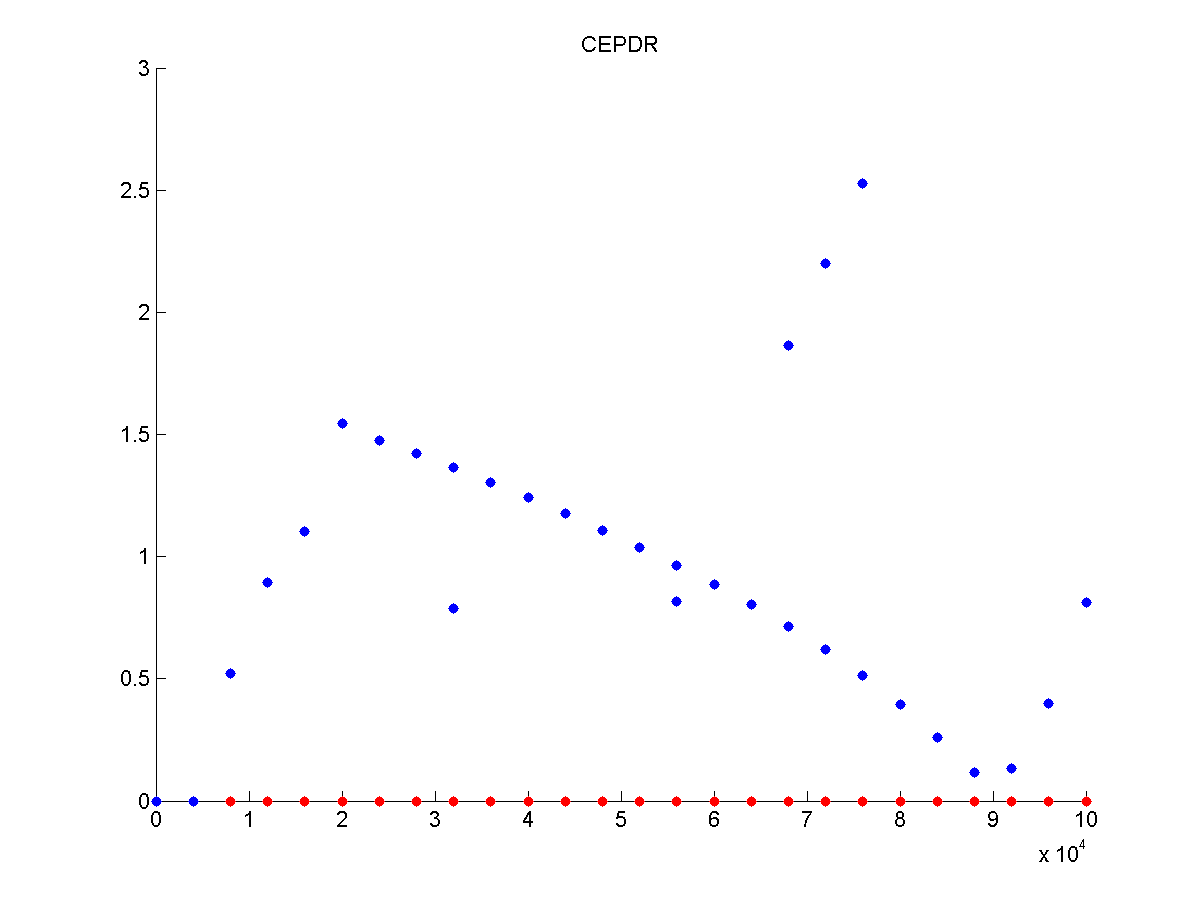

Supplement: Supplementary file 2 [file Presentation2.ZIP › CEPDR.png]

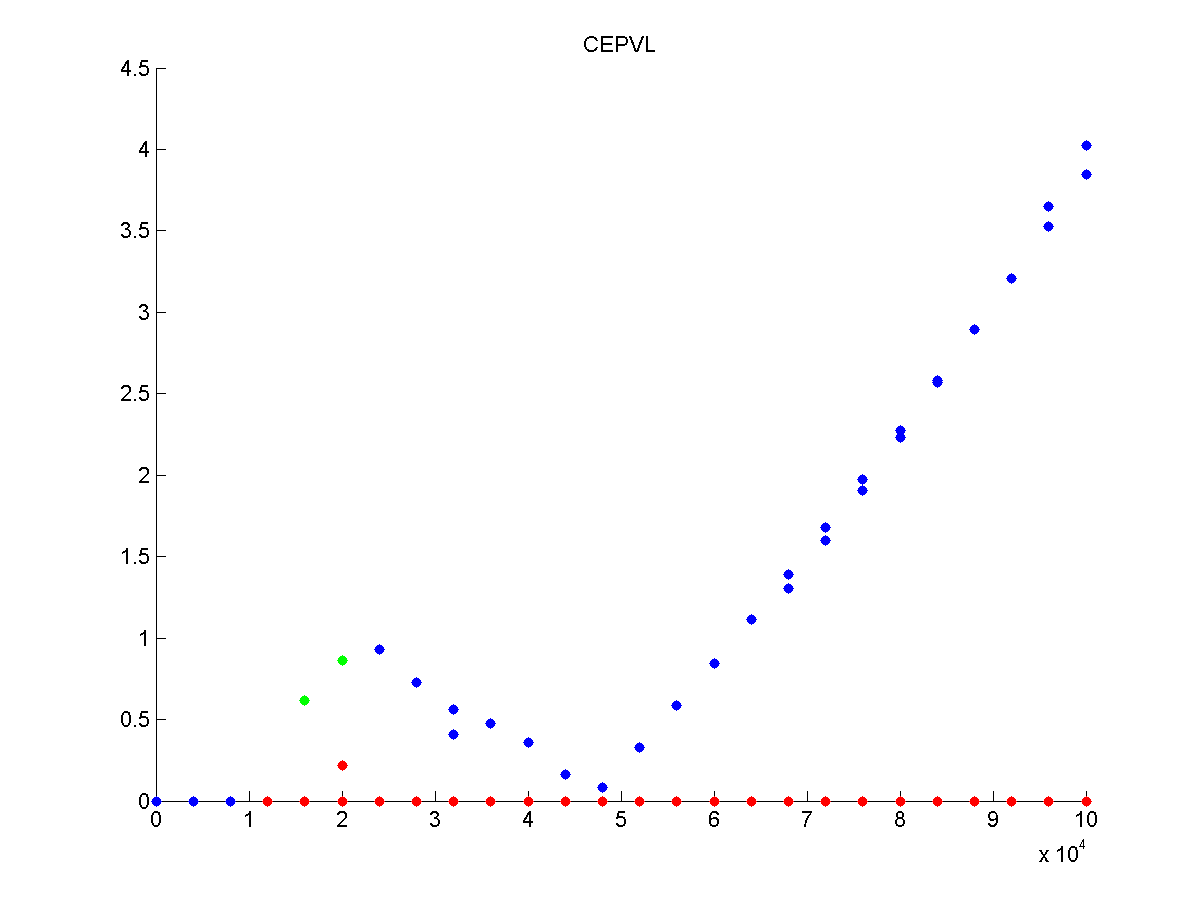

Supplement: Supplementary file 2 [file Presentation2.ZIP › CEPVL.png]

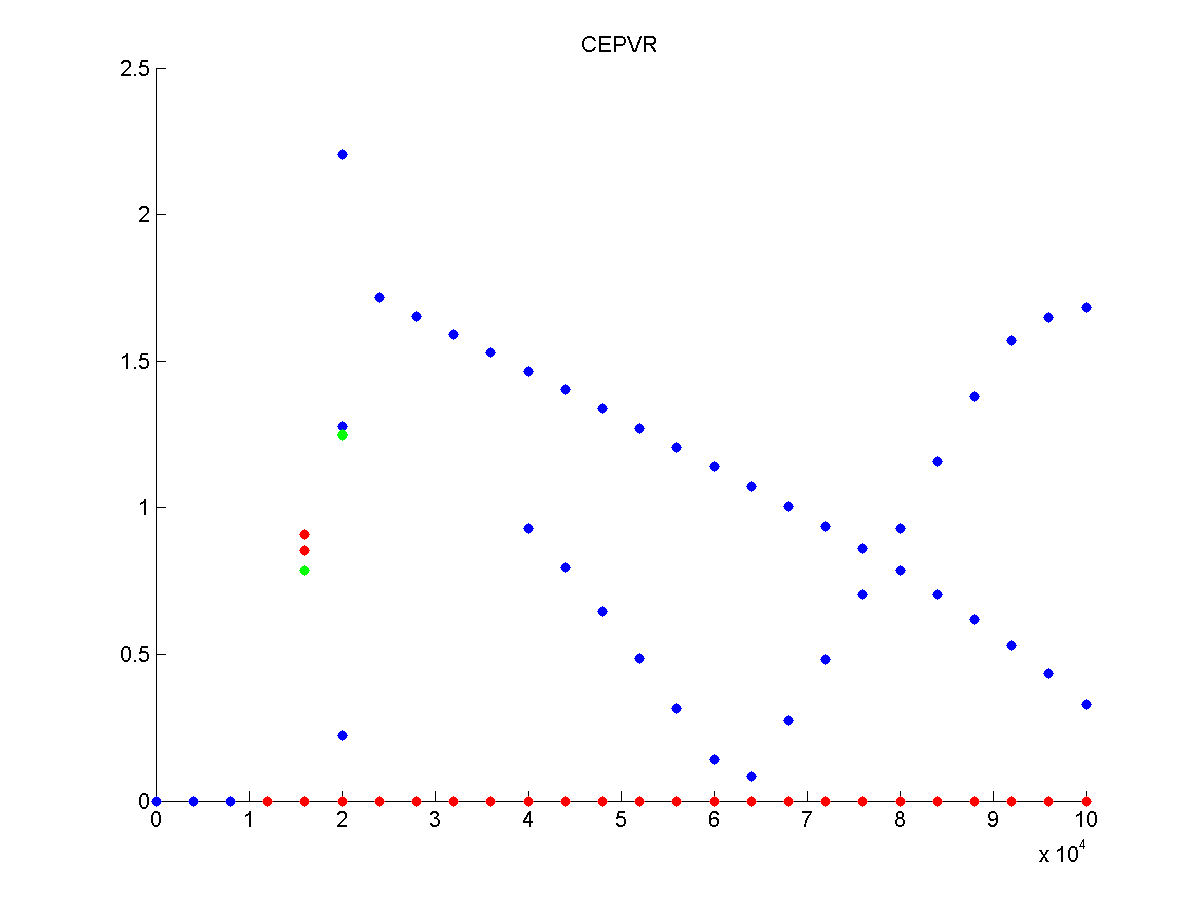

Supplement: Supplementary file 2 [file Presentation2.ZIP › CEPVR.png]

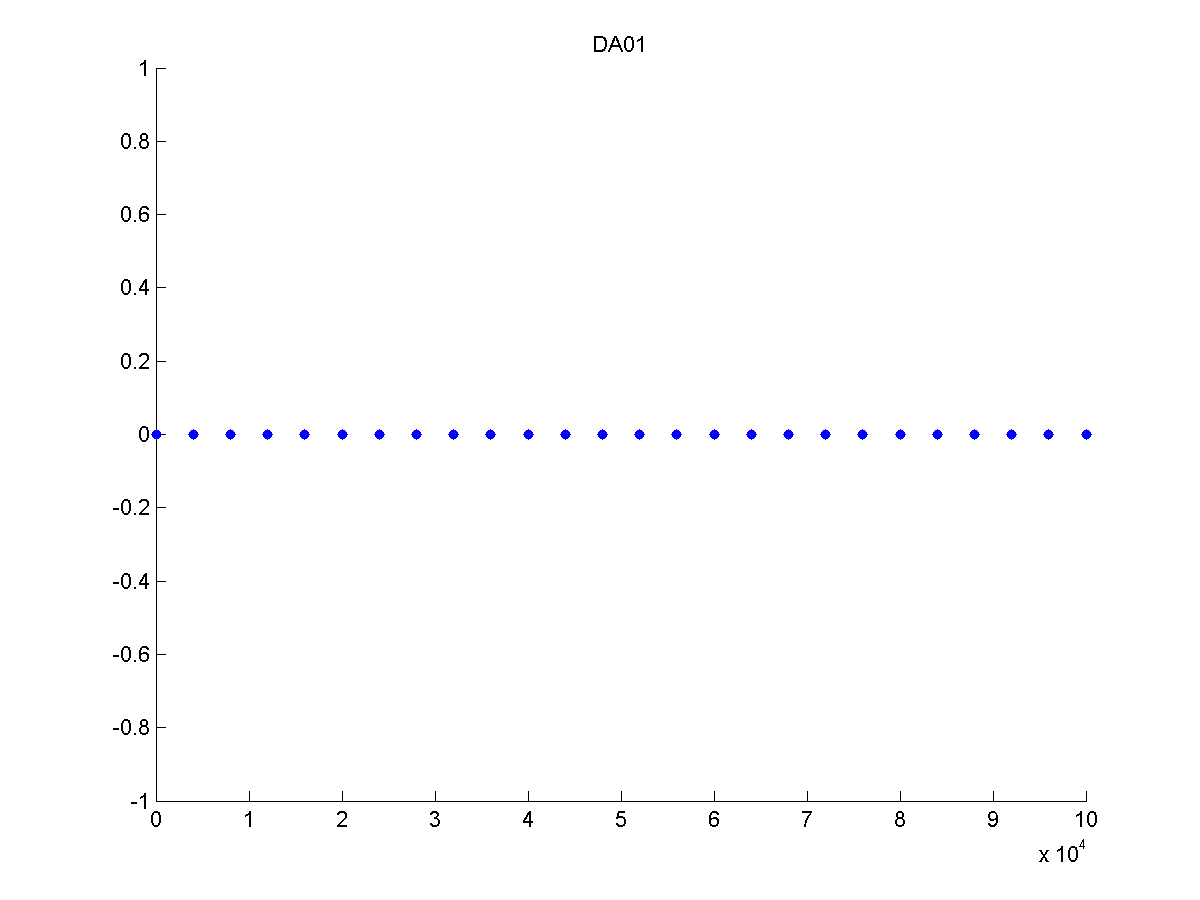

Supplement: Supplementary file 2 [file Presentation2.ZIP › DA01.png]

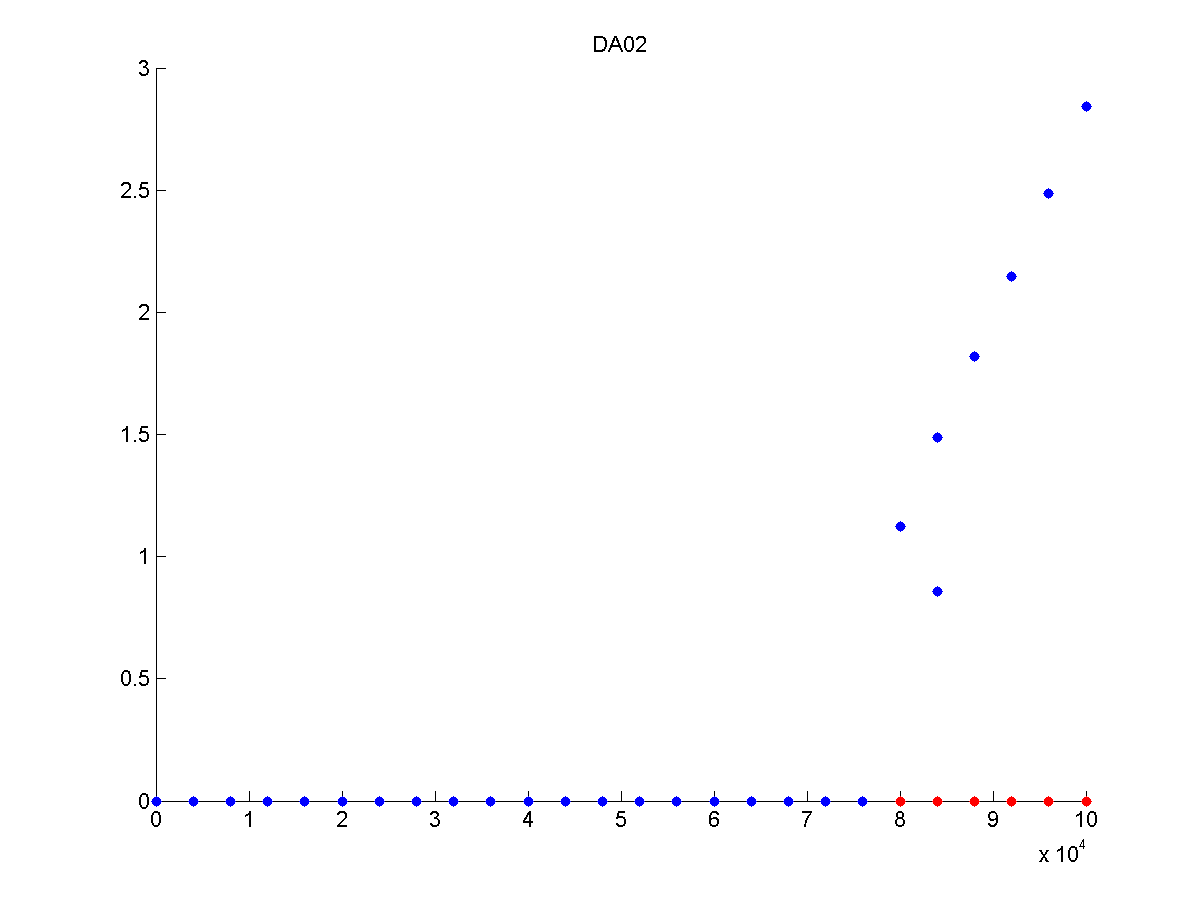

Supplement: Supplementary file 2 [file Presentation2.ZIP › DA02.png]

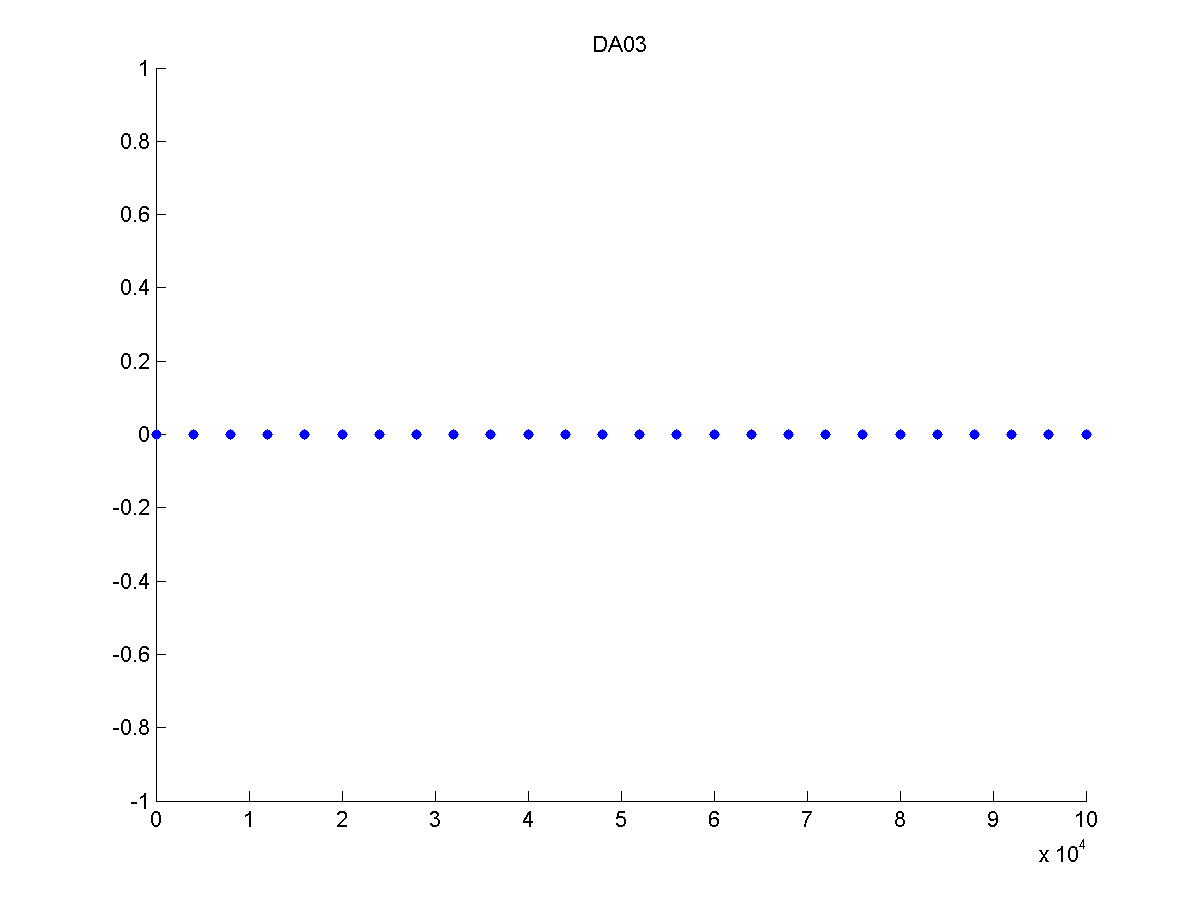

Supplement: Supplementary file 2 [file Presentation2.ZIP › DA03.png]

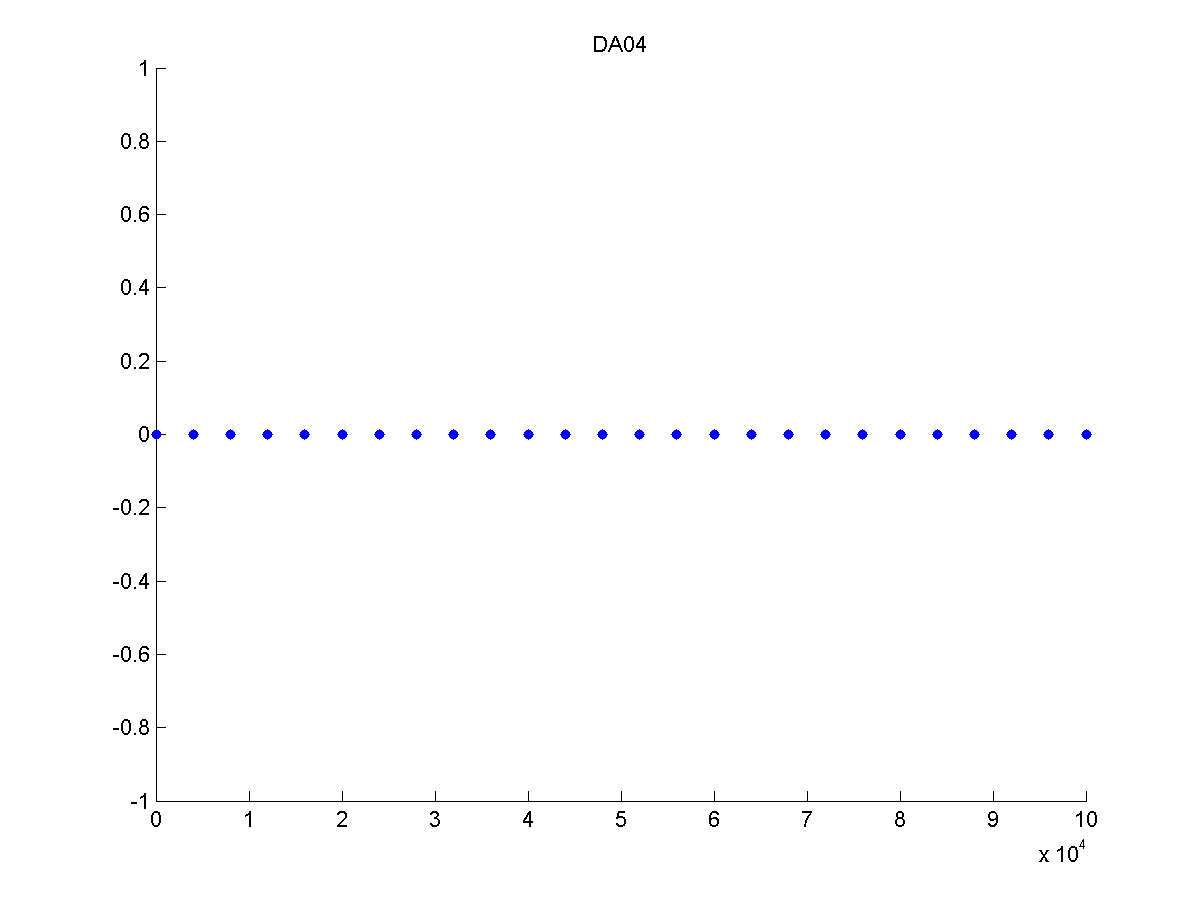

Supplement: Supplementary file 2 [file Presentation2.ZIP › DA04.png]

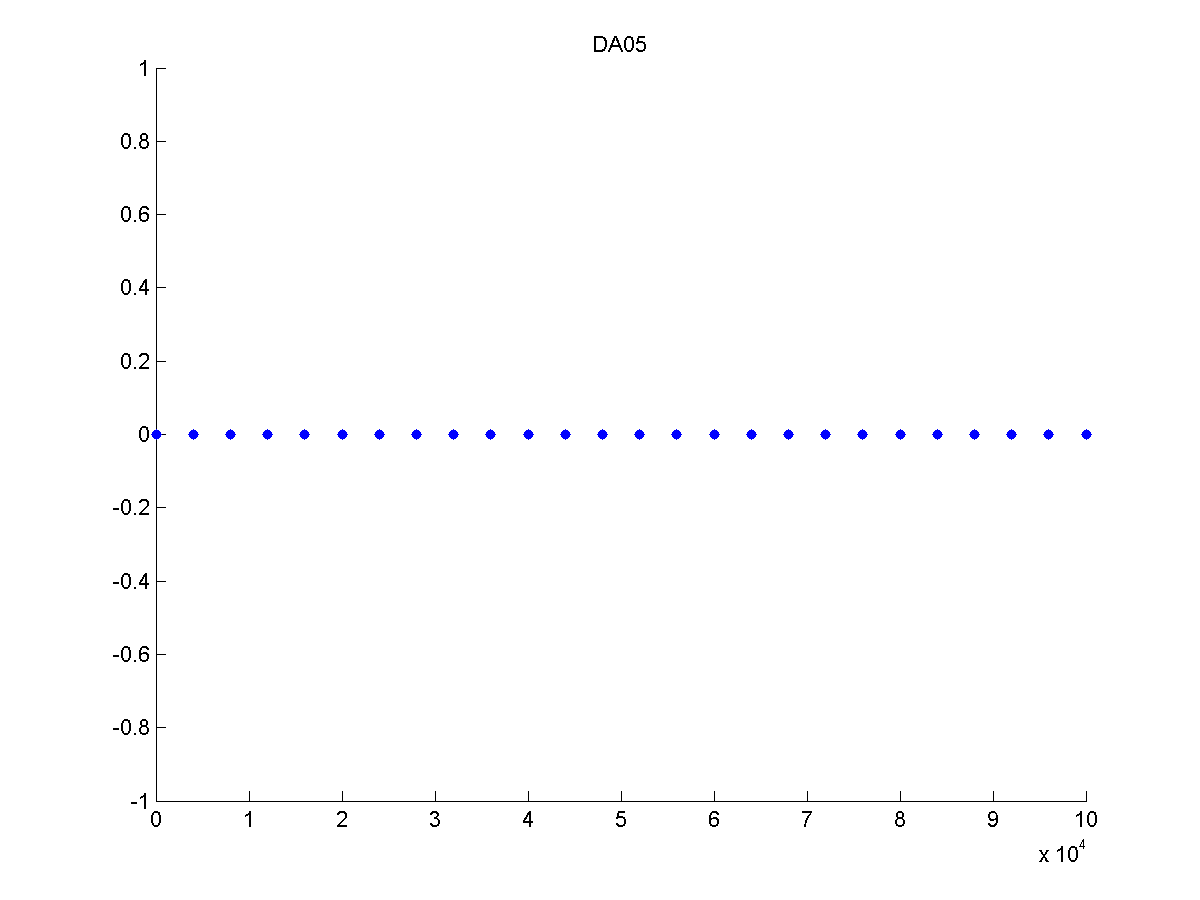

Supplement: Supplementary file 2 [file Presentation2.ZIP › DA05.png]

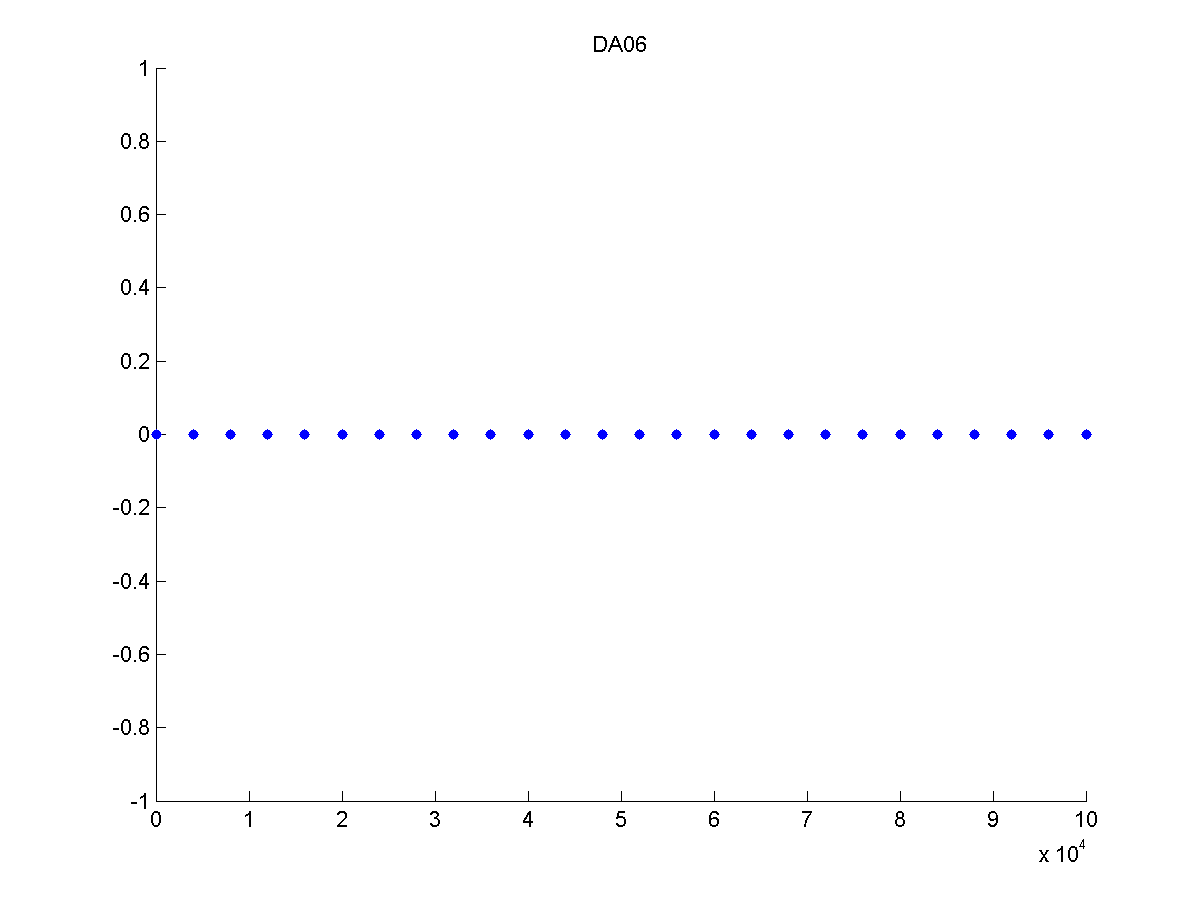

Supplement: Supplementary file 2 [file Presentation2.ZIP › DA06.png]

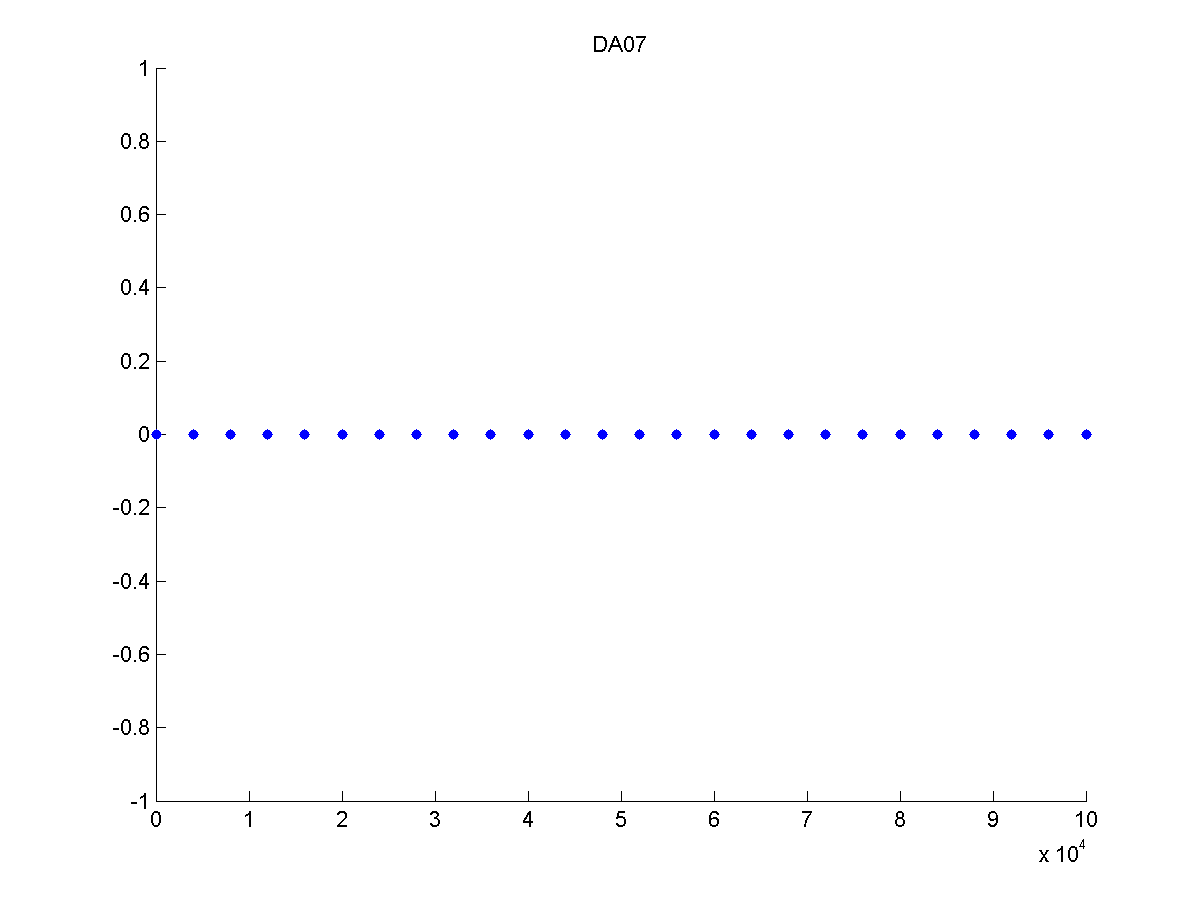

Supplement: Supplementary file 2 [file Presentation2.ZIP › DA07.png]

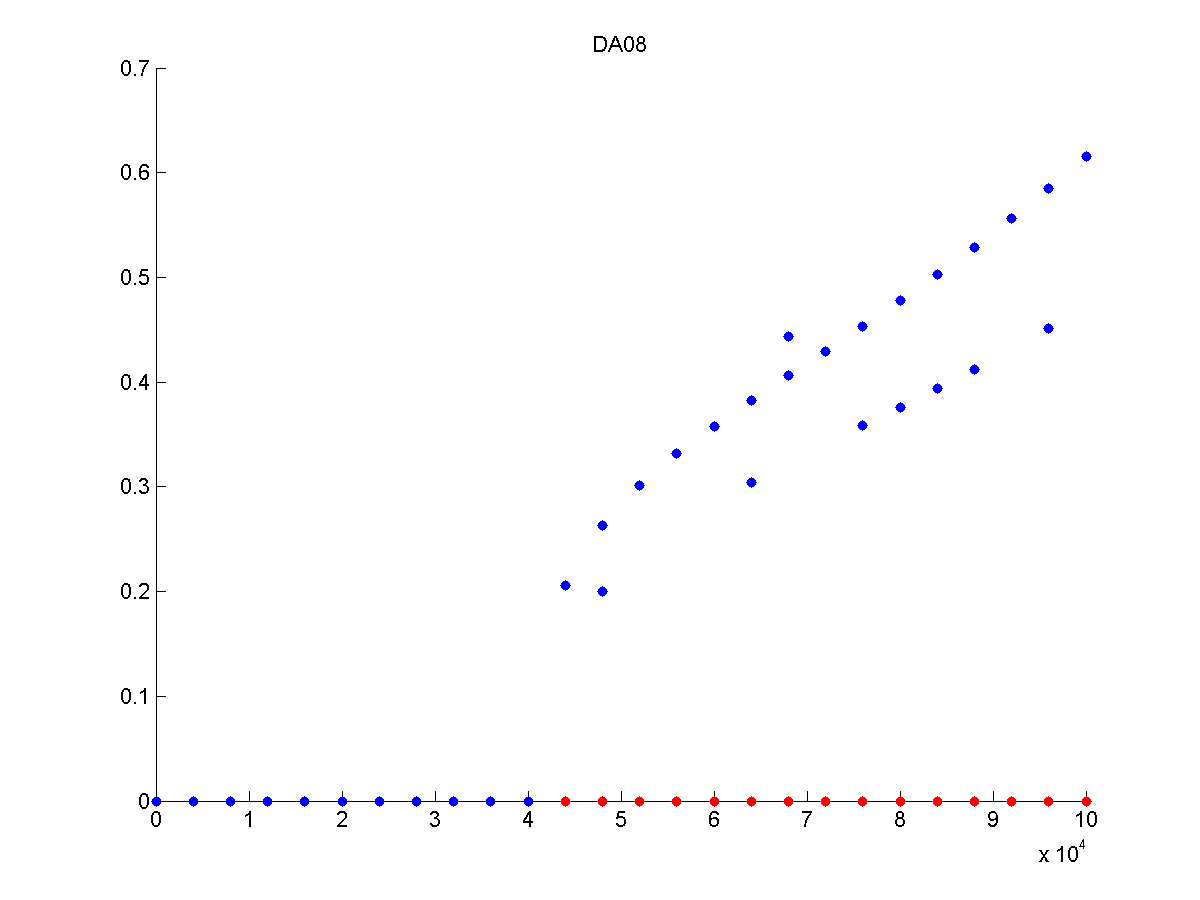

Supplement: Supplementary file 2 [file Presentation2.ZIP › DA08.png]

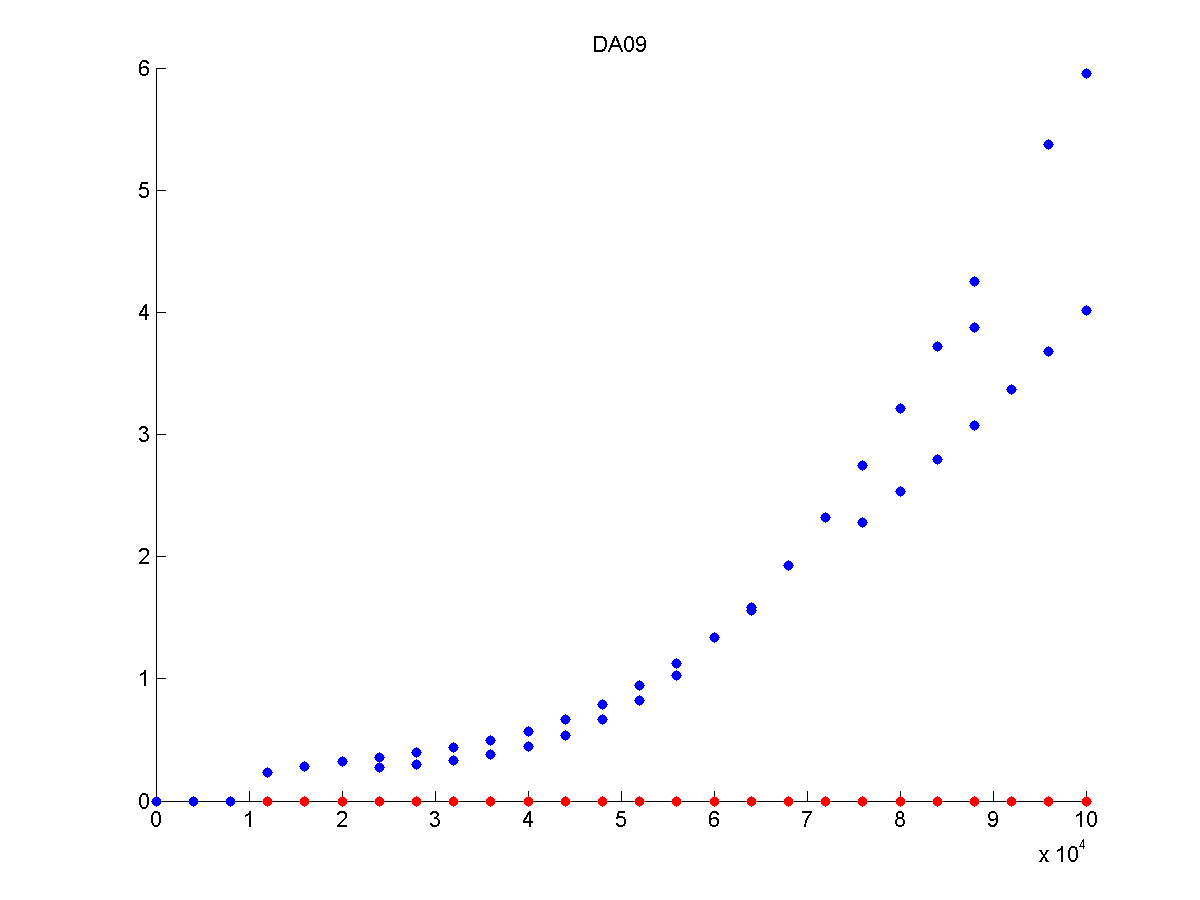

Supplement: Supplementary file 2 [file Presentation2.ZIP › DA09.png]

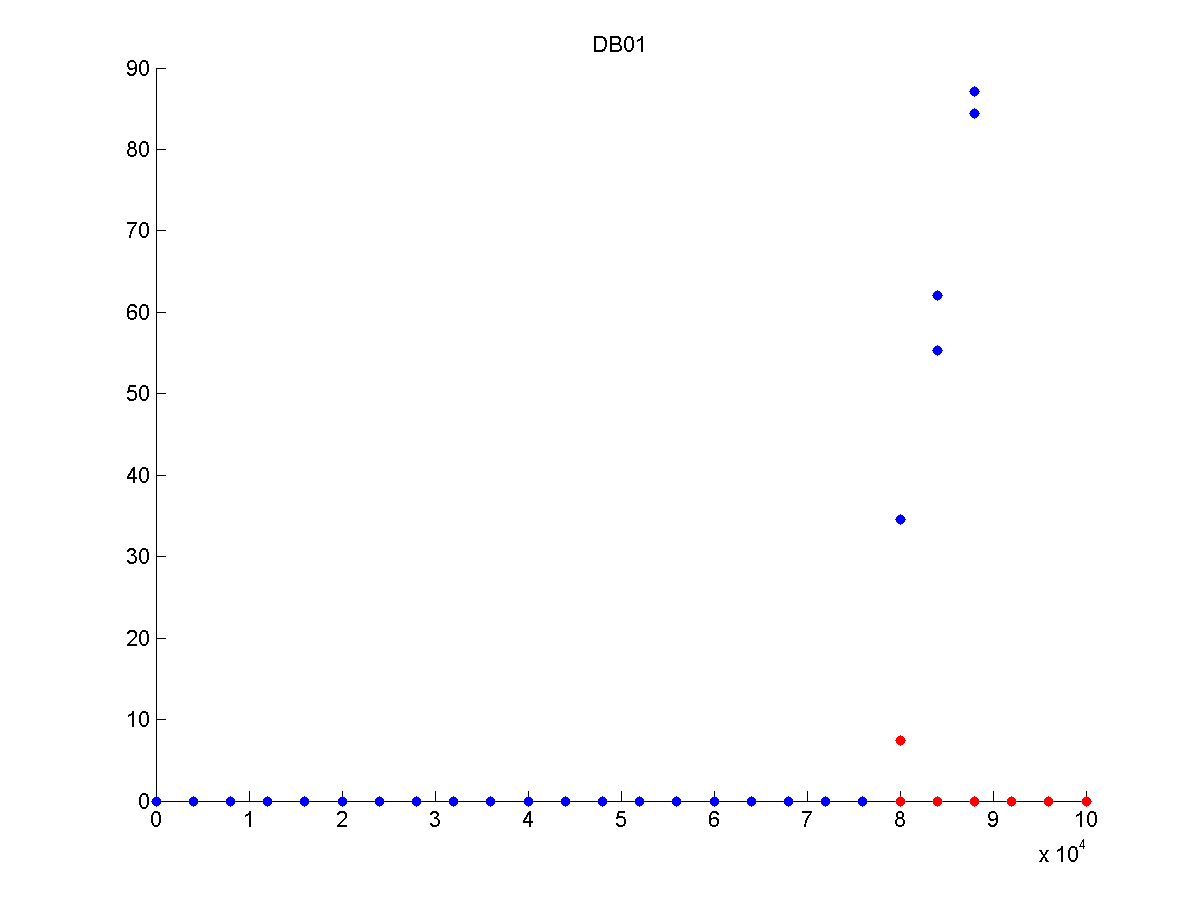

Supplement: Supplementary file 2 [file Presentation2.ZIP › DB01.png]

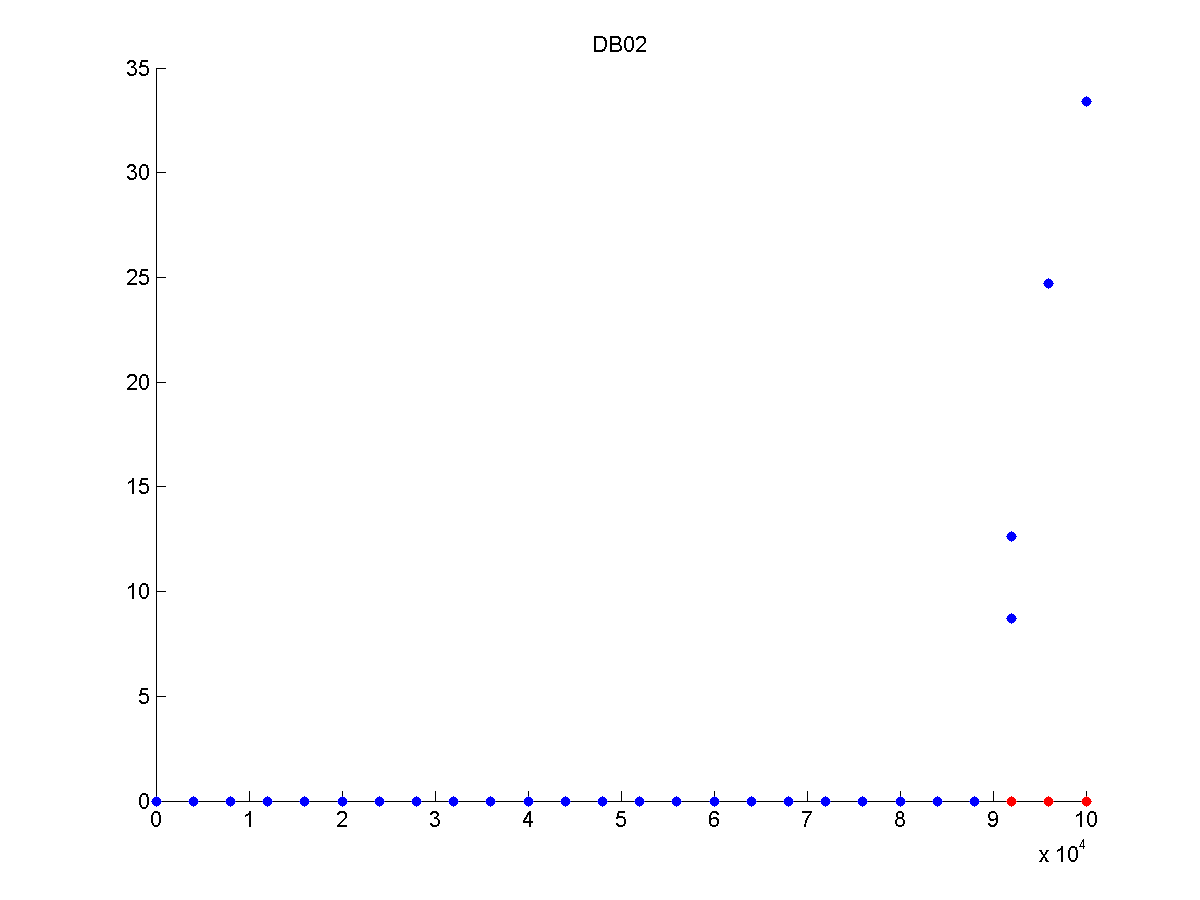

Supplement: Supplementary file 2 [file Presentation2.ZIP › DB02.png]

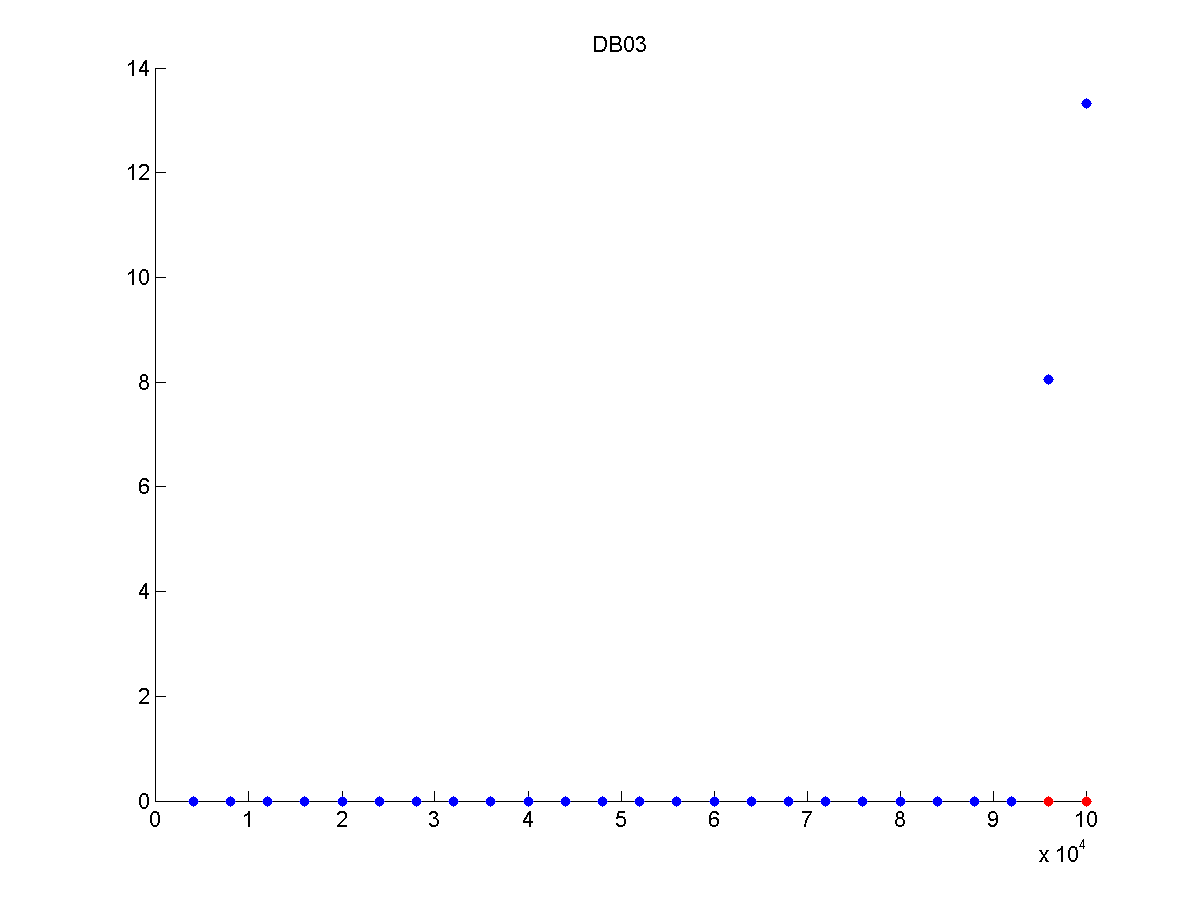

Supplement: Supplementary file 2 [file Presentation2.ZIP › DB03.png]

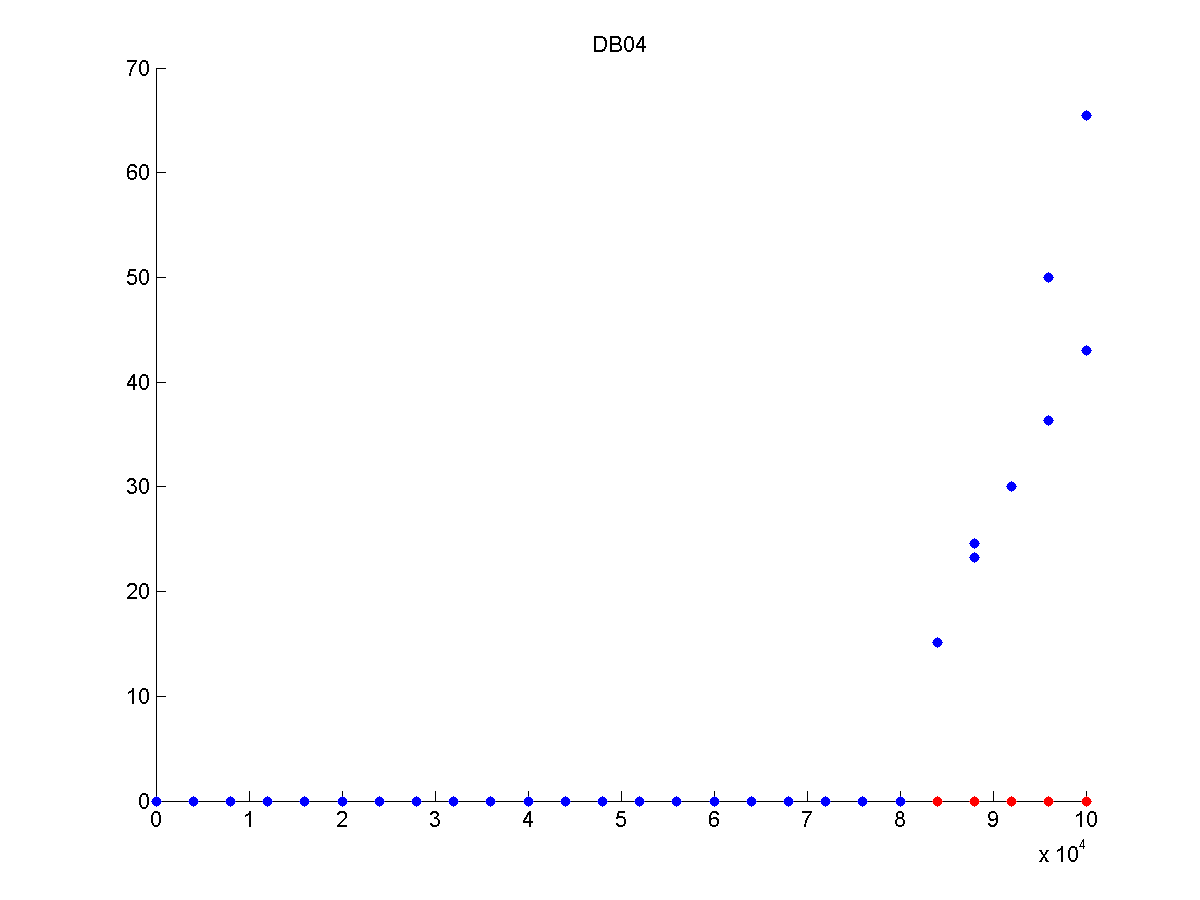

Supplement: Supplementary file 2 [file Presentation2.ZIP › DB04.png]

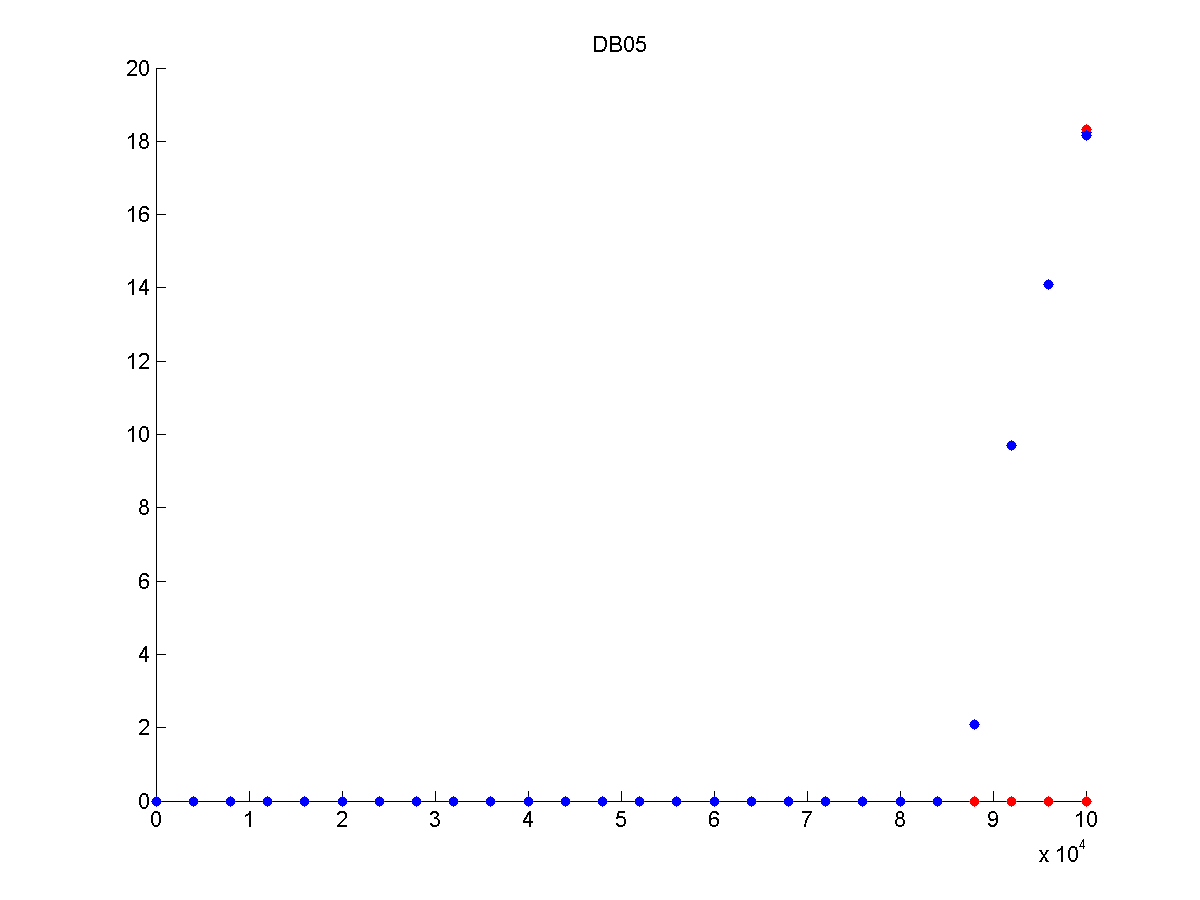

Supplement: Supplementary file 2 [file Presentation2.ZIP › DB05.png]
